# Isolation of phosphorus-hyperaccumulating microalgae from revolving algal biofilm (RAB) wastewater treatment systems

**DOI:** 10.3389/fmicb.2023.1219318

**Published:** 2023-07-17

**Authors:** Eric Schaedig, Michael Cantrell, Chris Urban, Xuefei Zhao, Drew Greene, Jens Dancer, Michael Gross, Jacob Sebesta, Katherine J. Chou, Jonathan Grabowy, Martin Gross, Kuldip Kumar, Jianping Yu

**Affiliations:** ^1^National Renewable Energy Laboratory, Biosciences Center, Golden, CO, United States; ^2^Gross-Wen Technologies, Slater, IA, United States; ^3^Metropolitan Water Reclamation District of Greater Chicago, Chicago, IL, United States

**Keywords:** microalgae, algae, phosphorus, polyphosphate, wastewater, revolving algal biofilm, bioprospecting

## Abstract

Excess phosphorus (P) in wastewater effluent poses a serious threat to aquatic ecosystems and can spur harmful algal blooms. Revolving algal biofilm (RAB) systems are an emerging technology to recover P from wastewater before discharge into aquatic ecosystems. In RAB systems, a community of microalgae take up and store wastewater P as polyphosphate as they grow in a partially submerged revolving biofilm, which may then be harvested and dried for use as fertilizer in lieu of mined phosphate rock. In this work, we isolated and characterized a total of 101 microalgae strains from active RAB systems across the US Midwest, including 82 green algae, 9 diatoms, and 10 cyanobacteria. Strains were identified by microscopy and 16S/18S ribosomal DNA sequencing, cryopreserved, and screened for elevated P content (as polyphosphate). Seven isolated strains possessed at least 50% more polyphosphate by cell dry weight than a microalgae consortium from a RAB system, with the top strain accumulating nearly threefold more polyphosphate. These top P-hyperaccumulating strains include the green alga *Chlamydomonas pulvinata* TCF-48 g and the diatoms *Eolimna minima* TCF-3d and *Craticula molestiformis* TCF-8d, possessing 11.4, 12.7, and 14.0% polyphosphate by cell dry weight, respectively. As a preliminary test of strain application for recovering P, *Chlamydomonas pulvinata* TCF-48 g was reinoculated into a bench-scale RAB system containing Bold basal medium. The strain successfully recolonized the system and recovered twofold more P from the medium than a microalgae consortium from a RAB system treating municipal wastewater. These isolated P-hyperaccumulating microalgae may have broad applications in resource recovery from various waste streams, including improving P removal from wastewater.

## 1. Introduction

Microalgae are an emerging tool for phosphorus (P) recovery and reuse technology. Phosphorus is a vital nutrient for all life on Earth, playing essential roles in biomolecule synthesis, cellular signaling, and energy storage. Anthropogenic manipulation of the P cycle for agriculture and other applications has led to global imbalances in P distribution (Elser and Bennett, 2011; Yuan et al., 2018). These imbalances are particularly pronounced in aquatic ecosystems, which receive excess P via several pathways including runoff from agricultural land and wastewater effluent discharge into waterways. These excess P inputs lead to eutrophication of aquatic ecosystems that may spur harmful algal blooms (Wurtsbaugh et al., 2019). In response to this persistent and growing issue, United States federal and state agencies overseeing water quality have tightened P emission limits in recent years, and these limits are expected to become increasingly strict (Fox, 2022). Conversely, human processes such as agriculture and manufacturing currently rely heavily on finite geological stores of high-grade phosphate rock (Nedelciu et al., 2020). New technologies are critically needed to not only limit P emissions to the environment but also to reclaim P from waste streams to alleviate global reliance on finite phosphate rock resources.

Wastewater is an attractive target for the development of such nutrient reclamation technologies because of its nutrient-rich composition. A typical conventional wastewater treatment plant can remove only 10% of total P from raw municipal influent through solids settling and 30% of total P through biological metabolism in conventional activated sludge (Cornel and Schaum, 2009). However, more recent data from conventional activated sludge treatment plants operated by the Metropolitan Water Reclamation District of Greater Chicago (MWRD Chicago) shows total P removal by activated sludge to range between 42 and 57% (MWRD, 2023). These removal rates are not sufficient to meet discharge limits, and the remaining P is often removed from wastewater as needed by precipitation with trivalent metal cations due to the high efficiency and low technical demand of the method. However, these phosphate precipitates are difficult to separate and reuse as bioavailable P, incurring a waste disposal burden on wastewater treatment plants and limiting the sustainability of this practice (Bunce et al., 2018). Phosphorus precipitation as magnesium ammonium phosphate (struvite) may also be used to efficiently remove P from water, and the captured P may be reused (Siciliano et al., 2020). However, issues with this method, including high operational costs and energy consumption, have slowed the widespread adoption of struvite precipitation (Sena and Hicks, 2018). Enhanced biological phosphorus removal may also be used to remove P from wastewater, and MWRD Chicago facilities operating the technology have demonstrated total P removal rates between 84 and 92% (MWRD, 2023). However, this method is operationally complex, energy-intensive, prone to fluctuations, and often restricted to large wastewater treatment facilities (Bunce et al., 2018).

Microalgae, or microscopic algae, are an emerging and powerful tool to reclaim P from wastewater (Slocombe et al., 2020). Microalgae are capable of growing in wastewater and take up P from the wastewater as a critical nutrient for biomolecule synthesis. However, microalgae often take up P at levels surpassing their nutritional needs (commonly referred to as “luxury uptake”). In the luxury uptake process, microalgae store excess P intracellularly as polyphosphate (polyP), a high molecular weight polymer of inorganic phosphate. This process has made microalgae a topic of interest in biological wastewater P recovery efforts and has previously been reviewed in depth (Sanz-Luque et al., 2020).

Microalgae have long been used to treat wastewater and remove nutrients and organic pollutants in open pond systems (Wollmann et al., 2019; Plöhn et al., 2021; Wu et al., 2022). More recently, revolving algal biofilm (RAB) systems have been developed to improve the efficiency of nutrient removal from wastewater by optimizing nutritional and light conditions to increase microalgal growth (Zhao et al., 2018). Microalgae present in the wastewater colonize the RAB system to form an attached biofilm on a vertical semi-submerged revolving belt. Exposure of the biofilm to the atmosphere allows gas exchange and light penetration into the biofilm, while intermittent submersion in the wastewater allows for adsorption and cellular uptake of P and other pollutants by the microalgae as they grow. The nutrient-rich microalgal biomass may then be harvested by scraping and used as fertilizer or as feedstock to produce biofuel or other bioproducts (Lindsey et al., 2021). The RAB system design has been optimized in recent years to improve rates of nutrient removal and biomass production through an engineering approach (Gross et al., 2013, 2015, 2016; Gross and Wen, 2014). However, RAB systems have not yet been optimized from a biological perspective. Manipulating the microalgae species that colonize and grow in these systems may lead to improvements in nutrient recovery, biomass production, and other performance parameters.

Luxury uptake is reported to be dynamic and varies across different species of microalgae (Sanz-Luque et al., 2020), suggesting that RAB system P recovery may be biologically enhanced by introducing strains of microalgae capable of hyperaccumulating P. Many studies have demonstrated elevated P levels attributable to luxury uptake in both environmental microalgal biomass and individual strains of green algae and cyanobacteria cultivated in wastewater (Powell, 2009; Powell et al., 2009; Lynch et al., 2015; Lavrinovičs et al., 2020, 2021). For example, Lavrinovičs et al. (2020) reported transient polyP levels of up to 24.5% cell dry weight (CDW) in the green alga *Chlorella protothecoides* when cultivated in primary municipal wastewater. However, RAB systems cultivate microalgae in a unique revolving aerial biofilm system that may be inhospitable to many previously identified P-hyperaccumulating strains. Thus, the isolation and characterization of new P-hyperaccumulating microalgae strains native to the RAB system is prudent to ensure the isolates are capable of recolonization, a necessary step in improving the P removal efficiency of the system.

Here, we seek to improve RAB system performance by building a culture collection of microalgae isolated from RAB systems treating wastewater across the United States Midwest with the aim of isolating P-hyperaccumulating microalgae. Isolates were characterized by microscopy, identified by 16S/18S ribosomal DNA (rDNA) sequencing, screened for elevated polyP content, and cryopreserved for future use. As a preliminary proof of principle test that isolated strains may be reintroduced to enhance system performance, two isolated strains were cultivated in bench-scale RAB systems containing artificial medium, and biomass productivity and P uptake were measured in comparison to a consortium of microalgae sampled from a RAB system treating municipal wastewater.

## 2. Materials and methods

### 2.1. Media composition, sample collection, and microalgae isolation

A variety of media compositions were used to isolate and cultivate microalgae from RAB samples. These include Bold basal medium (BBM) (Nichols and Bold, 1965) supplemented with 100 μg L^−1^ cyanocobalamin (vitamin B12; Sigma) (Croft et al., 2006), BG-11 (Rippka et al., 1979), and synthetic wastewater medium (GWT-SE) (Supplementary Table S1). GWT-SE medium composition was modified from Voumard et al. (2019) to mimic the physicochemical properties and nutrient content of secondary municipal sewage effluent (Supplementary Table S2). Liquid medium was autoclaved or filter-sterilized with a 0.22 μm polyethersulfone bottle-top filter (Corning). Solid medium was prepared by the addition of agar (BD, 1.5% w/v final concentration). Antibiotics, buffers, and other reagents were sterilized with a 0.22 μm polyethersulfone bottle-top filter or a 0.22 μm polyvinylidene fluoride syringe filter (NEST Scientific).

**Figure 1:**
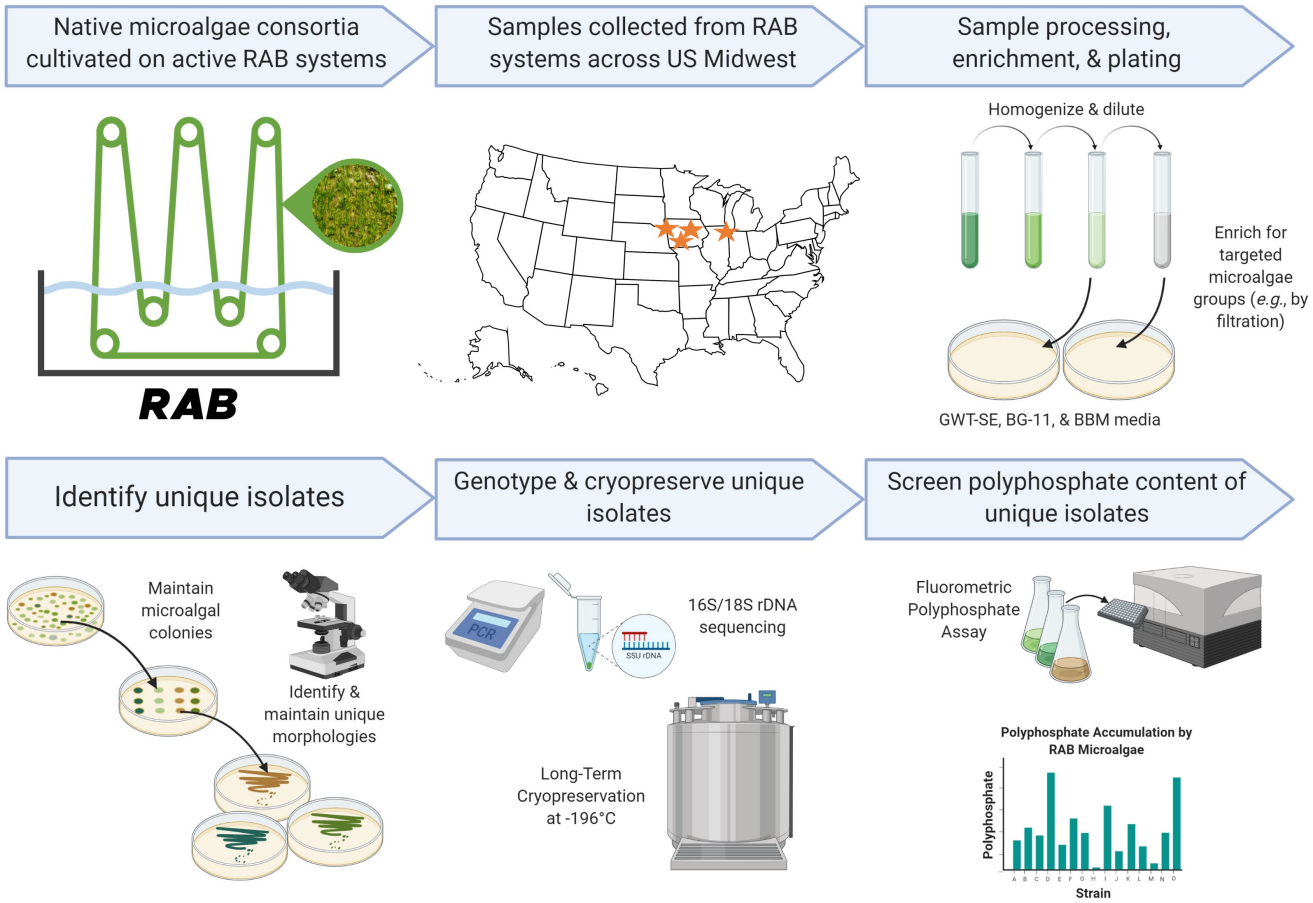
Research pipeline of the present study. Created in part with Biorender.com.

RAB samples were collected from active RAB systems across the United States Midwest in December 2020, February 2021, and April 2021. Collected samples were shipped overnight in 50 mL conical polypropylene tubes to the National Renewable Energy Laboratory in Golden, Colorado, United States for use in this study (Figure 1). Upon receipt, RAB samples were stirred, and 1–5 mL of sample was transferred to 125–250 mL borosilicate Erlenmeyer flasks containing liquid GWT-SE medium and placed under white LED panel lights at 70–130 μE m^−2^ s^−1^ for long-term maintenance. All cultivation in this study was conducted at room temperature. RAB samples and flask cultures were imaged via brightfield microscopy with a Carl Zeiss Axio Scope.A1 microscope. Microscopic images were taken with an AxioCam MRc digital camera (Carl Zeiss) and AxioVision software (Carl Zeiss; v4.8.2).

To isolate unicellular microalgae, RAB samples and/or flask cultures were homogenized by vortex mixing or stirring and serially diluted 1:10 into GWT-SE medium to a final dilution of 1:1,000. 100 μL of the dilutions were plated onto solid BG-11, BBM, and GWT-SE media and grown at 100–250 μE m^−2^ s^−1^ under white LED panel lights. Potential microalgal colonies (green or brown colonies) were transferred to fresh solid medium and maintained by restreaking approximately every 3 to 8 weeks. Eukaryotic microalgal isolates were axenized by successive streaking on solid medium adjusted to 100 μg mL^−1^ to 1 mg mL^−1^ sodium ampicillin (Gold Biotechnology) and 1 μg mL^−1^ to 5 μg mL^−1^ carbendazim (Aldrich).

Diatom strains were isolated using the same technique with solid media containing 5 g L^−1^ silicon dioxide (Sigma-Aldrich). Filamentous microalgae were isolated by sample filtration. A small amount of each biofilm sample (50–450 μL or a 2–3 mm piece) was transferred to a sterile 40–70 μm nylon mesh strainer (Fisher Scientific) over a vacuumed flask. The biofilm was rinsed with approximately 100 mL sterile 1X GWT-SE medium dispensed from a vented wash bottle. Strainers were immersed in a sterile dish containing liquid 1X GWT-SE medium, and 50 μL of biomass remaining on the strainer was spread on solid BG-11 medium and grown at 50–145 μE m^−2^ s^−1^ under white LED panel lights. Filamentous green or brown colonies were transferred to fresh solid medium and maintained as described above.

### 2.2. Morphological and phylogenetic characterization of microalgal isolates

All microalgal isolates were imaged and cataloged by cell morphology via brightfield microscopy as described in Section 2.1. One isolate per unique morphology (including cell size and shape, intracellular vesicles, appendages, chlorophyll distribution, etc.) from each RAB sample was maintained for phylogenetic identification, polyP screening, and cryopreservation.

Microalgae were phylogenetically assigned via 16S/18S rDNA PCR and Sanger sequencing. Colonies of microalgae were transferred from solid medium maintenance cultures to 0.2 mL PCR tubes with 50 μL of sterile lysis buffer containing 20 mM Tris (Biorad; pH 8.0) and 0.1% (v/v) Triton X-100 (Sigma-Aldrich). Tubes were vortex mixed briefly and lysed in a SimpliAmp thermal cycler (Life Technologies) at 60°C for 6 min and 80°C for 4 min. Cell-free lysates were also prepared as PCR and sequencing controls. Lysates were stored at −20°C until PCR amplification.

Lysates were thawed and cell debris was pelleted by centrifugation for 1 min on a LabMini 6 M Mini Centrifuge (Southwest Science). 1 μL of lysate supernatant was transferred to a 0.2 mL PCR tube containing the following reaction mixture: 12.5 μL Q5 Hot Start High Fidelity 2X Master Mix (New England Biolabs), 1.25 μL each of 10 μM forward and reverse primers (Supplementary Table S3; IDT) dissolved in 10 mM Tris (pH 8.0), and 9 μL nuclease-free water (Cytiva Life Sciences). All reactions were cycled on a ProFlex thermal cycler (Life Technologies) as follows: 98°C denaturation for 30 s; 30 cycles of 98°C denaturation for 10 s, annealing at varying temperatures for 30 s, and 72°C elongation for varying times (Supplementary Table S3); 72°C elongation for 2 min; 4°C hold. The annealing temperature for primers D512for/D978rev was determined experimentally for use with both diatom and green alga isolates. Annealing temperatures for all other primer sets were determined with the New England Biolabs Tm Calculator (v1.13.1). PCR amplicons were stored at 4°C and assessed for purity and length by gel electrophoresis in a 1% w/v agarose gel stained with SYBR Safe (Invitrogen). Gels were imaged on a Fluorchem Q imager with Fluorchem Q software (Cell Biosciences).

Primer extension sequencing was performed by Azenta Life Sciences (South Plainfield, New Jersey) with Applied Biosystems BigDye v3.1. The sequencing reactions were carried out on an Applied Biosystem 3730xl DNA Analyzer. Sequencing was performed unidirectionally for most isolates (Supplementary Table S3, Supplementary file S2). Several green algae isolates were sequenced bidirectionally with the mod-ss5/mod-ss3 primer set and unidirectionally with D512for to capture the full 18S rDNA gene. To obtain full 18S rDNA sequences, reverse complement sequences were obtained with the Sequence Manipulation Suite (Stothard, 2000) and the resulting unidirectional sequences were merged with EMBOSS (Rice et al., 2000). All 16S/18S rDNA sequences were deposited to GenBank under accession numbers OP143966 to OP144056 (eukaryotes) and OP142377 to OP142385 (prokaryotes). A top phylogenetic match was determined for each microalgal isolate based on homology with available 16S/18S rDNA sequences in GenBank using NCBI BLASTn (Altschul et al., 1990). The tentative phylogenetic assignment for each strain was supported by microscopy.

### 2.3. Phosphorus content screening of isolated microalgae

To screen the polyP content of isolated microalgae, isolates were cultivated in suspended flask cultures, and polyP was extracted and measured using a fluorometric assay. Isolates were cultivated by transferring microalga colonies to 35 mL liquid medium in 125 mL non-baffled borosilicate Erlenmeyer flasks with aluminum foil caps. BBM amended with different nutrients was used for cultivating the various microalgae isolates (Supplementary Table S3, Supplementary file S2). Each strain was cultivated for polyP measurement as a single biological replicate. Unicellular microalgae were grown on an Orbital Genie SI-1700 shaker at 100 rpm illuminated at 50–85 μE m^−2^ s^−1^ by white light LED panels. Filamentous microalgae were cultivated under the same conditions without shaking. PolyP content has been shown to fluctuate, often peaking early in the growth curve (Sanz-Luque et al., 2020). As such, cultures were grown to a low cell density to capture the upper range of polyP accumulation, with unicellular cyanobacteria and green algae harvested at a low optical density at 730 nm wavelength (OD_730_) as measured with a Biochrom WPA Biowave II (Supplementary file S2). Unicellular cyanobacteria and green algae cultures were measured for OD_730_ every 1–2 days and those exceeding a previously determined minimum detection threshold of the fluorometric polyP assay of OD_730_ 0.5 (data not shown) were harvested. Diatoms and filamentous microalgae, for which even subsampling and accurate OD_730_ measurements were not possible due to cell aggregation in liquid culture, were grown to a visually determined low cell density over 1–3 weeks (Supplementary file S2).

Once grown, screening cultures were harvested for polyP and CDW measurements. Cultures were placed on ice, and cells were harvested for polyP measurements by transferring 1 mL culture to a microcentrifuge tube and adjusting to a final concentration of 0.001% (v/v) Triton X-100 to improve harvesting yield. For non-homogenous cultures, the entire culture was adjusted to 0.001% (v/v) Triton X-100 and homogenized by pipetting and vortex mixing the culture before transferring the culture to microcentrifuge tubes. Tubes were centrifuged at 16,000 × g for 5 min and the supernatant was removed. Cell pellets were washed in ultrapure water containing the same concentration of Triton X-100 and recentrifuged as before. The supernatant was removed, and cell pellets were stored at −80°C until polyP measurement. A RAB biofilm (microalgae consortium) from a pilot-scale RAB system treating municipal secondary effluent in Slater, Iowa was also diluted 1:100 in ultrapure water and harvested as described above for polyP measurement.

CDWs were determined by filtering two 10 mL aliquots of each culture through pre-rinsed, dried, and weighed 0.7 μm hydrophilic glass fiber filters (Millipore Sigma) over a vacuumed Buchner funnel. Filters were dried for 48 h at 60°C and reweighed. The CDW value of the microalgae consortium was determined by centrifuging triplicate 20 mL biofilm samples at 5,000 × g for 15 min, removing the supernatant, freeze-drying the biofilm in a Harvest Right HRFD-PMED-WH freeze-dryer, and weighing the dried biomass.

Phosphorus accumulation by each isolate and the microalgae consortium was quantified by extracting and measuring polyP. To extract polyP, 300 μg (CDW) of harvested cell material was suspended in 600 μL of 10 mM HEPES buffer (pH 6.8) and transferred to 2 mL screwcap tubes containing 600 μL of 0.1 mm silica/zirconia beads (BioSpec). Samples were vortex mixed and boiled on a dry heat block for 5 min at 100° C. Samples were then cooled on ice, and cells were lysed in an Eppendorf Tissue Lyzer II for 5 min at 30 Hz. Cell debris and beads were removed by centrifuging twice at 16,000 × g for 3 min, transferring lysate supernatant to fresh tubes each time. The recovered lysate containing the extracted polyP was treated with enzymes to remove biomolecules known to interfere with fluorometric polyP quantification (Martin and Van Mooy, 2013). 300 μL of supernatant was treated consecutively at 37°C with 5 μL Ambion RNase Cocktail for 10 min, 5 μL Invitrogen TURBO Dnase for 10 min, and 10 μL Roche Proteinase K for 30 min. Blank solutions were prepared with the same protocol but without cells. PolyP concentration in the enzyme-treated lysate was quantified using the ProFoldin MicroMolar PolyP Assay Kit (Cat. No. PPD1000) according to the manufacturer’s protocol. Fluorescence measurements were taken in Andwin Scientific 96-well clear-bottom black microtiter plates (Cat. No. 655096) with a Tecan Infinite M200 Pro plate reader.

### 2.4. Cryopreservation and culture collection submission of isolated microalgae

Microalgae isolates were cryopreserved with protocols adapted from Elliott et al. (2012) and the Culture Collection of Algae at the University of Texas at Austin (UTEX). Isolates were cryopreserved as either liquid cultures or agar slant cultures. Briefly, liquid-cultured isolates were cultured in 20 mL BBM in 50 mL non-baffled borosilicate Erlenmeyer flasks with aluminum foil caps. Upon reaching OD_730_ 0.5–1.2, 1.9 mL aliquots of the cultures were transferred to 2 mL cryotubes (Corning), adjusted to 5% (v/v) DMSO (Sigma-Aldrich), gently mixed, and cooled to −80°C in Mr. Frosty (Nalgene) freezing containers before being transferred to a liquid nitrogen cryopreservation tank (−196°C) for long-term storage. Slant-cultured isolates were cultured as streaks on 1-mL BBM agar slants (Supplementary Table S1) prepared in 2 mL cryotubes. Slant cultures were gently overlaid with 800 μL of liquid BBM containing 5% (v/v) DMSO and cryopreserved as described above. Diatoms were cryopreserved as described above, but with a DMSO concentration of 12% (v/v).

In addition to cryopreservation, a selection of P-hyperaccumulating and diverse microalgae isolated in this study were submitted to UTEX^1^ and are publicly available. Submitted strains can be searched on the UTEX website using the identifiers assigned in this study (e.g., TCF-8d) or the keywords “wastewater remediation” or “polyphosphate accumulation.”

### 2.5. Bench-scale RAB colonization and phosphorus removal testing

Four bench-scale RAB systems, each with a 4 L reservoir and a cotton belt (Gross et al., 2013) with 0.1 m^2^ surface area were used to assess strain performance in a RAB system in comparison to a naturally occurring consortium. The experiment was conducted in a non-sterile greenhouse in Boone, Iowa in March 2022 at 20°C and supplemented with constant artificial illumination by custom-made white LED panel lights (Reliance Laboratories) at 200 μE m^−2^ s^−1^. *Chlamydomonas pulvinata* TCF-48 g and *Chlorellaceae* sp. TCF-17 g were selected for testing due to their high P content and fast growth, respectively. Strains possessing higher levels of polyP (i.e., diatoms) were not included, as they had not yet been screened for polyP at the time of RAB testing. The same RAB microalgae consortium included as a community baseline in the polyP screen was also included as a community baseline in this test.

To prepare seed cultures, 100 mL of a low-density BBM culture of each strain or a 1:100 dilution of the RAB microalgae consortium into ultrapure water was inoculated into triplicate Erlenmeyer flasks containing 900 mL BBM. Seed cultures were grown for 4–7 days in a Sanyo MCO-17AI CO_2_ Incubator on a Velp Scientifica magnetic multi-stirrer at 100 rpm, 32°C, and 2% CO_2_. Each of the four bench-scale RAB system reservoirs was filled with 2 L of seed culture (*Chlamydomonas pulvinata* TCF-48 g, *Chlorellaceae* sp. TCF-17 g, a 1:1 mixture of both strain seed cultures, or the natural consortium) and 2 L of fresh BBM. BBM was chosen for this preliminary test in consistency with the conditions used for polyphosphate screening.

The RAB systems were operated at a constant speed of 6 rpm with a velocity of 6 cm s^−1^ for 3 weeks with a semi-continuous flow of BBM. Approximately 500 mL of ultrapure water was added to each RAB reservoir daily to accommodate for evaporation and maintain a total solution volume of 4 L. Every 7 days, the biomass on the belt was observed with an Olympus CX31 microscope to verify belt colonization by the respective strain(s) and harvested by scraping into 50 mL conical tubes. Harvested biomass was centrifuged at 5,000 x g for 15 min, the supernatant was removed, and the biomass was freeze-dried as in Section 2.3. Freeze-dried biomass samples were stored at −20°C.

After the first belt harvest at 7 days, 2 L of RAB reservoir solution was drained from each RAB system and replenished with 1 L of fresh seed culture and 1 L of fresh BBM. After the second harvest at 14 days, 2 L of solution was drained from each RAB reservoir and replenished with 2 L of fresh BBM. Total P fed into each RAB system throughout the experiment (as fresh media or seed culture) was 424 mg P in 8 L, calculated using the media recipe (Nichols and Bold, 1965). The freeze-dried harvested biomass samples were weighed to determine biomass productivity and sent to Midwest Laboratories, Inc. (Omaha, Nebraska) for total P content analysis using inductively coupled plasma mass spectrometry (ICP-MS). Biomass productivity measurements were collected for each of the three harvests from each RAB belt. ICP-MS P measurements were collected for biomass from the first harvest and for pooled biomass samples from both the second and third harvest from each RAB belt.

## 3. Results

### 3.1. Biodiversity in the wastewater treatment biofilms

**Figure 2:**
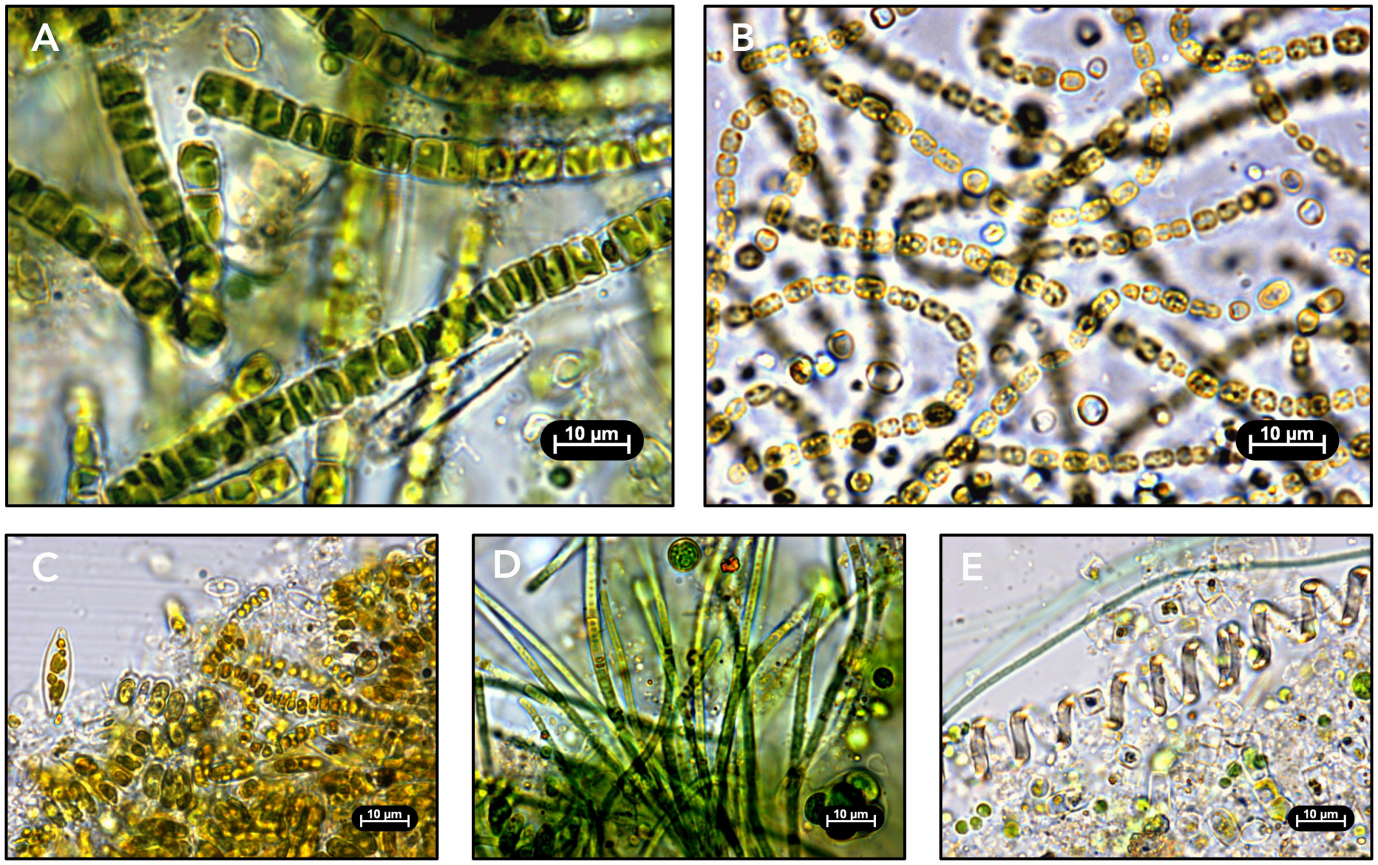
Light microscopy images of RAB biofilm samples. **(A)** Biofilm sampled in April 2021 from a demonstration-scale RAB treating municipal final clarifier effluent in Creston, Iowa. **(B)** Biofilm sampled in December 2020 from a pilot-scale RAB treating industrial (meat processing) anaerobic digester effluent in Sioux City, Iowa. **(C)** Biofilm sampled in April 2021 from a demonstration-scale RAB treating municipal tertiary effluent in Chicago, Illinois. **(D)** Biofilm sampled in December 2020 from a demonstration-scale RAB treating municipal secondary effluent in Slater, Iowa. **(E)** Biofilm sampled in February 2021 from a pilot-scale RAB treating municipal intermediate clarifier effluent in Creston, Iowa.

RAB samples were imaged upon arrival and possessed a large diversity of microalgae, including cyanobacteria, diatoms, and green algae (Figure 2). RAB systems notably also supported diverse organisms of higher trophic levels, including bacteria; filamentous fungi; protozoa including amoebae, euglenids, and ciliates; and animals including nematodes, rotifers, and tardigrades. RAB community compositions varied drastically across RAB system locations and sampling time points. This suggests that RAB microbial communities are dynamic and may change with fluctuations such as seasonal shifts in light and temperature or influent composition.

### 3.2. Strain isolation and morphological characterization

**Figure 3:**
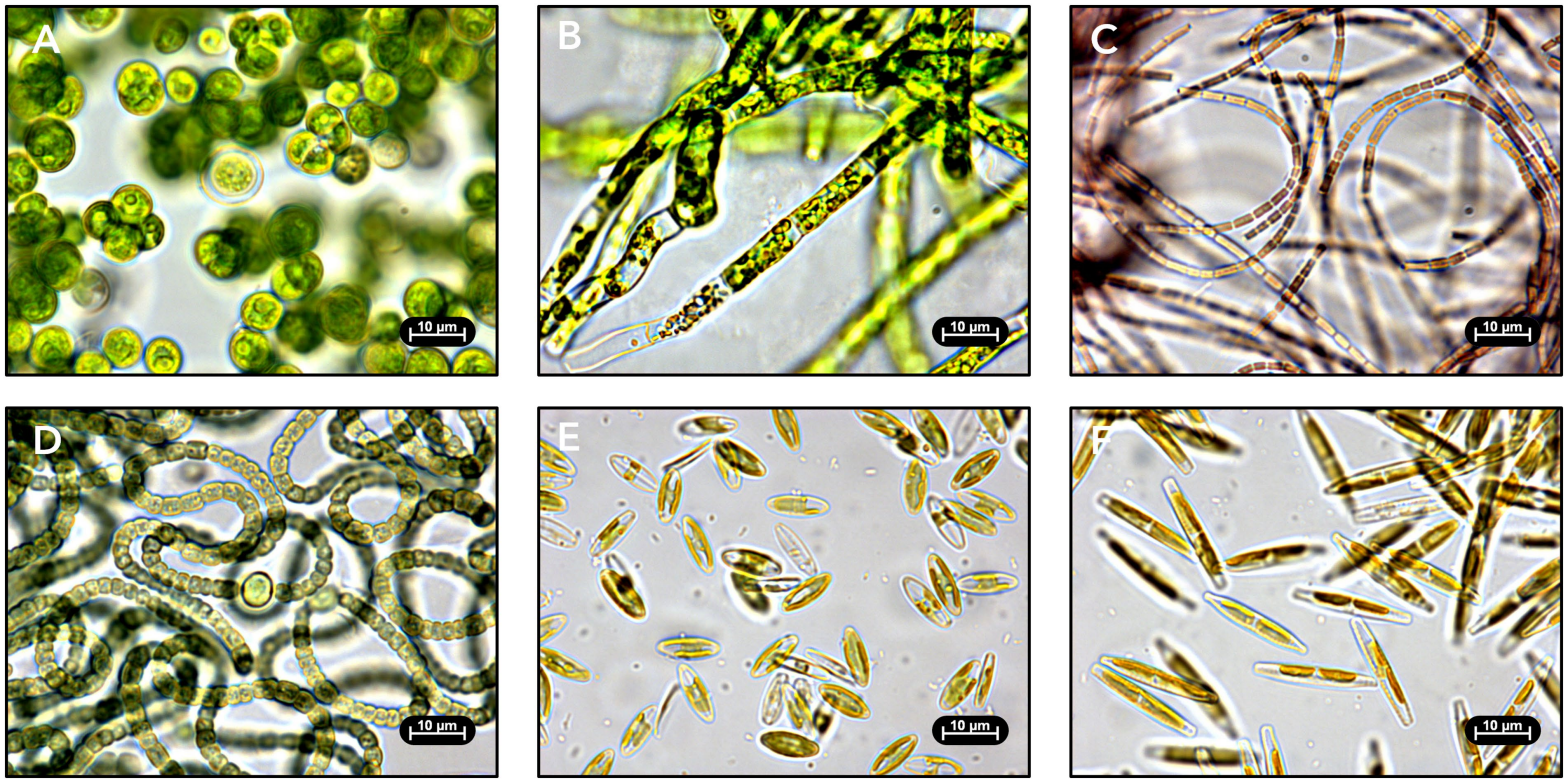
Light microscopy images of isolated RAB microalgae. Strains were identified by 16S/18S ribosomal DNA sequencing. **(A)** Green alga *Desmodesmus* sp. TCF-3 g isolated from Chicago, Illinois. **(B)** Green alga *Caespitella pascheri* TCF-75 g isolated from Creston, Iowa. **(C)** Cyanobacterium *Pseudanabaena* sp. TCF-9c isolated from Creston, Iowa. **(D)** Cyanobacterium *Nostoc edaphicum* TCF-1c isolated from Chicago, Illinois. **(E)** Diatom *Craticula molestiformis* TCF-1d isolated from Creston, Iowa. **(F)** Diatom *Nitzschia palea* TCF-4d isolated from Creston, Iowa.

Approximately 770 microalgal colonies were obtained in total from the samples collected from eight active RAB systems across three time points. Microscopic imaging of each colony revealed a diverse range of green algae (Figures 3A,B), cyanobacteria (Figures 3C,D), and diatoms (Figures 3E,F) among the isolates. Microscopy images were used to identify morphologically unique isolates for retention in the culture collection. One isolate per distinct morphology from each sample was considered unique and retained in the culture collection (i.e., multiple isolates of the same morphology were retained if they originated from different RAB samples). A total of 101 strains were retained in the culture collection. Targeted isolation techniques, including silicon enrichment and RAB sample filtration, were successful and resulted in the isolation of nine diatoms and ten filamentous microalgae, respectively.

### 3.3. Genetic identification of isolated microalgae

Microalgae retained in the culture collection were identified by 16S/18S rDNA sequencing and microscopy. A representative microscopy image of each strain, as well as the RAB microalgae consortium used in this study, is available in Supplementary file S1. It should be noted that many isolated strains display varying morphology between cells attributable to the species’ life cycle. High-quality rDNA sequences were obtained for 100 of the 101 isolated strains (Supplementary file S2). Of these sequenced strains, 97 strains were assigned tentative phylogenetic matches at the genus or species level.

Of the 101 microalgae isolated, 82 were green algae, with the vast majority assigned to the *Scenedesmaceae* (51) and *Chlorellaceae* (21) families. Green algae strains assigned as *Chlamydomonas pulvinata*, *Caespitella pascheri*, and *Chlorolobion* spp. were also isolated. Additionally, nine diatom strains were isolated and assigned as *Craticula molestiformis*, *Eolimna minima*, *Nitzschia palea*, and *Nitzschia* sp. Ten cyanobacteria strains were isolated and assigned as *Synechocystis* sp., *Leptolyngbya* spp., *Nodosilinea* spp., *Nostoc edaphicum*, and *Pseudanabaena* sp.

### 3.4. Polyphosphate content screening of isolated microalgae

**Figure 4:**
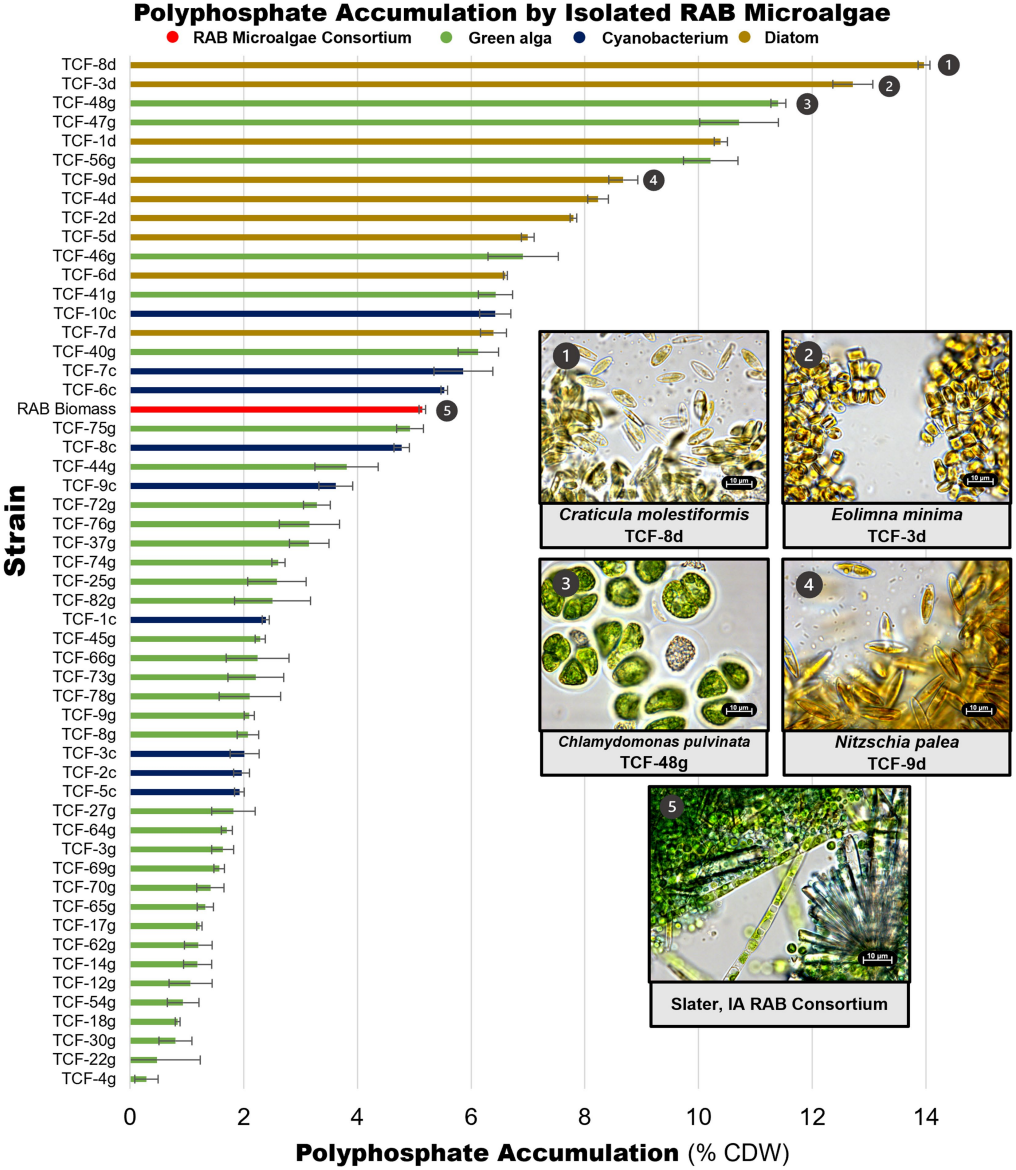
Polyphosphate content of isolated RAB microalgae. Unique microalgae were screened for polyphosphate content, along with a RAB microalgae consortium from a RAB system treating municipal wastewater in Slater, Iowa. Data depicts measured polyphosphate as a percentage of cell dry weight (CDW). Light microscopy images of the top P-hyperaccumulating morphologically unique strains and the RAB microalgae consortium are shown. Thirty-eight green algae with polyphosphate content lower than the microalgae consortium were excluded for visual clarity and are available in Supplementary file S2. Error bars reflect the standard deviation of triplicate fluorescence measurements of extracted polyphosphate from a single strain culture or consortium sample.

Microalgae isolates were screened for P accumulation by extracting and measuring polyP. PolyP measurements were obtained for 91 of the 101 strains retained in the culture collection, including all strains that could be successfully cultivated in liquid BBM medium (Figure 4, Supplementary file S2). A RAB microalgae consortium sampled from a RAB system in Slater, Iowa was also screened to serve as a baseline representing P accumulation in existing RAB systems. The RAB microalgae consortium accumulated 5.1% polyP by CDW. In comparison, seven isolates accumulated at least 50% more polyP, including three isolates of *Chlamydomonas pulvinata*, two isolates of *Craticula molestiformis*, and one isolate each of *Eolimna minima* and *Nitzschia palea*. The top P-hyperaccumulating strain, *Craticula molestiformis* TCF-8d, accumulated 14.0% polyP by CDW.

Notably, all green algae were found to be below the RAB microalgae baseline, except for multiple isolates of *Chlamydomonas pulvinata*. All nine diatom strains and three filamentous cyanobacteria strains accumulated more polyP than the RAB microalgae consortium.

### 3.5. Recolonization and performance of *Chlamydomonas pulvinata* TCF-48 g in bench-scale RAB systems

As an initial test to show that isolated P-hyperaccumulating microalgae can recolonize the RAB system and improve P removal, one P-hyperaccumulating green alga strain, *Chlamydomonas pulvinata* TCF-48 g (Figure 4, panel 3), was tested for recolonization, biomass productivity, and total P accumulation in a bench-scale RAB system containing BBM medium. At the time of testing, *C. pulvinata* TCF-48 g was the highest P-accumulating strain identified. A RAB microalgae consortium from a Slater, Iowa RAB system (Figure 4, panel 5) and a fast-growing, low P-accumulating isolate *Chlorellaceae* TCF-17 g were also tested in parallel for comparison.

**Figure 5:**
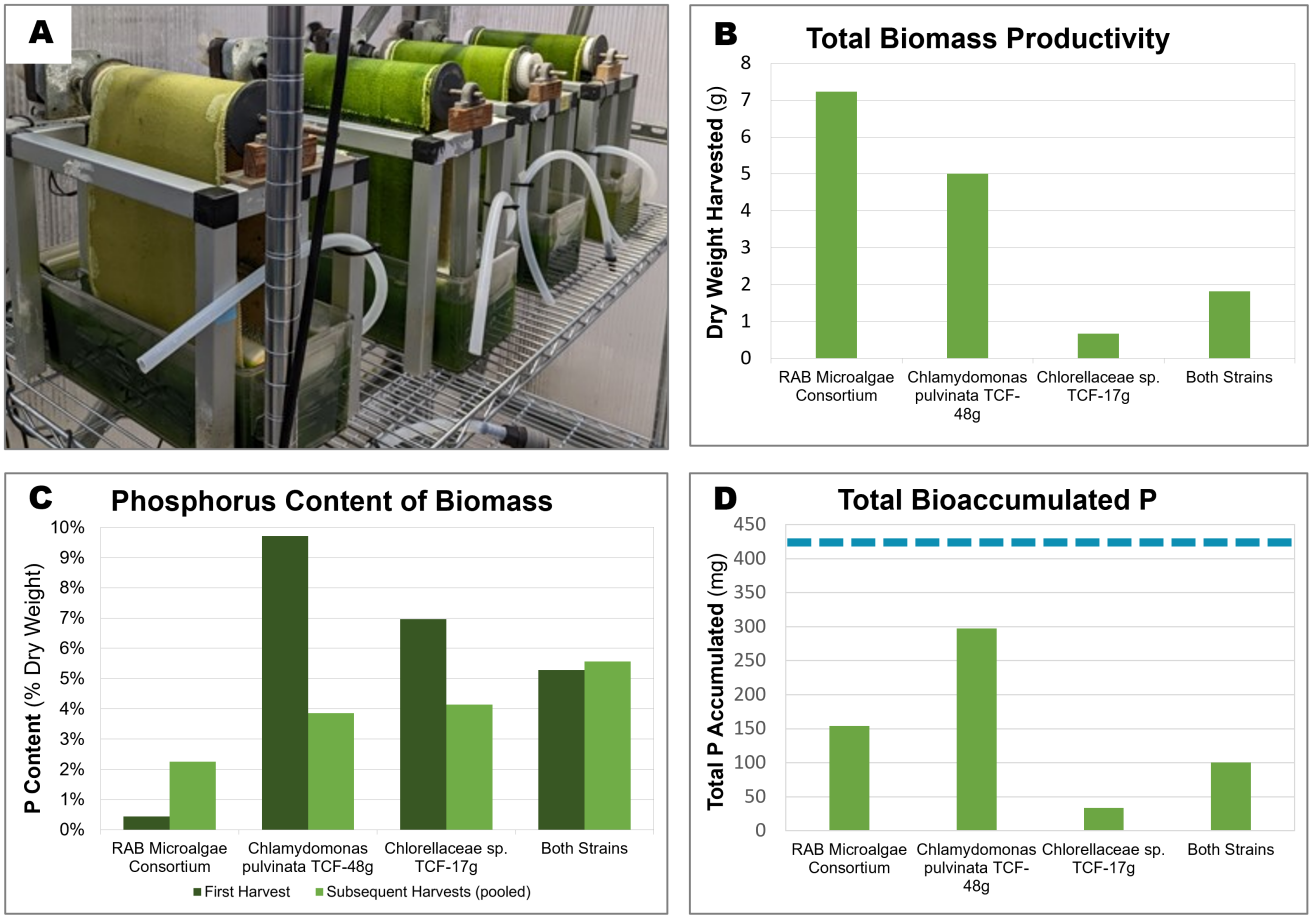
Biomass productivity, phosphorus content, and accumulated P of microalgae grown in bench-scale RAB systems containing BBM medium. Two isolated microalgae were cultivated over 3 weeks in bench-scale RAB systems alongside a RAB microalgae consortium from a RAB system treating municipal wastewater in Slater, Iowa. **(A)** Four bench-scale RAB systems used to cultivate (from left to right) *Chlorellaceae* sp. TCF-17 g, *Chlamydomonas pulvinata* TCF-48 g, both strains together, and the RAB microalgae consortium **(B)** Total dry biomass productivity of the four RAB systems over the course of 3 weeks. **(C)** Total phosphorus content of biomass harvested from each RAB system across the first harvest (7 days) and subsequent pooled harvests (14 and 21 days). **(D)** Total accumulated phosphorus by each RAB system over 3 weeks of growth (calculated from biomass productivity and P content). The dashed blue line represents the total amount of phosphorus added to each system throughout the experiment (424 mg).

All four inocula successfully colonized the RAB belts (Figure 5A, Supplementary Figure S1). However, stark differences in productivity were observed between the inocula, with the RAB microalgae consortium producing the greatest amount (7.2 g) of total dry biomass throughout the experiment (Figure 5B). The P-hyperaccumulating strain *C. pulvinata* TCF-48 g was 31% less productive, producing 5 g of dried biomass.

Of the harvested biomass from the RAB systems, the RAB microalgae consortium possessed the lowest P content, ranging between 0.43% CDW total P at the initial harvest and increasing to 2.25% P in subsequent harvests (Figure 5C). The isolated P-hyperaccumulating *C. pulvinata* TCF-48 g possessed approximately 22-fold more P than the RAB microalgae consortium in the initial harvest, containing 9.7% P. However, this value decreased to 3.9% P in subsequent harvests.

Taking together the productivity and P content data, the harvested biomass from *C. pulvinata* TCF-48 g contained 297 mg P out of the 424 mg P added to each RAB reservoir throughout the experiment. This equates to a P removal rate of 70% under the conditions tested, which is two-fold greater than that of the RAB microalgae consortium, which removed 36.4% P (Figure 5D). It should be noted that any additional bioaccumulated P in microalgae biomass remaining on the belt after scraping or in the reservoir was not measured.

## 4. Discussion

### 4.1. Diversity of algal strains isolated from RAB biofilm

RAB systems are an emerging algal technology for wastewater treatment (Gross et al., 2013), but to date, the microalgal composition has not been characterized. We found that the microbial community composition of RAB systems varies drastically by system location, influent composition, and season of sampling. This work isolated 101 microalgal strains from these RAB system microbial communities. Further, the targeted isolation of diverse microalgae from these samples was successful. Diatoms were isolated by supplementing media with silicon dioxide, and filamentous microalgae were isolated by filtering the RAB samples before plating. While these techniques proved useful in isolating microalgae based on morphology and nutritional requirements, other media compositions and isolation techniques may further improve the diversity of strains isolated for future microalgal bioprospecting efforts.

### 4.2. Diversity and dynamics of algal polyphosphate accumulation

This work identified significant variation in polyP accumulation among microalgal strains, ranging from 0 to 14% CDW. This equates to 0–5.4% P attributable to polyP (with elemental P accounting for approximately 40% of polyP by weight). It should be noted that cells typically possess an additional 1% elemental P attributable to other phosphorous biomolecules (e.g., nucleic acids, phospholipids) not measured here (Grobbelaar, 2013). As a reference, the RAB microalgae consortium possessed 5.1% CDW polyP, or approximately 3% total P including other P biomolecules, in the polyP screen and between 0.43% and 2.25% CDW total P in the bench-scale RAB testing. This is consistent with previous research demonstrating that naturally occurring microalgae in waste stabilization ponds possess between 0.21 and 3.85% P by CDW (Powell, 2009).

Notably, our results suggest that P accumulation varies across different phylogenetic groups. Diatom strains generally accumulated higher levels of polyP than most green algae strains (Figure 4). This is a significant finding considering previous microalgal bioprospecting work for resource recovery has focused heavily on green algae (Massimi and Kirkwood, 2016; Sweiss, 2017; Lavrinovičs et al., 2020). Diatoms are known to accumulate high levels of both phosphorus and nitrogen as a means to survive under unfavorable conditions (Lomas and Glibert, 2000; Kamp et al., 2011; Coppens et al., 2014). Dyhrman et al. (2012) discovered that even under low P, a condition known to inhibit polyP synthesis in most microalgae, the diatom *Thalassiosira pseudonana* increased polyP synthesis. Further research into P metabolism in diatoms is needed to fully understand and exploit these unusual polyP dynamics (Lovio-Fragoso et al., 2021). The screening data presented in this study indicate that diatoms may possess an enormous amount of unexplored potential as tools to achieve a circular nutrient economy. These data also demonstrate that diverse strain isolation in bioprospecting work is key to identifying valuable strains for resource recovery from wastewater.

In this work, the polyP content of most strains was screened within a single culture condition and at a single time point within their growth. However, it has been reported that polyP accumulation can fluctuate in microalgae depending on nutrient availability (Powell et al., 2009; Voronkov and Sinetova, 2019) and growth phase (Ota et al., 2016). For example, Lavrinovičs et al. (2020) found that two out of three tested green algae strains accumulated high levels of polyP after 2 days of growth in municipal wastewater, but the polyP content decreased rapidly thereafter. Similarly, Voronkov and Sinetova (2019) observed that the cyanobacterium *Synechocystis* sp. PCC 6803 accumulated polyP within minutes of surplus phosphate exposure, but that polyP levels decreased rapidly and depleted within 2 days. Thus, the single time point used for strain screening may have missed the peak P accumulation capacity in some strains, precluding the discovery of other algal strains capable of P hyperaccumulation. Measuring polyP under varying conditions and at multiple time points during strain growth could elucidate the full capacity and dynamics of P accumulation by the microalgae strains isolated in this study.

### 4.3. Application of the algal strain collection

The P-hyperaccumulating microalgae isolated in this study have the potential to boost the P removal of RAB systems. This work included an initial test of recolonization and strain performance of two algal strains in bench-scale RAB systems containing BBM medium. The tested strain *C. pulvinata* TCF-48 g was able to recolonize the RAB biofilm and outperform a RAB microalgae consortium in P removal. Further, the P-rich biomass produced from this strain possessed up to 9.7% P, higher than the 5–7% P typically found in biosolids produced by enhanced biological phosphorus recovery (Yuan et al., 2012), a competing biological P-removal technology. This observation is consistent with the high polyP content of this strain grown in a flask during the polyP content screen (Figure 4). However, the high level of total P measured in the bench-scale RAB testing was observed only during the first harvest of algae biomass from the RAB system and decreased to 3.9% P in subsequent harvests. The reduction in the P content of this strain in subsequent harvests may have been due to the exhaustion of available phosphate in the medium. 70% of the P added to the RAB system was recovered in *C. pulvinata* TCF-48 g biomass scraped from the belt (Figure 5D). However, residual algae biomass remaining on the RAB belt after scraping and in the RAB reservoir also bioaccumulated P that was not measured. Further, precipitation of phosphate may have occurred in the system, though this likely would have been negligible due to the high proportion of P recovered in algae biomass. Therefore, it is conceivable that the available P in the medium had been depleted at the time of the second and third harvests. The P accumulation observed in this strain during the initial harvest may thus represent the true upper limit of P accumulation by this strain in conditions where phosphate is not limited, such as in the conditions of commercial-scale RAB systems, in which wastewater is supplied continuously. This possibility will be tested in future studies.

While these preliminary test results are promising, whether other P-hyperaccumulating microalgae isolated in this study can easily recolonize RAB systems and similarly outperform RAB microalgae consortia remains to be studied. Furthermore, the performance of isolated P-hyperaccumulating microalgae in RAB systems treating wastewater (rather than synthetic medium) remains to be tested. P-hyperaccumulating strains are likely to face much higher levels of ecological competition in systems fed wastewater. The inoculation of polycultures of multiple P-hyperaccumulating strains with varying ecological niches may be a useful approach to improve inoculum colonization and resilience in RAB systems.

In addition to recovering P, the P-hyperaccumulating strains isolated in this study may be valuable for other waste remediation applications, such as metal removal and recovery from industrial (e.g., mining) and municipal wastewater. PolyP is a highly negatively charged polymer known to chelate and accumulate cationic metals in microalgae (Suresh Kumar et al., 2015). Uranium (Acharya et al., 2012), lead (Maldonado et al., 2010), copper (Ballan-Dufrançais et al., 1991; Adams et al., 2016), silver (Ballan-Dufrançais et al., 1991), cadmium (Nishikawa et al., 2003), and other metals have been shown to concentrate in microalgal polyP bodies. As such, P-hyperaccumulating microalgae may be useful in both recovering valuable metals and removing harmful metals from wastewater streams. RAB systems have previously been found to remove metals from wastewater, including chromium, manganese, copper, and other metals from municipal sludge thickening supernatant (Zhao et al., 2018) and high levels of nickel from synthetic media (Zhou et al., 2021), although the relationship between biofilm polyP levels and metal accumulation has not been studied. RAB systems and other algal waste remediation technologies may benefit from the use of P-hyperaccumulating microalgae with greater capacity to take up and store metals from wastewater.

While these strains were isolated to improve P removal by RAB systems, the P-hyperaccumulating microalgae isolated in this study could potentially be used to improve P removal in other microalgal wastewater treatment systems (e.g., waste stabilization ponds or photobioreactors). Additionally, these microalgae isolates may also possess other valuable phenotypes for downstream applications of wastewater-grown algal biomass, such as high lipid content for biofuel production (Elliott et al., 2012; Massimi and Kirkwood, 2016). Future screening efforts may thus identify other valuable microalgae strains isolated in this study.

Overall, this work demonstrates the potential of bioprospecting as an effective approach to improve resource recovery from wastewater by microalgae. Of the 101 microalgae strains isolated from RAB systems in this study, multiple species of diatoms and one species of green alga were found to accumulate high levels of polyP. These isolates may serve as valuable tools to remove and recover P and other resources from wastewater.

## Data availability statement

The genetic data presented in this study can be found in online repositories. The names of the repository/repositories and accession number(s) can be found at: https://www.ncbi.nlm.nih.gov/, accession numbers OP143966 to OP144056 (eukaryotes) and OP142377 to OP142385 (prokaryotes).

## Author contributions

JY, MaG, KK, and JG designed the study. ES, MC, DG, JS, and KC developed methods. ES, MC, CU, DG, XZ, JD, and MiG collected and analyzed data. ES, DG, and JY drafted the manuscript. All authors contributed to the article and approved the submitted version.

## Funding

This work was authored in part by the National Renewable Energy Laboratory, operated by Alliance for Sustainable Energy, LLC, for the U.S. Department of Energy (DOE) under Contract No. DE-AC36-08GO28308. Funding provided by the U.S. Department of Energy, BioEnergy Technologies Office and Technology Commercialization Office; and in part by Office of Science, Office of Biological and Environmental Research, Genomic Science Program under Secure Biosystems Design Science Focus Area (SFA), IMAGINE BioSecurity: Integrative Modeling and Genome-scale Engineering for Biosystems Security. The views expressed in the article do not necessarily represent the views of the DOE or the U.S. Government. The U.S. Government retains and the publisher, by accepting the article for publication, acknowledges that the U.S. Government retains a nonexclusive, paid-up, irrevocable, worldwide license to publish or reproduce the published form of this work, or allow others to do so, for U.S. Government purposes.

## Conflict of interest

XZ, DG, JD, MiG, and MaG are current or former employees of Gross-Wen Technologies.

The remaining authors declare that the research was conducted in the absence of any commercial or financial relationships that could be construed as a potential conflict of interest.

## Correction note

This article has been corrected with minor changes. These changes do not impact the scientific content of the article.

## Publisher’s note

All claims expressed in this article are solely those of the authors and do not necessarily represent those of their affiliated organizations, or those of the publisher, the editors and the reviewers. Any product that may be evaluated in this article, or claim that may be made by its manufacturer, is not guaranteed or endorsed by the publisher.

## Footnotes

^1^ https://utex.org/

## Abbreviations

RAB: Revolving algal biofilm
P: Phosphorus
PolyP: Polyphosphate
CDW: Cell dry weight
rDNA: Ribosomal deoxyribonucleic acid
MWRD Chicago: Metropolitan Water Reclamation District of Greater Chicago
EBPR: Enhanced biological phosphorus removal
OD_730_: Optical density at 730 nm wavelength
BBM: Bold Basal Medium
UTEX: Culture Collection of Algae at the University of Texas at Austin
ICP-MS: Inductively coupled plasma mass spectrometry
*C. pulvinata*: *Chlamydomonas pulvinata*

## References

[ref1] Acharya C. Chandwadkar P. Apte S. K. (2012). Interaction of uranium with a filamentous, heterocystous, nitrogen-fixing cyanobacterium, *Anabaena torulosa*. Bioresour. Technol. 116, 290–294. doi: 10.1016/j.biortech.2012.03.068 22522016

[ref2] Adams M. S. Dillon C. T. Vogt S. Lai B. Stauber J. Jolley D. F. (2016). Copper uptake, intracellular localization, and speciation in marine microalgae measured by synchrotron radiation X-ray fluorescence and absorption microspectroscopy. Environ. Sci. Technol. 50, 8827–8839. doi: 10.1021/acs.est.6b00861 27437565

[ref3] Altschul S. F. Gish W. Miller W. Myers E. W. Lipman D. J. (1990). Basic local alignment search tool. J. Mol. Biol. 215, 403–410. doi: 10.1016/S0022-2836(05)80360-2 2231712

[ref4] Ballan-Dufrançais C. Marcaillou C. Amiard-Triquet C. (1991). Response of the phytoplanctonic alga Tetraselmis suecica to copper and silver exposure: vesicular metal bioaccumulation and lack of starch bodies. Biol. Cell. 72, 103–112. doi: 10.1016/0248-4900(91)90084-z

[ref5] Bunce J. T. Ndam E. Ofiteru I. D. Moore A. Graham D. W. (2018). A review of phosphorus removal technologies and their applicability to small-scale domestic wastewater treatment systems. Front. Environ. Sci. 6:8. doi: 10.3389/fenvs.2018.00008

[ref6] Coppens J. Decostere B. Van Hulle S. Nopens I. Vlaeminck S. E. De Gelder L.. (2014). Kinetic exploration of nitrate-accumulating microalgae for nutrient recovery. Appl. Microbiol. Biotechnol. 98, 8377–8387. doi: 10.1007/s00253-014-5854-9 25001595

[ref7] Cornel P. Schaum C. (2009). Phosphorus recovery from wastewater: needs, technologies and costs. Water Sci. Technol. 59, 1069–1076. doi: 10.2166/wst.2009.045 19342801

[ref8] Croft M. T. Warren M. J. Smith A. G. (2006). Algae need their vitamins. Eukaryot. Cell 5, 1175–1183. doi: 10.1128/EC.00097-06, PMID: 16896203 PMC1539151

[ref9] Dyhrman S. T. Jenkins B. D. Rynearson T. A. Saito M. A. Mercier M. L. Alexander H.. (2012). The transcriptome and proteome of the diatom *Thalassiosira pseudonana* reveal a diverse phosphorus stress response. PLoS One 7:e33768. doi: 10.1371/journal.pone.0033768, PMID: 22479440 PMC3315573

[ref10] Elliott L. G. Feehan C. Laurens L. M. L. Pienkos P. T. Darzins A. Posewitz M. C. (2012). Establishment of a bioenergy-focused microalgal culture collection. Algal Res. 1, 102–113. doi: 10.1016/j.algal.2012.05.002

[ref11] Elser J. Bennett E. (2011). A broken biogeochemical cycle. Nature 478, 29–31. doi: 10.1038/478029a 21979027

[ref12] Fox R. (2022). Accelerating nutrient pollution reductions in the Nation’s waters. Washington, DC: US Environmental Protection Agency.

[ref13] Grobbelaar J. U. (2013). “Inorganic algal nutrition,” in Handbook of Microalgal Culture: Applied Phycology and Biotechnology, 2nd Edn, eds Emeritus A. R. Hu Q. (Hoboken, NJ: Wiley), 123–133.

[ref14] Gross M. Henry W. Michael C. Wen Z. (2013). Development of a rotating algal biofilm growth system for attached microalgae growth with in situ biomass harvest. Bioresour. Technol. 150, 195–201. doi: 10.1016/j.biortech.2013.10.016 24161650

[ref15] Gross M. Mascarenhas V. Wen Z. (2015). Evaluating algal growth performance and water use efficiency of pilot-scale revolving algal biofilm (RAB) culture systems. Biotechnol. Bioeng. 112, 2040–2050. doi: 10.1002/bit.25618 25899246

[ref16] Gross M. Wen Z. (2014). Yearlong evaluation of performance and durability of a pilot-scale revolving algal biofilm (RAB) cultivation system. Bioresour. Technol. 171, 50–58. doi: 10.1016/j.biortech.2014.08.052 25189508

[ref17] Gross M. Zhao X. Mascarenhas V. Wen Z. (2016). Effects of the surface physico-chemical properties and the surface textures on the initial colonization and the attached growth in algal biofilm. Biotechnol. Biofuels 9:38. doi: 10.1186/s13068-016-0451-z, PMID: 26884812 PMC4754892

[ref18] Kamp A. de Beer D. Nitsch J. L. Lavik G. Stief P. (2011). Diatoms respire nitrate to survive dark and anoxic conditions. Proc. Natl. Acad. Sci. 108, 5649–5654. doi: 10.1073/pnas.1015744108, PMID: 21402908 PMC3078364

[ref19] Lavrinovičs A. Mežule L. Juhna T. (2020). Microalgae starvation for enhanced phosphorus uptake from municipal wastewater. Algal Res. 52:102090. doi: 10.1016/j.algal.2020.102090

[ref20] Lavrinovičs A. Murby F. Zīverte E. Mežule L. Juhna T. (2021). Increasing phosphorus uptake efficiency by phosphorus-starved microalgae for municipal wastewater post-treatment. Microorganisms 9:1598. doi: 10.3390/microorganisms9081598, PMID: 34442678 PMC8399584

[ref21] Lindsey A. J. Thoms A. W. Dancer J. Gross M. (2021). Evaluation of algae-based fertilizers produced from revolving algal biofilms on Kentucky bluegrass. Agronomy 11:1288. doi: 10.3390/agronomy11071288

[ref22] Lomas M. W. Glibert P. M. (2000). Comparisons of nitrate uptake, storage, and reduction in marine diatoms and flagellates. J. Phycol. 36, 903–913. doi: 10.1046/j.1529-8817.2000.99029.x

[ref23] Lovio-Fragoso J. P. de Jesús-Campos D. López-Elías J. A. Medina-Juárez L. Á. Fimbres-Olivarría D. Hayano-Kanashiro C. (2021). Biochemical and molecular aspects of phosphorus limitation in diatoms and their relationship with biomolecule accumulation. Biology 10:565. doi: 10.3390/biology10070565, PMID: 34206287 PMC8301168

[ref24] Lynch F. Santana-Sánchez A. Jämsä M. Sivonen K. Aro E.-M. Allahverdiyeva Y. (2015). Screening native isolates of cyanobacteria and a green alga for integrated wastewater treatment, biomass accumulation and neutral lipid production. Algal Res. 11, 411–420. doi: 10.1016/j.algal.2015.05.015

[ref25] Maldonado J. de los Rios A. Esteve I. Ascaso C. Puyen Z. M. Brambilla C.. (2010). Sequestration and *in vivo* effect of lead on DE2009 microalga, using high-resolution microscopic techniques. J. Hazard. Mater. 183, 44–50. doi: 10.1016/j.jhazmat.2010.06.085 20675042

[ref26] Martin P. Van Mooy B. A. S. (2013). Fluorometric quantification of polyphosphate in environmental plankton samples: extraction protocols, matrix effects, and nucleic acid interference. Appl. Environ. Microbiol. 79, 273–281. doi: 10.1128/AEM.02592-12 23104409 PMC3536087

[ref27] Massimi R. Kirkwood A. E. (2016). Screening microalgae isolated from urban storm- and wastewater systems as feedstock for biofuel. PeerJ 4:e2396. doi: 10.7717/peerj.2396, PMID: 27635353 PMC5012288

[ref28] MWRD (2023). Water reclamation plants data. Monitoring and Research Department Reports. Available at: http://www.mwrd.org/.

[ref29] Nedelciu C. E. Ragnarsdottir K. V. Schlyter P. Stjernquist I. (2020). Global phosphorus supply chain dynamics: assessing regional impact to 2050. Glob. Food Secur. 26:100426. doi: 10.1016/j.gfs.2020.100426, PMID: 32953430 PMC7490587

[ref30] Nichols H. W. Bold H. C. (1965). Trichosarcina polymorpha gen. Et Sp. Nov. J. Phycol. 1, 34–38. doi: 10.1111/j.1529-8817.1965.tb04552.x

[ref31] Nishikawa K. Yamakoshi Y. Uemura I. Tominaga N. (2003). Ultrastructural changes in Chlamydomonas acidophila (Chlorophyta) induced by heavy metals and polyphosphate metabolism. FEMS Microbiol. Ecol. 44, 253–259. doi: 10.1016/S0168-6496(03)00049-7 19719642

[ref32] Ota S. Yoshihara M. Yamazaki T. Takeshita T. Hirata A. Konomi M.. (2016). Deciphering the relationship among phosphate dynamics, electron-dense body and lipid accumulation in the green alga Parachlorella kessleri. Sci. Rep. 6:25731. doi: 10.1038/srep25731, PMID: 27180903 PMC4867602

[ref33] Plöhn M. Spain O. Sirin S. Silva M. Escudero-Oñate C. Ferrando-Climent L.. (2021). Wastewater treatment by microalgae. Physiol. Plant. 173, 568–578. doi: 10.1111/ppl.13427 33860948

[ref34] Powell N. (2009). Biological phosphorus removal by microalgae in waste stabilisation ponds. Ph.D. thesis. Palmerston North: Massey University.

[ref35] Powell N. Shilton A. Chisti Y. Pratt S. (2009). Towards a luxury uptake process via microalgae – defining the polyphosphate dynamics. Water Res. 43, 4207–4213. doi: 10.1016/j.watres.2009.06.011 19616819

[ref36] Rice P. Longden I. Bleasby A. (2000). EMBOSS: the European molecular biology open software suite. Trends Genet. 16, 276–277. doi: 10.1016/S0168-9525(00)02024-2 10827456

[ref37] Rippka R. Deruelles J. Waterbury J. B. Herdman M. Stanier R. Y. (1979). Generic assignments, strain histories and properties of pure cultures of cyanobacteria. Microbiology 111, 1–61. doi: 10.1099/00221287-111-1-1

[ref38] Sanz-Luque E. Bhaya D. Grossman A. R. (2020). Polyphosphate: a multifunctional metabolite in Cyanobacteria and algae. Front. Plant Sci. 11:938. doi: 10.3389/fpls.2020.00938 32670331 PMC7332688

[ref39] Sena M. Hicks A. (2018). Life cycle assessment review of struvite precipitation in wastewater treatment. Resour. Conserv. Recycl. 139, 194–204. doi: 10.1016/j.resconrec.2018.08.009

[ref40] Siciliano A. Limonti C. Curcio G. M. Molinari R. (2020). Advances in struvite precipitation Technologies for Nutrients Removal and Recovery from aqueous waste and wastewater. Sustainability 12:7538. doi: 10.3390/su12187538

[ref41] Slocombe S. P. Zúñiga-Burgos T. Chu L. Wood N. J. Camargo-Valero M. A. Baker A. (2020). Fixing the broken phosphorus cycle: wastewater remediation by microalgal polyphosphates. Front. Plant Sci. 11:982. doi: 10.3389/fpls.2020.00982, PMID: 32695134 PMC7339613

[ref42] Stothard P. (2000). The sequence manipulation suite: Java script programs for analyzing and formatting protein and DNA sequences. Bio Techniques 28, 1102–1104. doi: 10.2144/00286ir01 10868275

[ref43] Suresh Kumar K. Dahms H.-U. Won E.-J. Lee J.-S. Shin K.-H. (2015). Microalgae – a promising tool for heavy metal remediation. Ecotoxicol. Environ. Saf. 113, 329–352. doi: 10.1016/j.ecoenv.2014.12.019 25528489

[ref44] Sweiss M. (2017). Microalgae for wastewater treatment and biomass production from bioprospecting to biotechnology. Ph.D. thesis. Bath: University of Bath.

[ref45] Voronkov A. Sinetova M. (2019). Polyphosphate accumulation dynamics in a population of Synechocystis sp. PCC 6803 cells under phosphate overplus. Protoplasma 256, 1153–1164. doi: 10.1007/s00709-019-01374-2 30972564

[ref46] Voumard M. Giannakis S. Carratalà A. Pulgarin C. (2019). *E. coli* – MS2 bacteriophage interactions during solar disinfection of wastewater and the subsequent post-irradiation period. Chem. Eng. J. 359, 1224–1233. doi: 10.1016/j.cej.2018.11.055

[ref47] Wollmann F. Dietze S. Ackermann J. Bley T. Walther T. Steingroewer J.. (2019). Microalgae wastewater treatment: biological and technological approaches. Eng. Life Sci. 19, 860–871. doi: 10.1002/elsc.201900071, PMID: 32624978 PMC6999062

[ref48] Wu P. Zhang Z. Luo Y. Bai Y. Fan J. (2022). Bioremediation of phenolic pollutants by algae - current status and challenges. Bioresour. Technol. 350:126930. doi: 10.1016/j.biortech.2022.126930, PMID: 35247559

[ref49] Wurtsbaugh W. A. Paerl H. W. Dodds W. K. (2019). Nutrients, eutrophication and harmful algal blooms along the freshwater to marine continuum. WIREs Water 6:e1373. doi: 10.1002/wat2.1373

[ref50] Yuan Z. Jiang S. Sheng H. Liu X. Hua H. Liu X.. (2018). Human perturbation of the global phosphorus cycle: changes and consequences. Environ. Sci. Technol. 52, 2438–2450. doi: 10.1021/acs.est.7b03910 29402084

[ref51] Yuan Z. Pratt S. Batstone D. J. (2012). Phosphorus recovery from wastewater through microbial processes. Curr. Opin. Biotechnol. 23, 878–883. doi: 10.1016/j.copbio.2012.08.001 22922003

[ref52] Zhao X. Kumar K. Gross M. A. Kunetz T. E. Wen Z. (2018). Evaluation of revolving algae biofilm reactors for nutrients and metals removal from sludge thickening supernatant in a municipal wastewater treatment facility. Water Res. 143, 467–478. doi: 10.1016/j.watres.2018.07.001 29986255

[ref53] Zhou H. Zhao X. Kumar K. Kunetz T. Zhang Y. Gross M.. (2021). Removing high concentration of nickel (II) ions from synthetic wastewater by an indigenous microalgae consortium with a revolving algal biofilm (RAB) system. Algal Res. 59:102464. doi: 10.1016/j.algal.2021.102464

