# Supplementary material for: Isolation of phosphorus-hyperaccumulating microalgae from revolving algal biofilm (RAB) wastewater treatment systems
Source: Front Microbiol. 2023 Jul 17;14:1219318. doi: 10.3389/fmicb.2023.1219318 (PMC10389661; doi:10.3389/fmicb.2023.1219318)
Supplement: Supplementary file 1 [file Data_Sheet_1.zip › Supplementary Material - Schaedig et al. (2023)/Supplemental File S1.pdf]

Isolation of Phosphorus-Hyperaccumulating Microalgae from Revolving Algal Biofilm (RAB) Wastewater Treatment Systems

Eric Schaedig, Michael Cantrell, Chris Urban, Xuefei Zhao, Drew Greene, Jens Dancer, Michael Gross, Jacob Sebesta, Katherine J. Chou, Jonathan Grabowy, Martin Gross, Kuldip Kumar, and Jianping Yu

Supplemental File S1. Microscopy image bank. Representative microscopy images of each unique strain isolated in this study and of the RAB microalgae consortium included in both the polyphosphate screening and bench-scale RAB testing.

Table of Contents

Green Algae……………………………………………………3
Cyanobacteria…………………………………………86
Diatoms……………………………………………………97
RAB Microalgae Consortium……………………107

Green Algae

Dictyosphaerium sp. TCF-1g

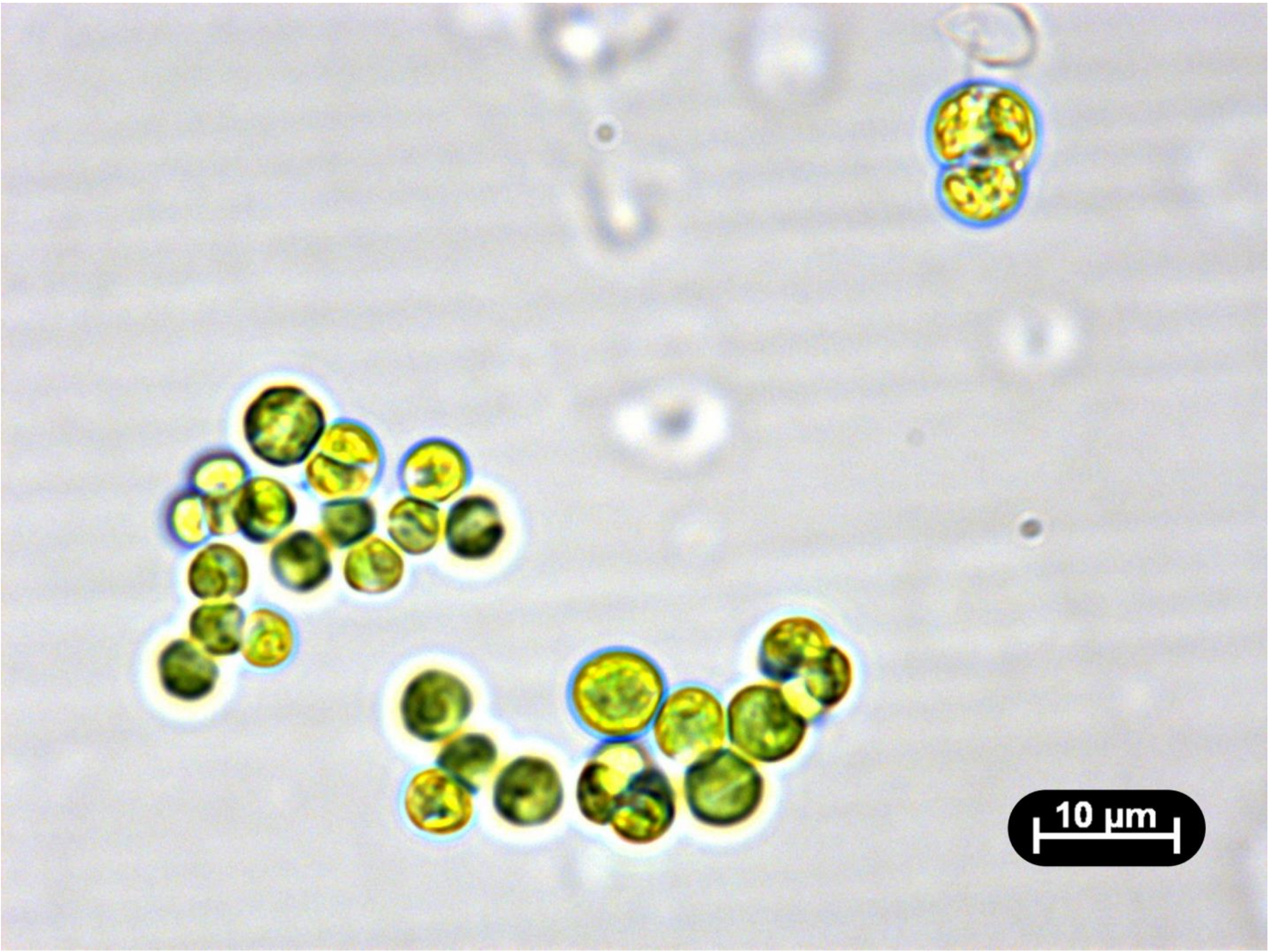

Chlorolobion sp. TCF-2g

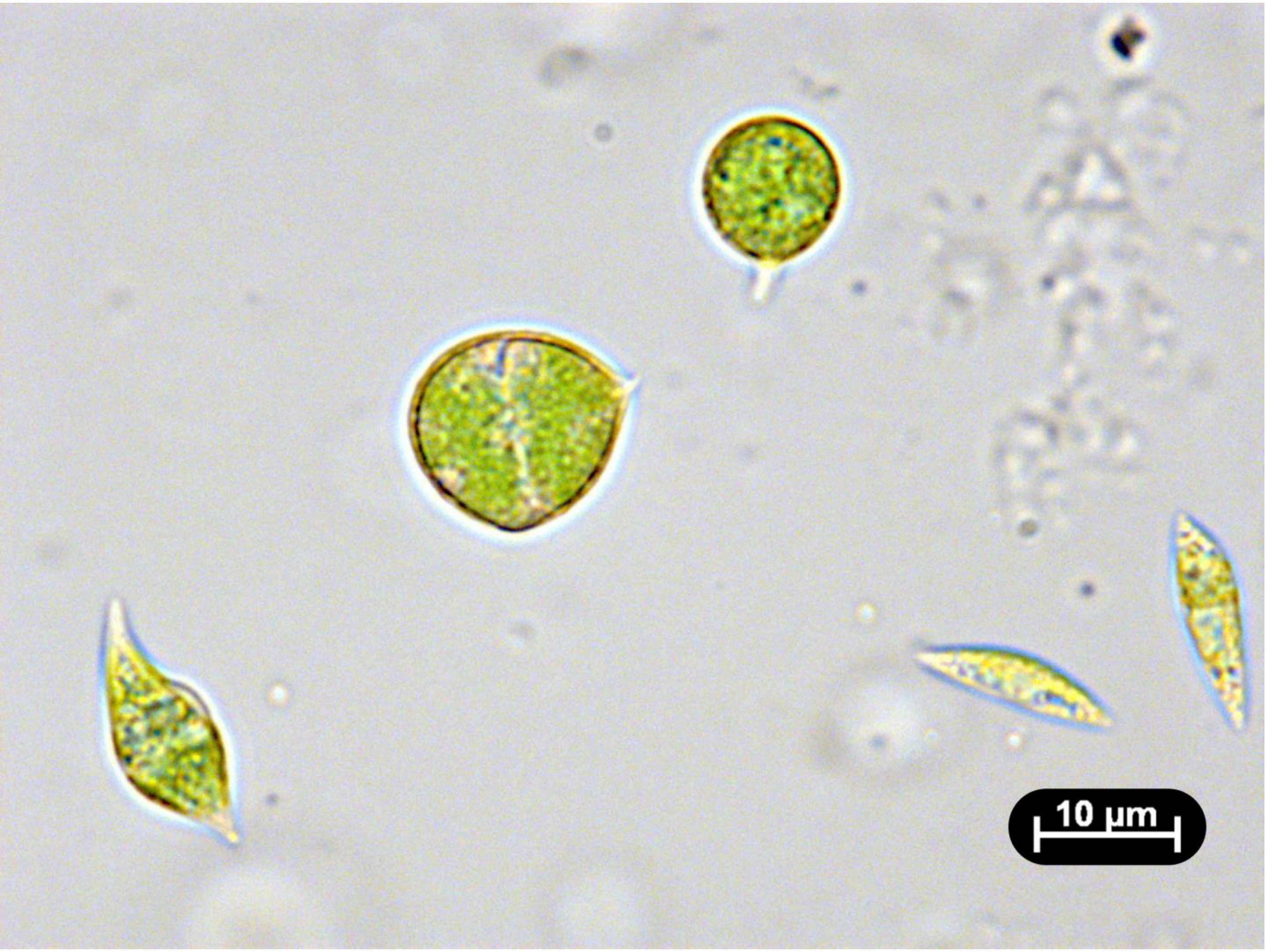

Desmodesmus sp. TCF-3g

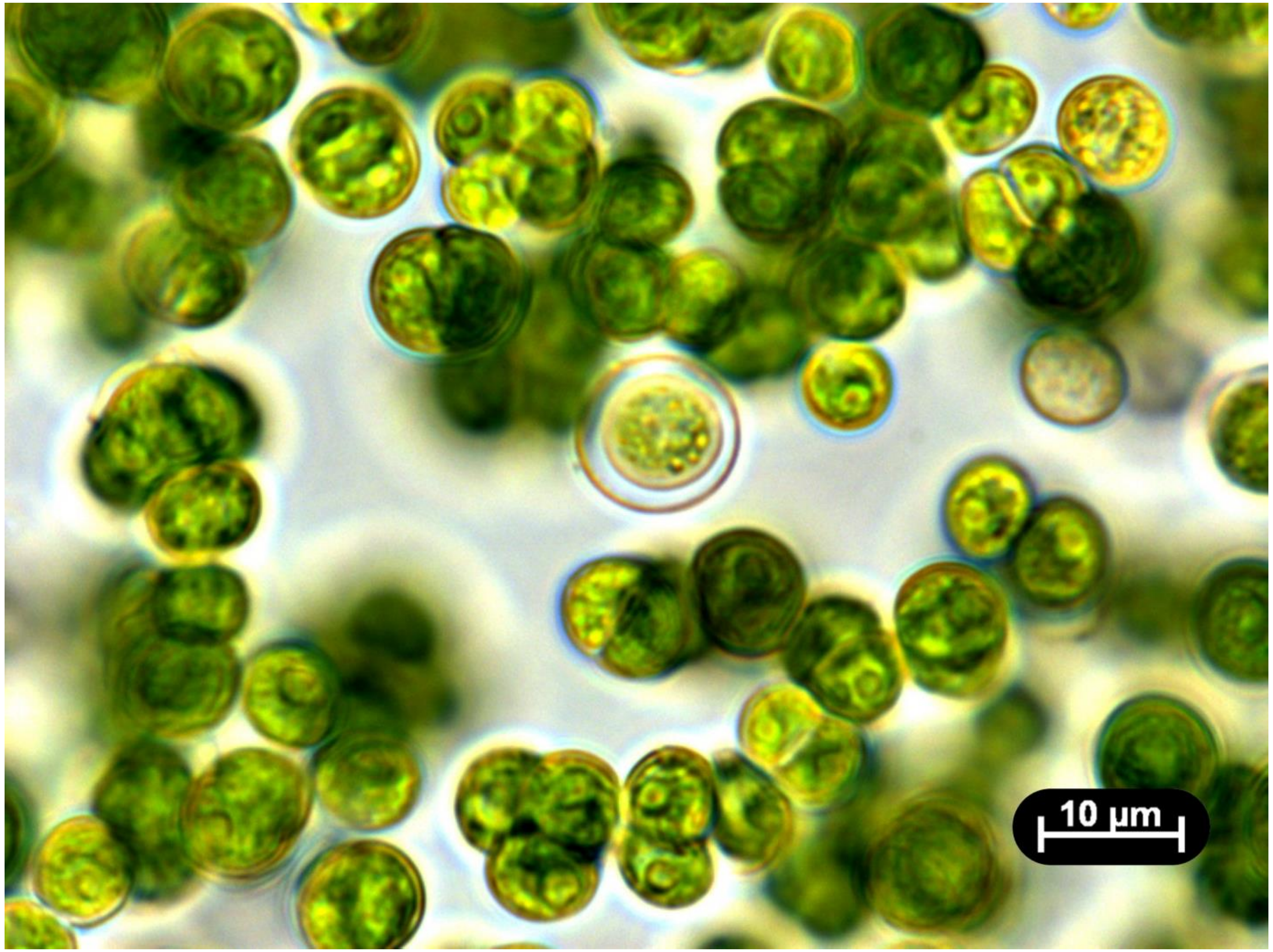

Desmodesmus sp. TCF-4g

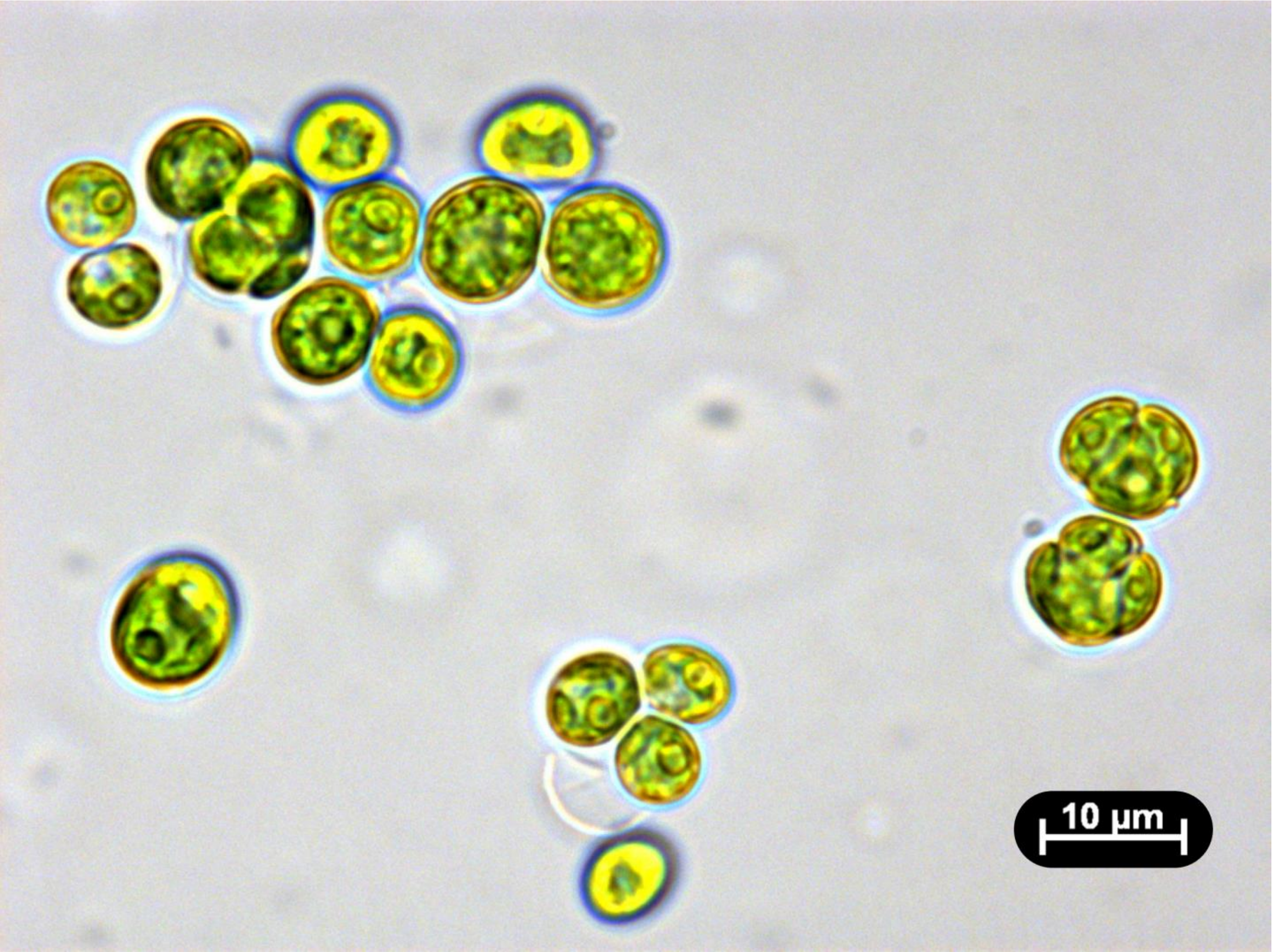

Chlorolobion sp. TCF-5g

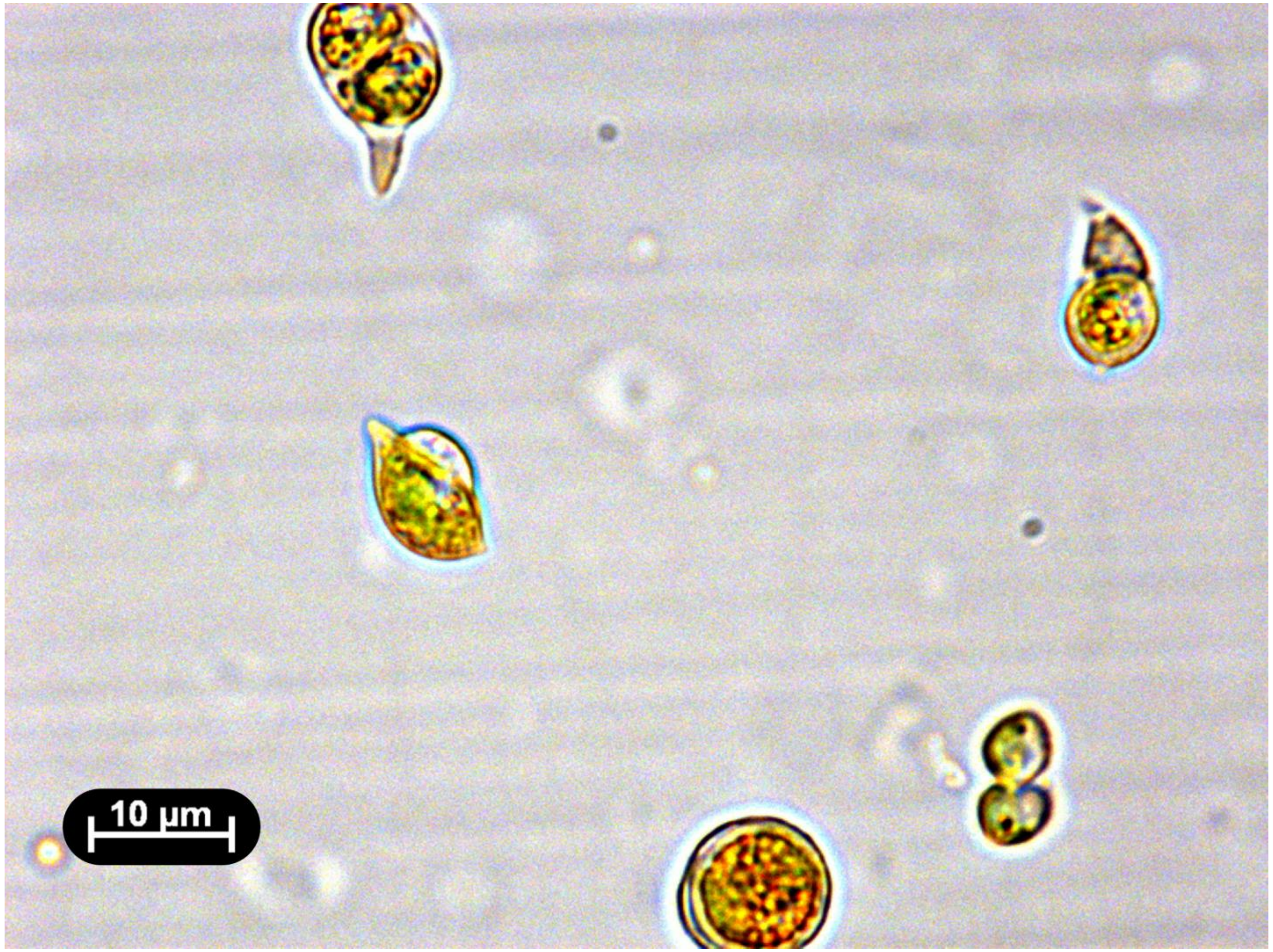

Desmodesmus abundans TCF-6g

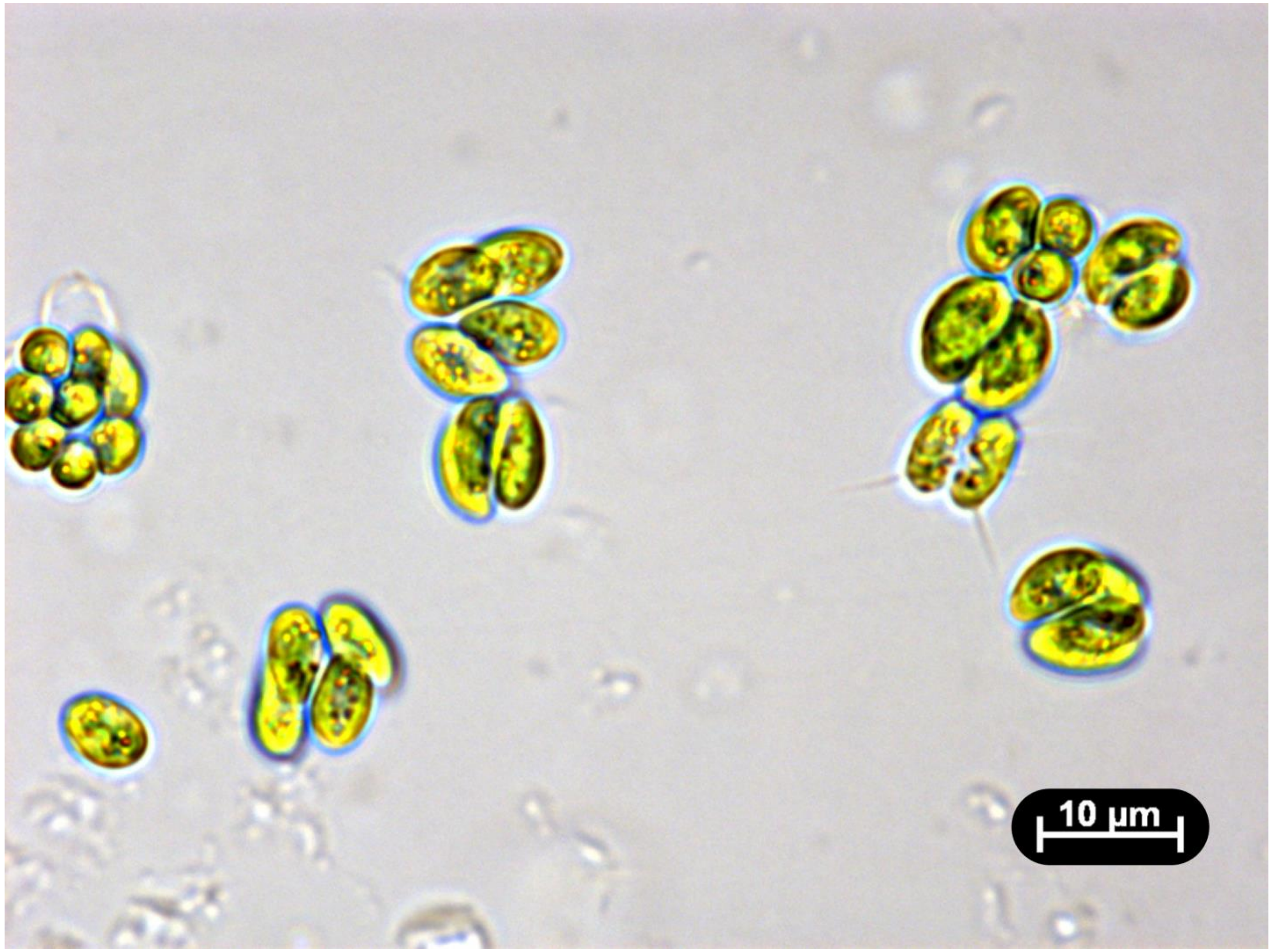

Desmodesmus abundans TCF-7g

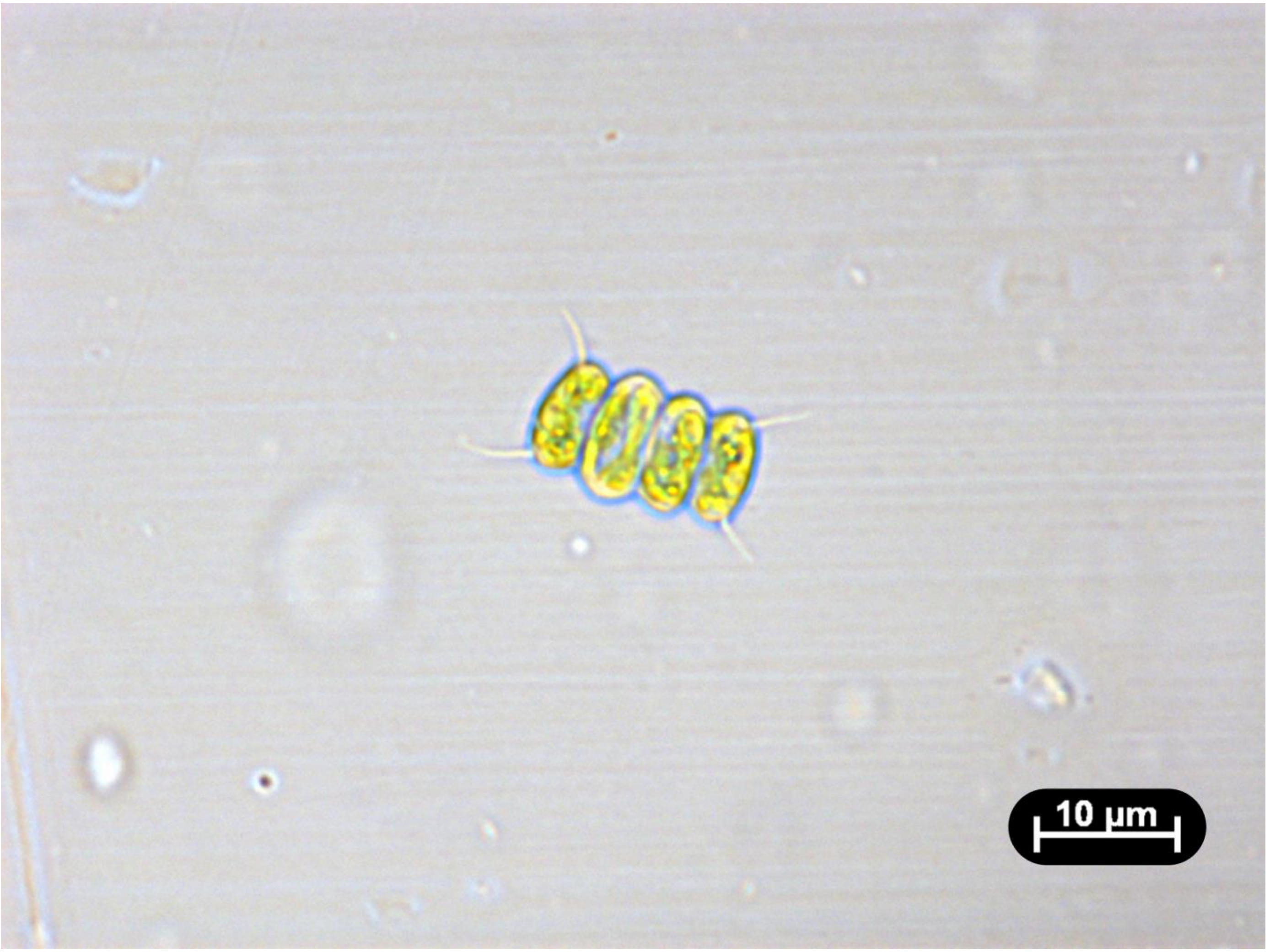

Dictyosphaerium sp. TCF-8g

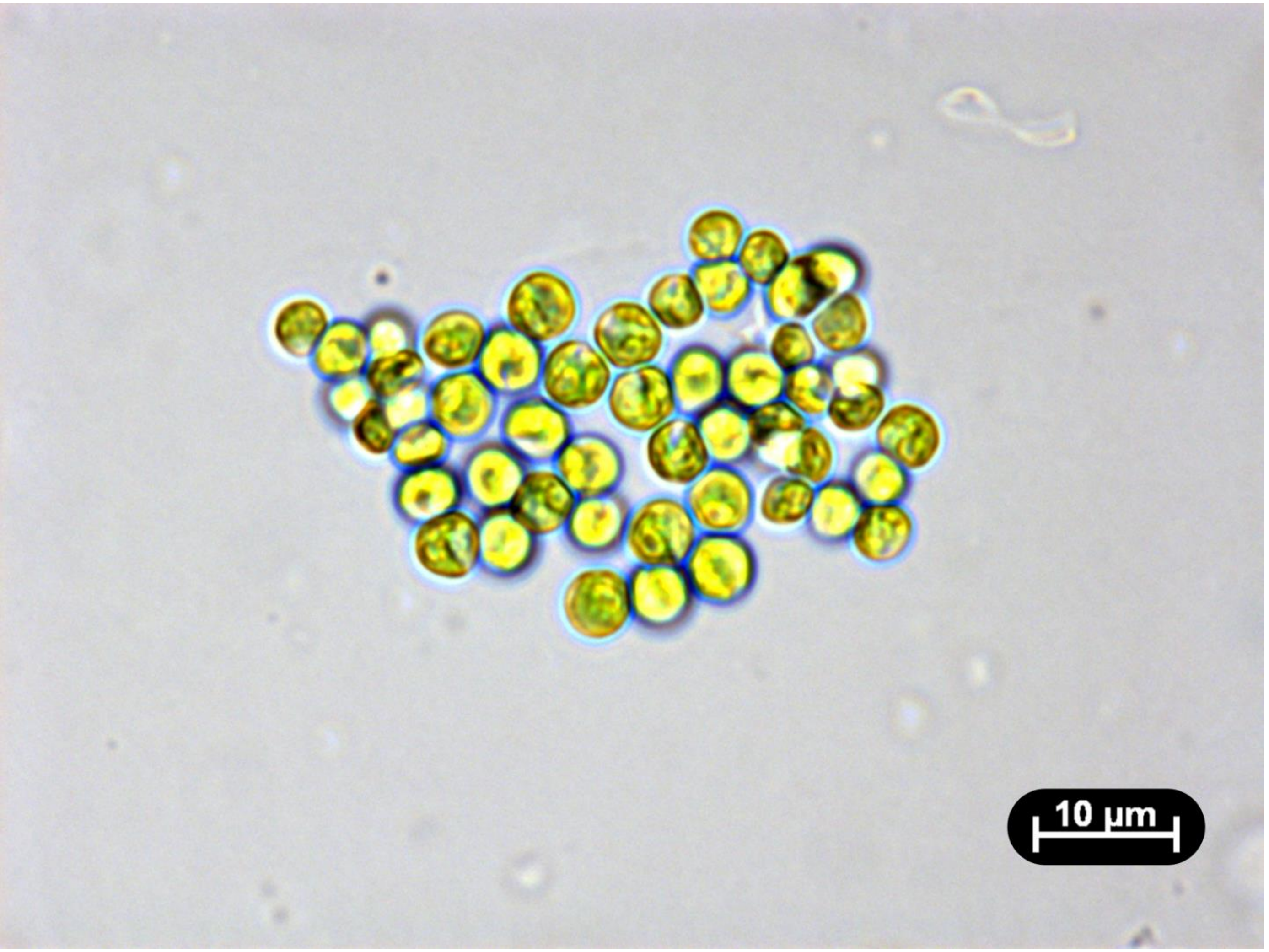

Dictyosphaerium sp. TCF-9g

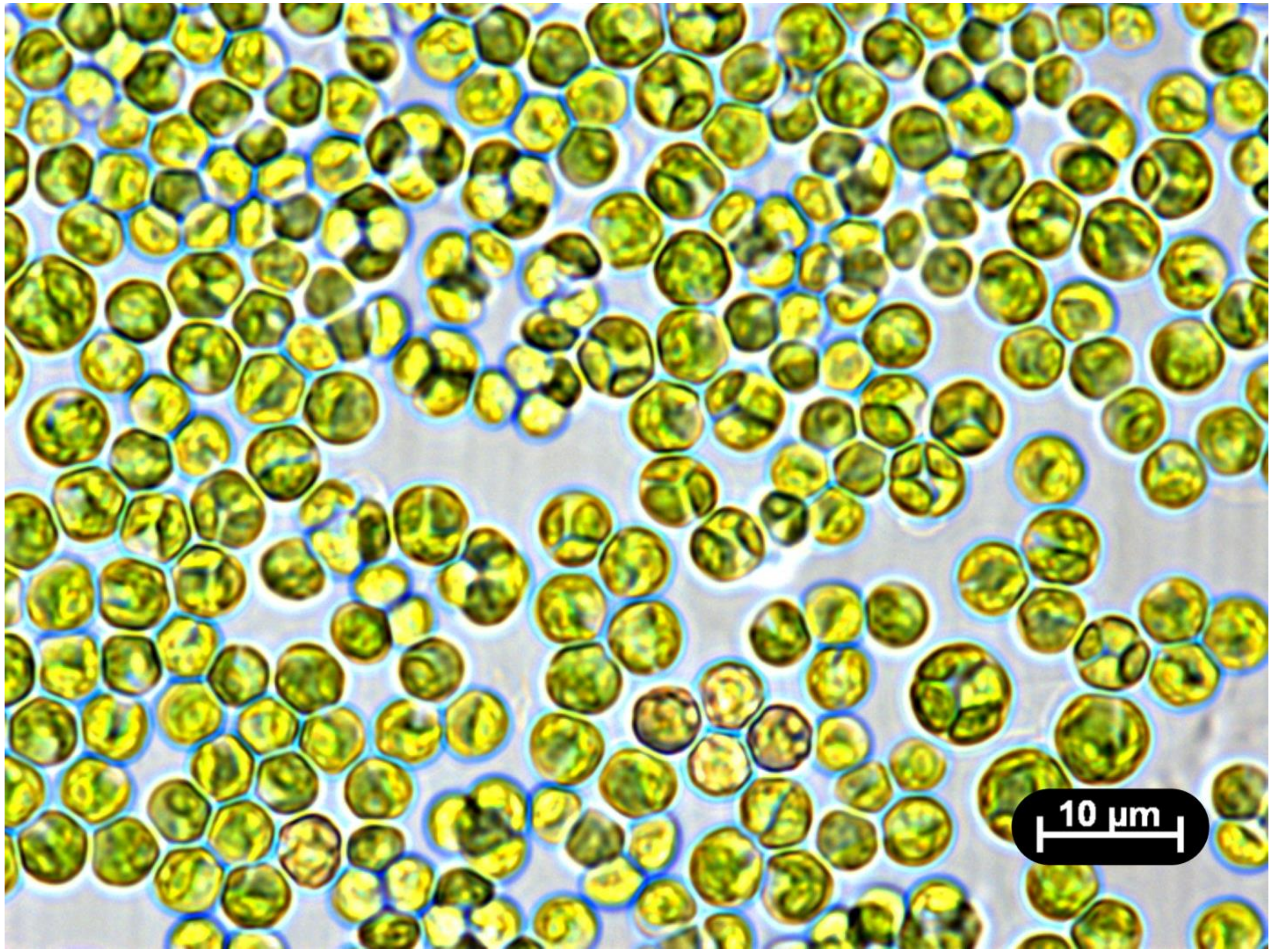

Dictyosphaerium sp. TCF-10g

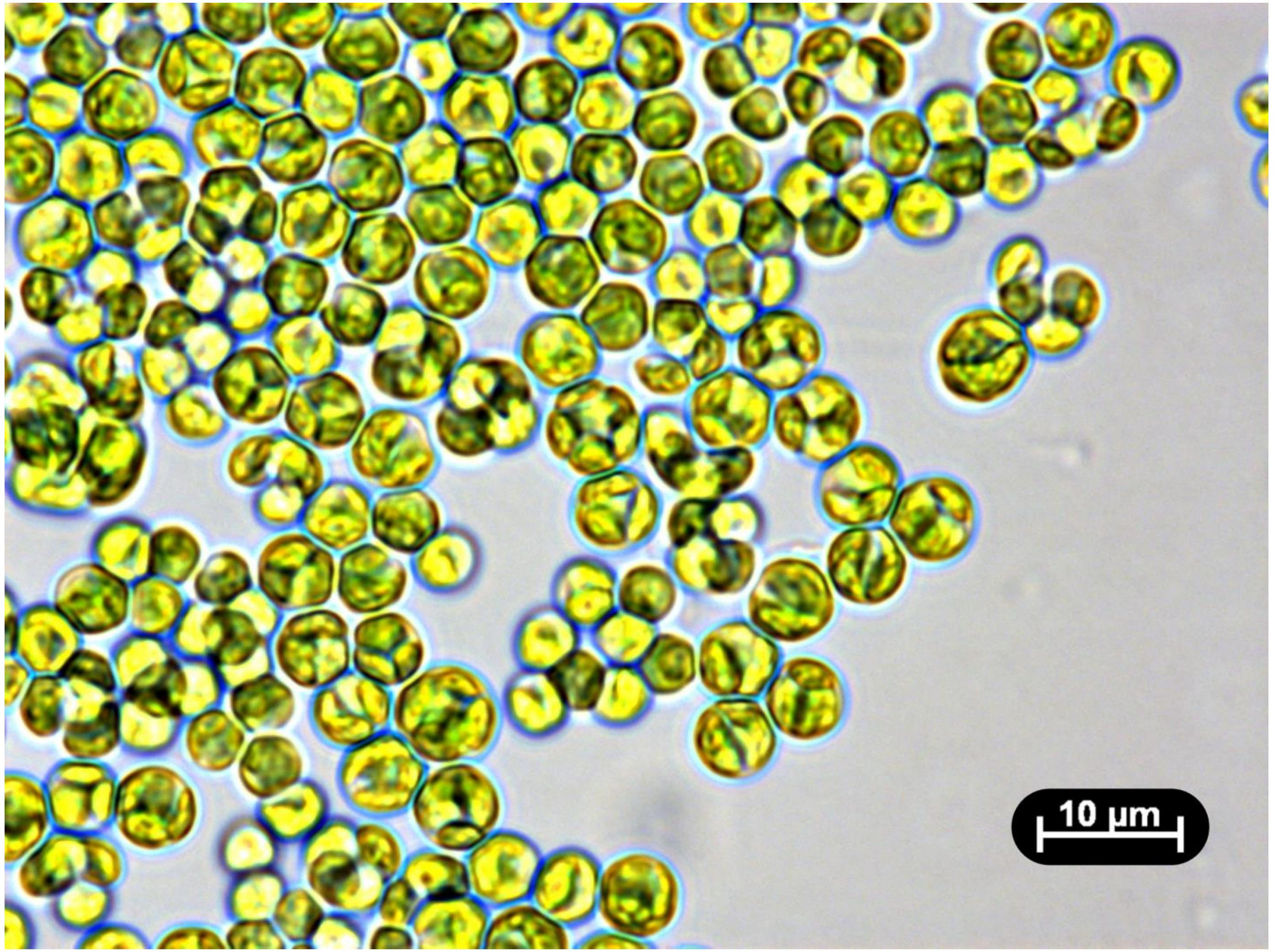

Dictyosphaerium sp. TCF-11g

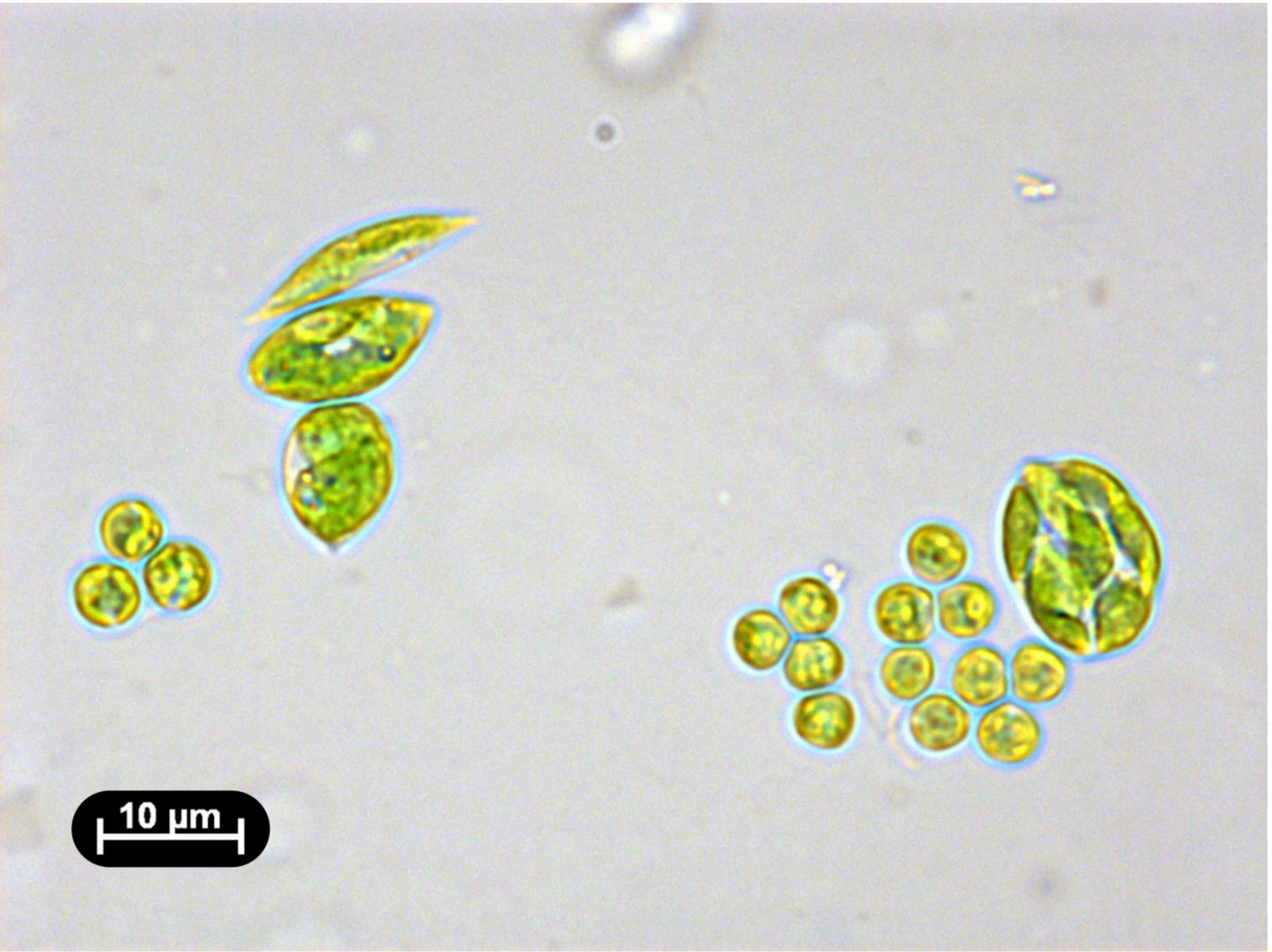

*Isolate appears to contain more than one strain.

Dictyosphaerium sp. TCF-12g

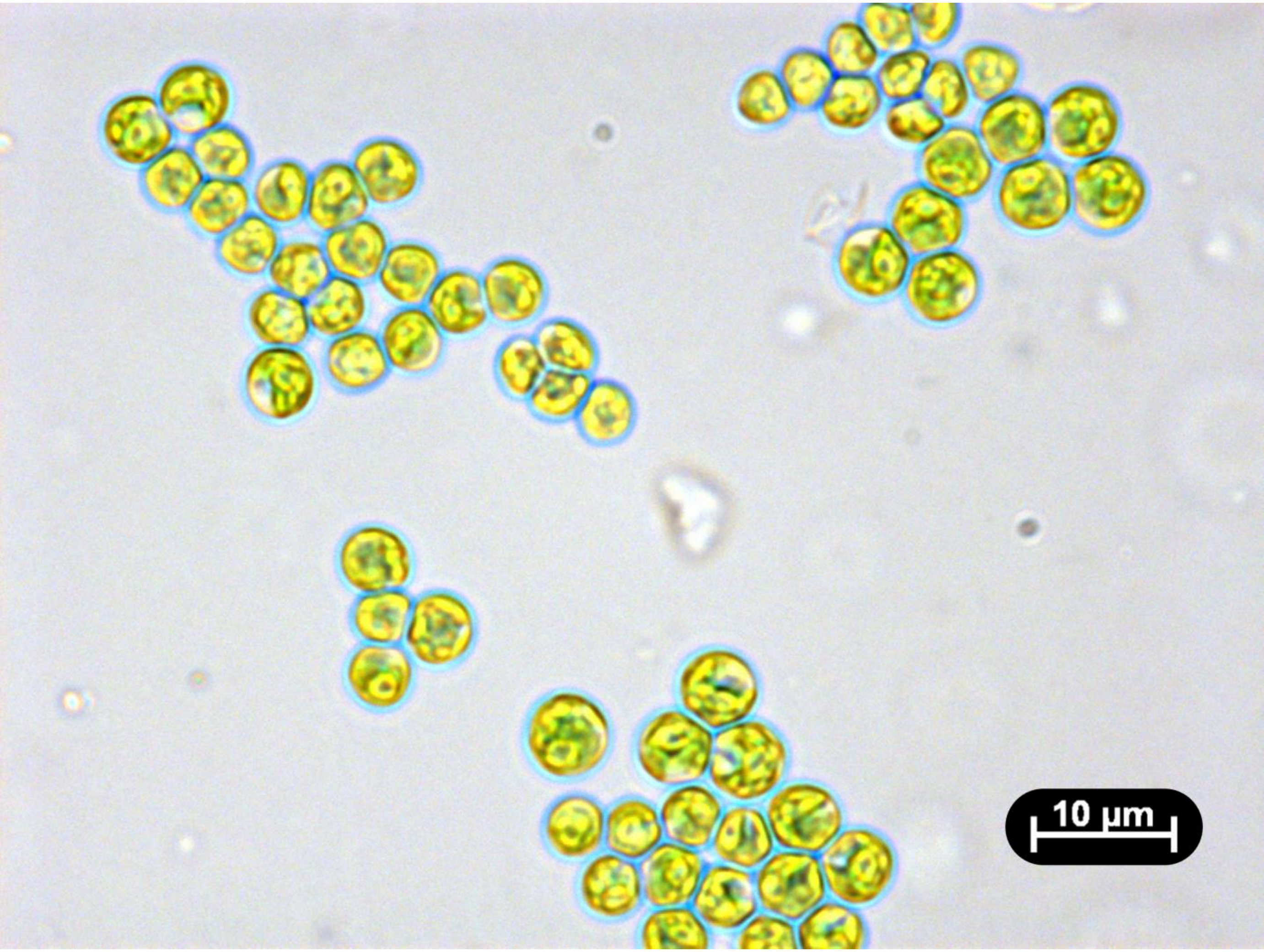

Chlorella sorokiniana TCF-13g

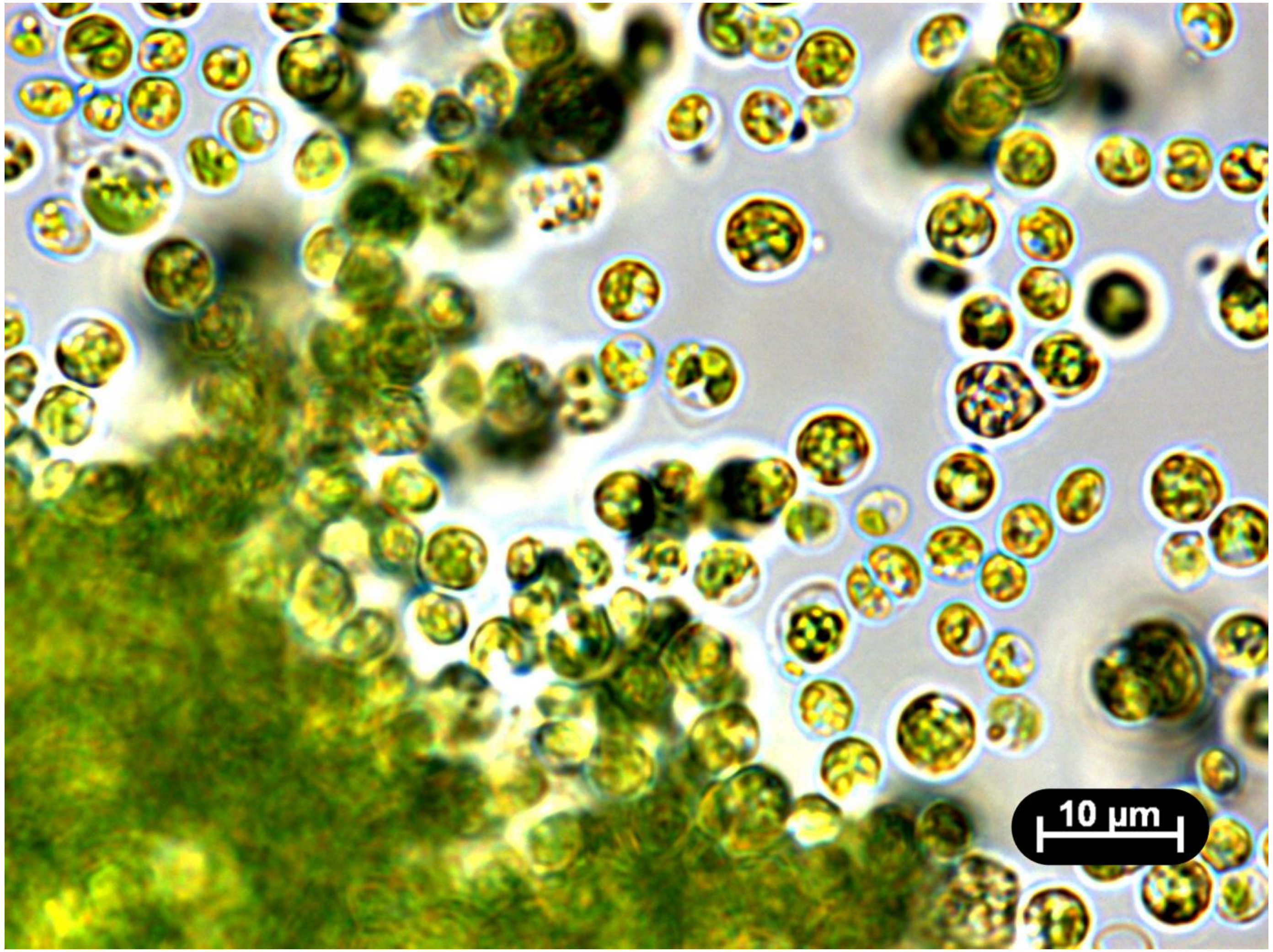

Desmodesmus armatus TCF-14g

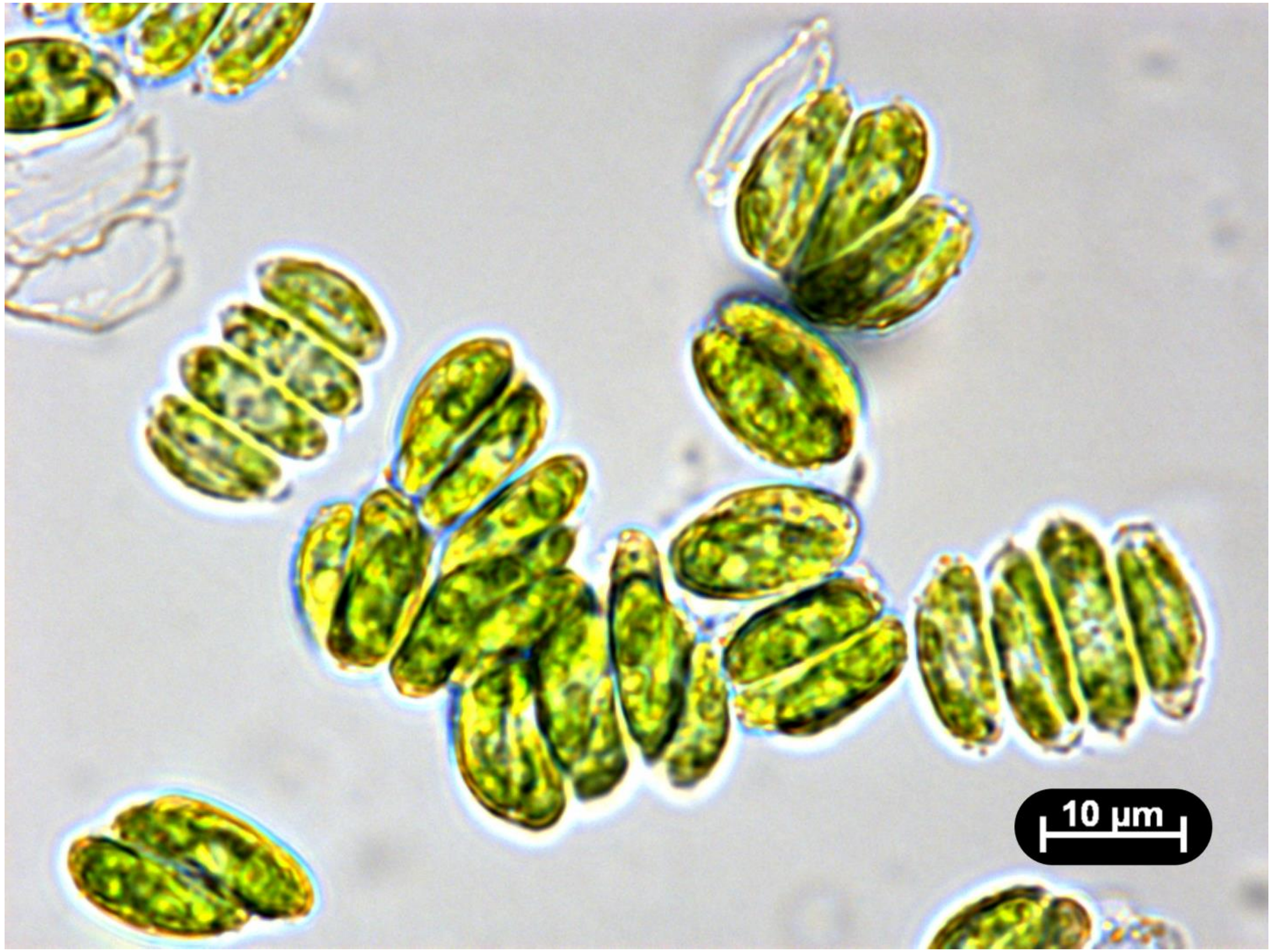

Desmodesmus sp. TCF-15g

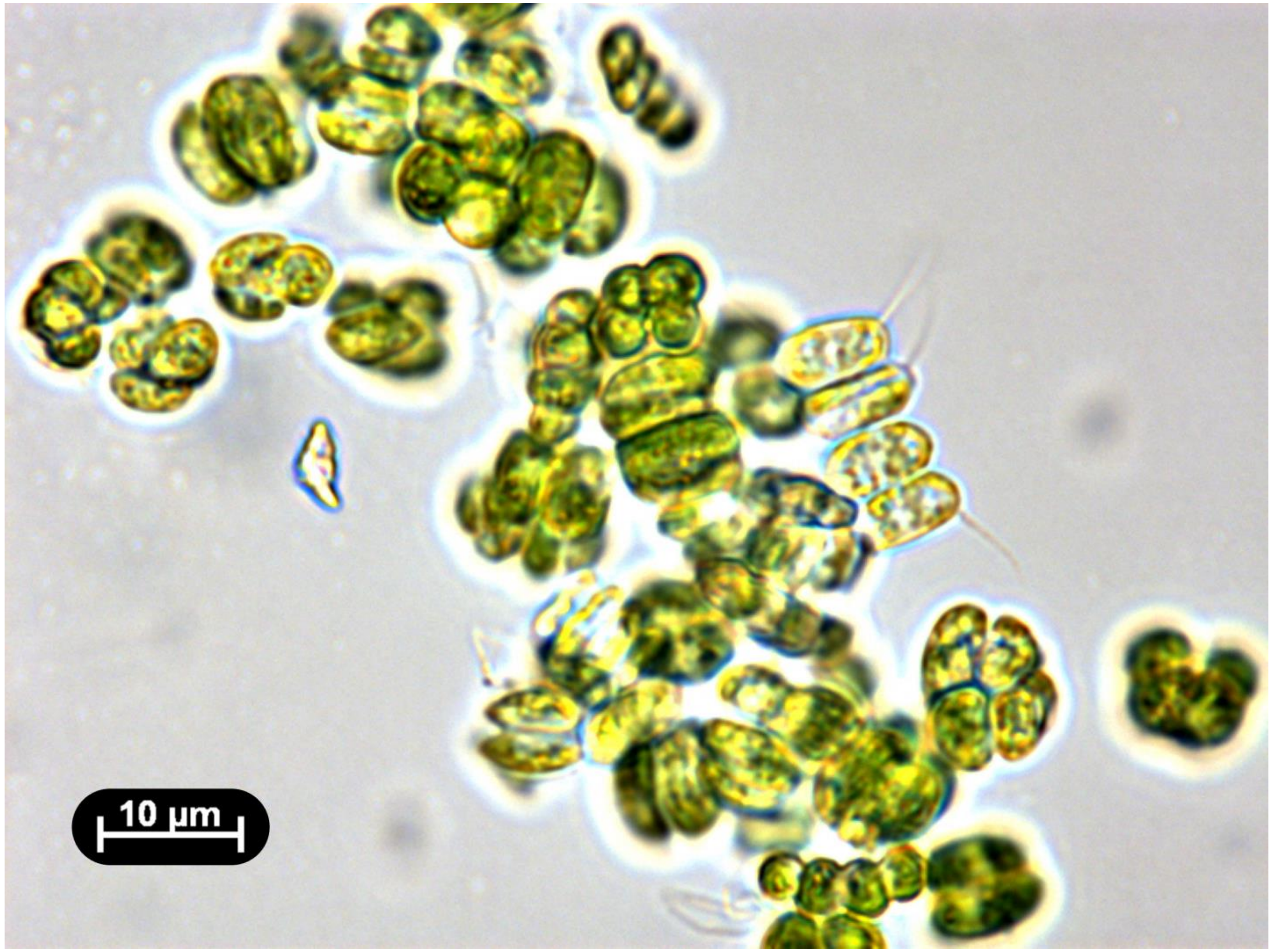

Tetradesmus sp. TCF-16g

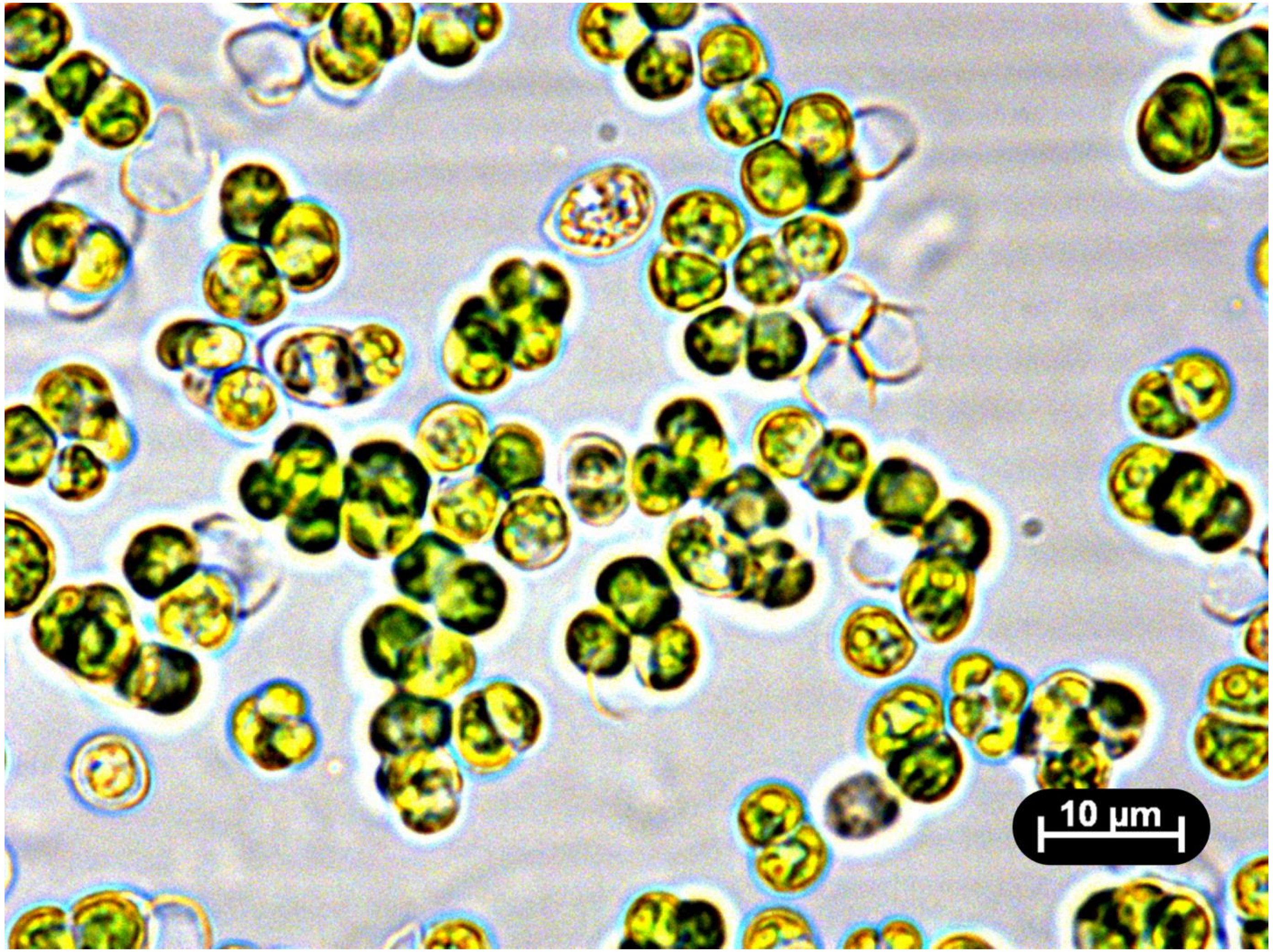

Chlorellaceae sp. TCF-17g

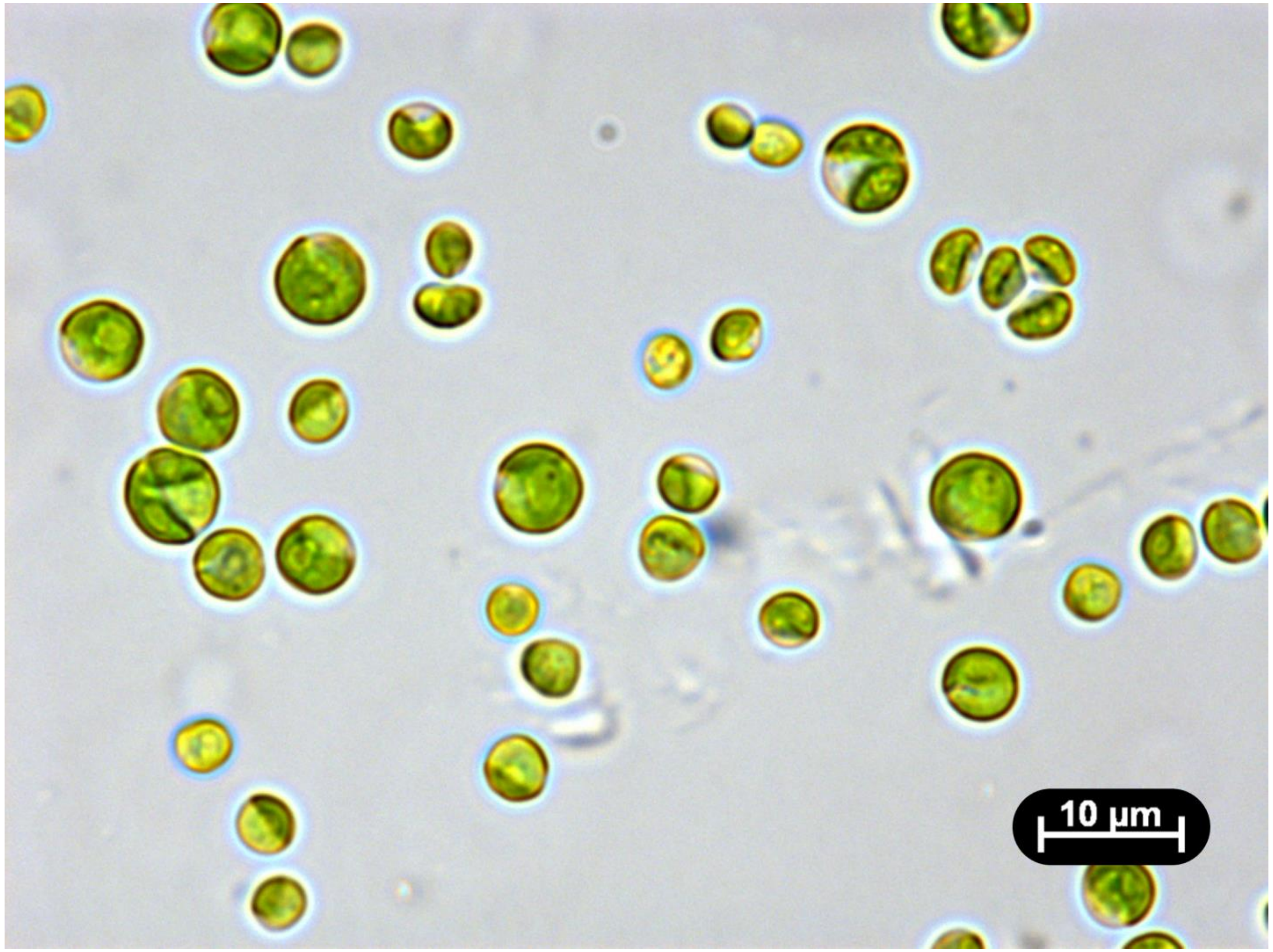

Chlorellaceae sp. TCF-18g

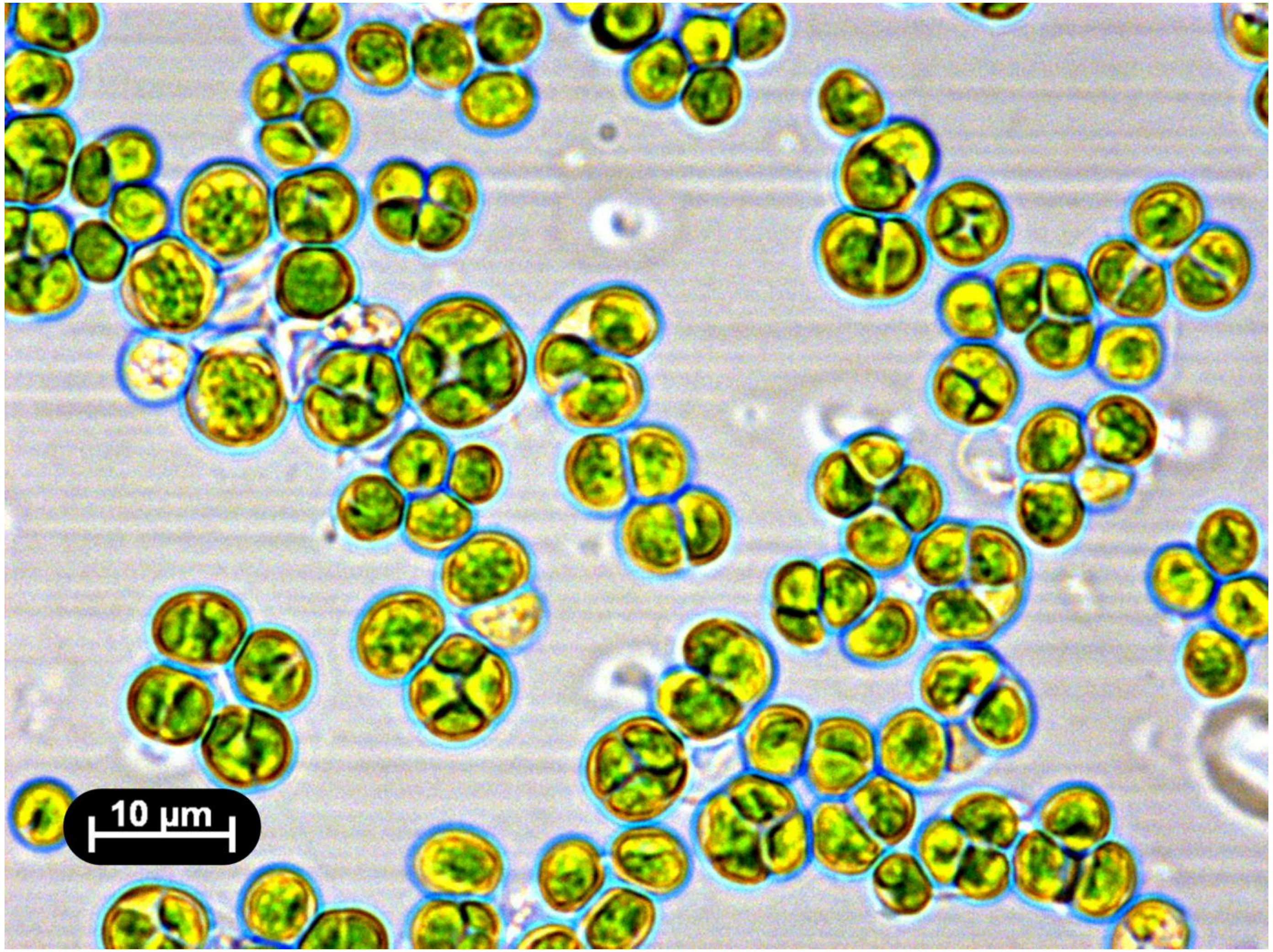

Scenedesmus sp. TCF-19g

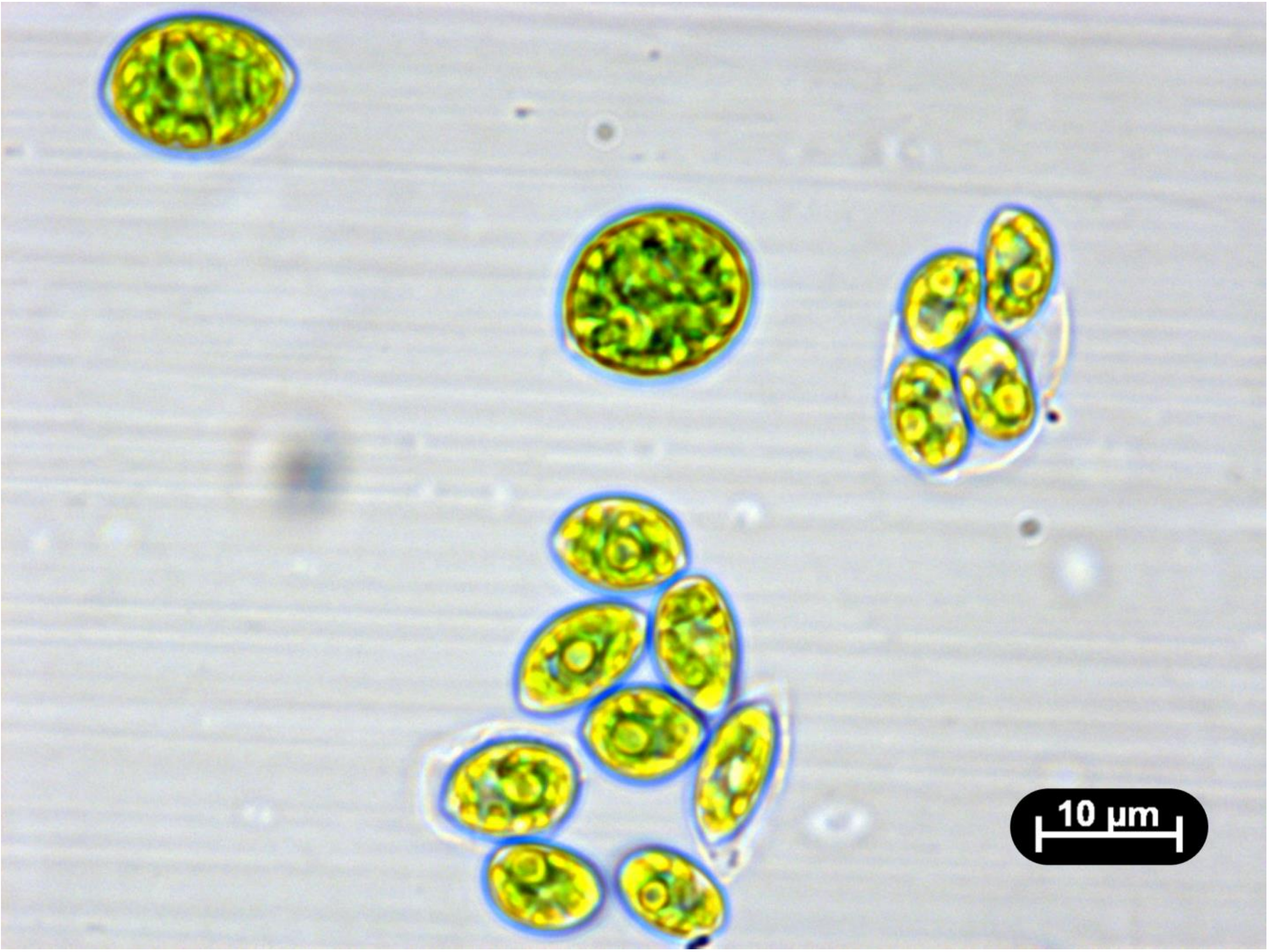

Chlorella sorokiniana TCF-20g

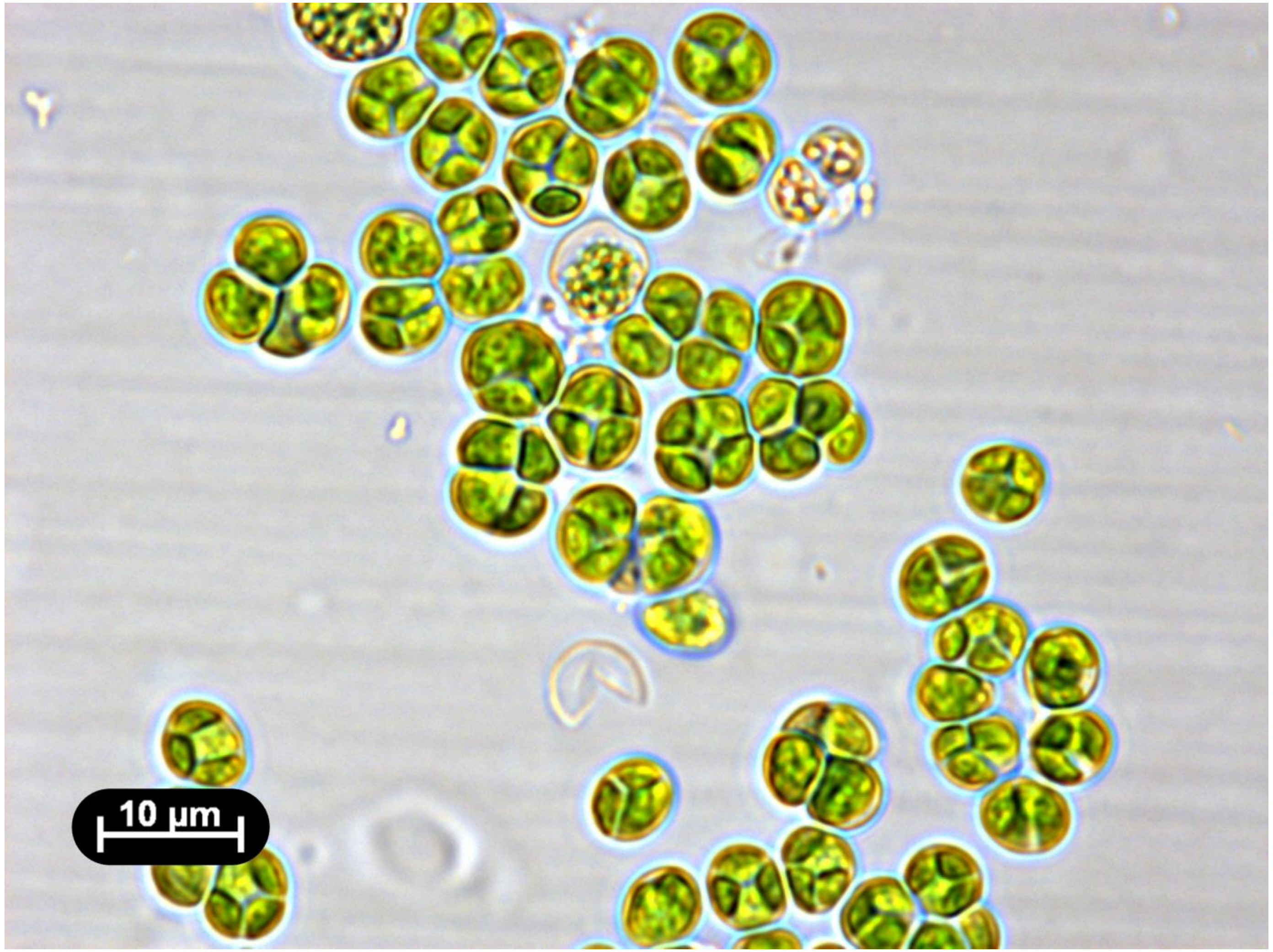

Tetradesmus obliquus TCF-21g

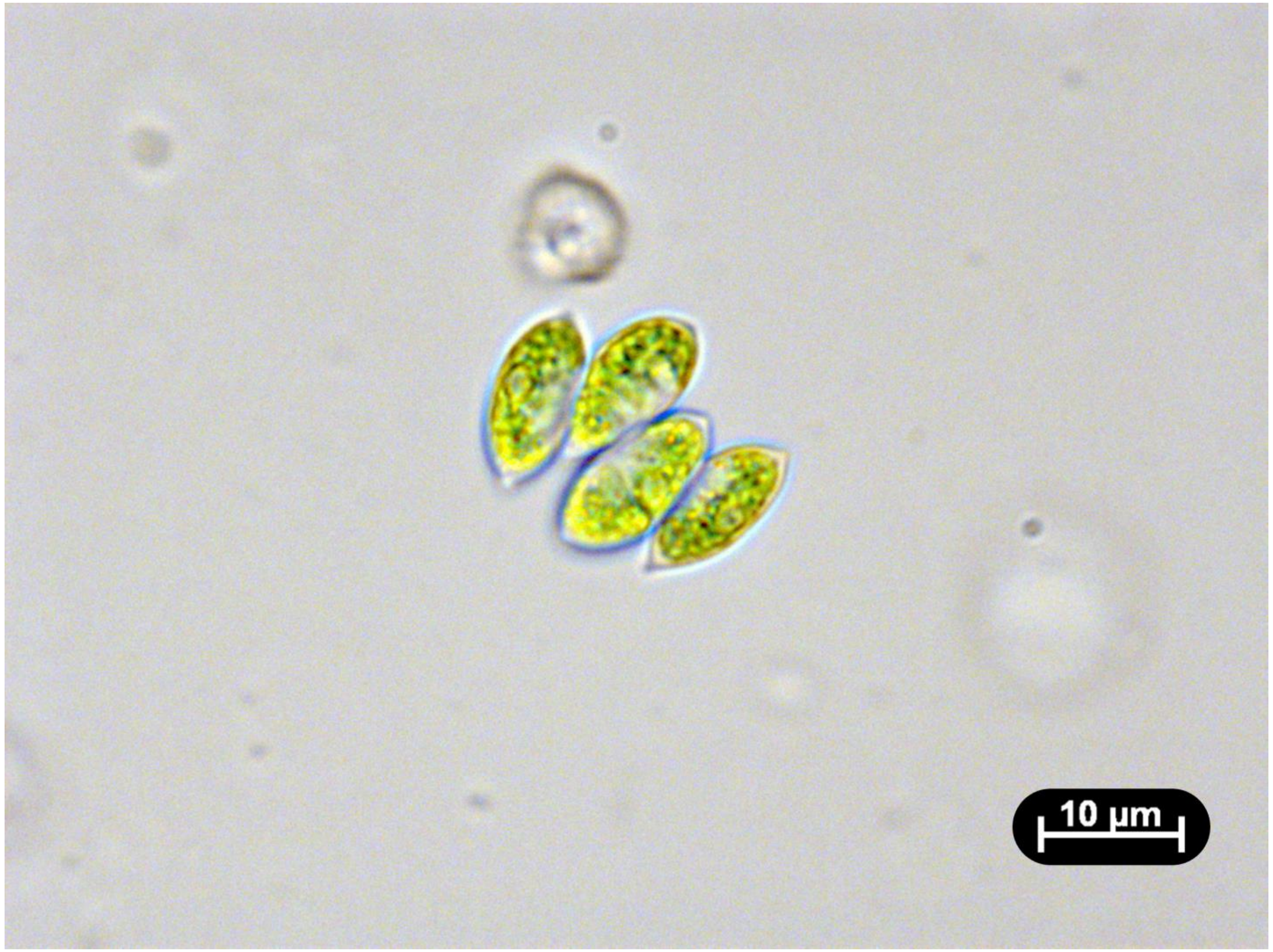

Chlorolobion sp. TCF-22g

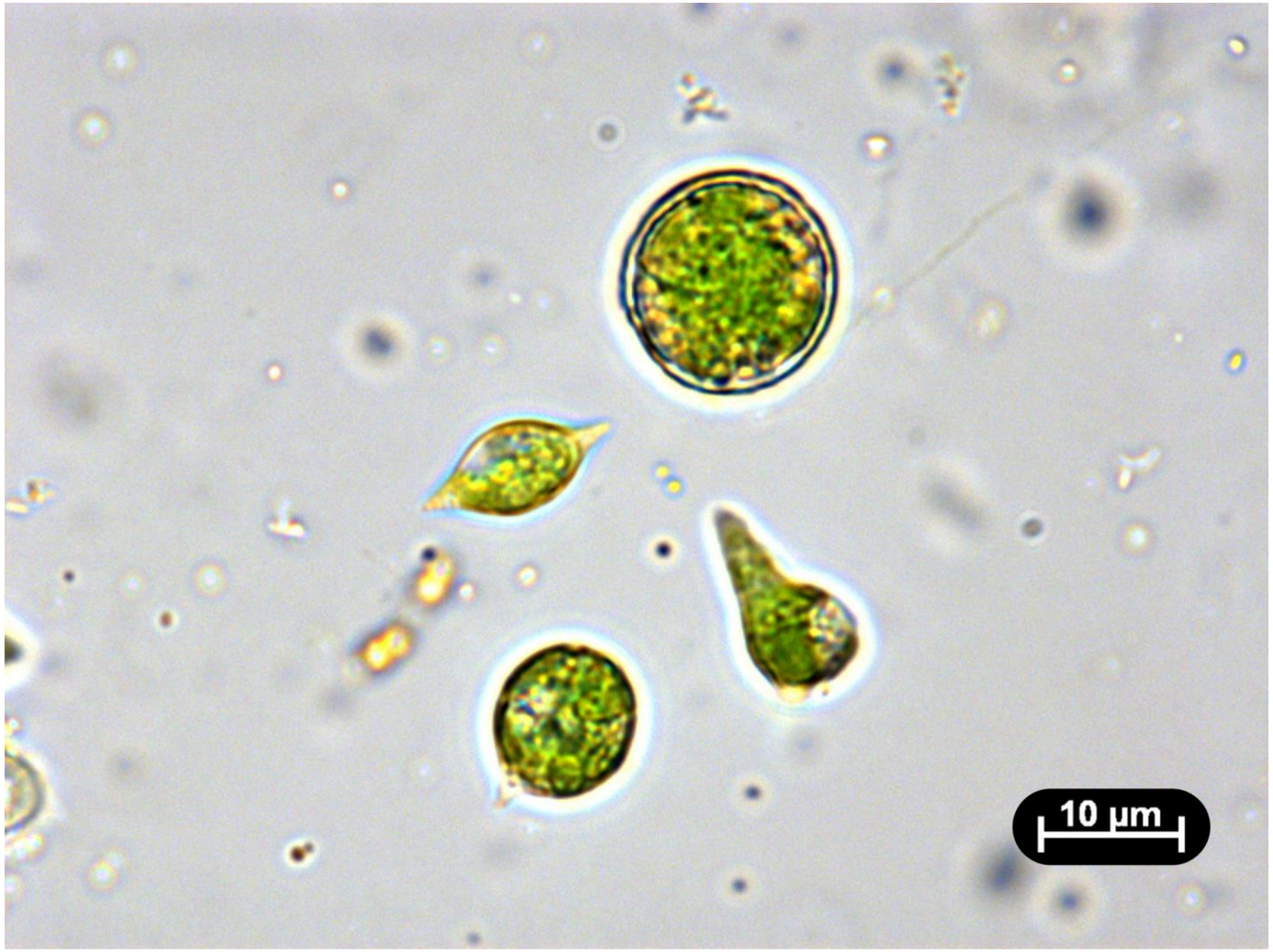

Chlorella sorokiniana TCF-23g

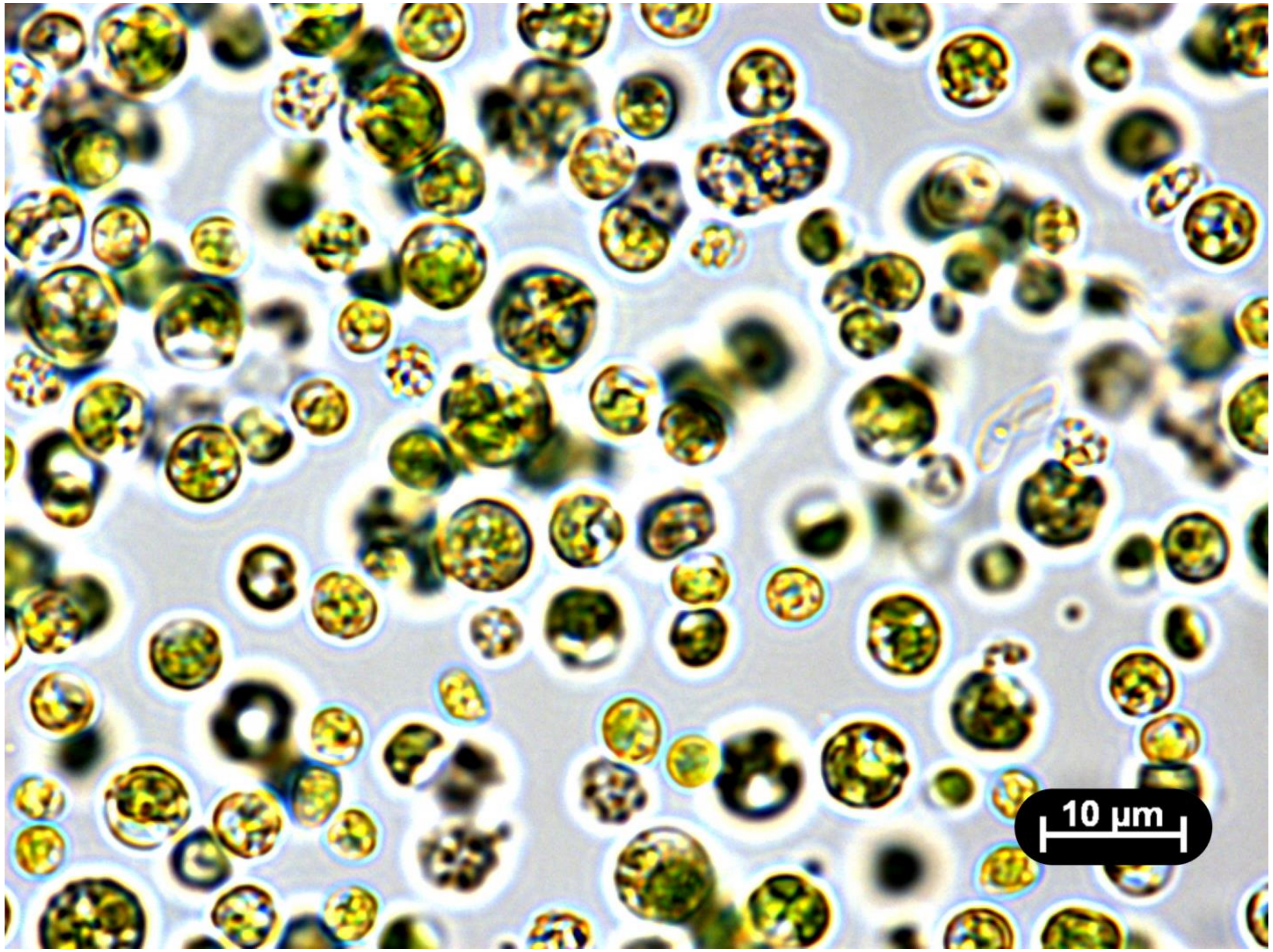

Chlorella sorokiniana TCF-24g

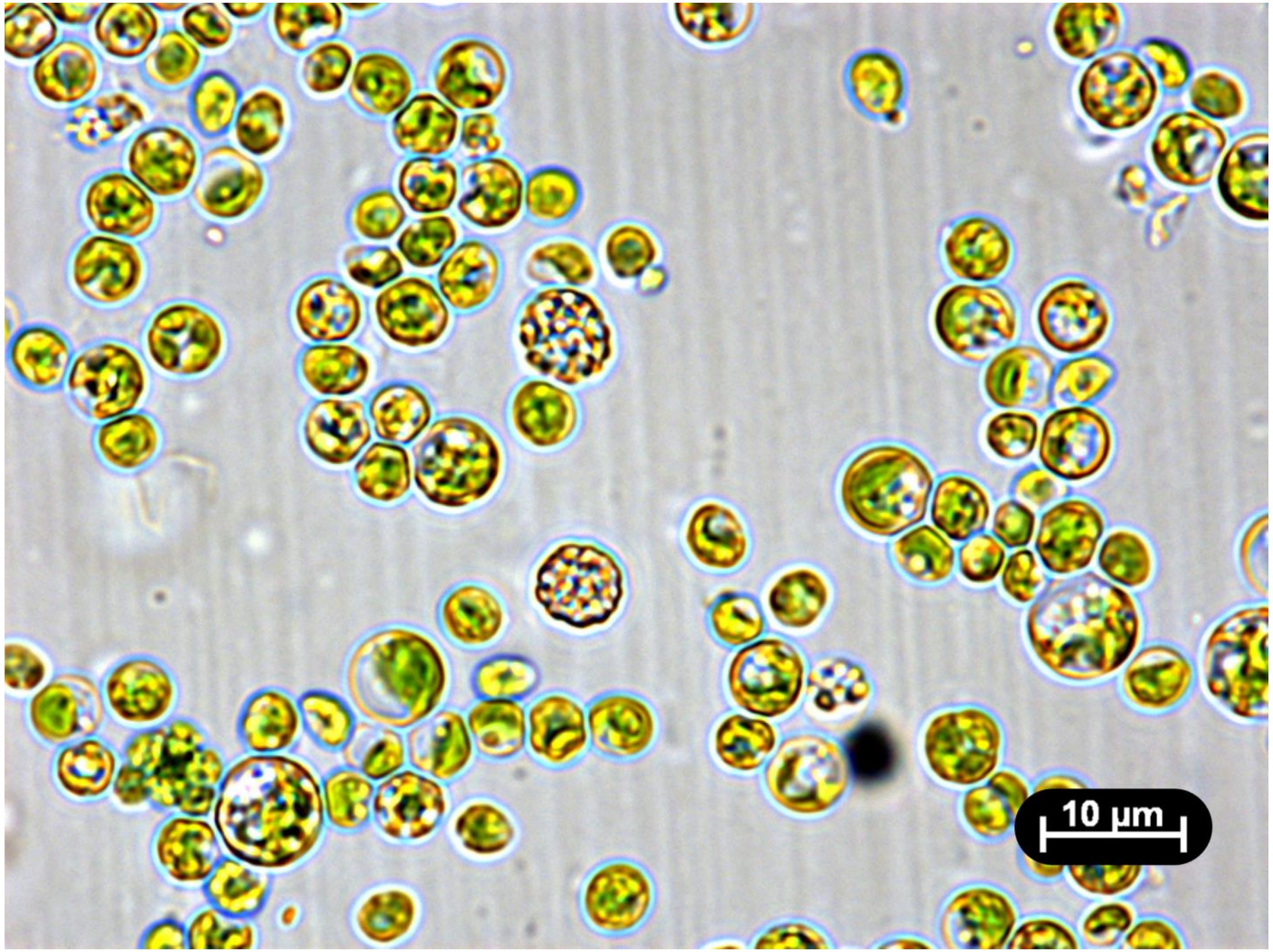

Desmodesmus abundans TCF-25g

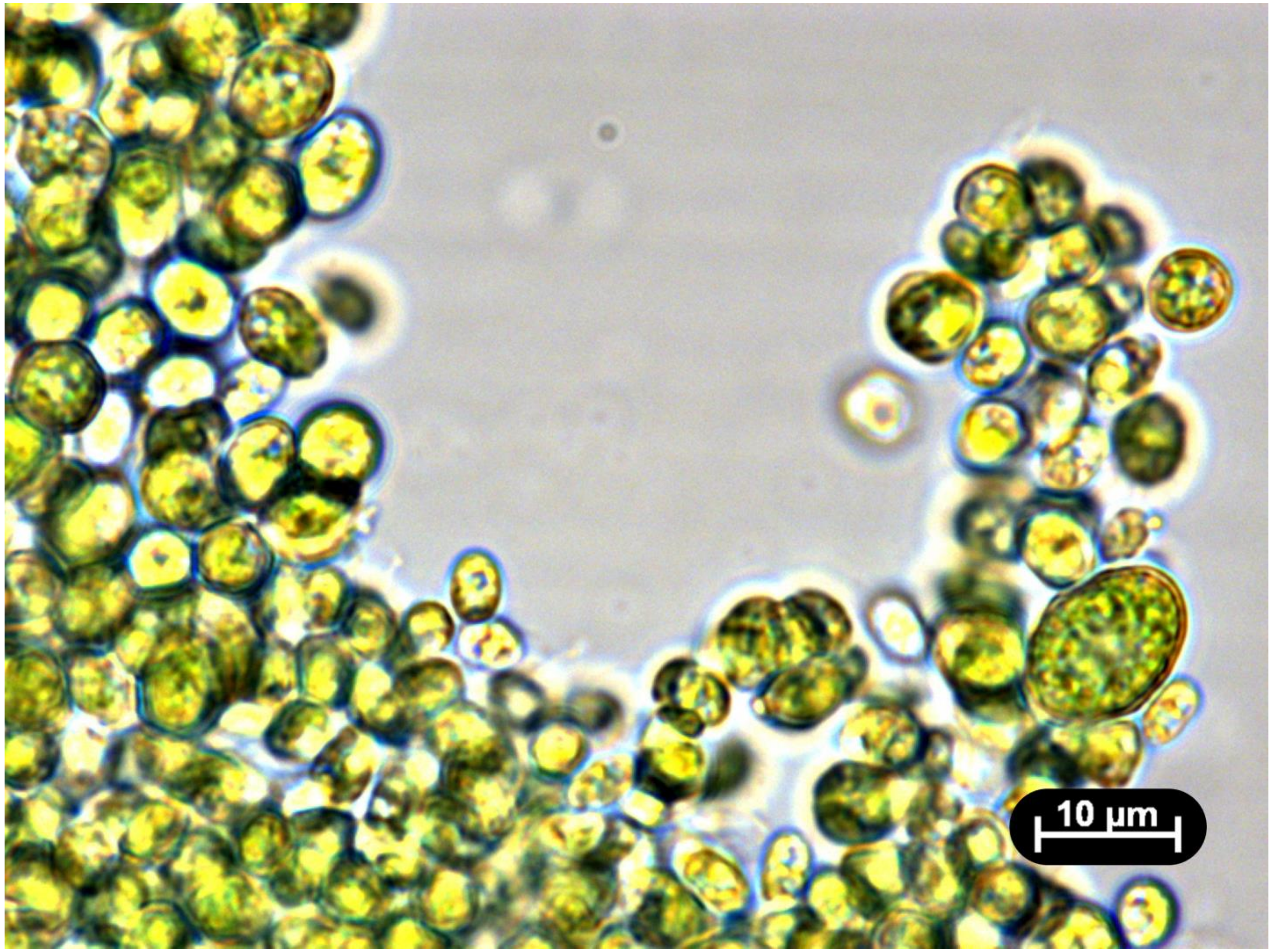

Desmodesmus abundans TCF-26g

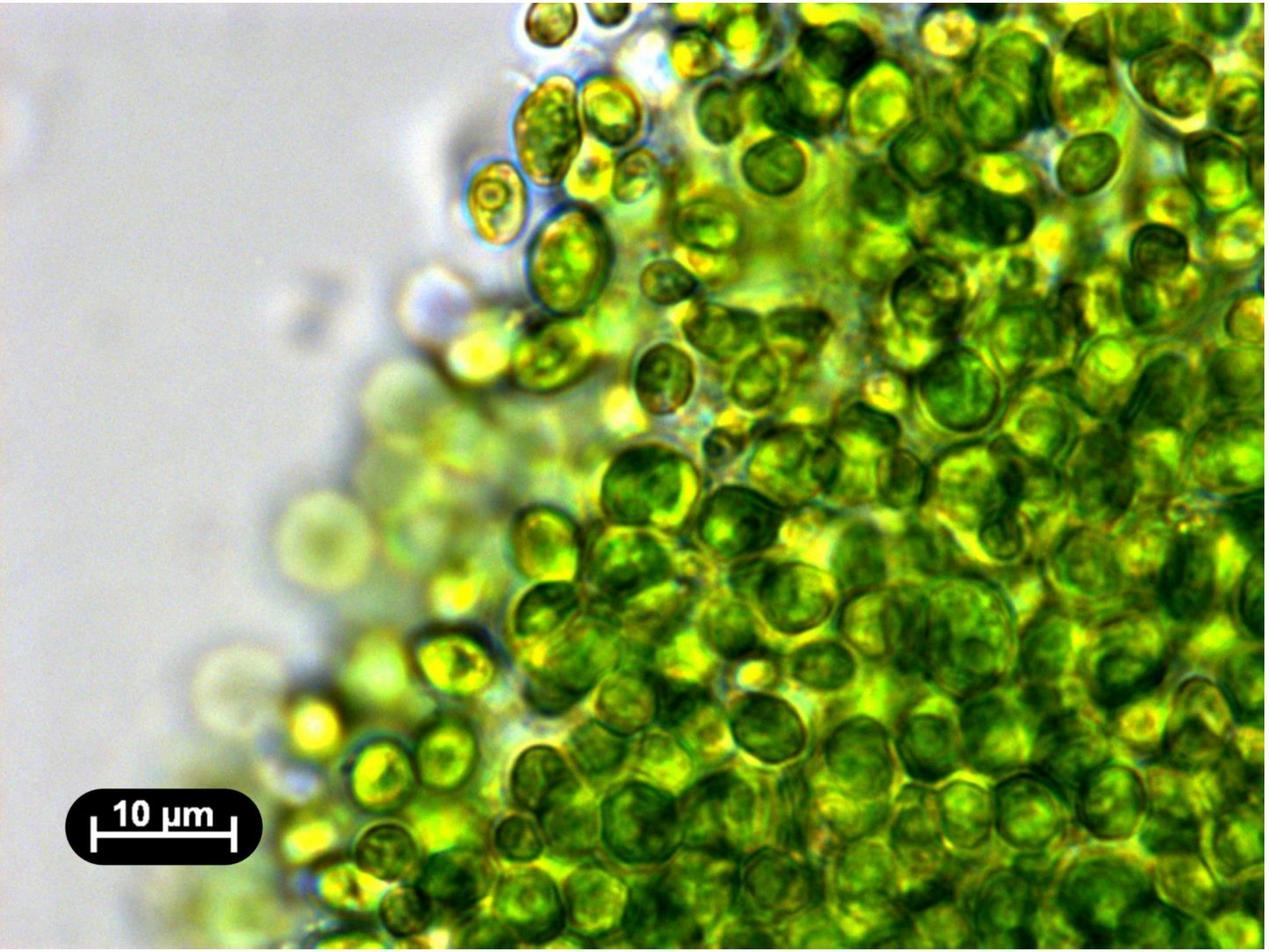

Desmodesmus abundans TCF-27g

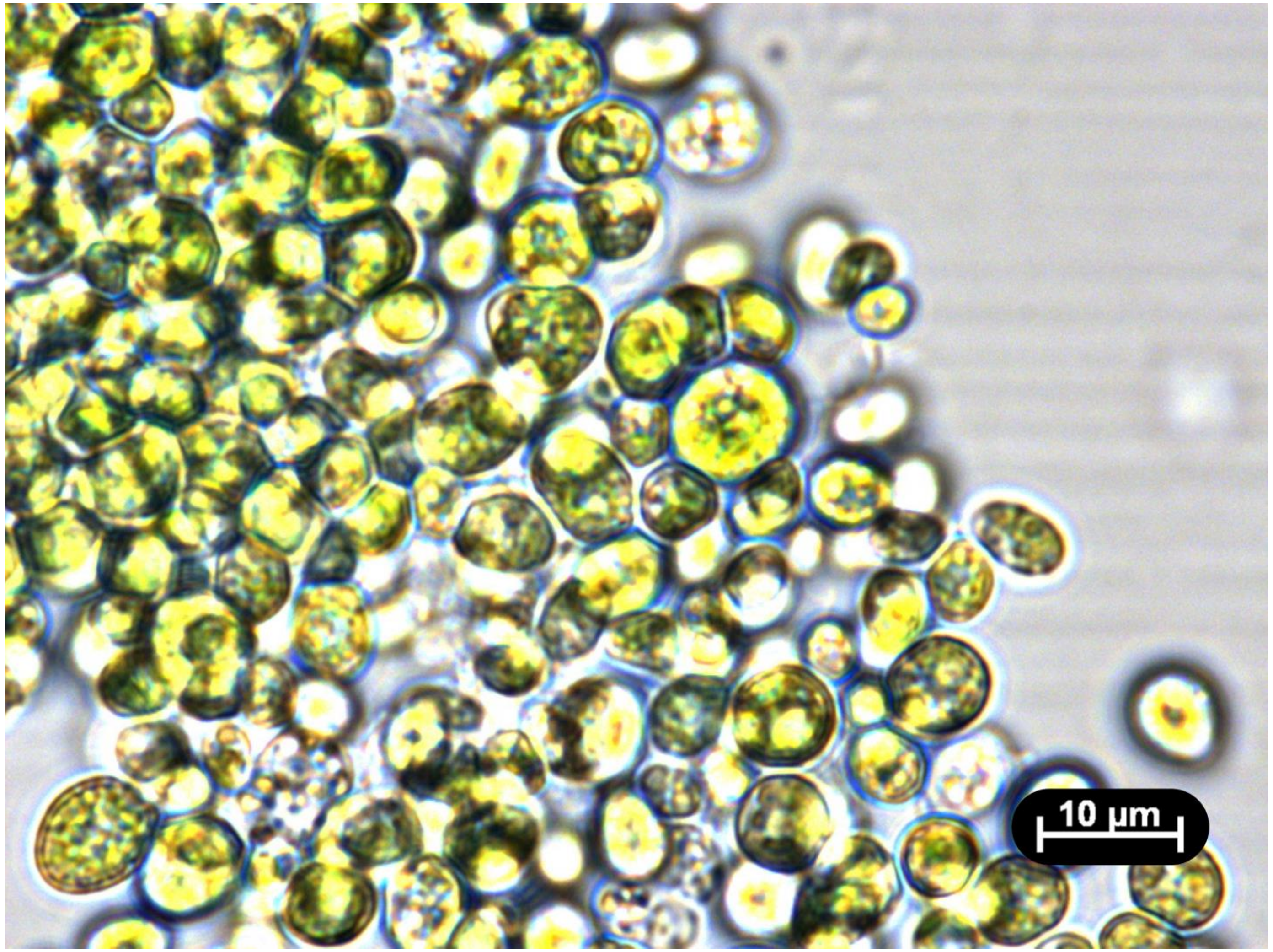

Desmodesmus sp. TCF-28g

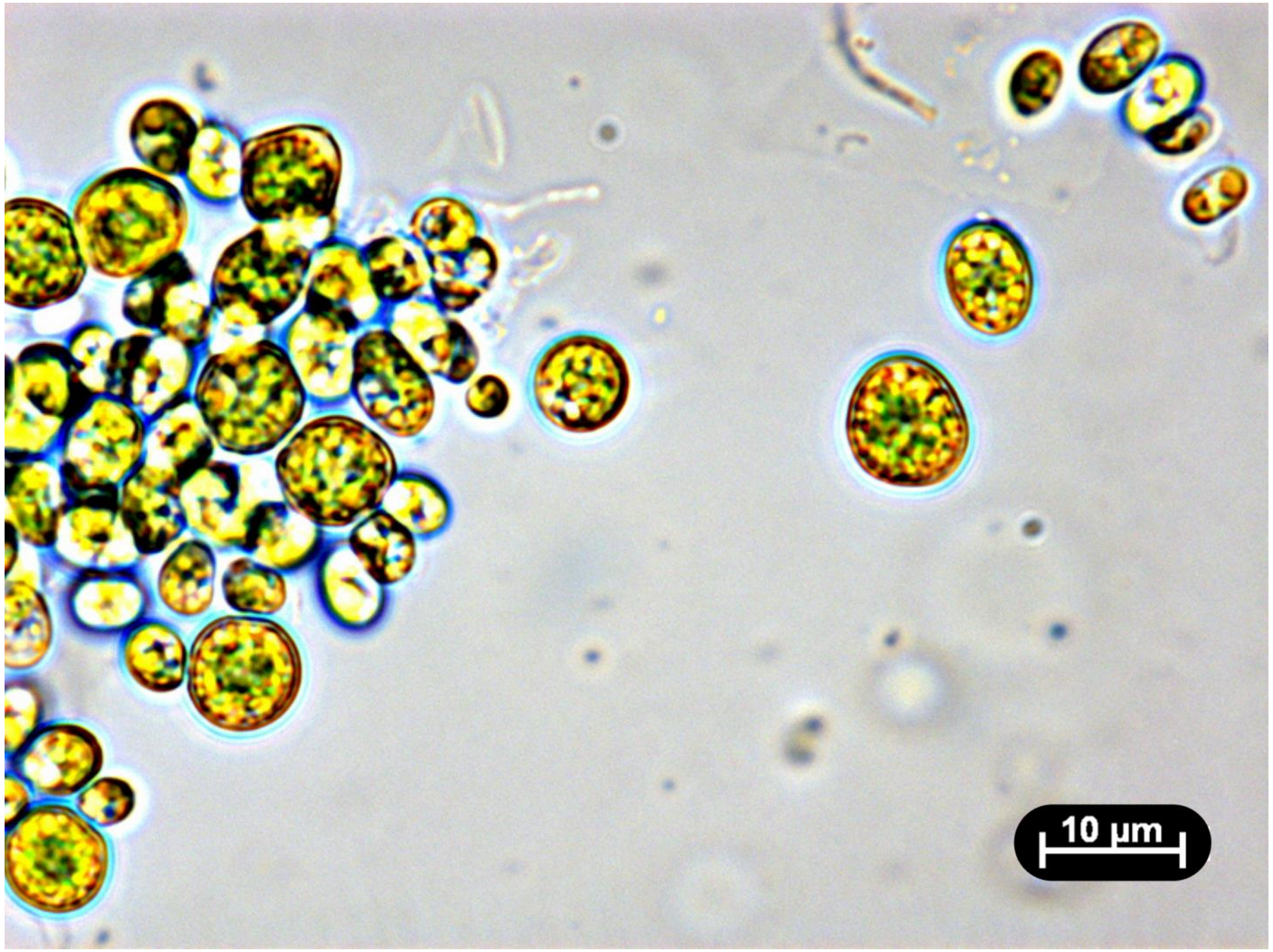

Desmodesmus sp. TCF-29g

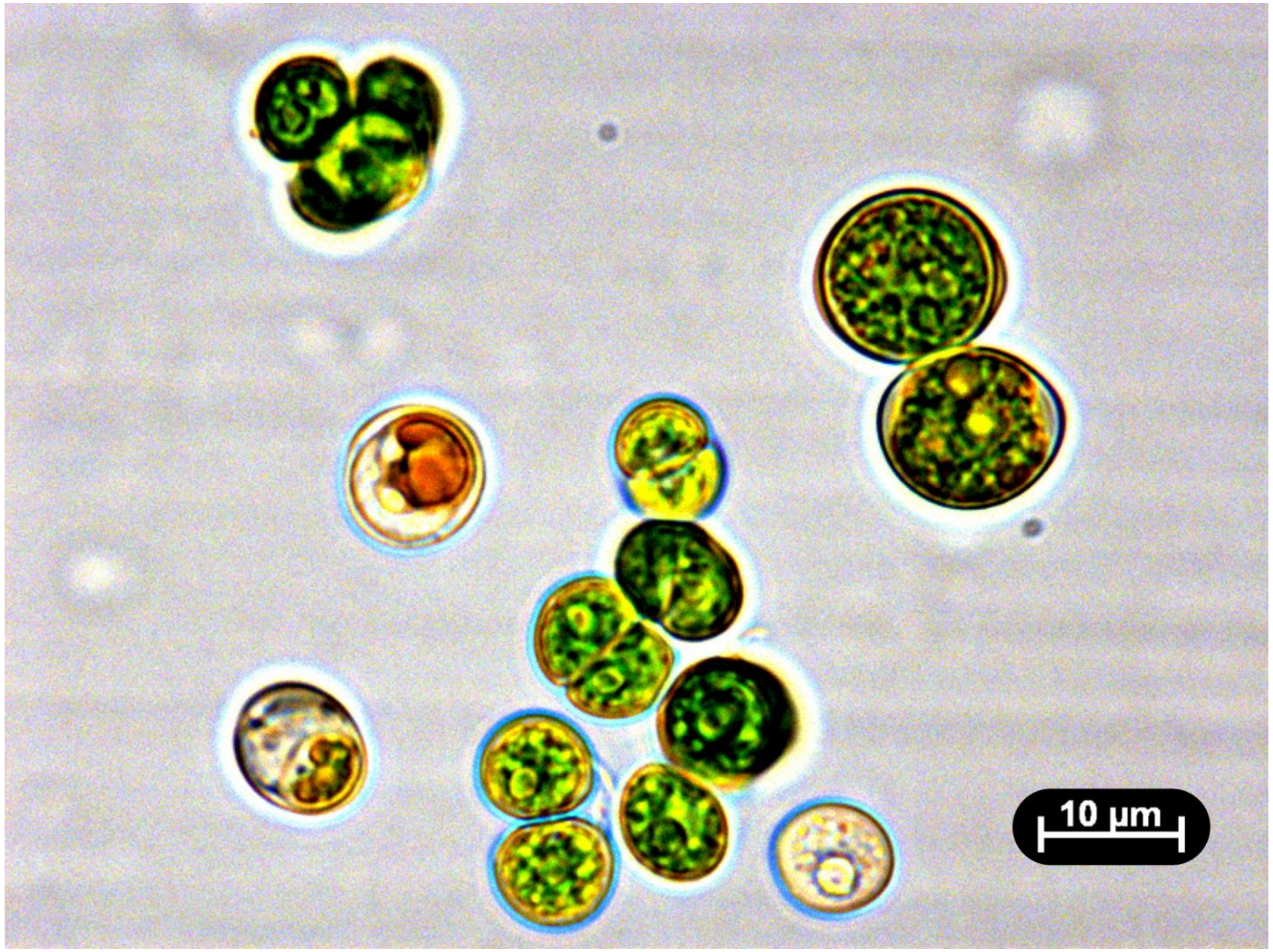

Desmodesmus sp. TCF-30g

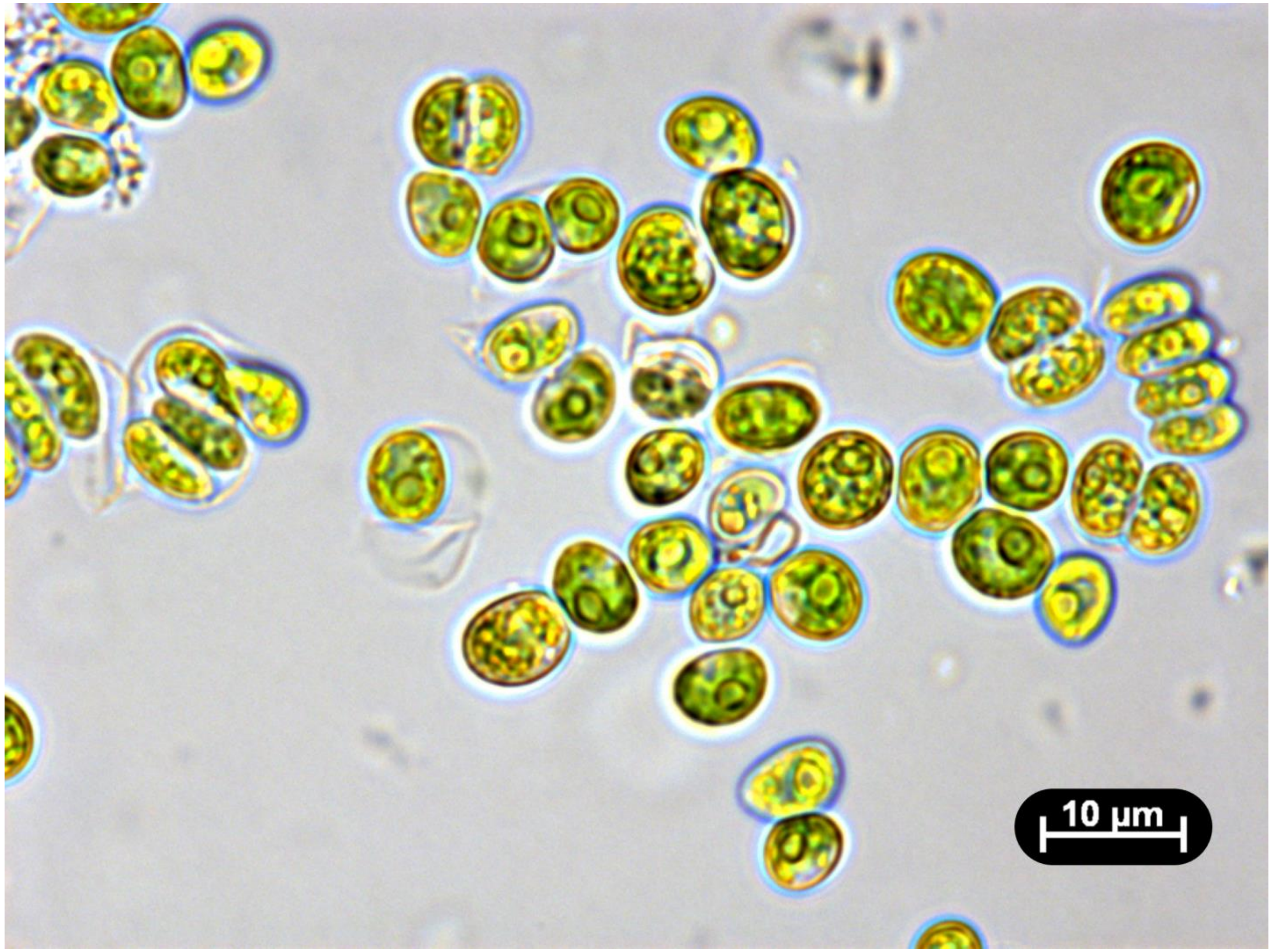

Scenedesmaceae sp. TCF-31g

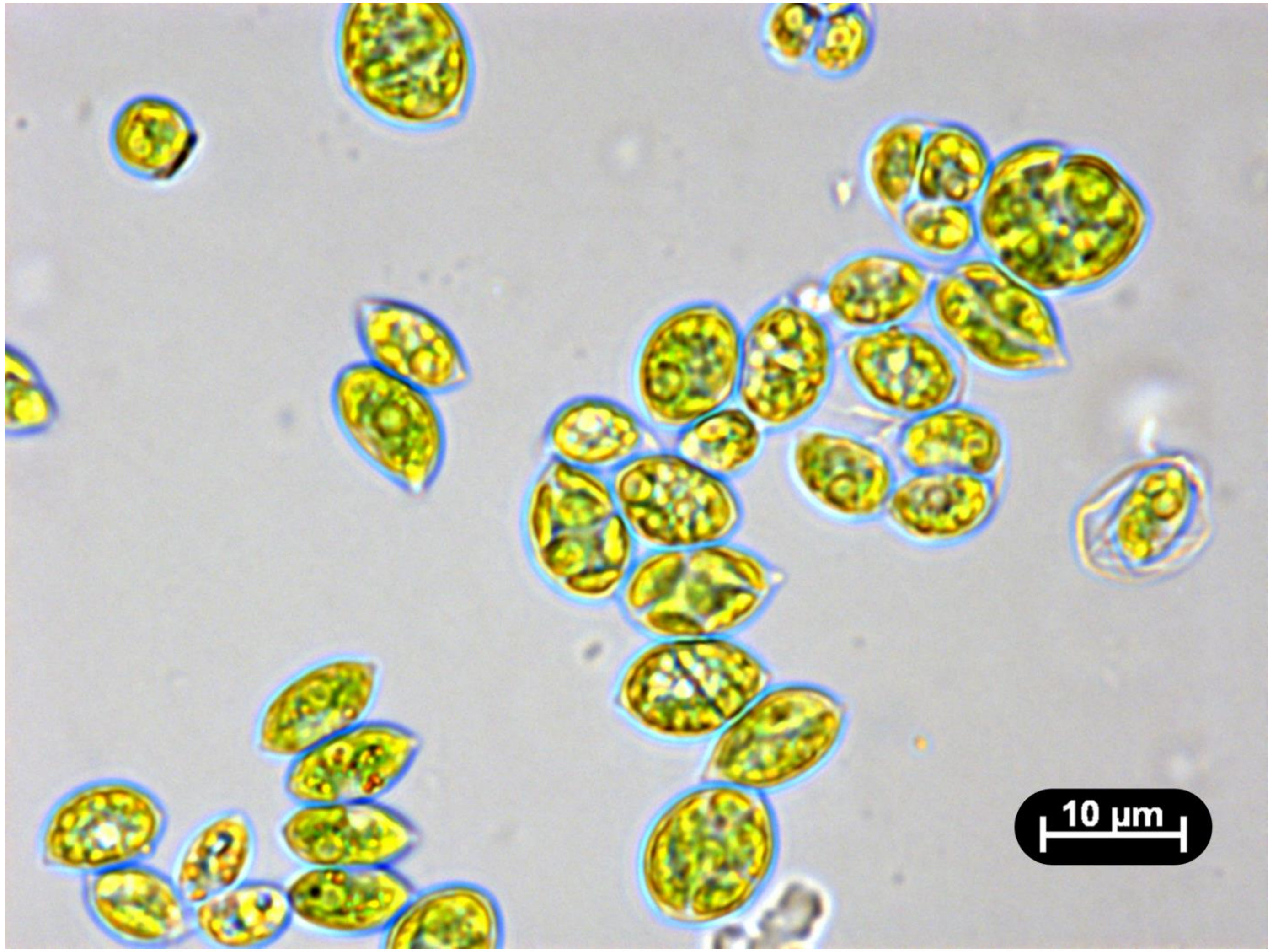

Tetradesmus obliquus TCF-32g

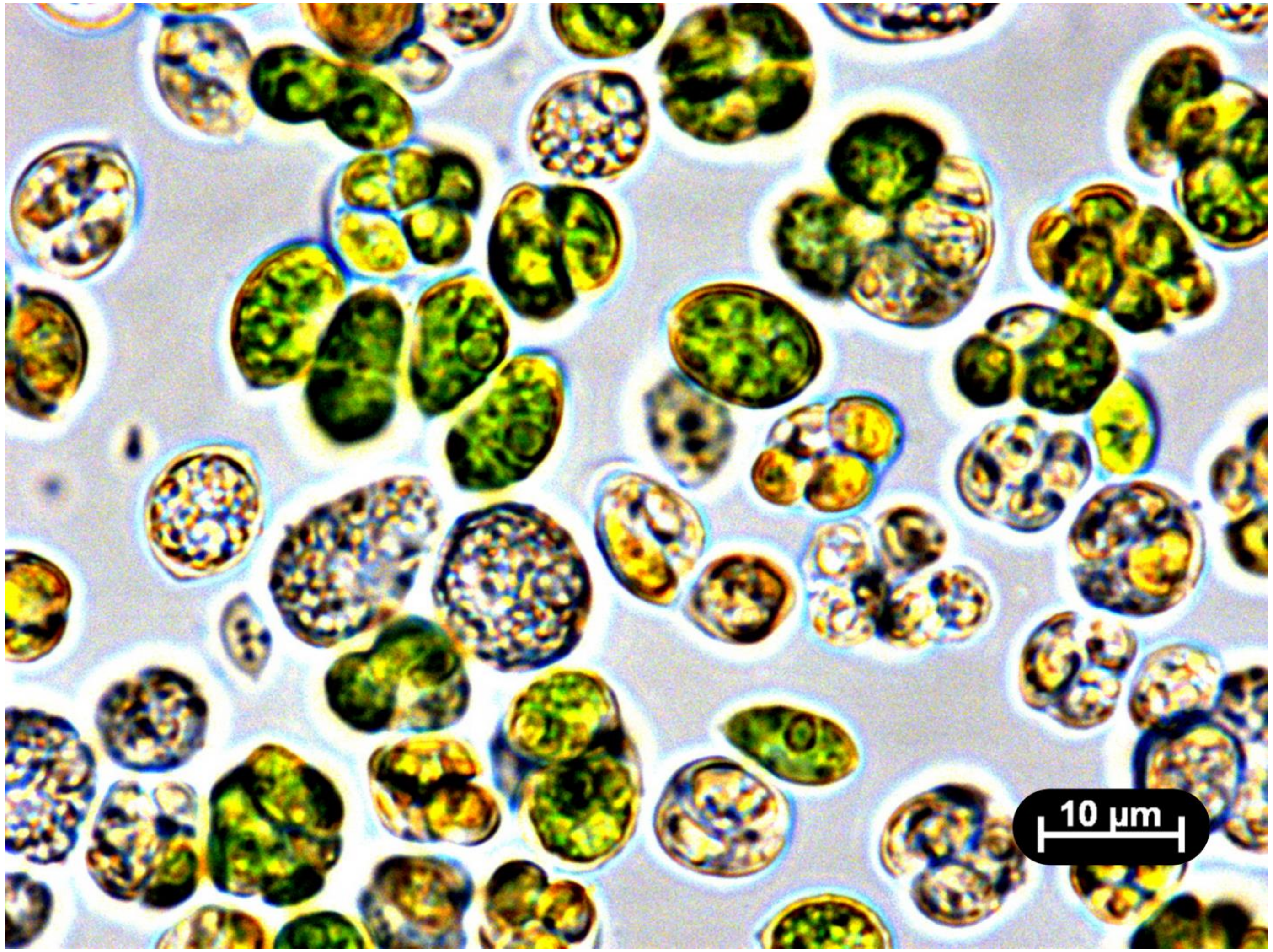

Tetradesmus sp. TCF-33g

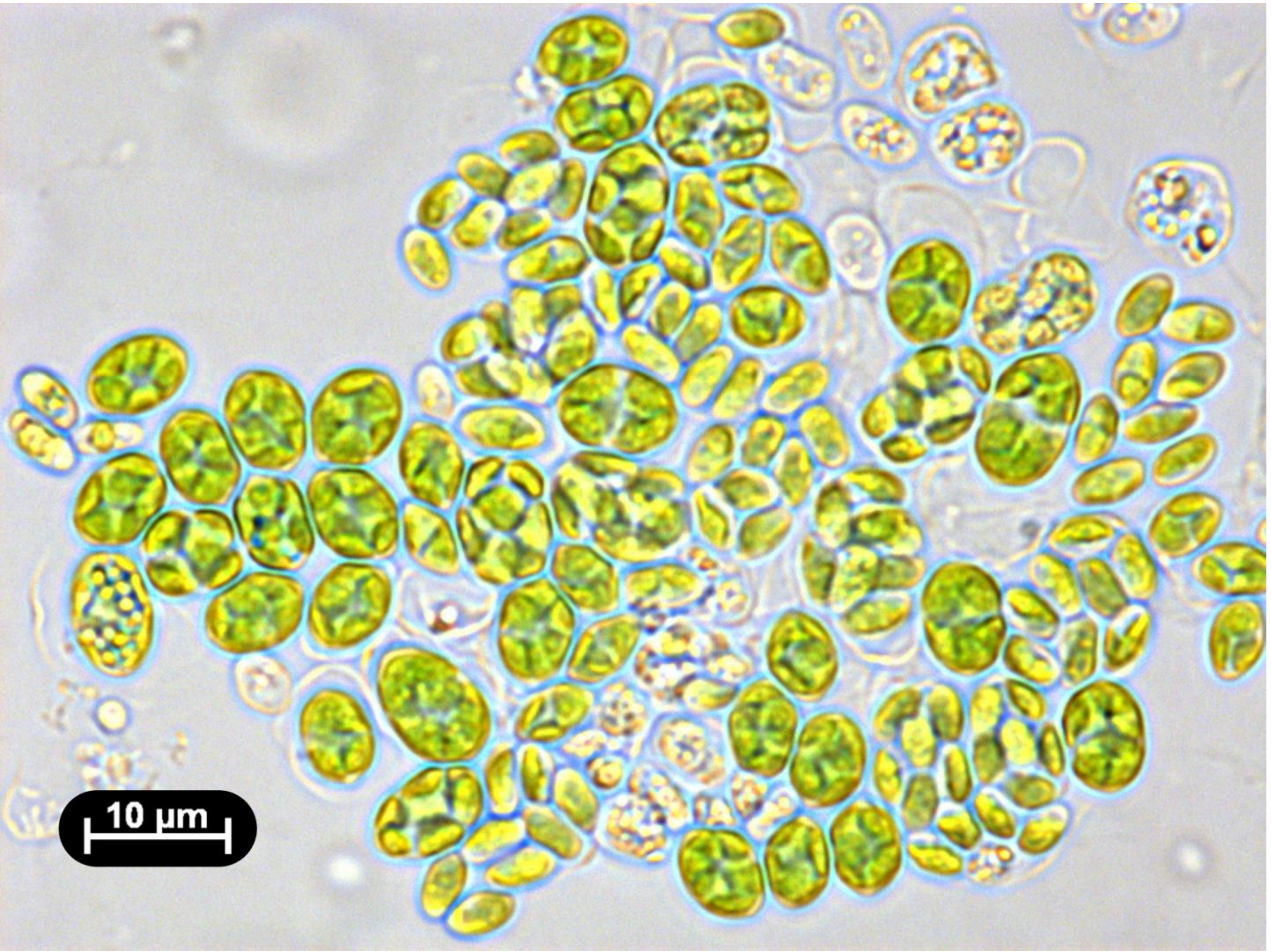

Tetradesmus sp. TCF-34g

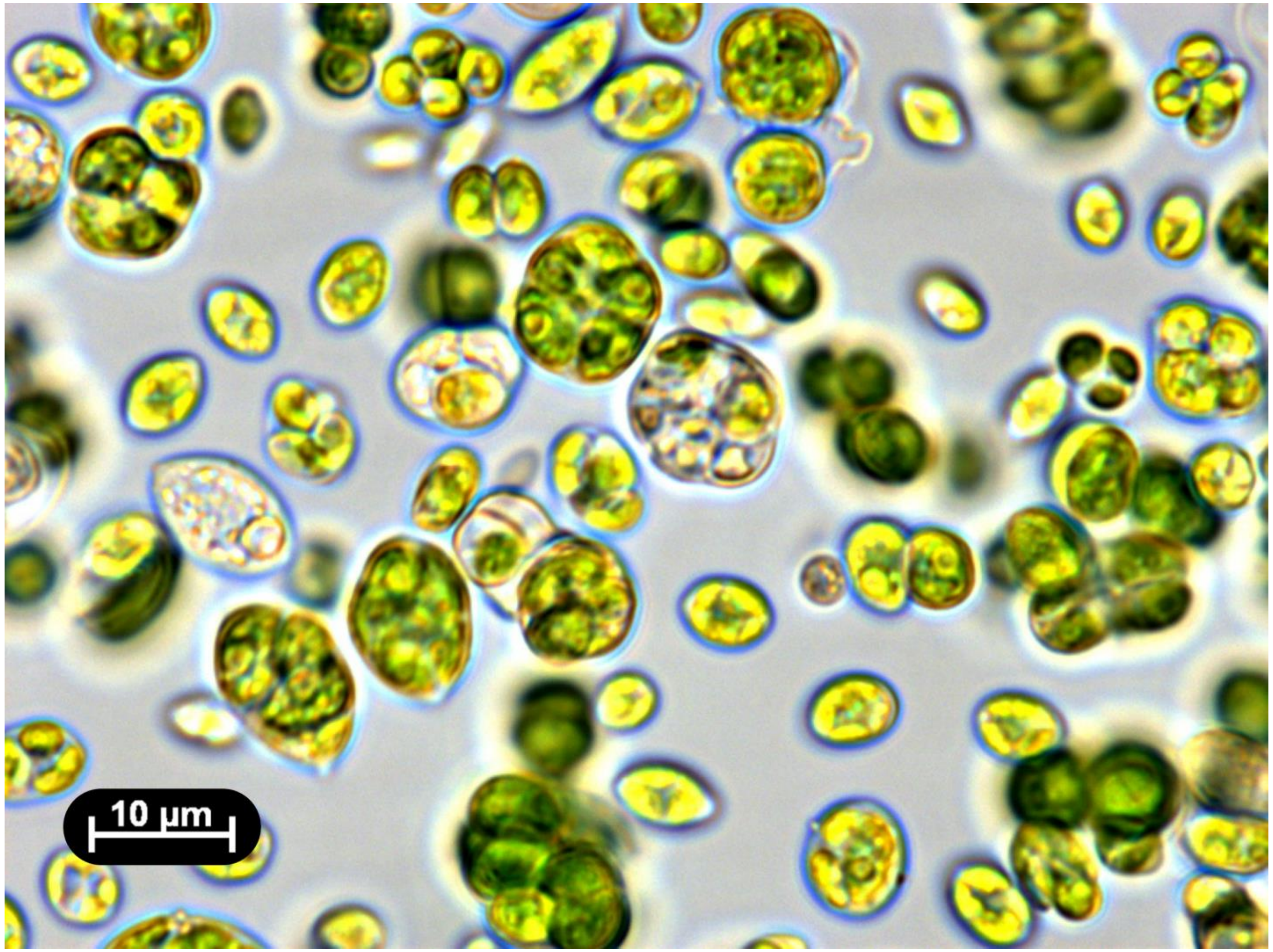

Tetradesmus sp. TCF-35g

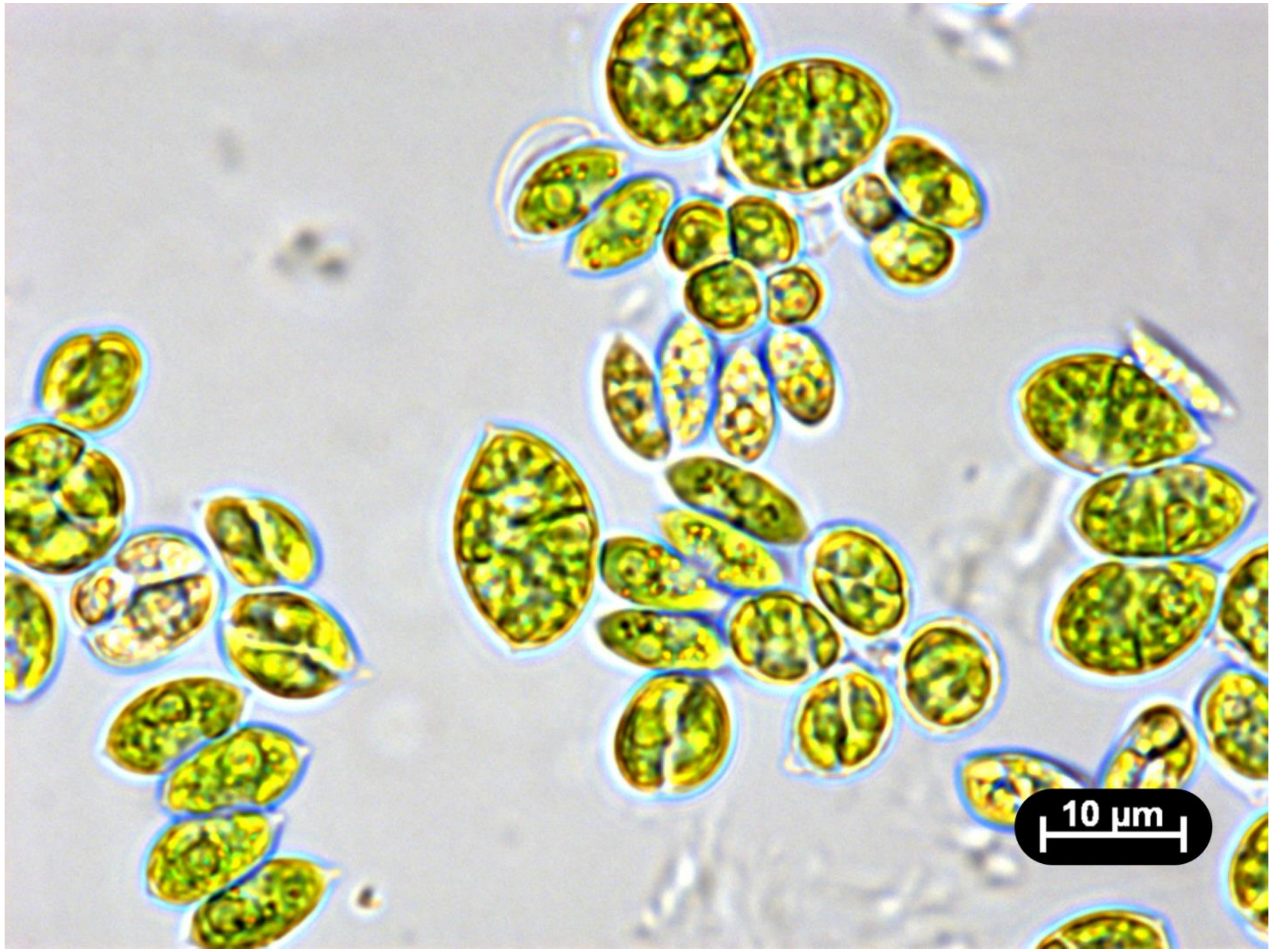

Desmodesmus abundans TCF-36g

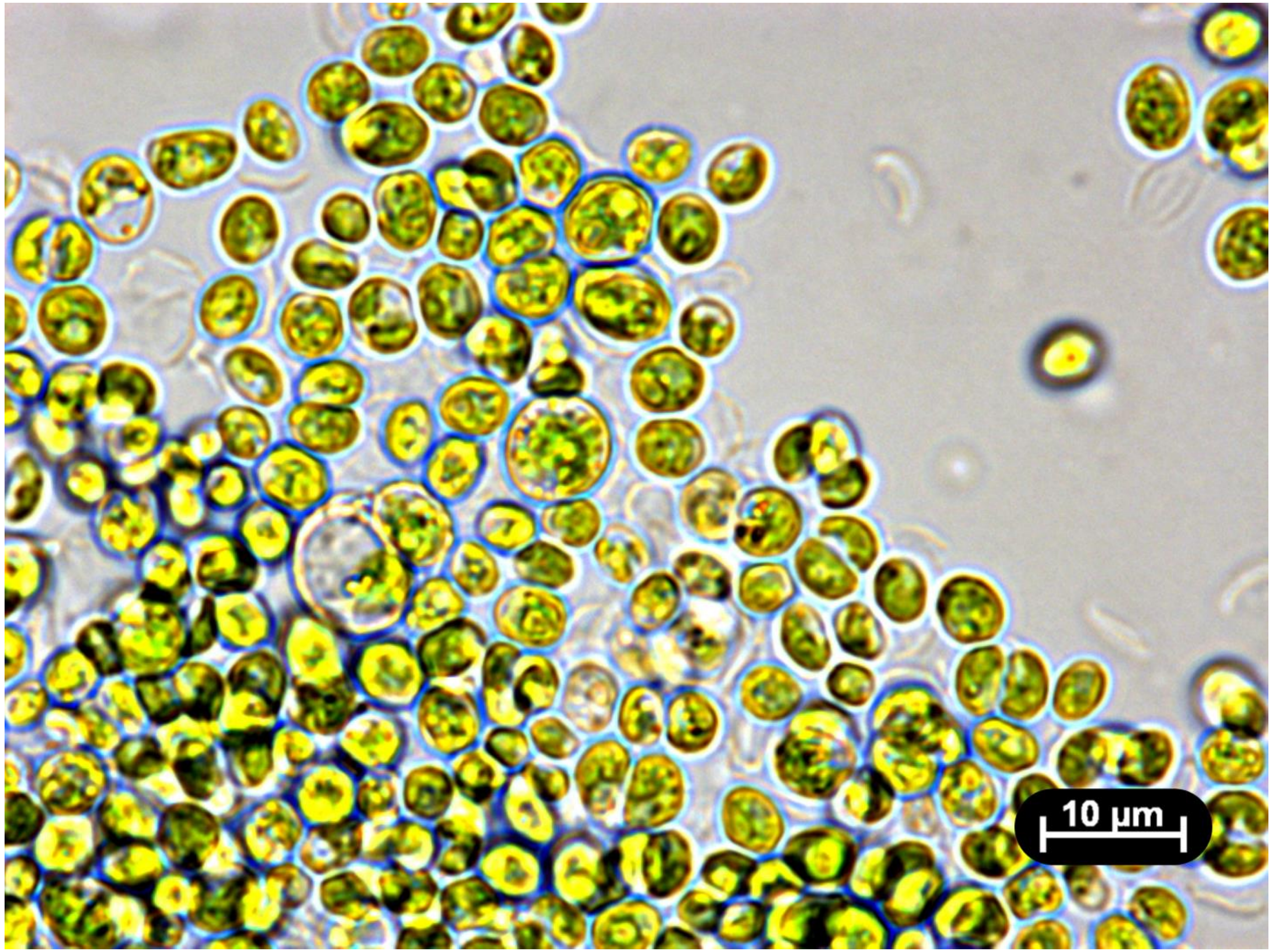

Desmodesmus armatus TCF-37g

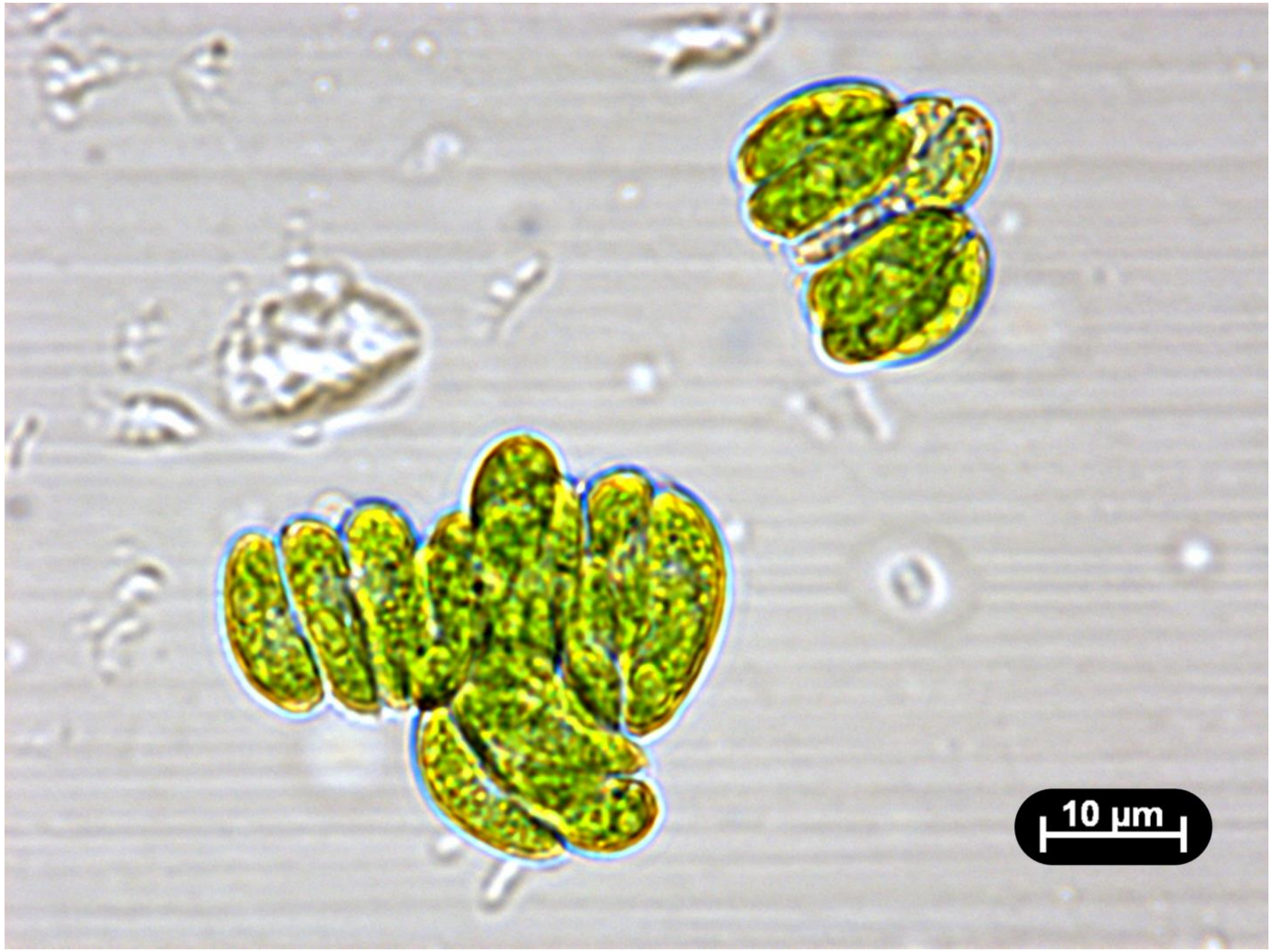

Desmodesmus sp. TCF-38g

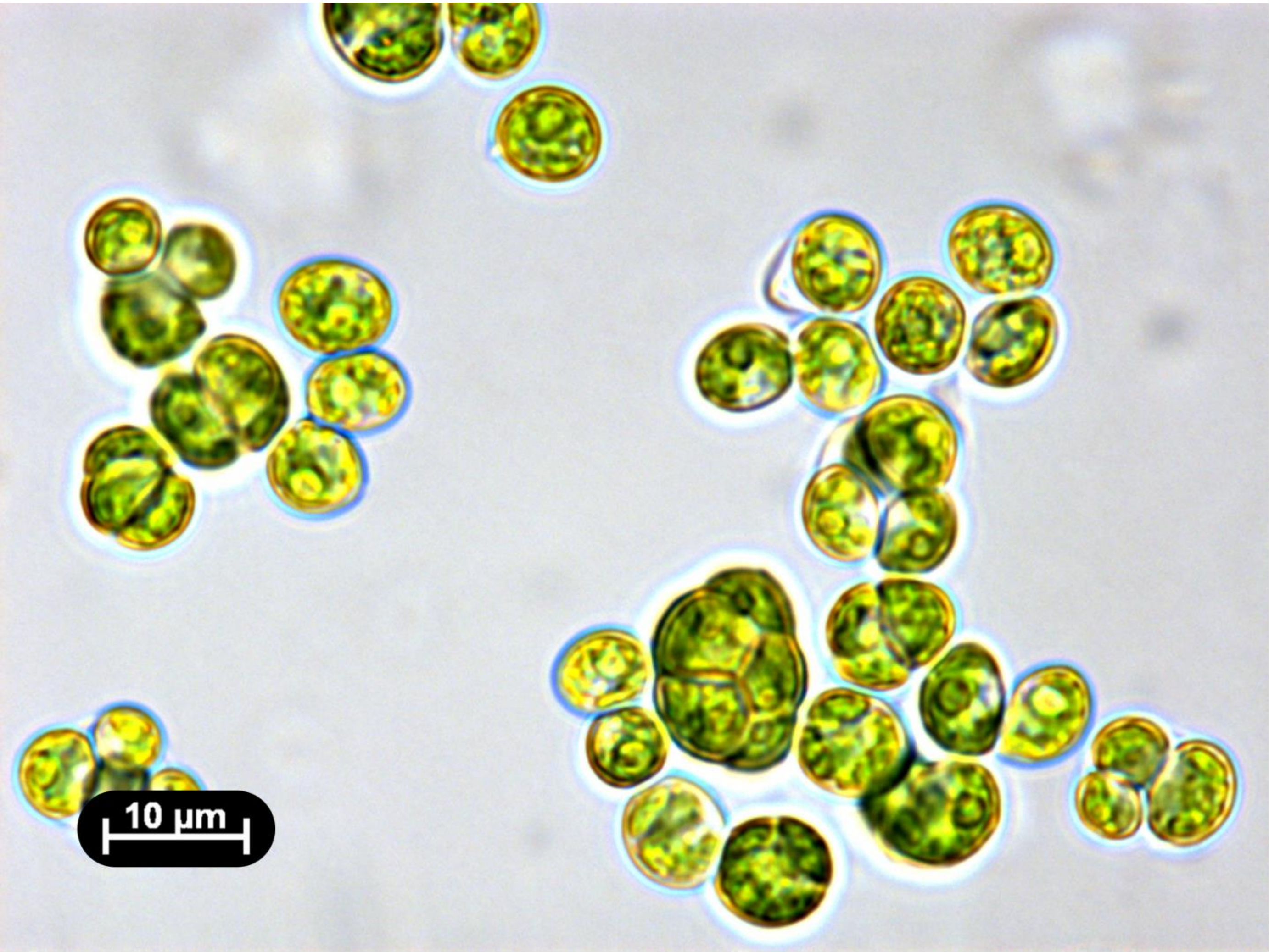

Tetradesmus sp. TCF-39g

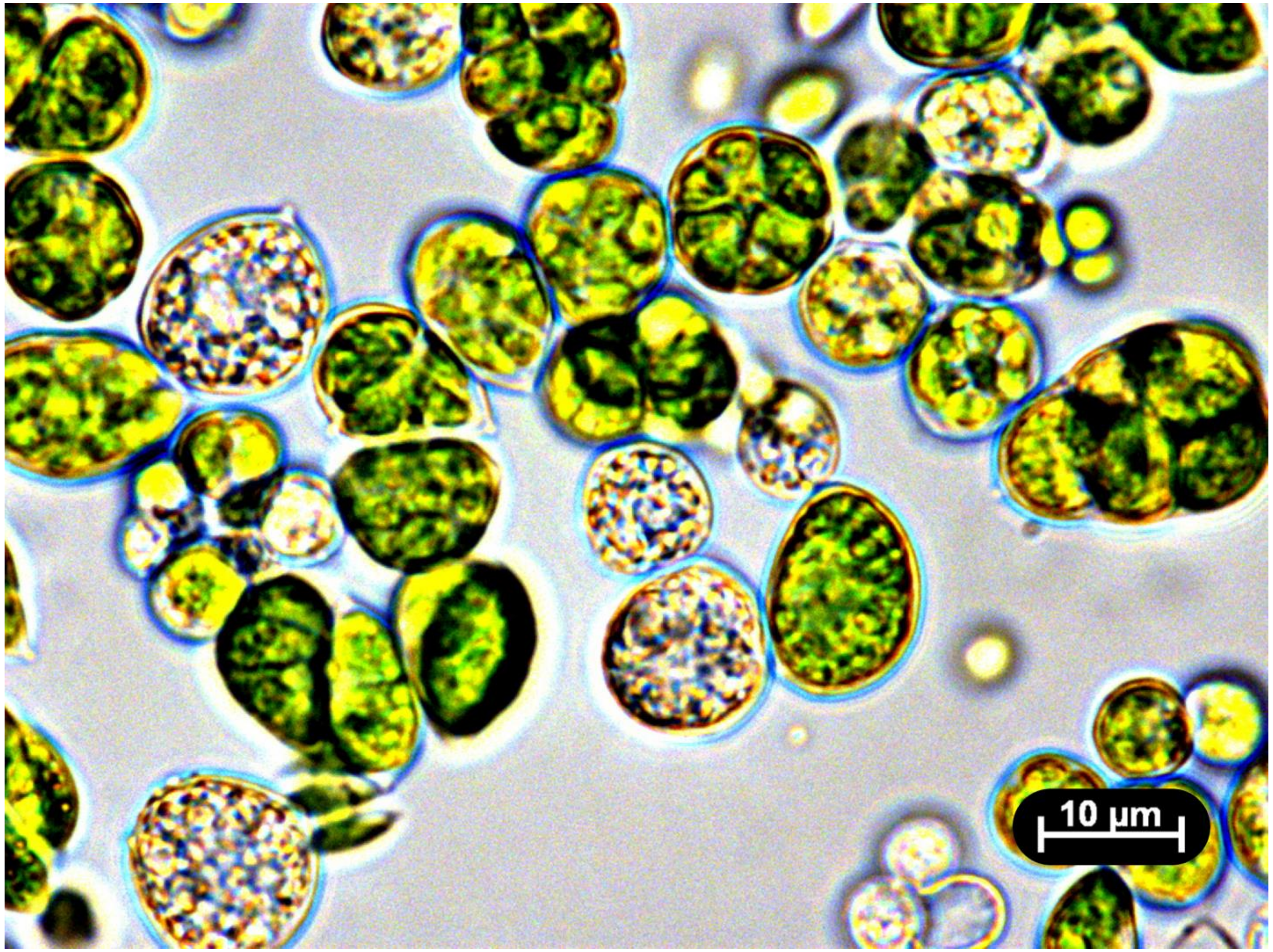

Chlamydomonas pulvinata TCF-40g

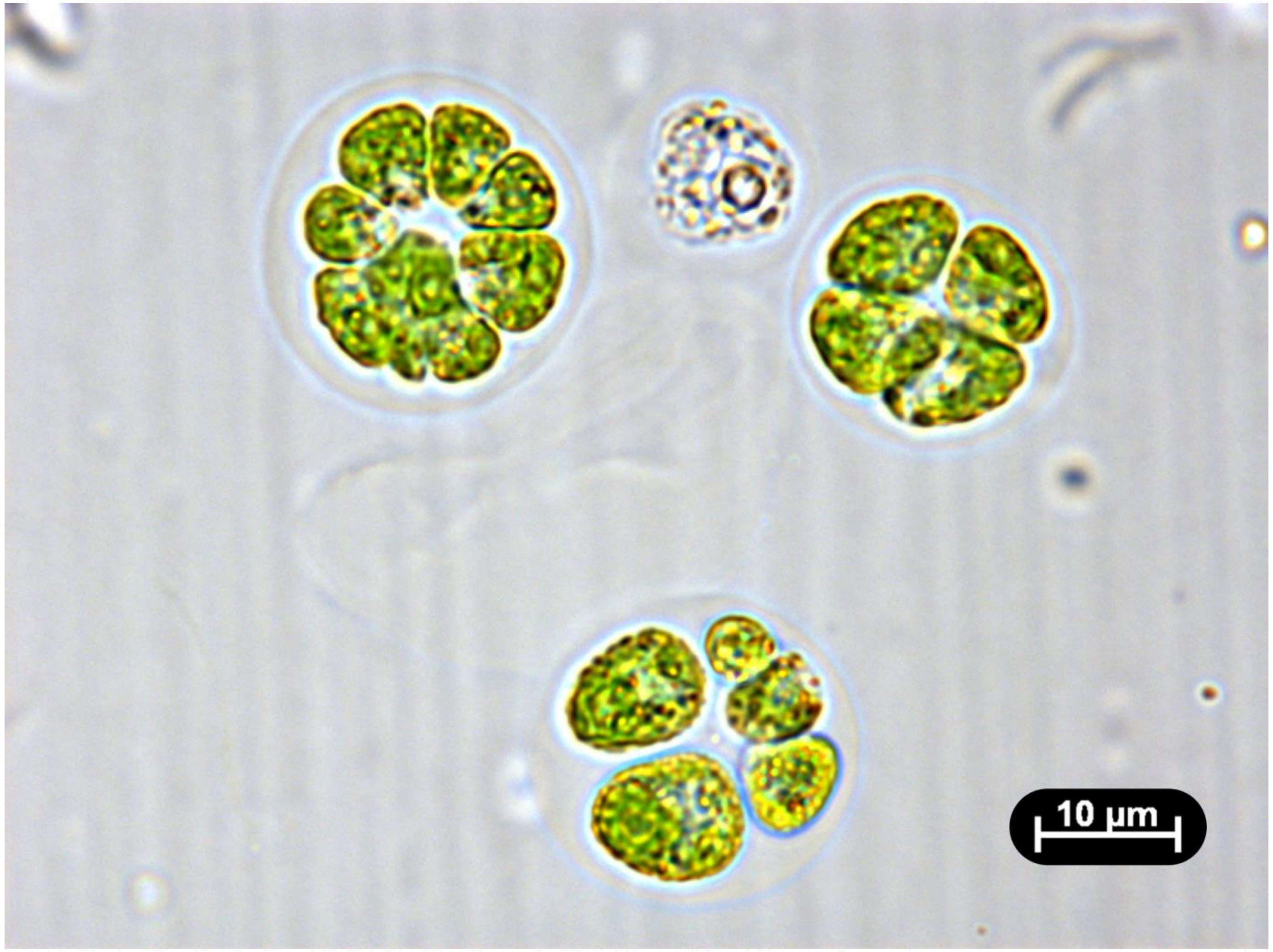

Chlamydomonas pulvinata TCF-41g

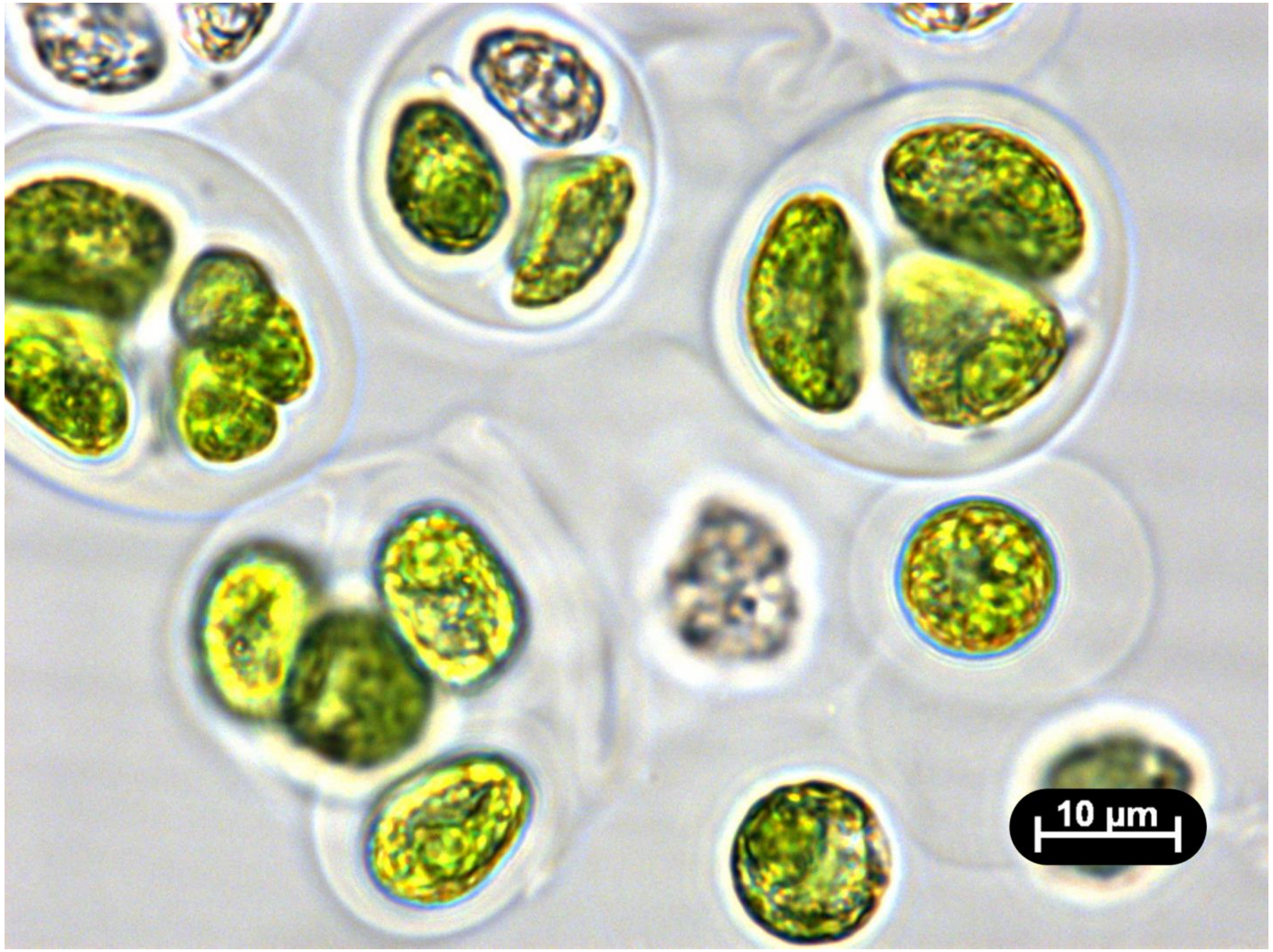

Desmodesmus armatus TCF-42g

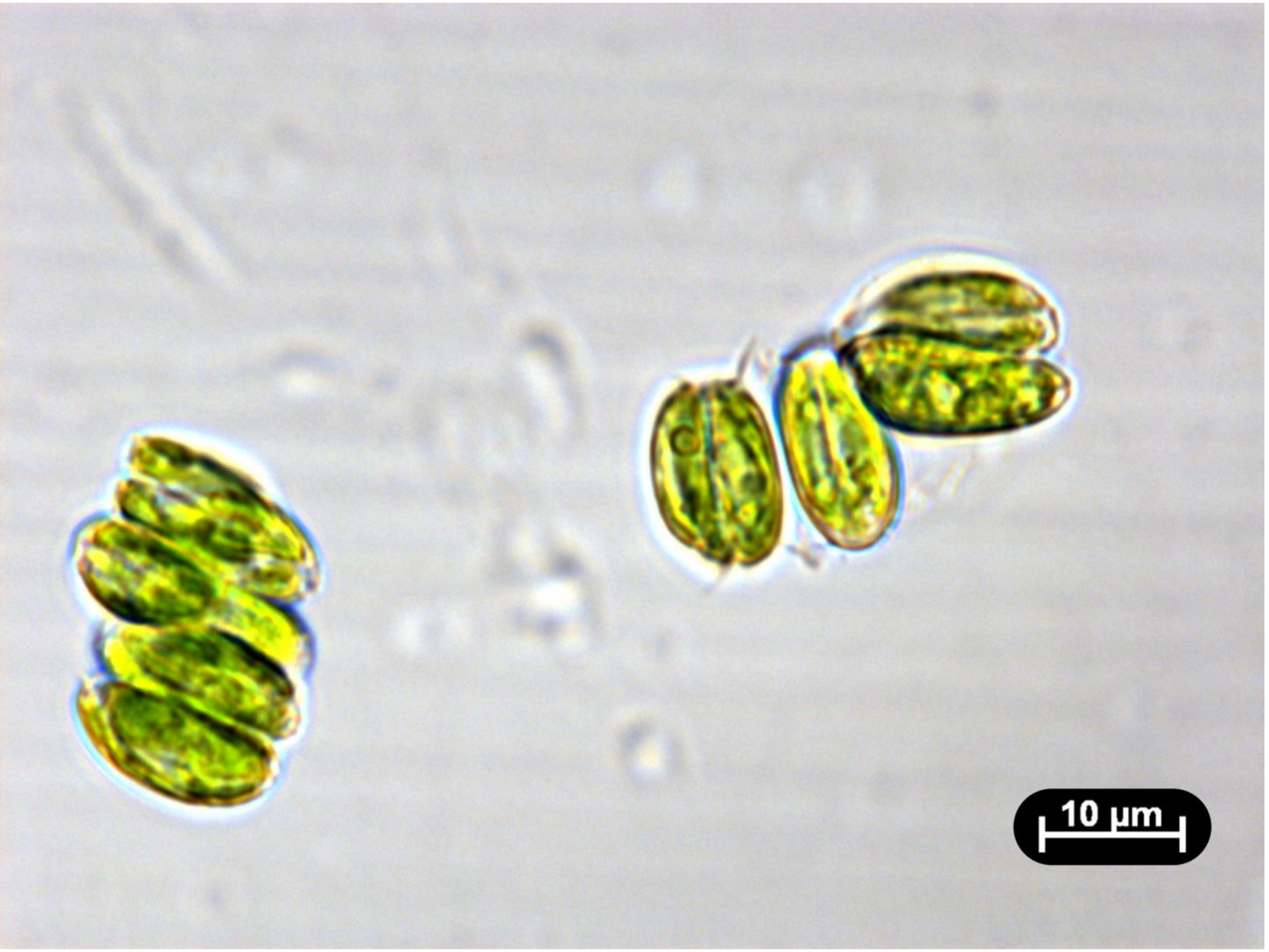

Desmodesmus sp. TCF-43g

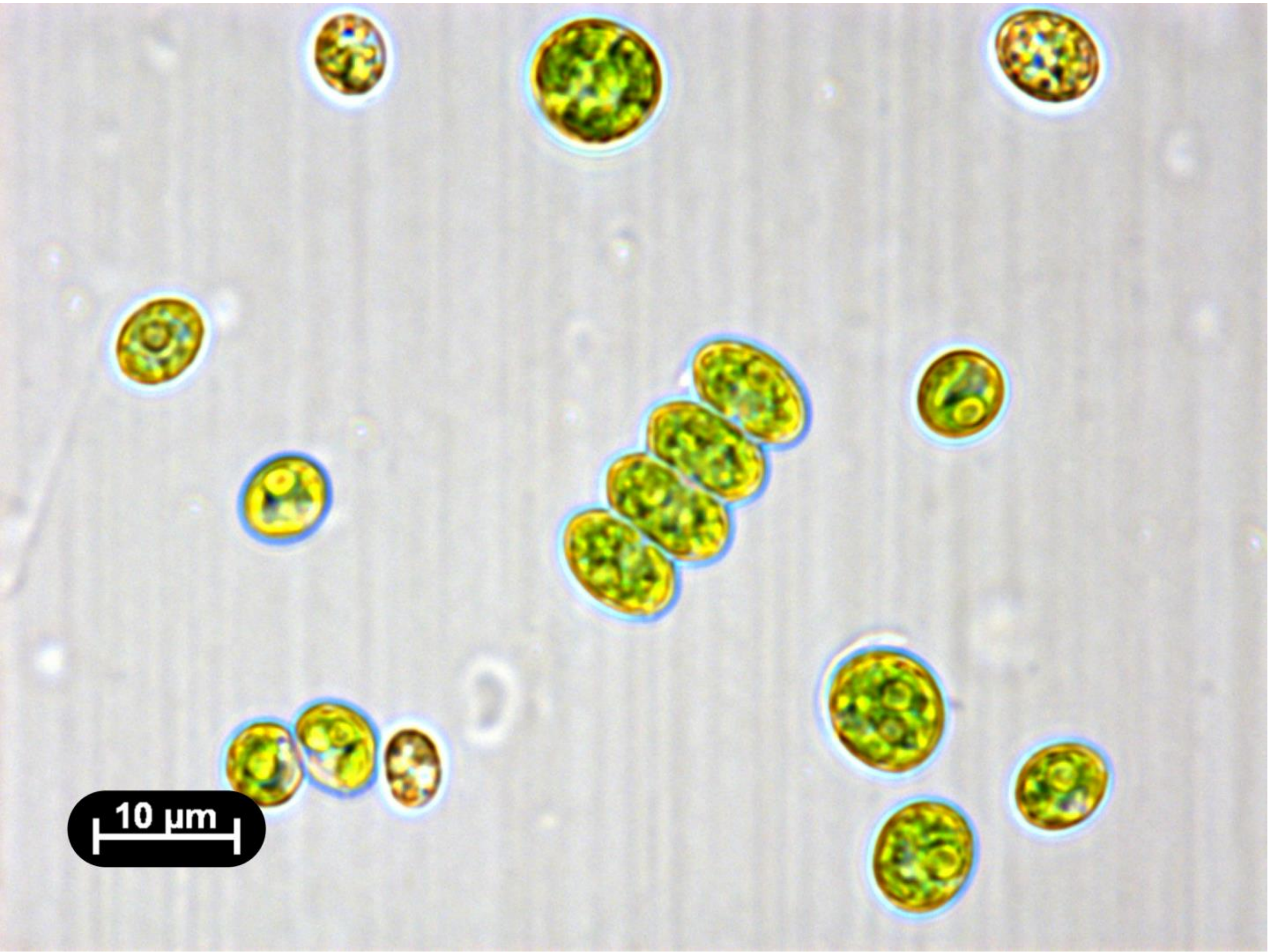

Scenedesmus sp. TCF-44g

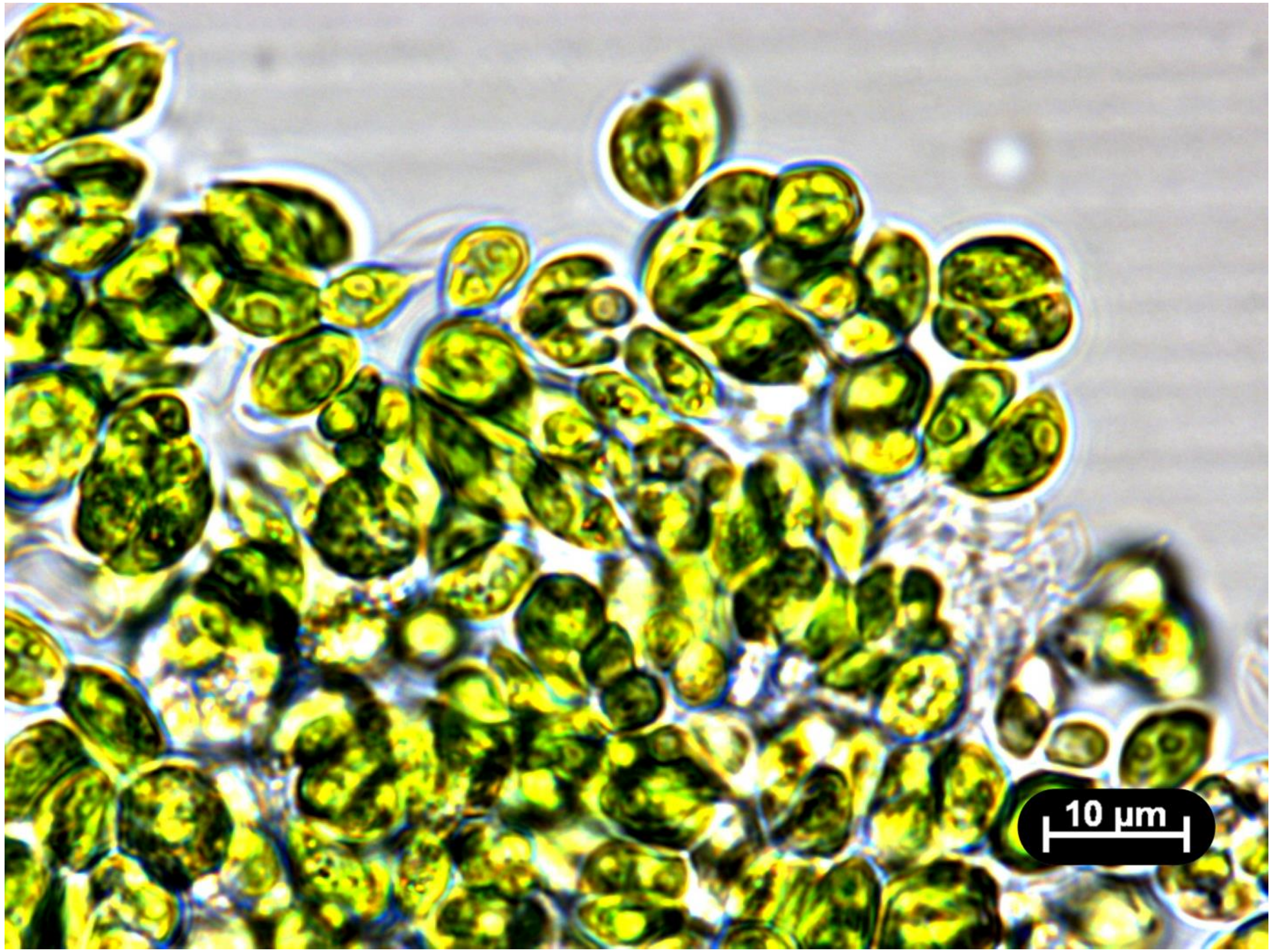

Tetradesmus dimorphus TCF-45g

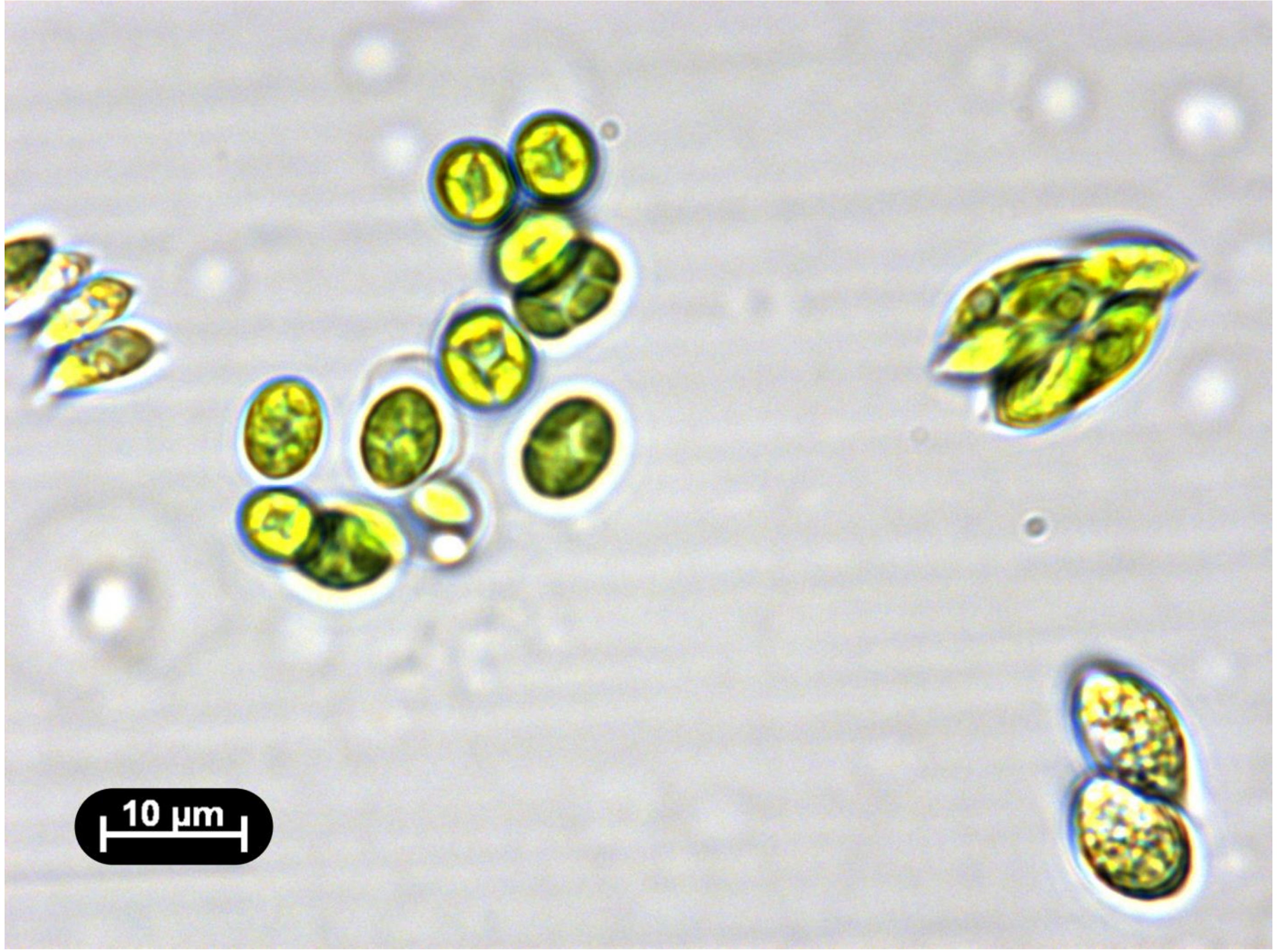

Chlamydomonas pulvinata TCF-46g

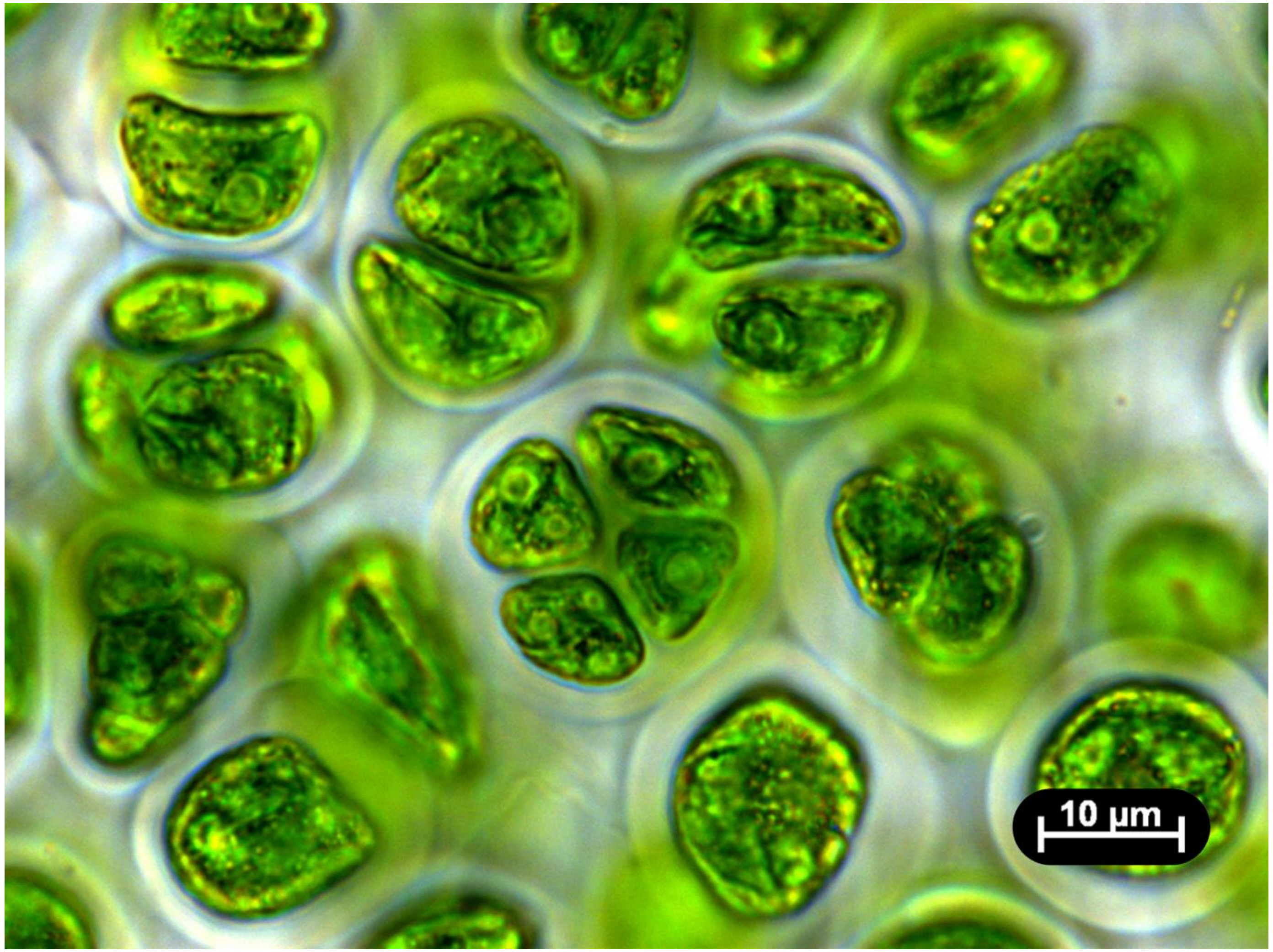

Chlamydomonas pulvinata TCF-47g

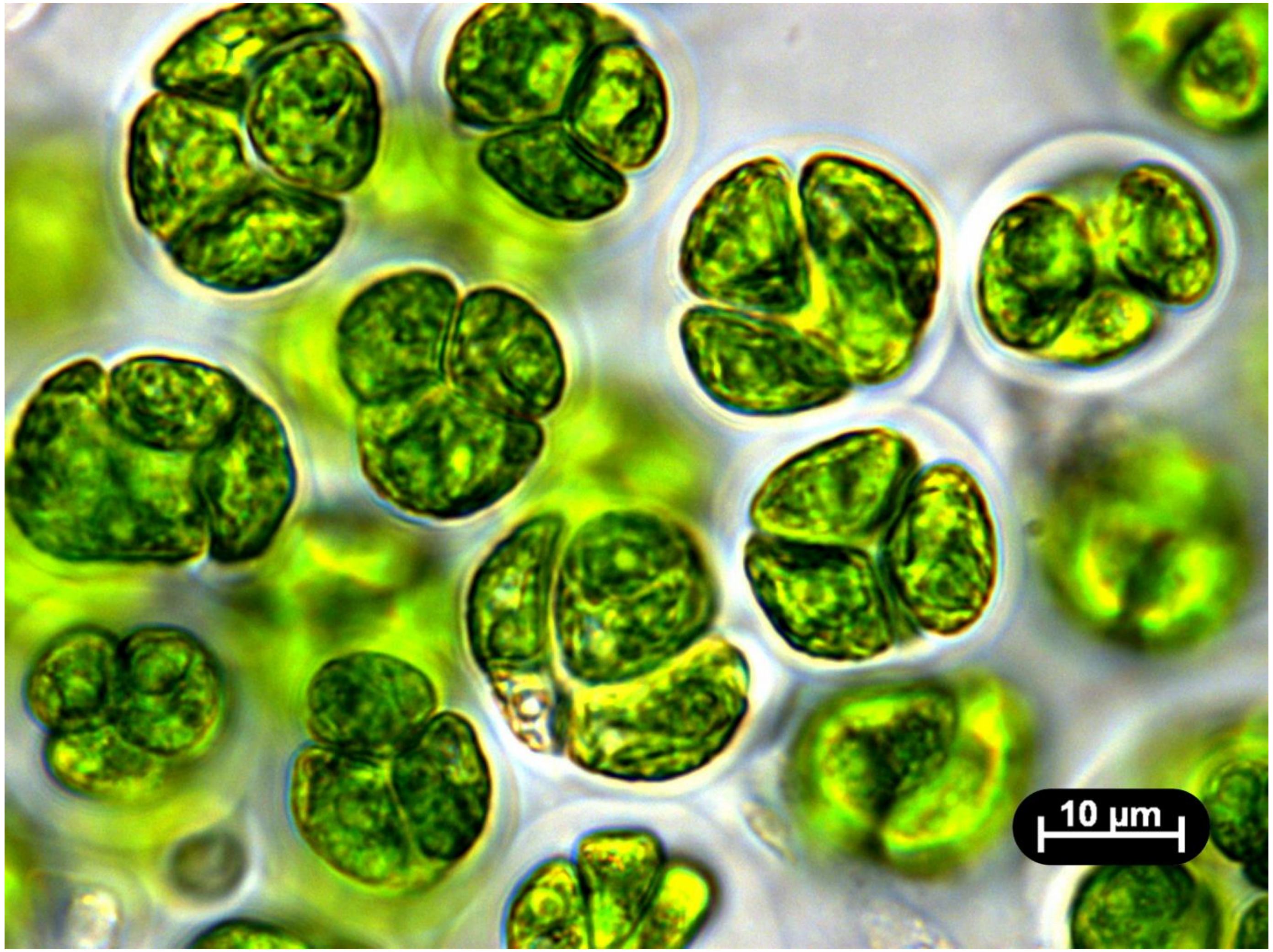

Chlamydomonas pulvinata TCF-48g

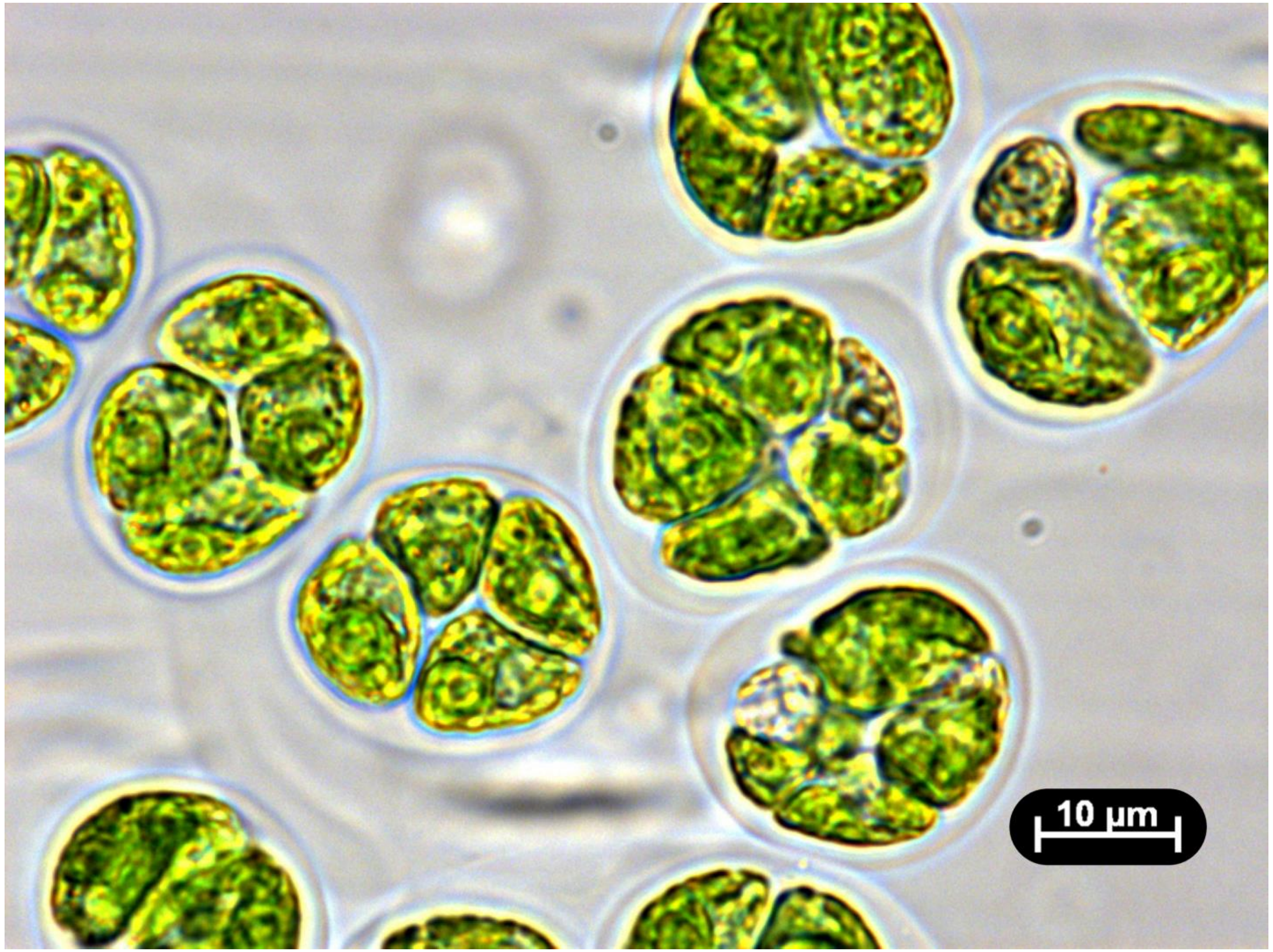

Chlorella sorokiniana TCF-49g

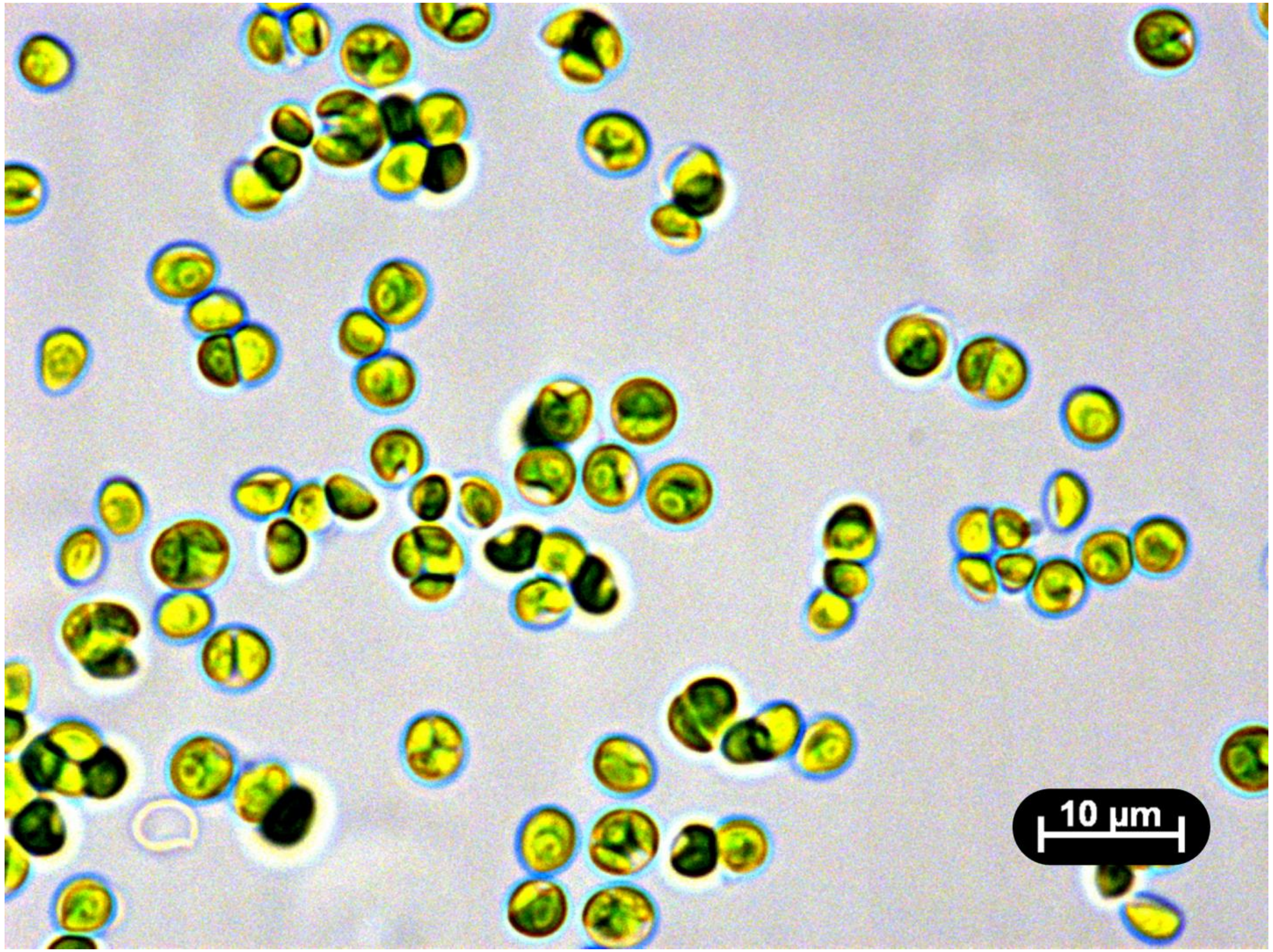

Desmodesmus abundans TCF-50g

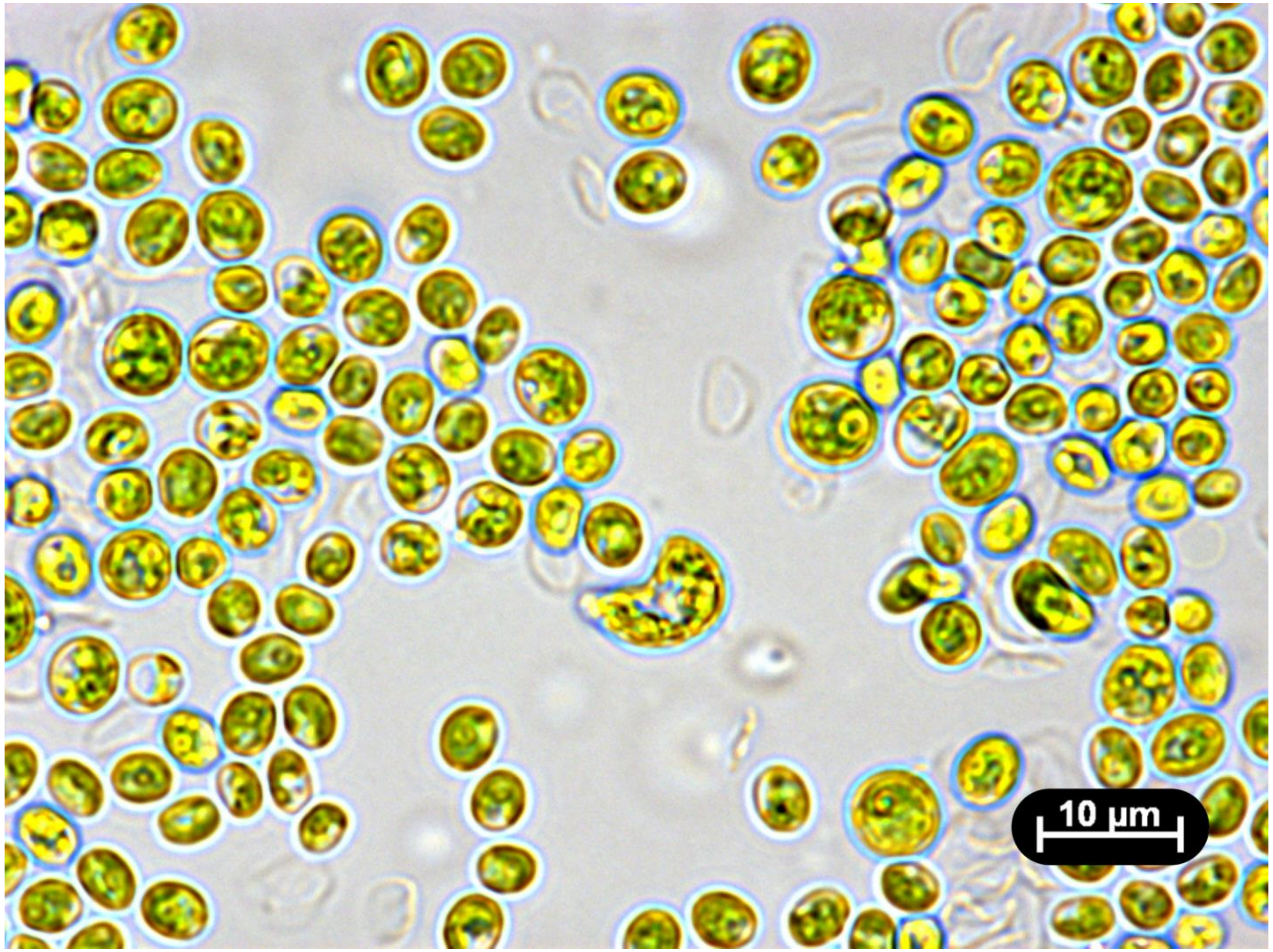

Desmodesmus abundans TCF-51g

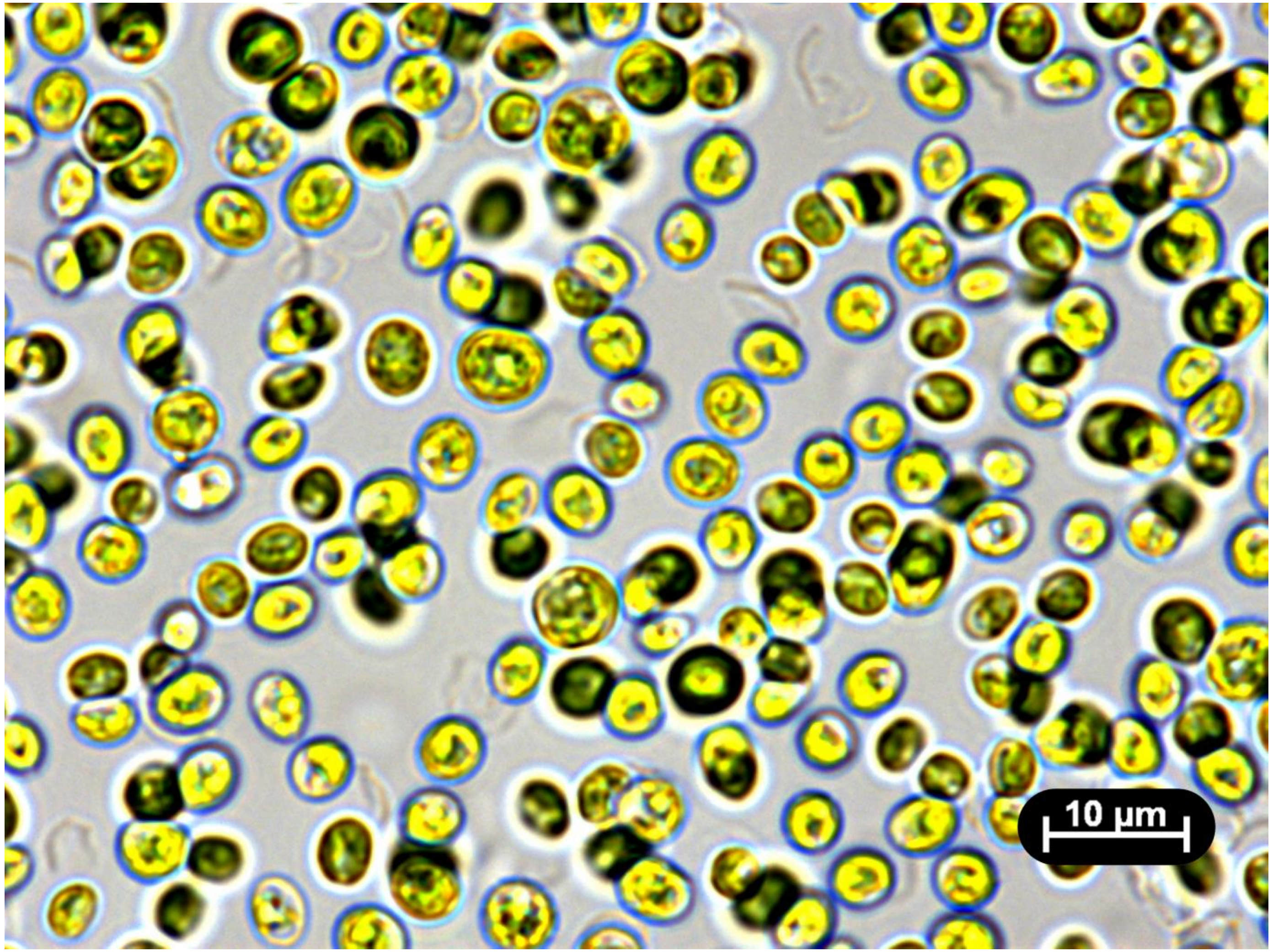

Desmodesmus sp. TCF-52g

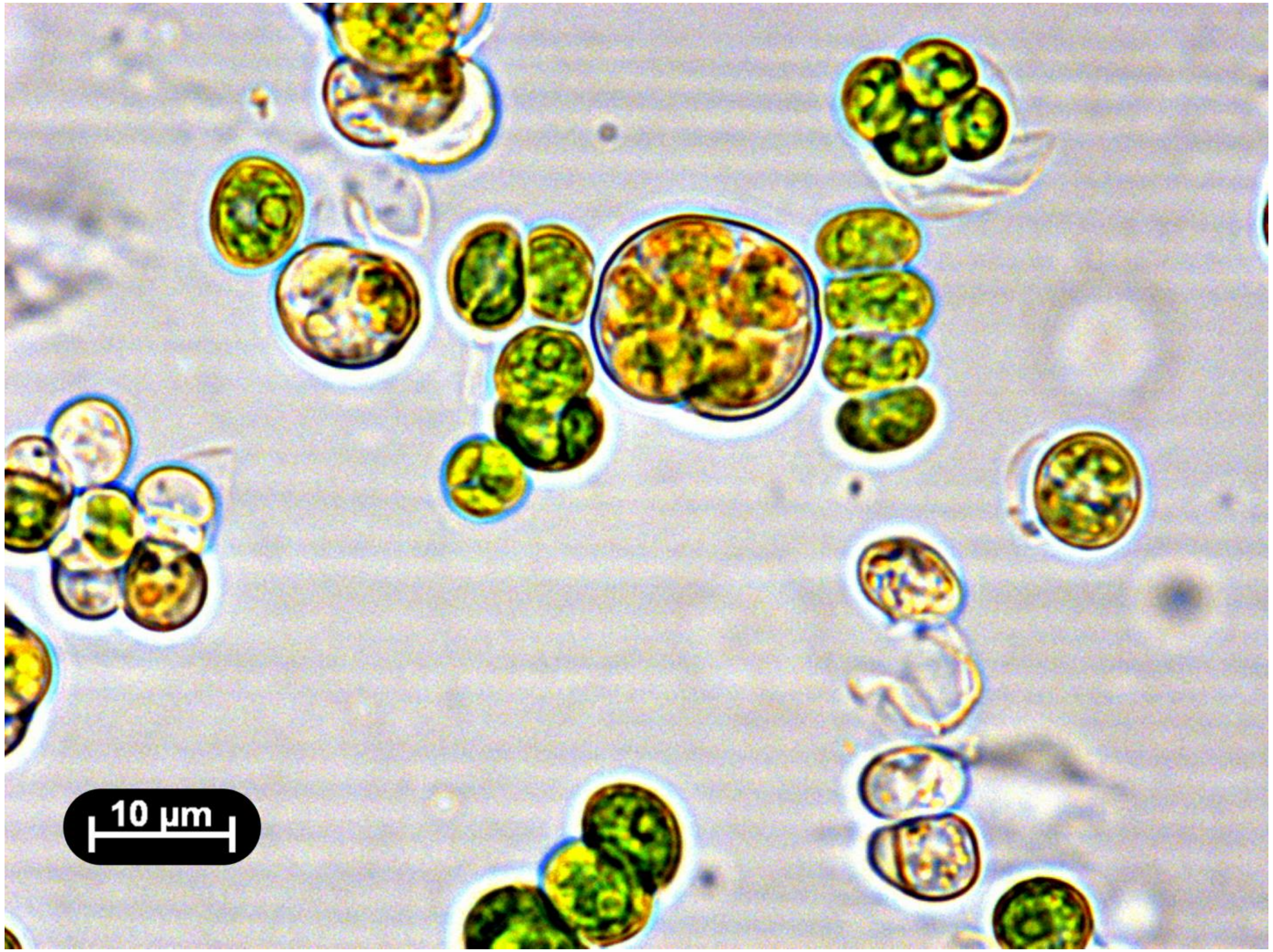

Parachlorella sp. TCF-53g

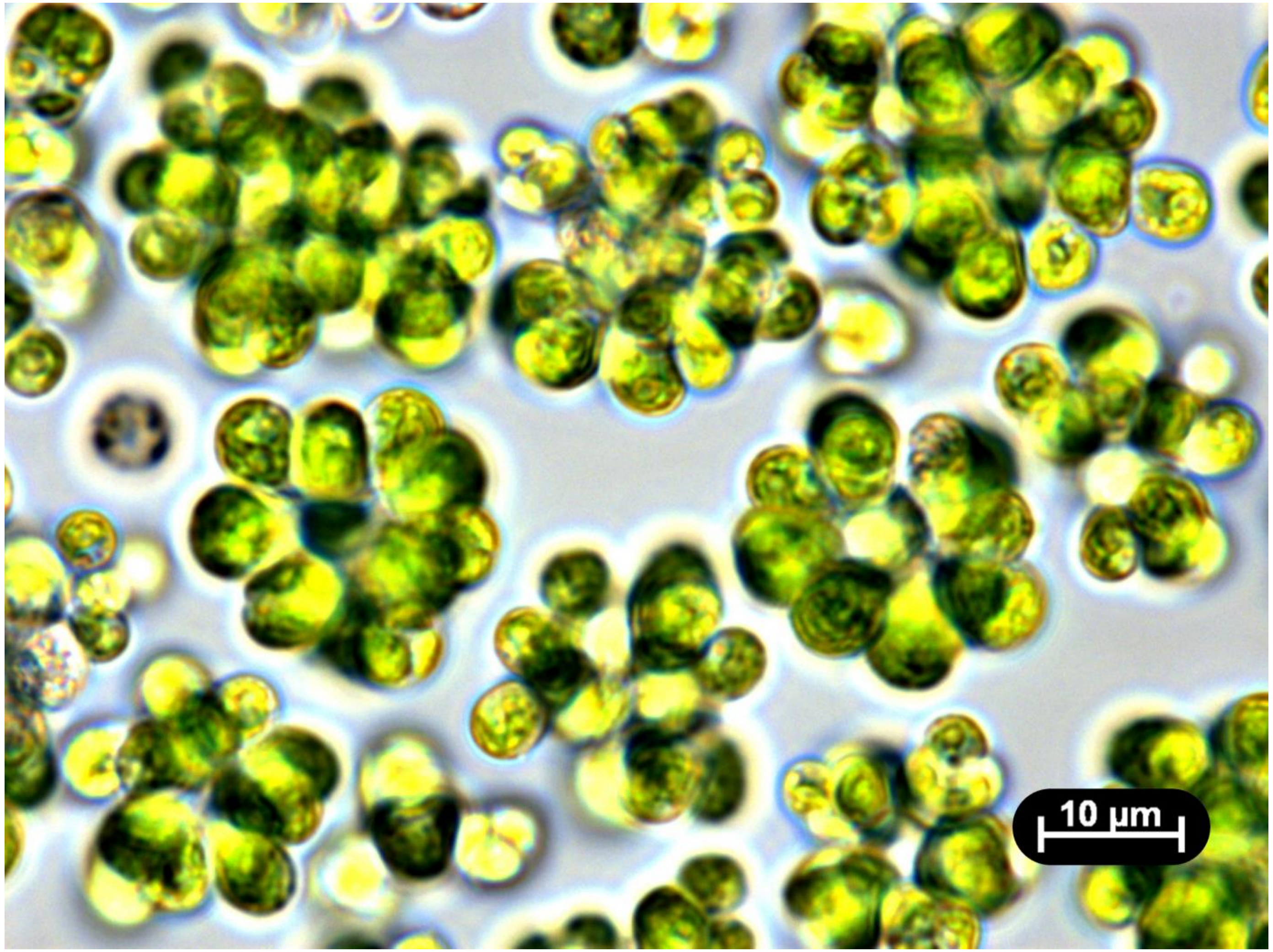

Tetradesmus dimorphus TCF-54g

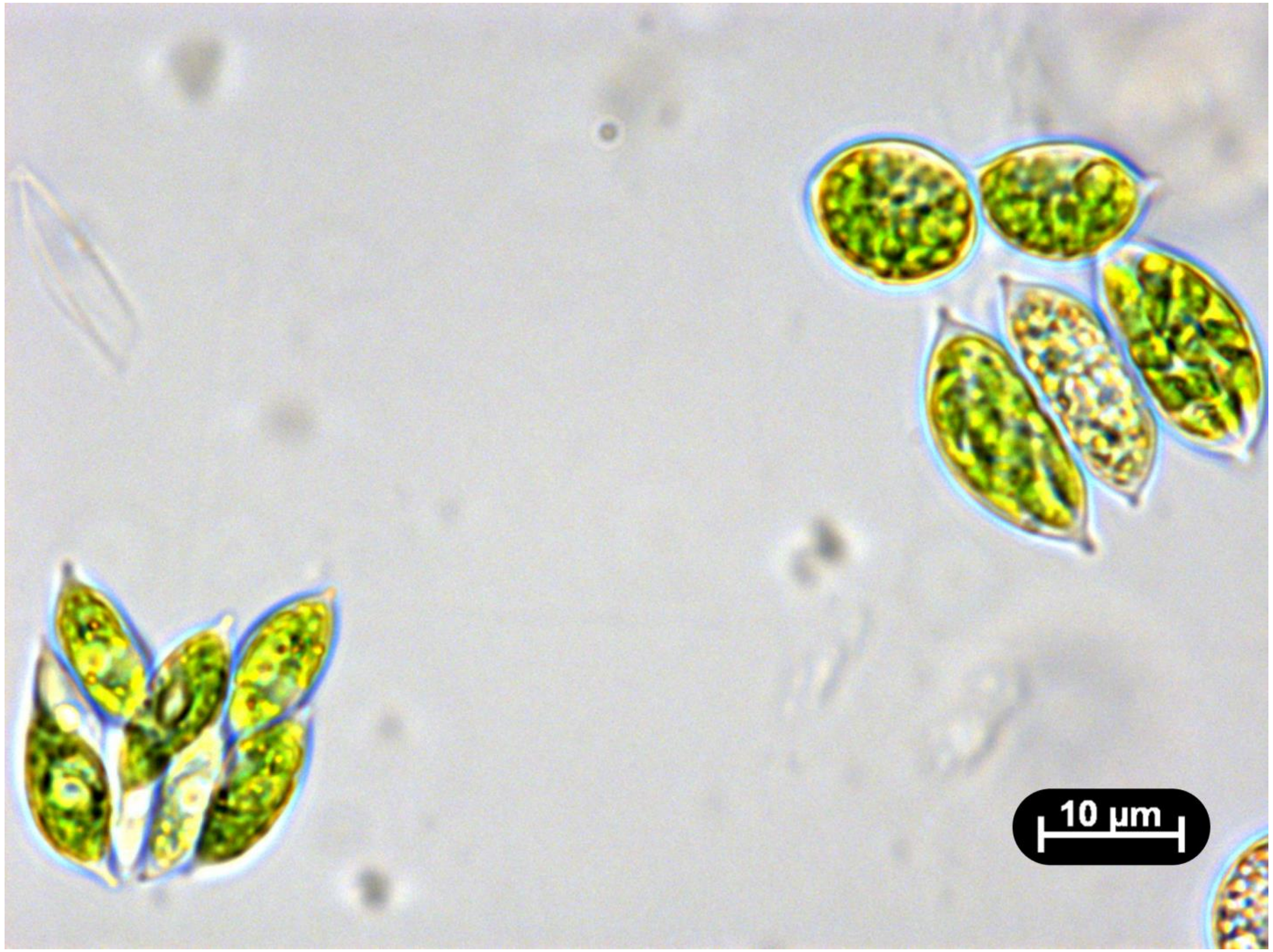

Tetradesmus obliquus TCF-55g

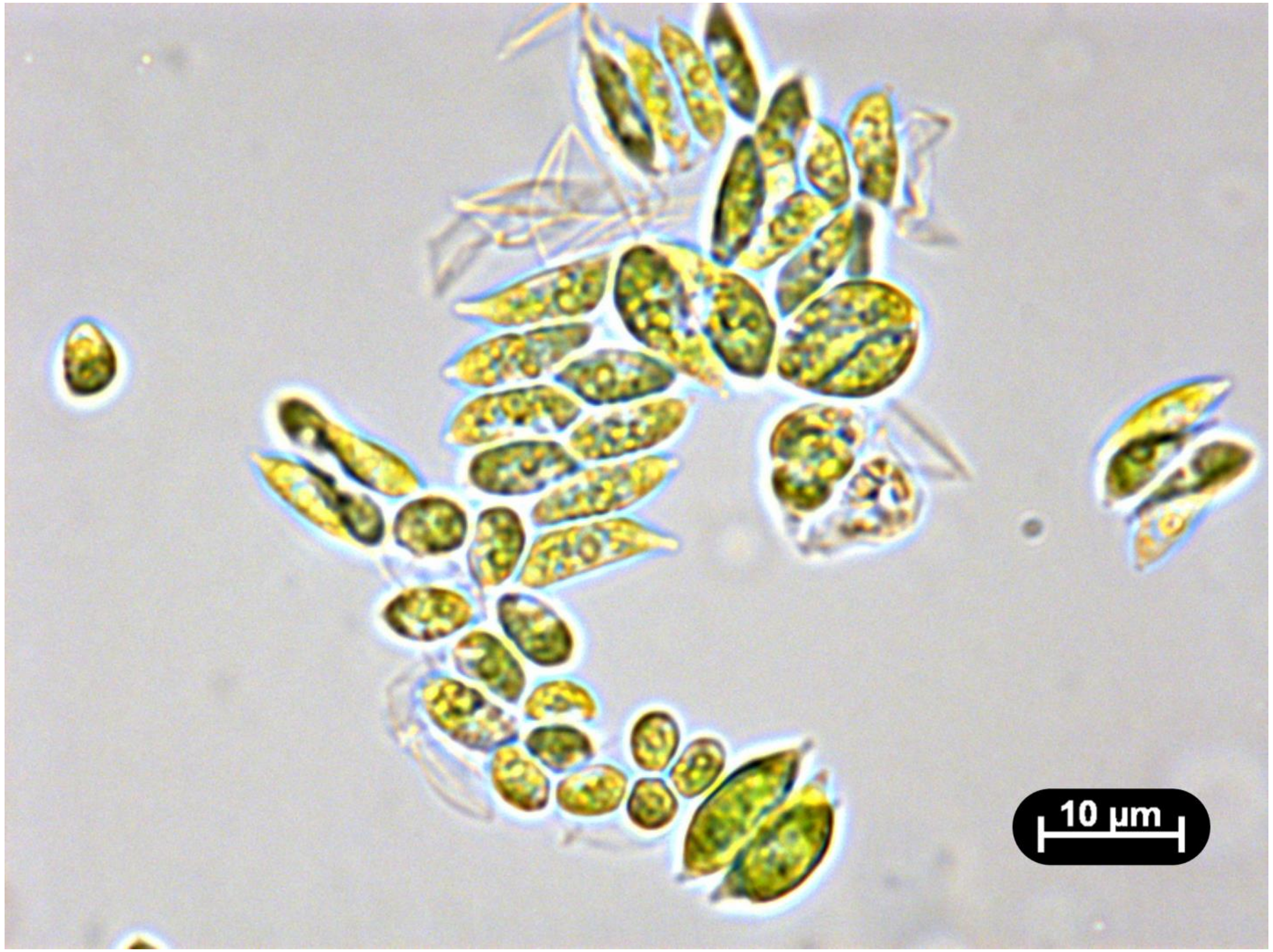

Chlamydomonas pulvinata TCF-56g

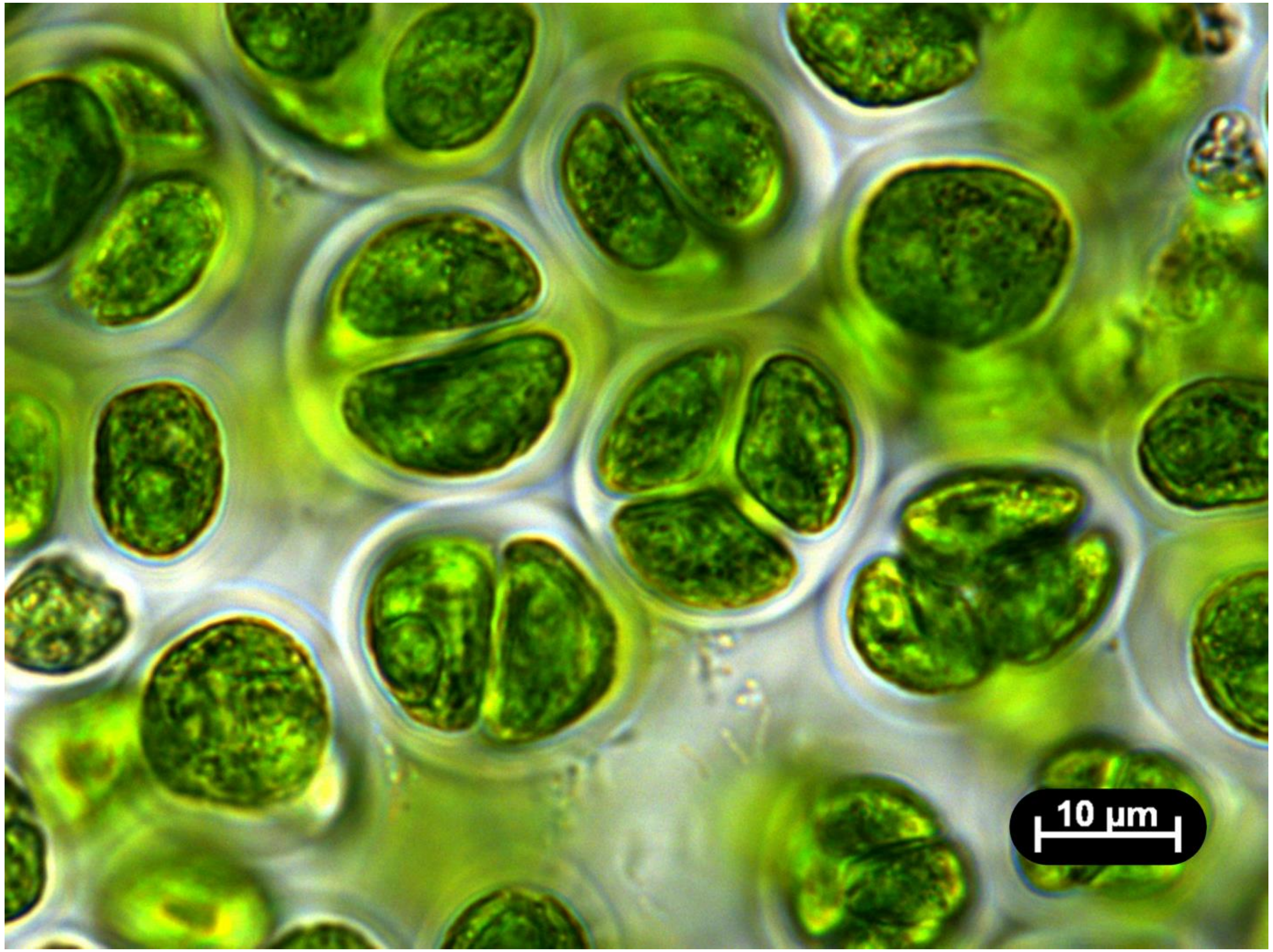

Chlorella sorokiniana TCF-57g

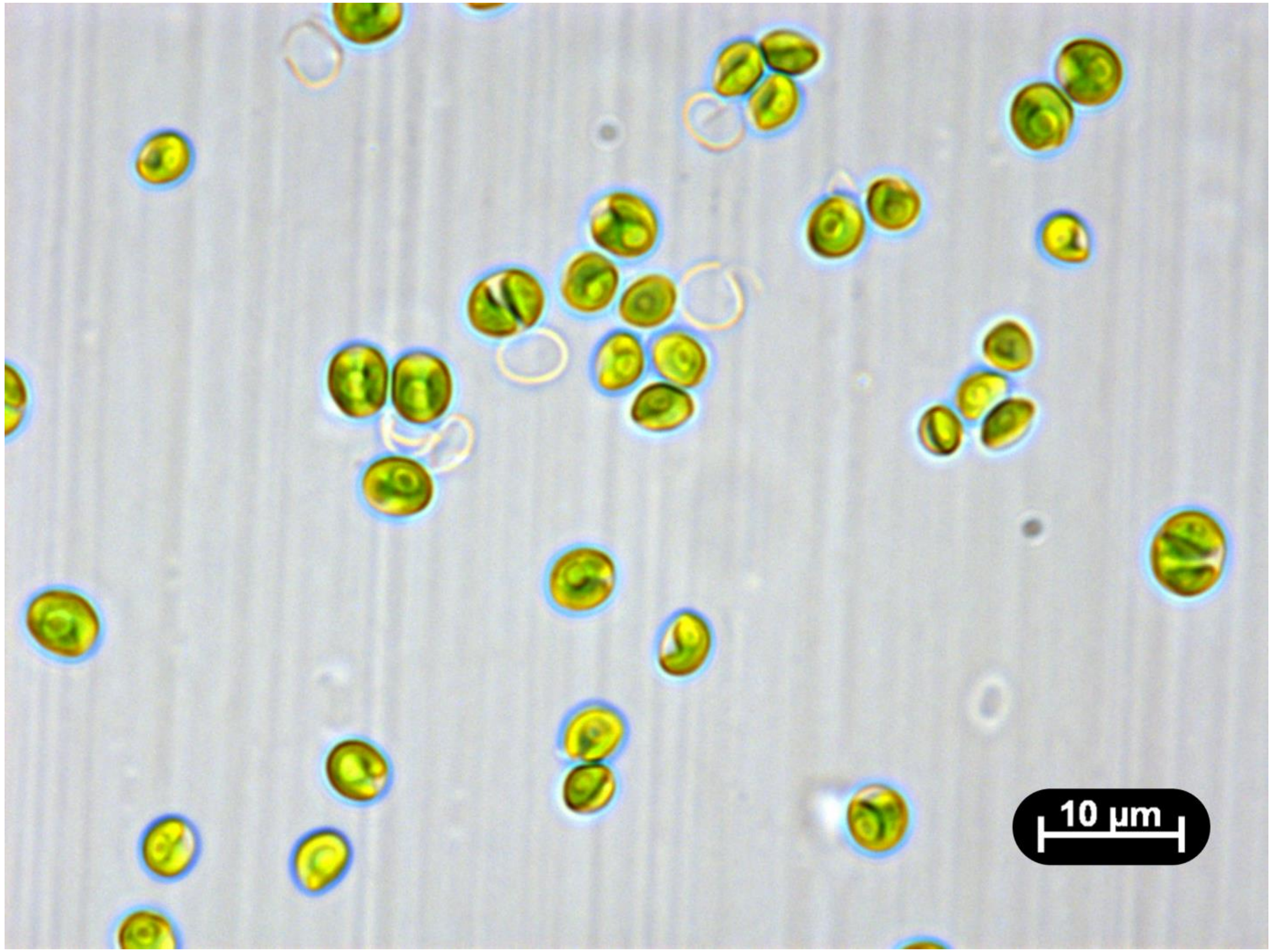

Desmodesmus abundans TCF-58g

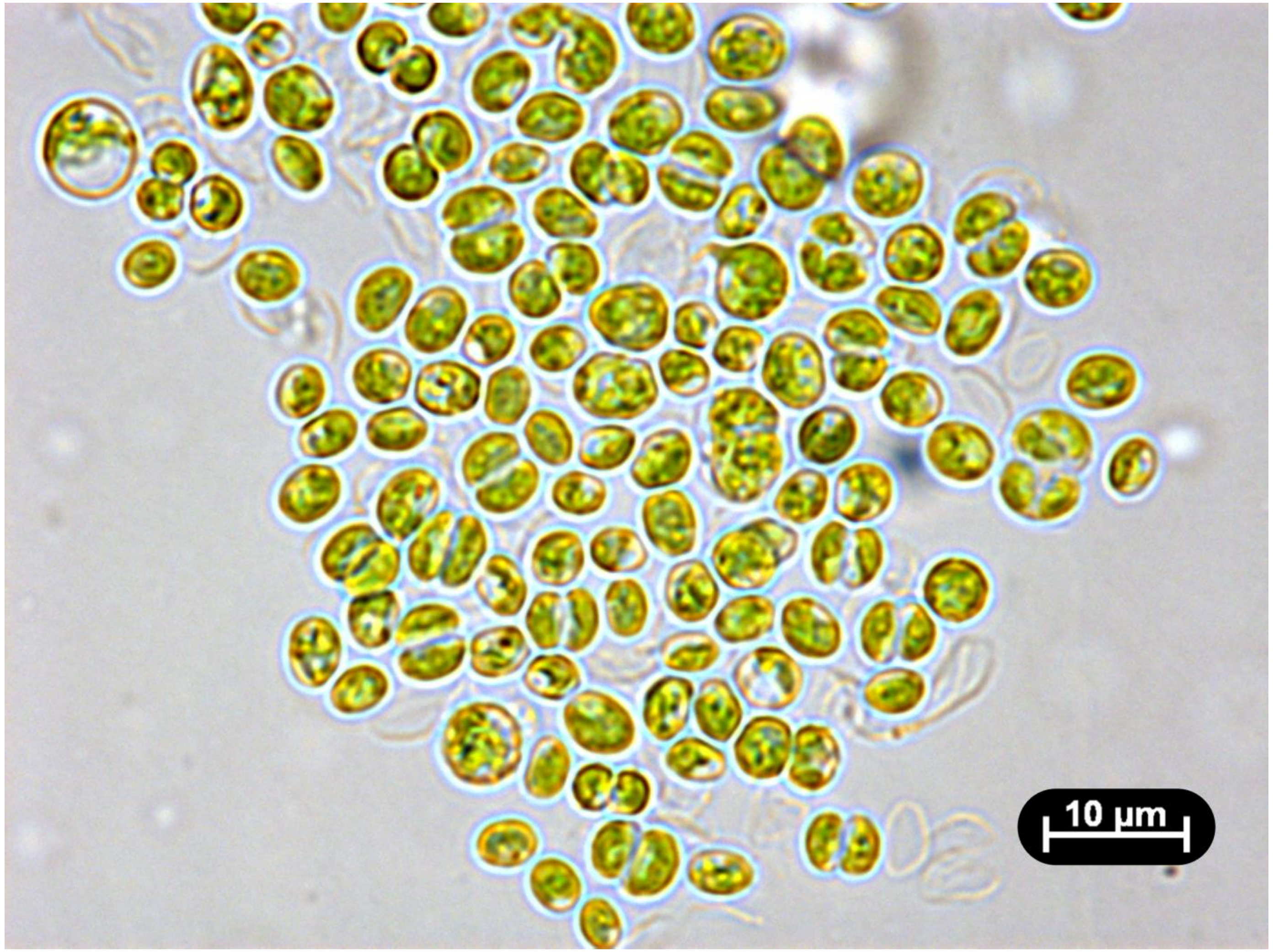

Tetradesmus sp. TCF-59g

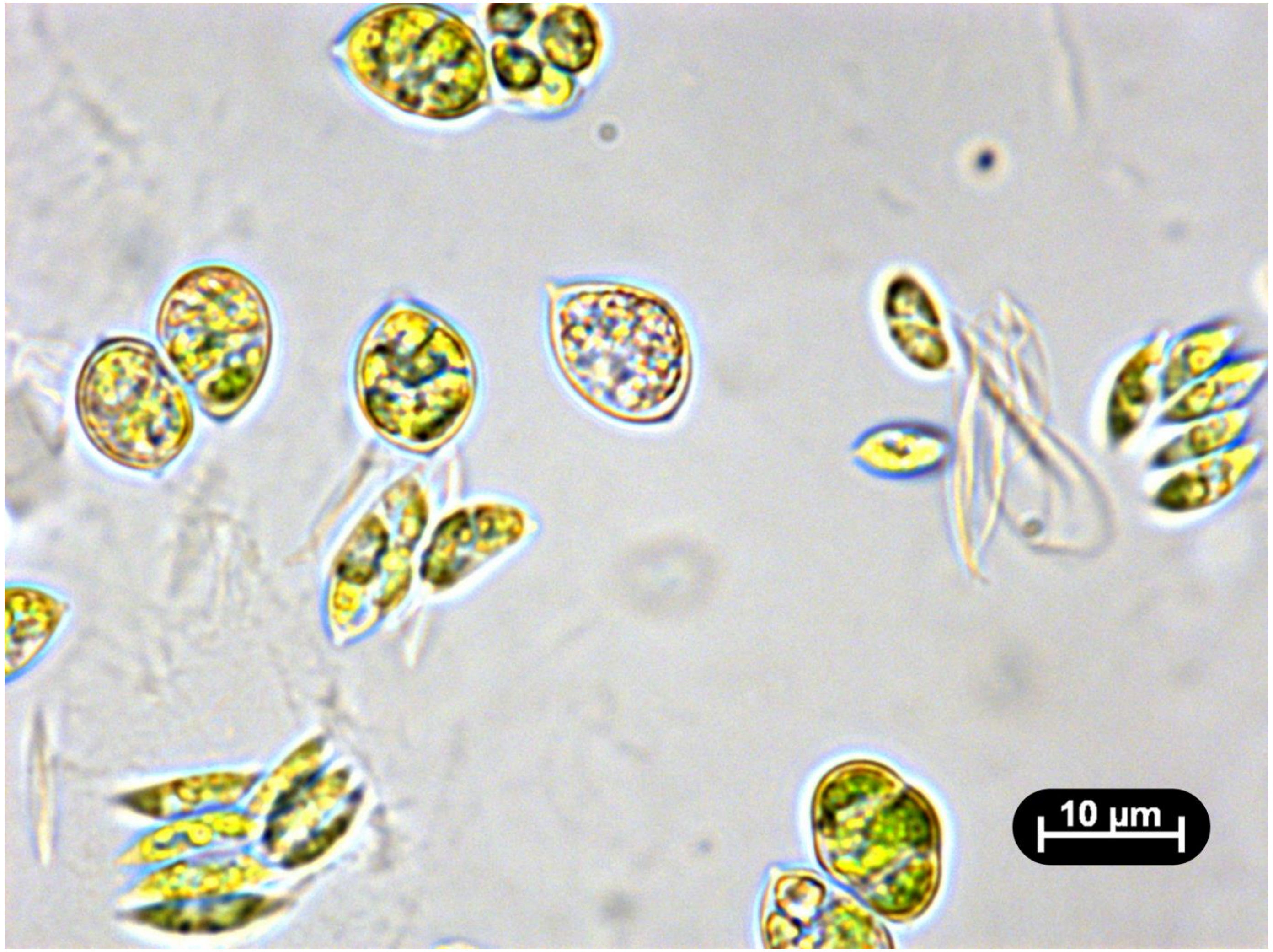

Tetradesmus sp. TCF-60g

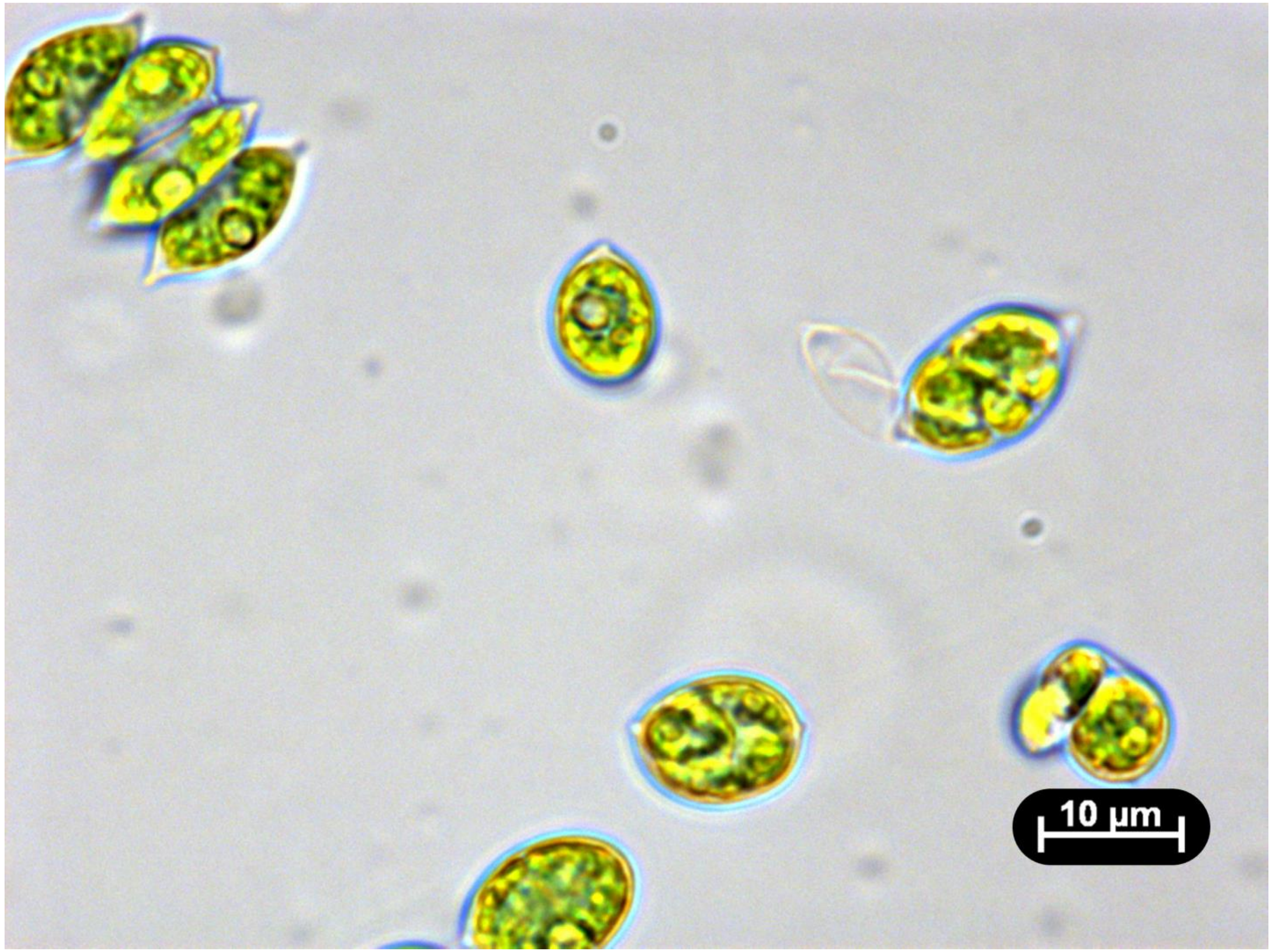

Tetradesmus sp. TCF-61g

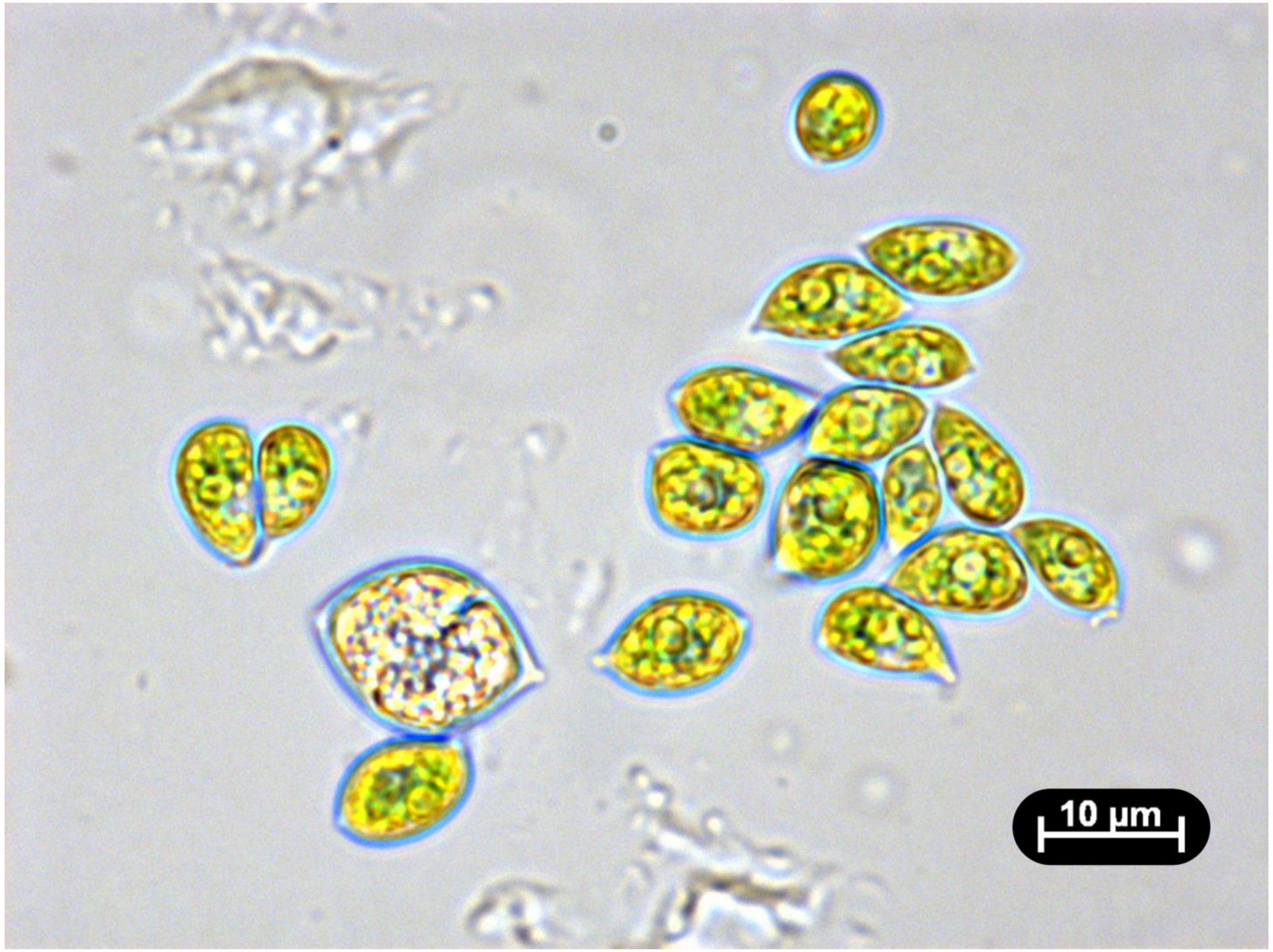

Tetradesmus sp. TCF-62g

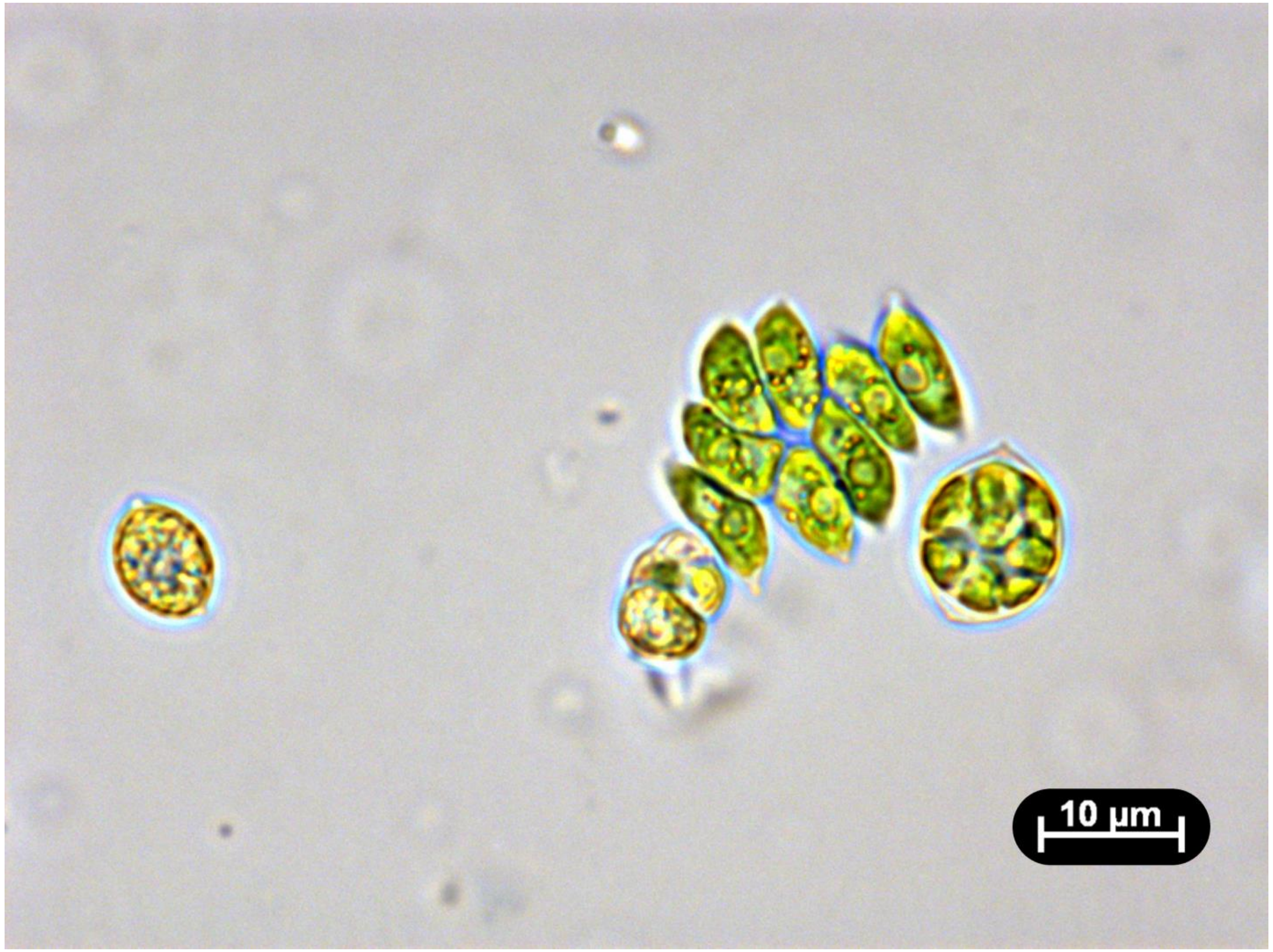

Desmodesmus armatus TCF-63g

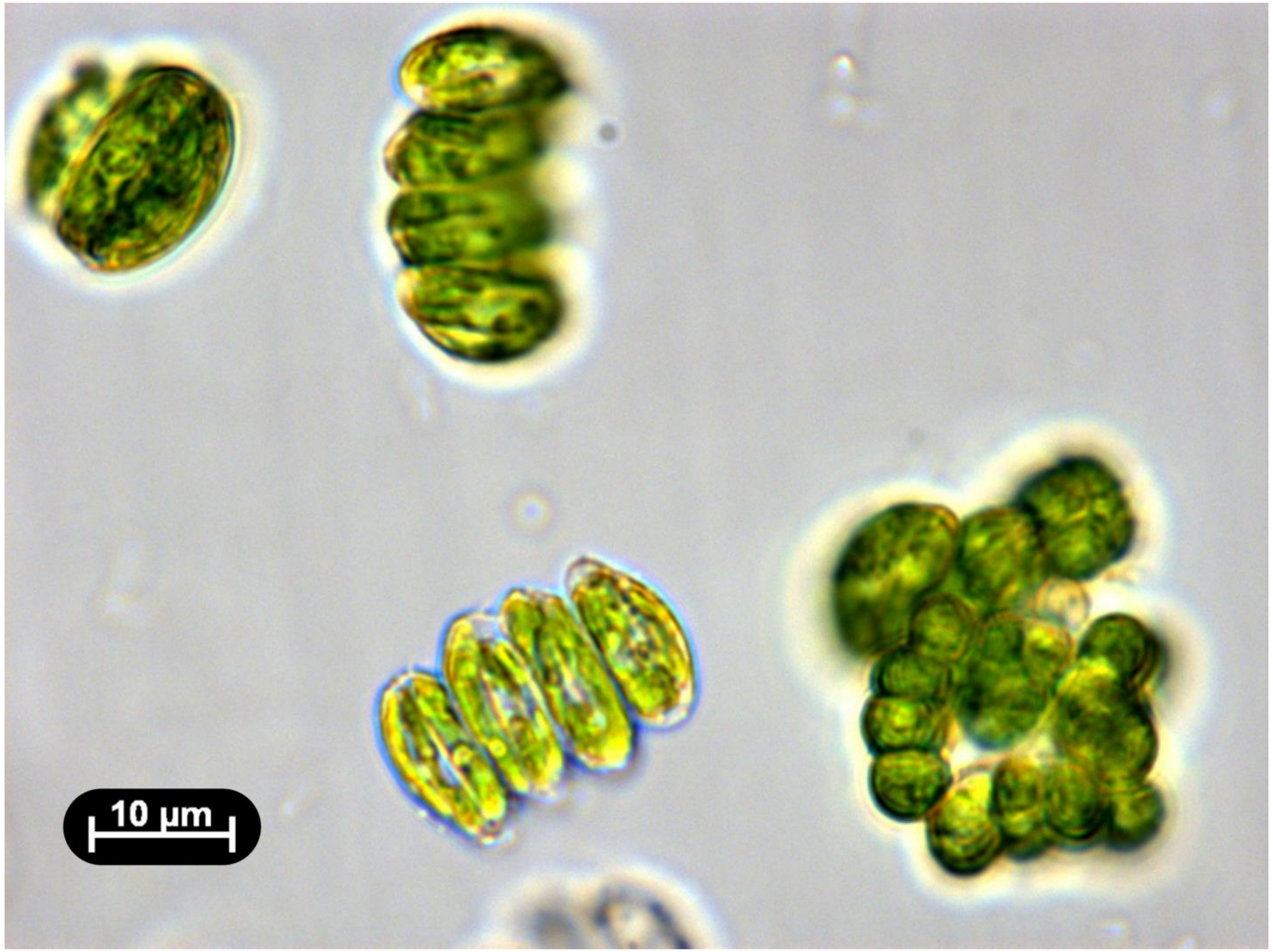

Desmodesmus sp. TCF-64g

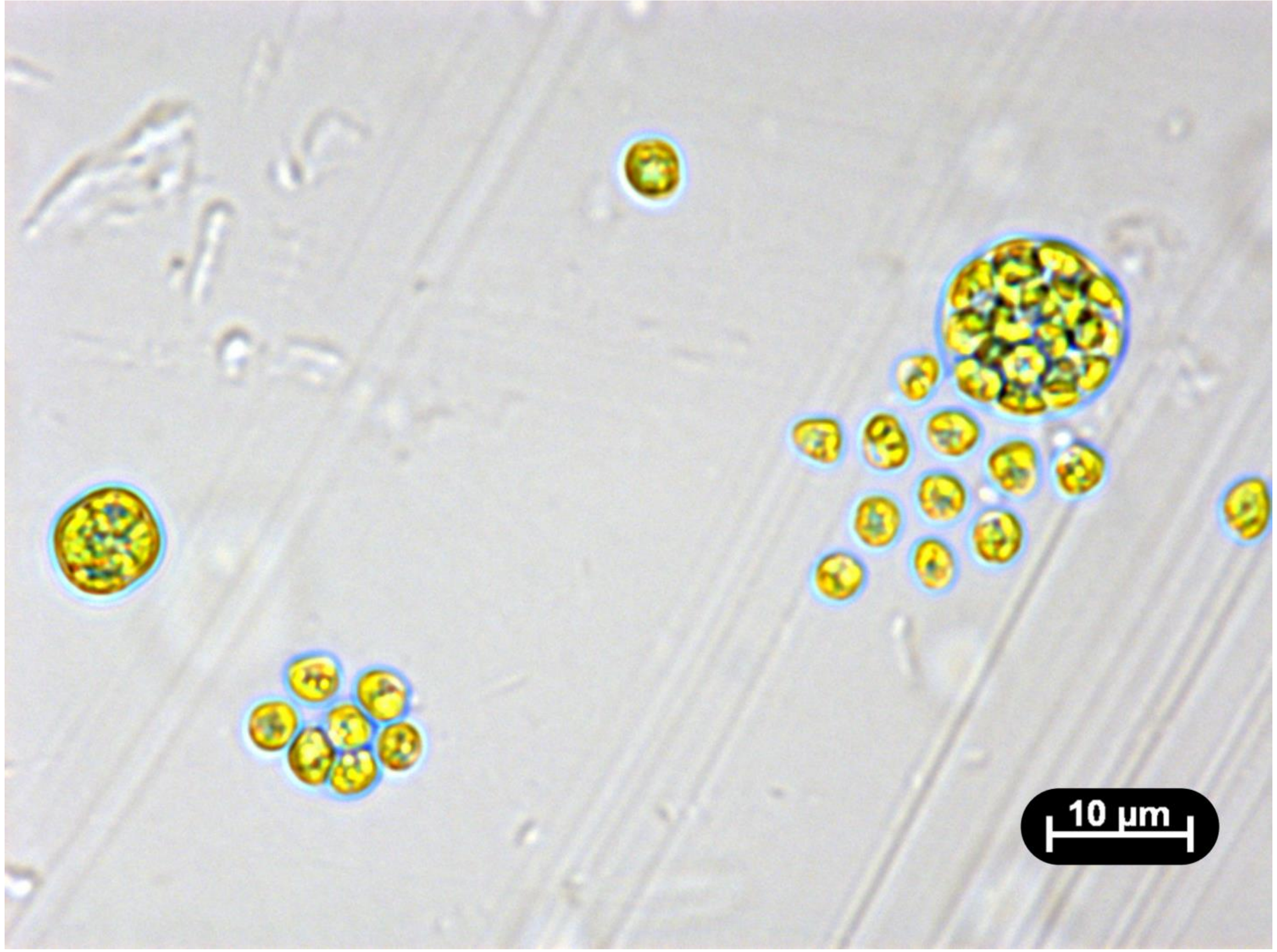

Desmodesmus sp. TCF-65g

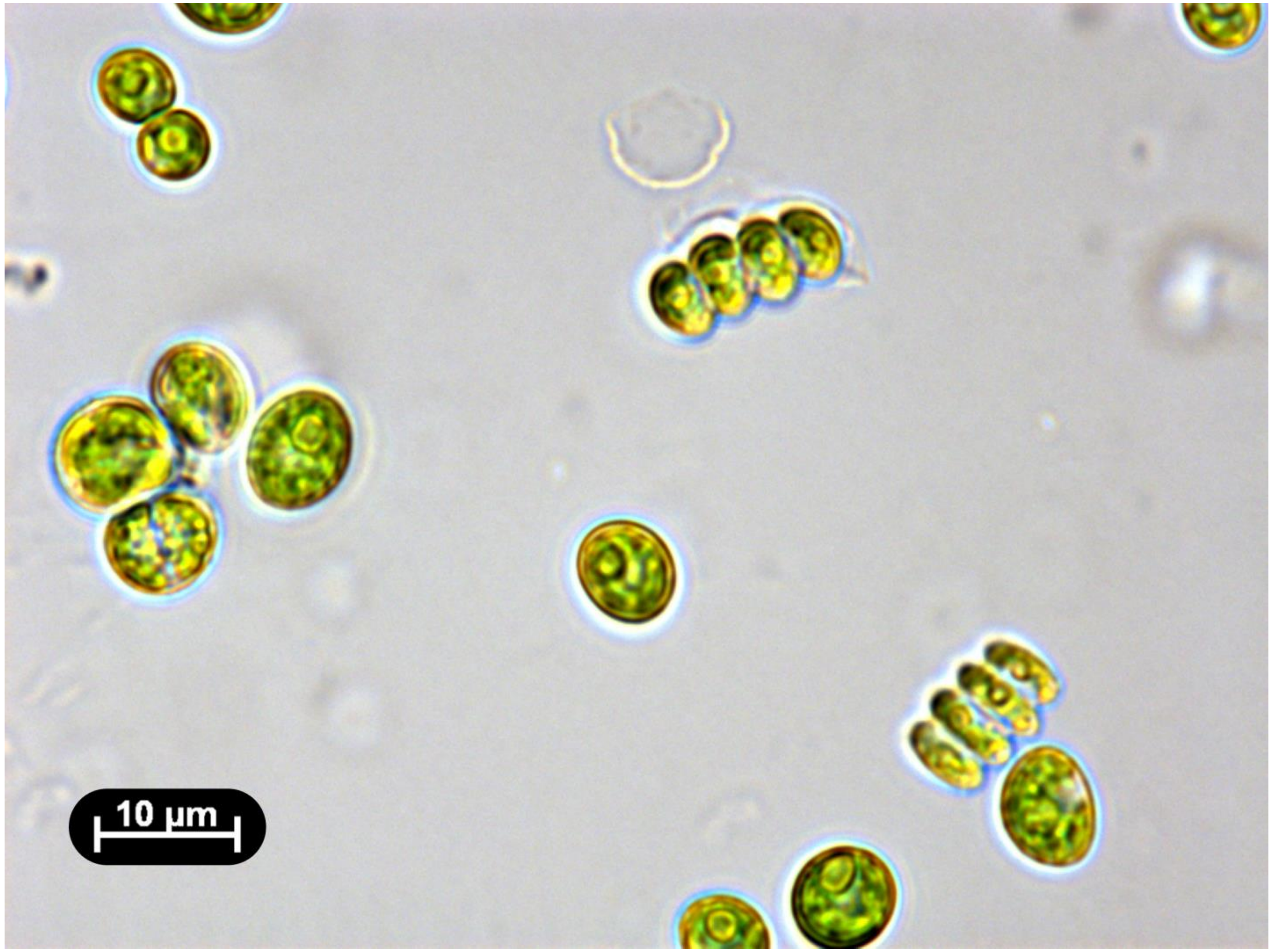

Chlorella sorokiniana TCF-66g

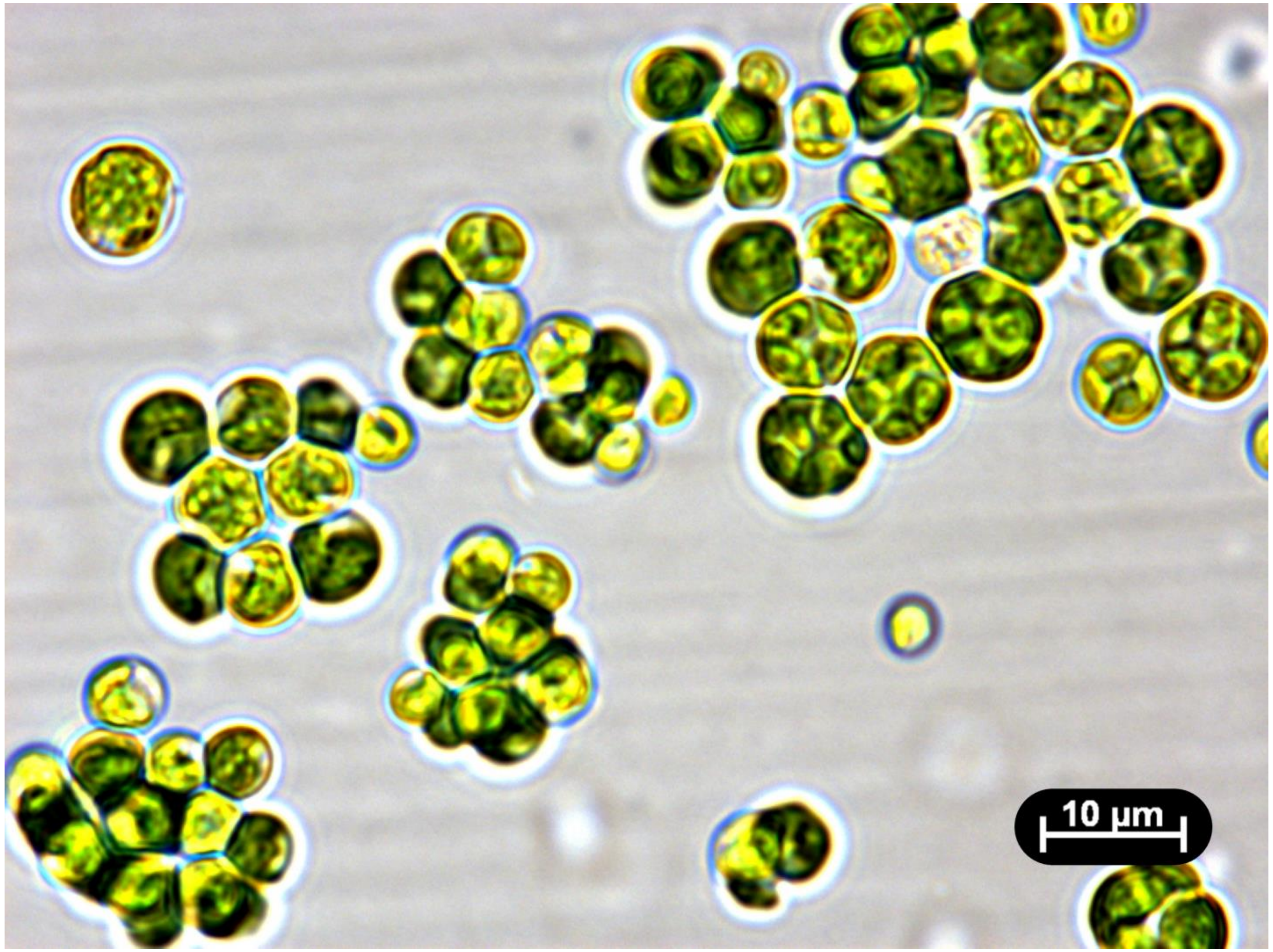

Desmodesmus abundans TCF-67g

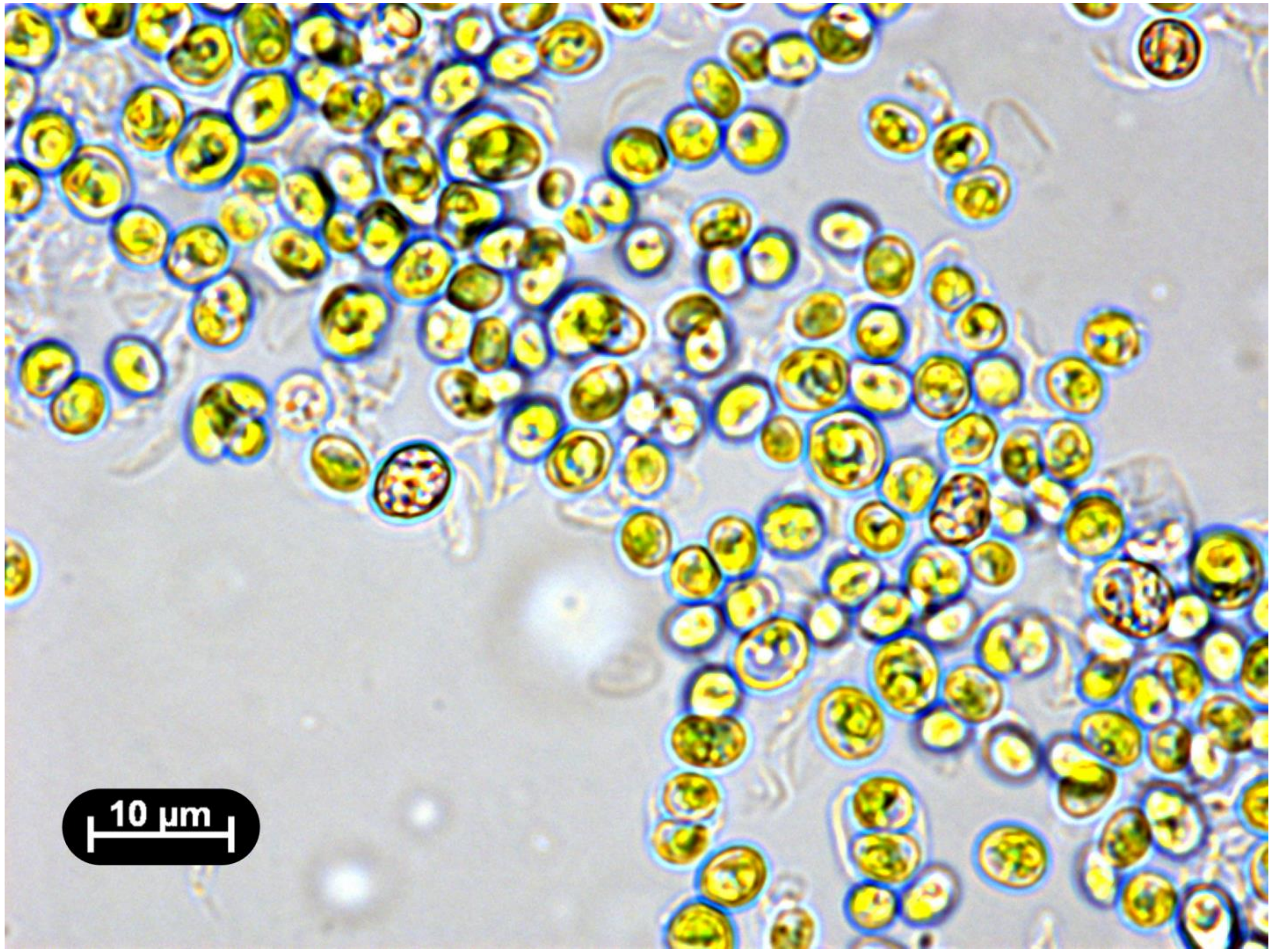

Desmodesmus abundans TCF-68g

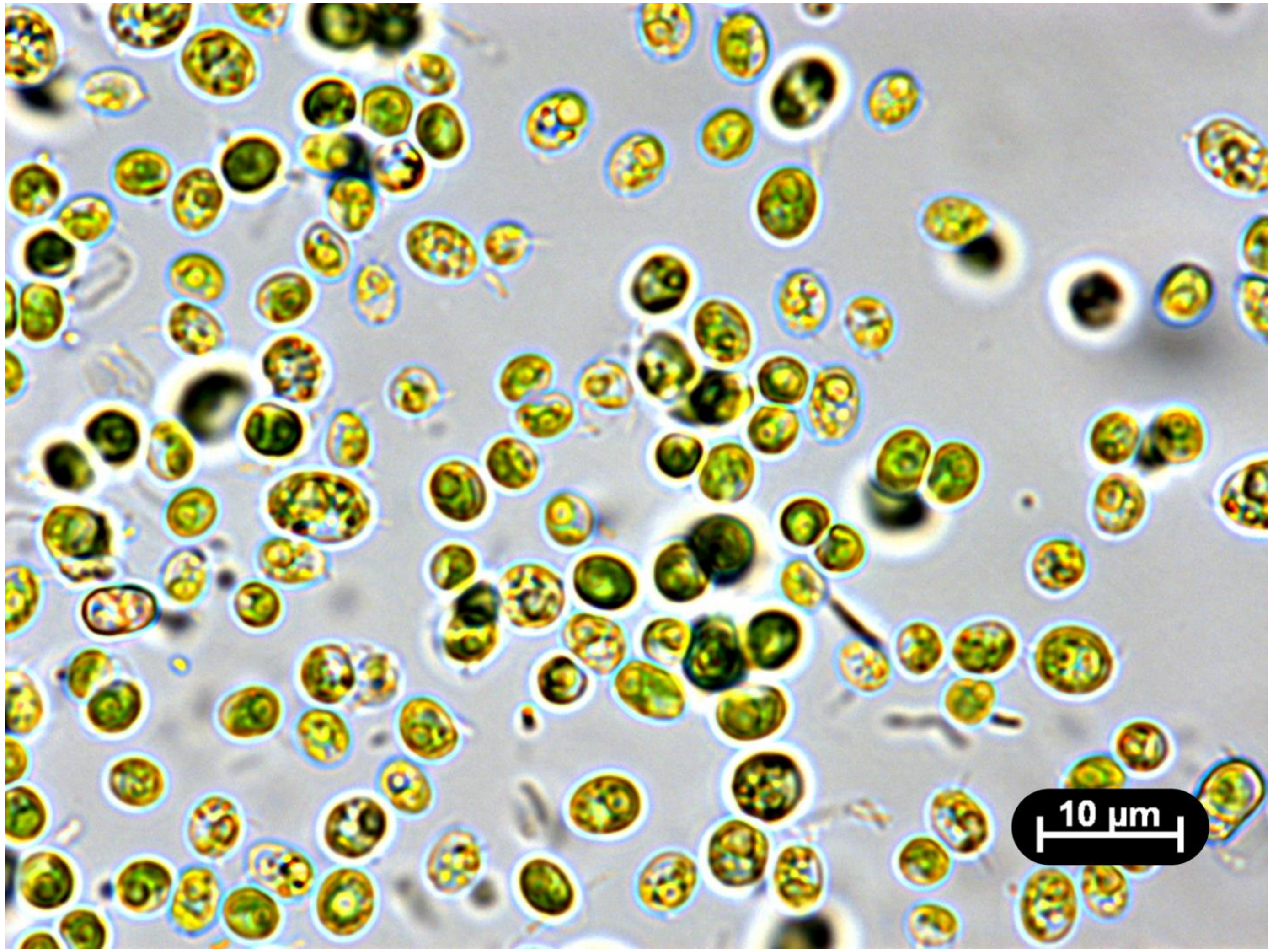

Desmodesmus sp. TCF-69g

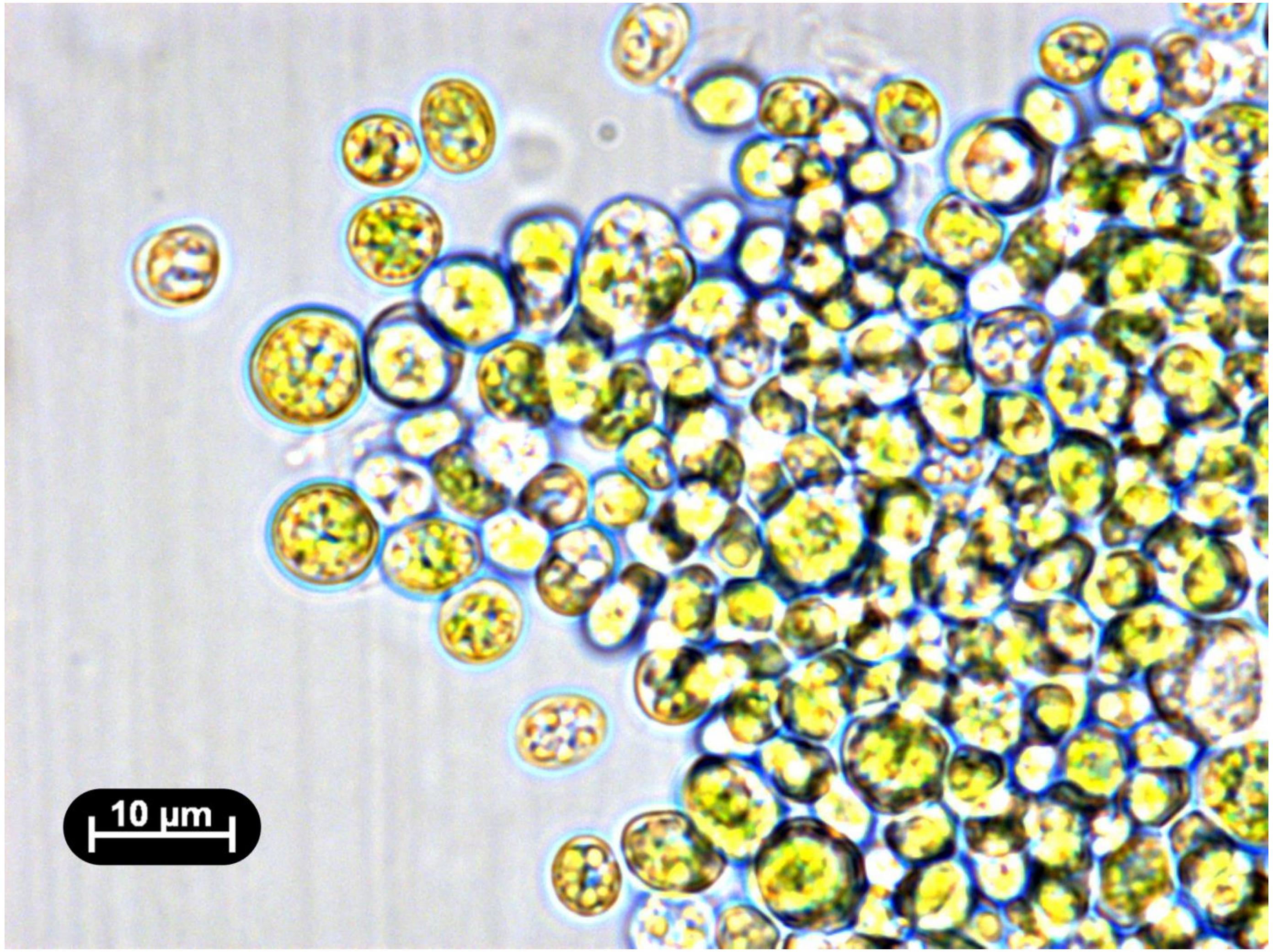

Desmodesmus sp. TCF-70g

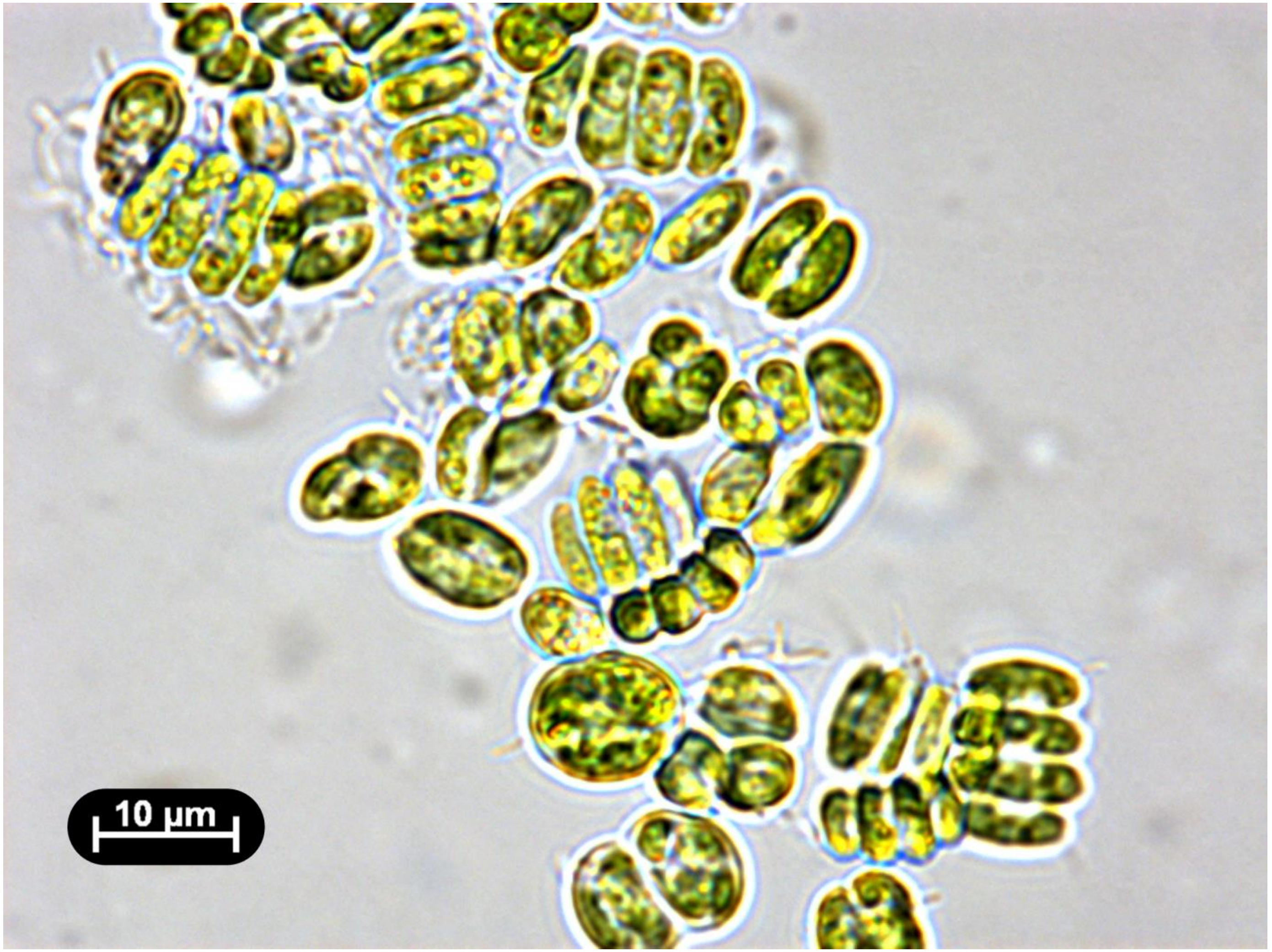

Dictyosphaerium sp. TCF-71g

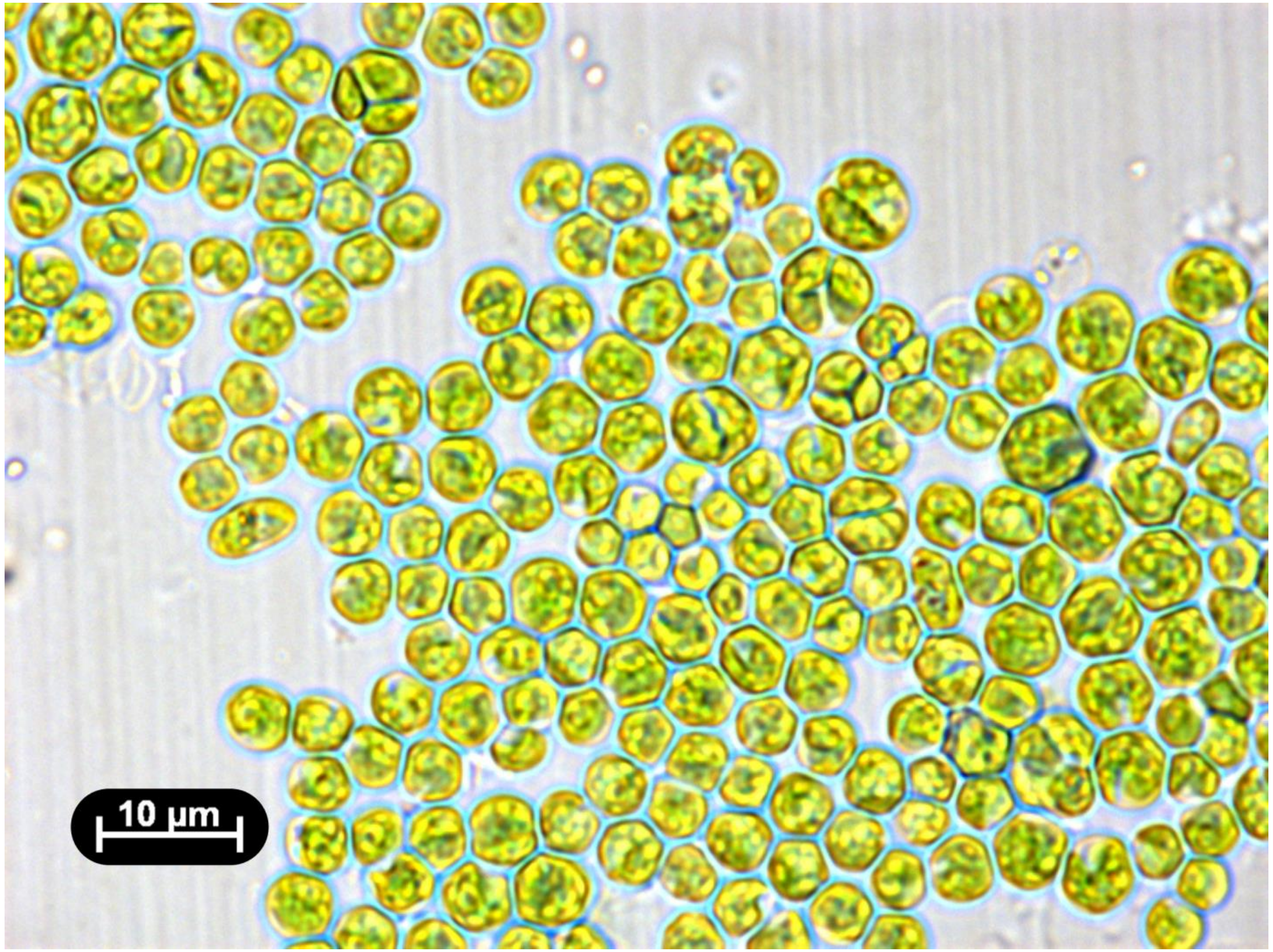

Dictyosphaerium sp. TCF-72g

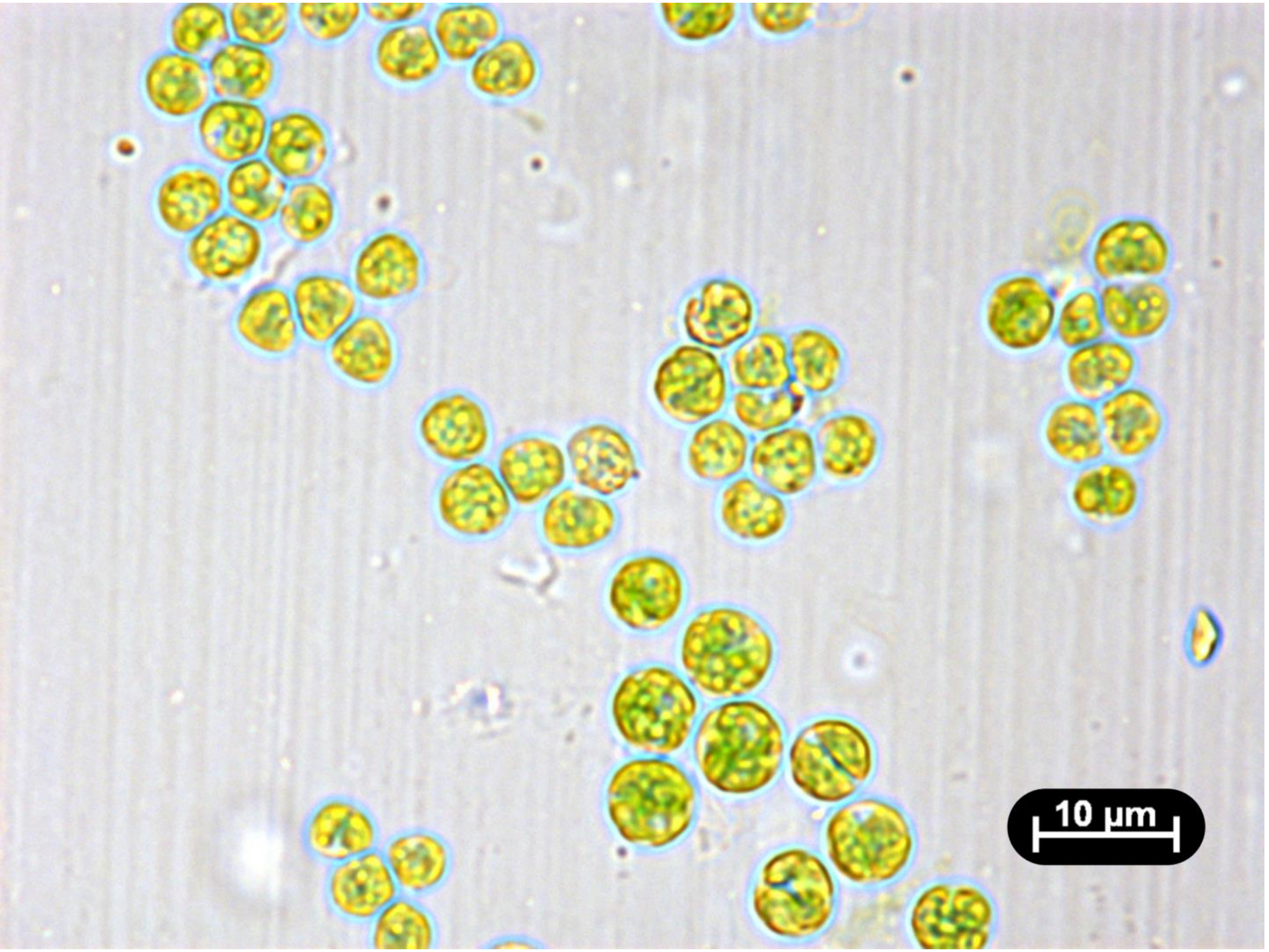

Tetradesmus obliquus TCF-73g

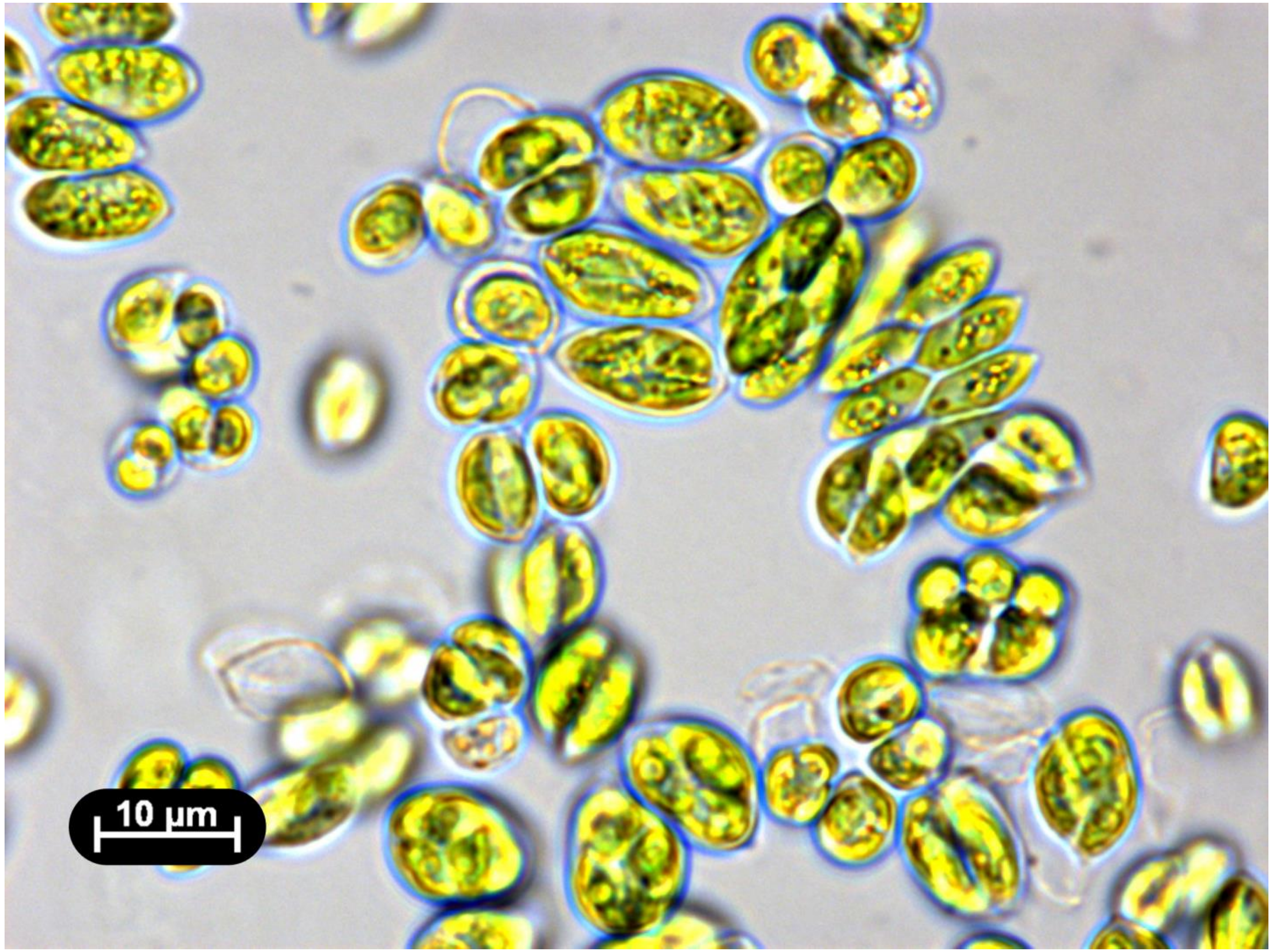

Chlorella sorokiniana TCF-74g

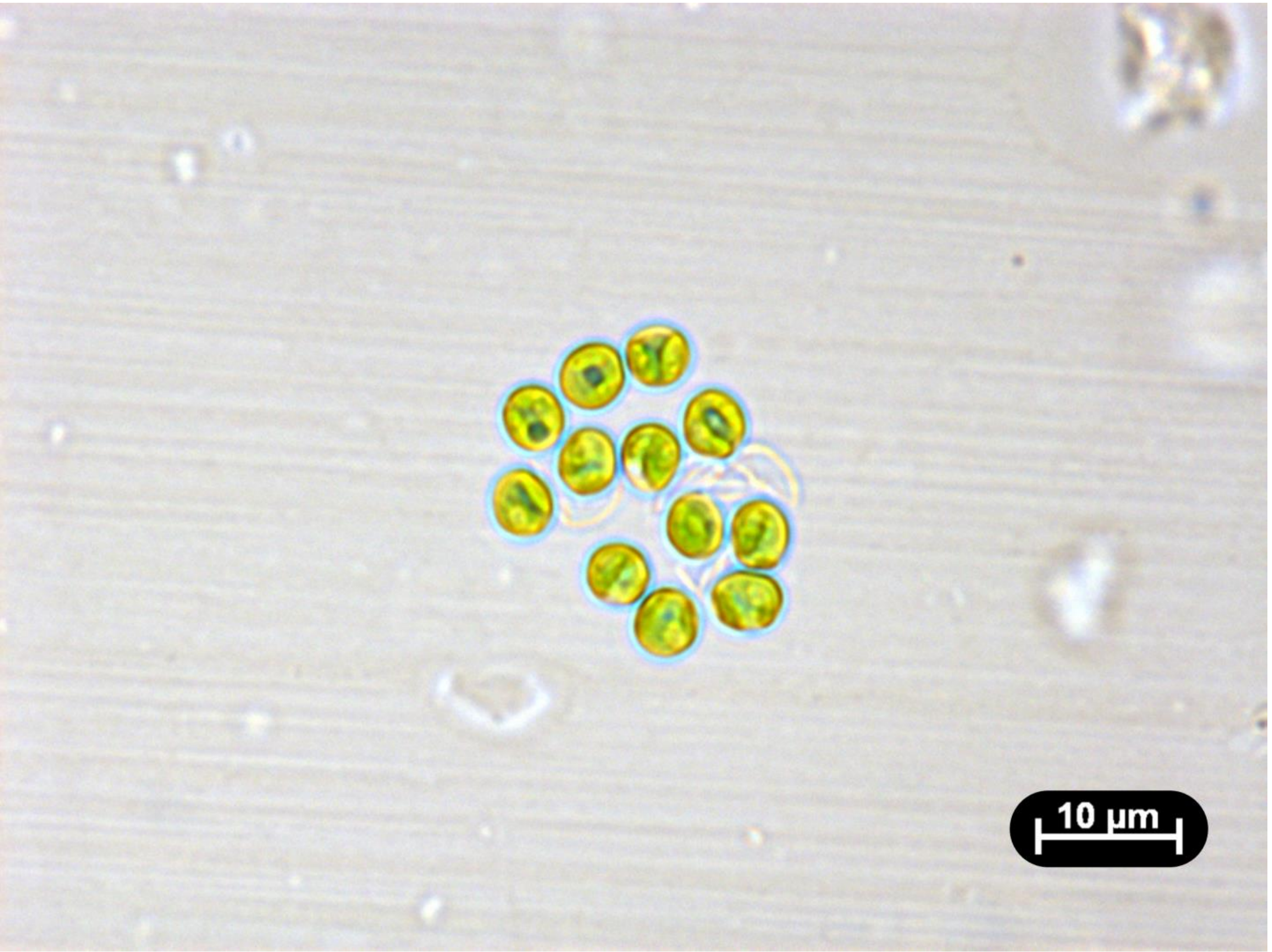

Caespitella pascheri TCF-75g

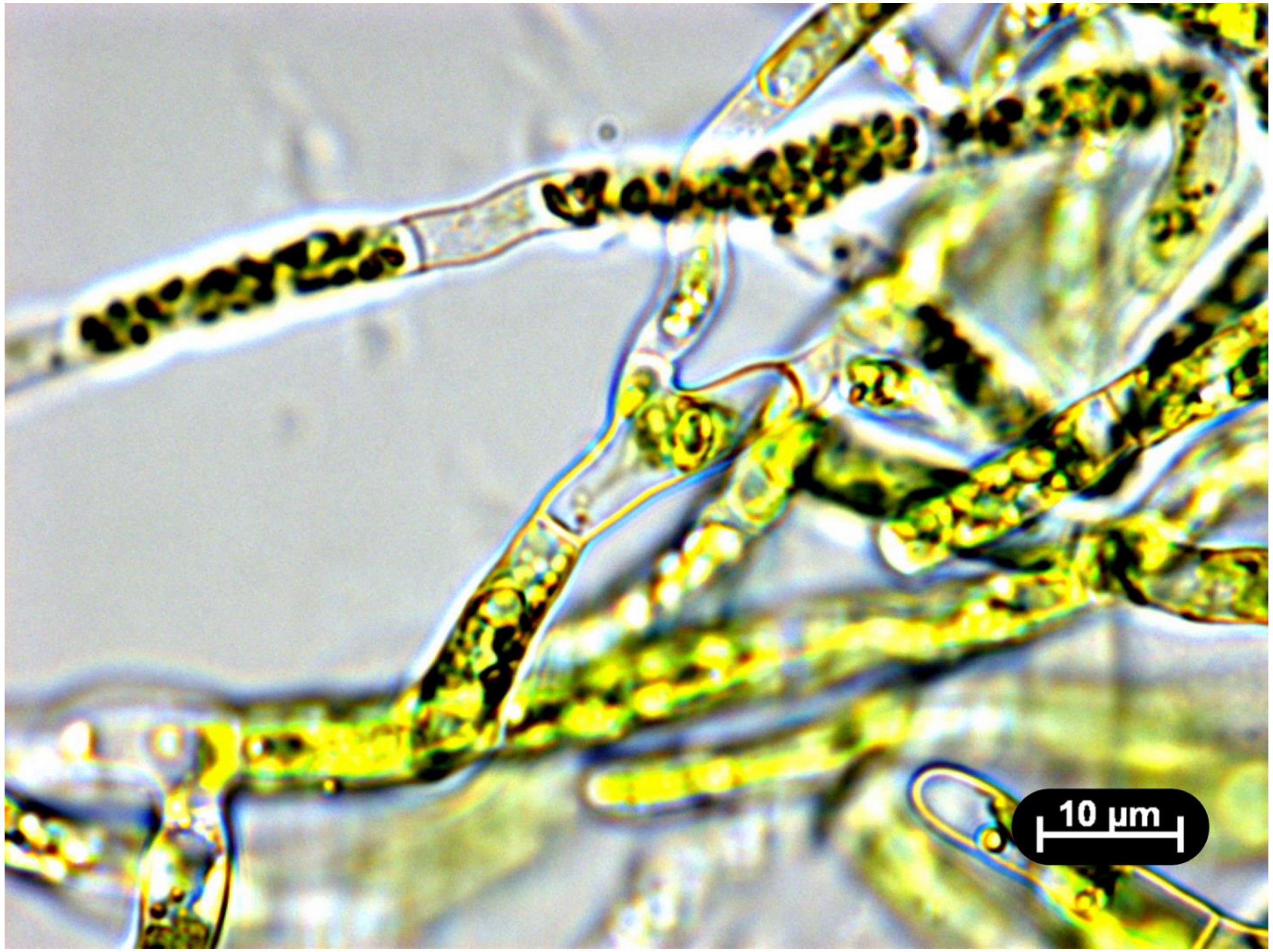

Tetradesmus obliquus TCF-76g

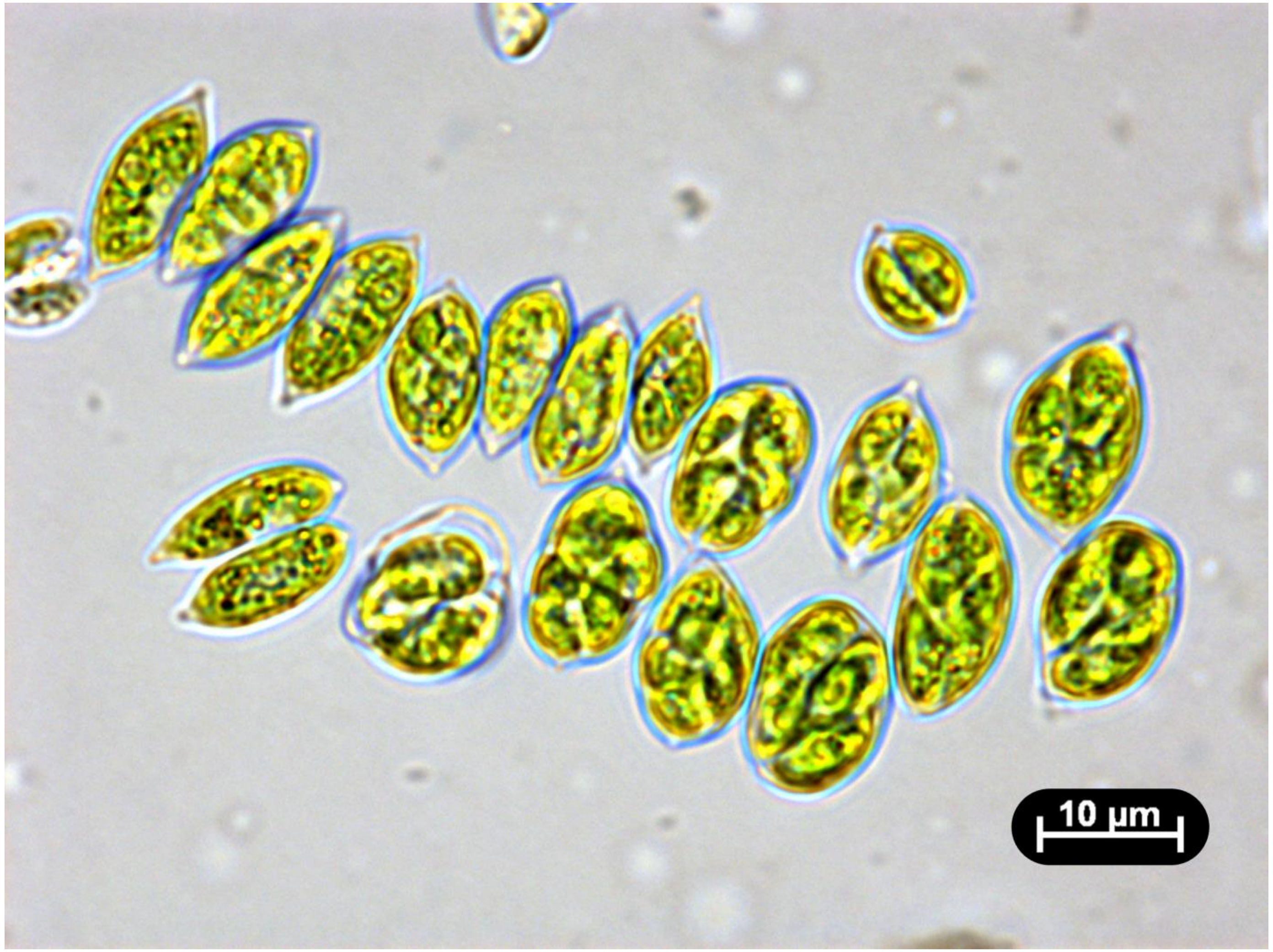

Dictyosphaerium sp. TCF-77g

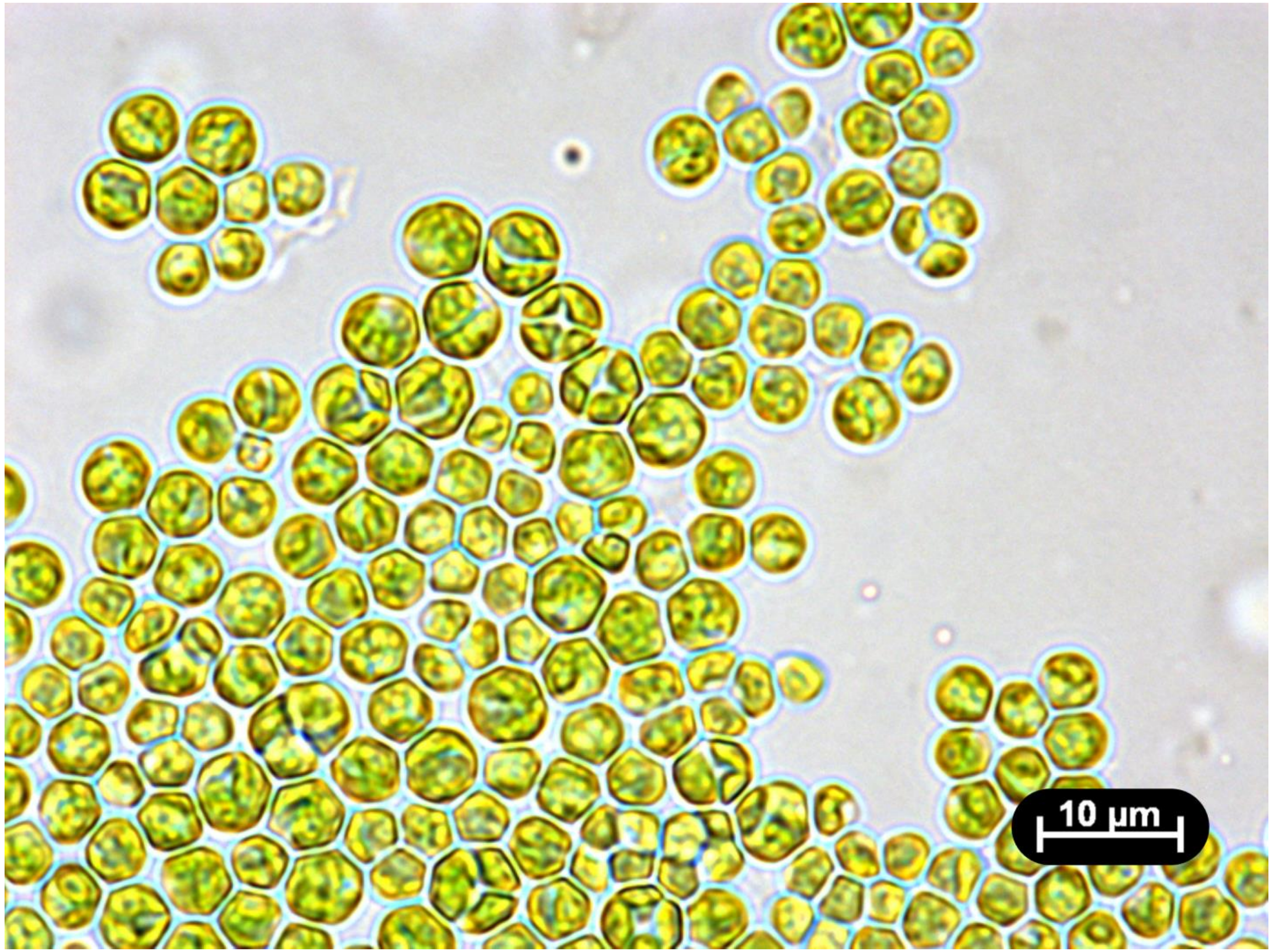

Dictyosphaerium sp. TCF-78g

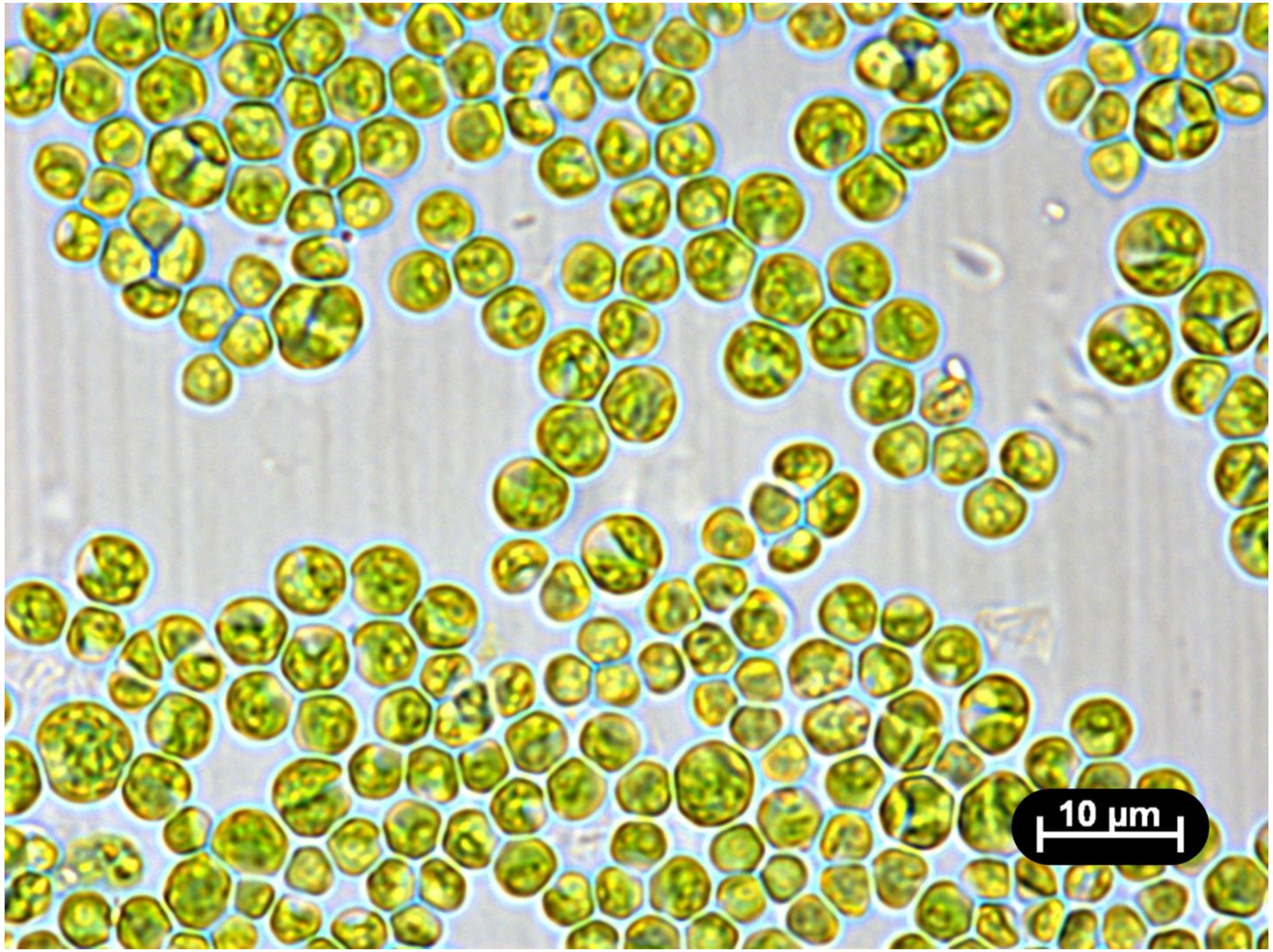

Desmodesmus sp. TCF-79g

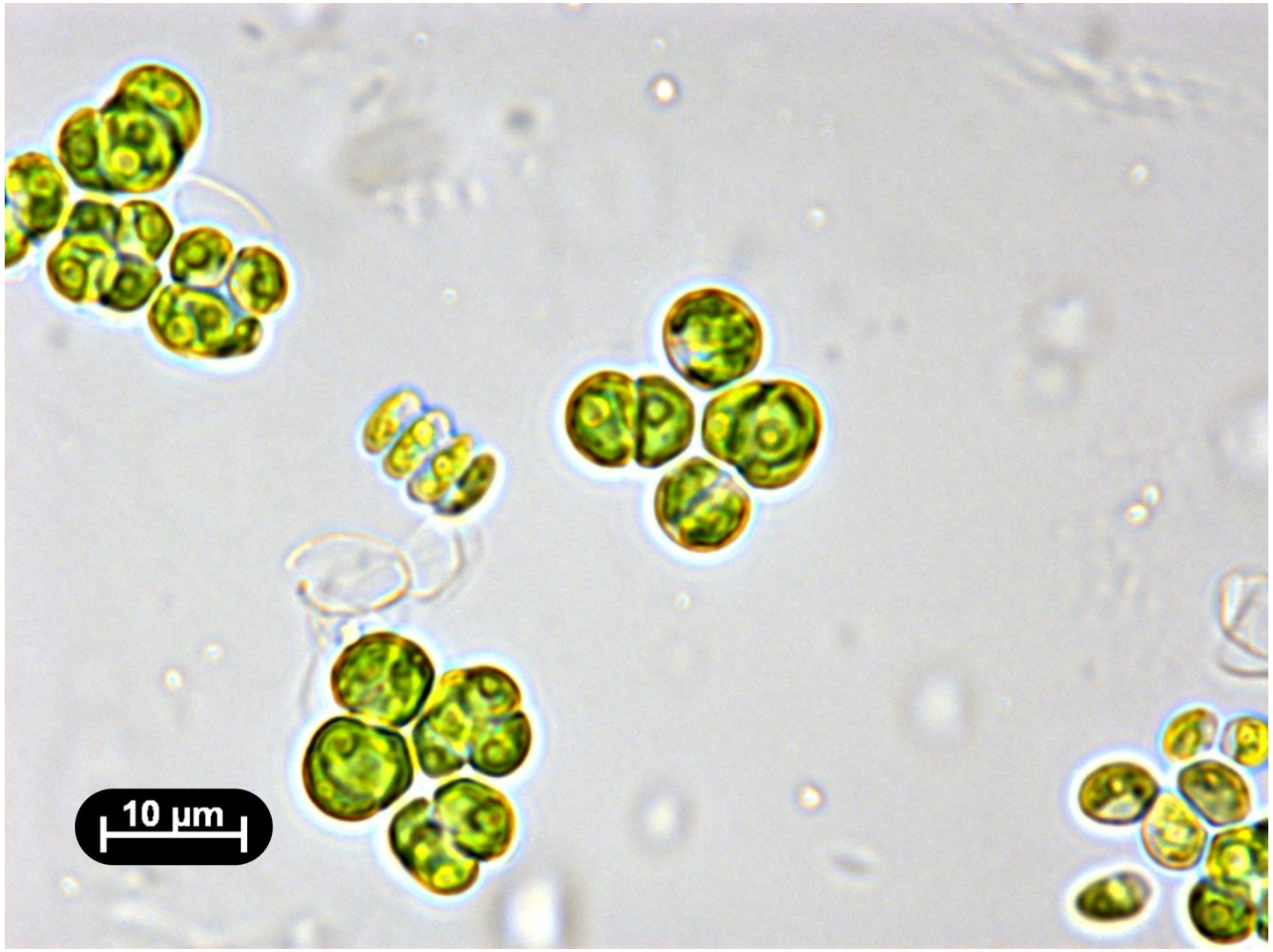

Desmodesmus sp. TCF-80g

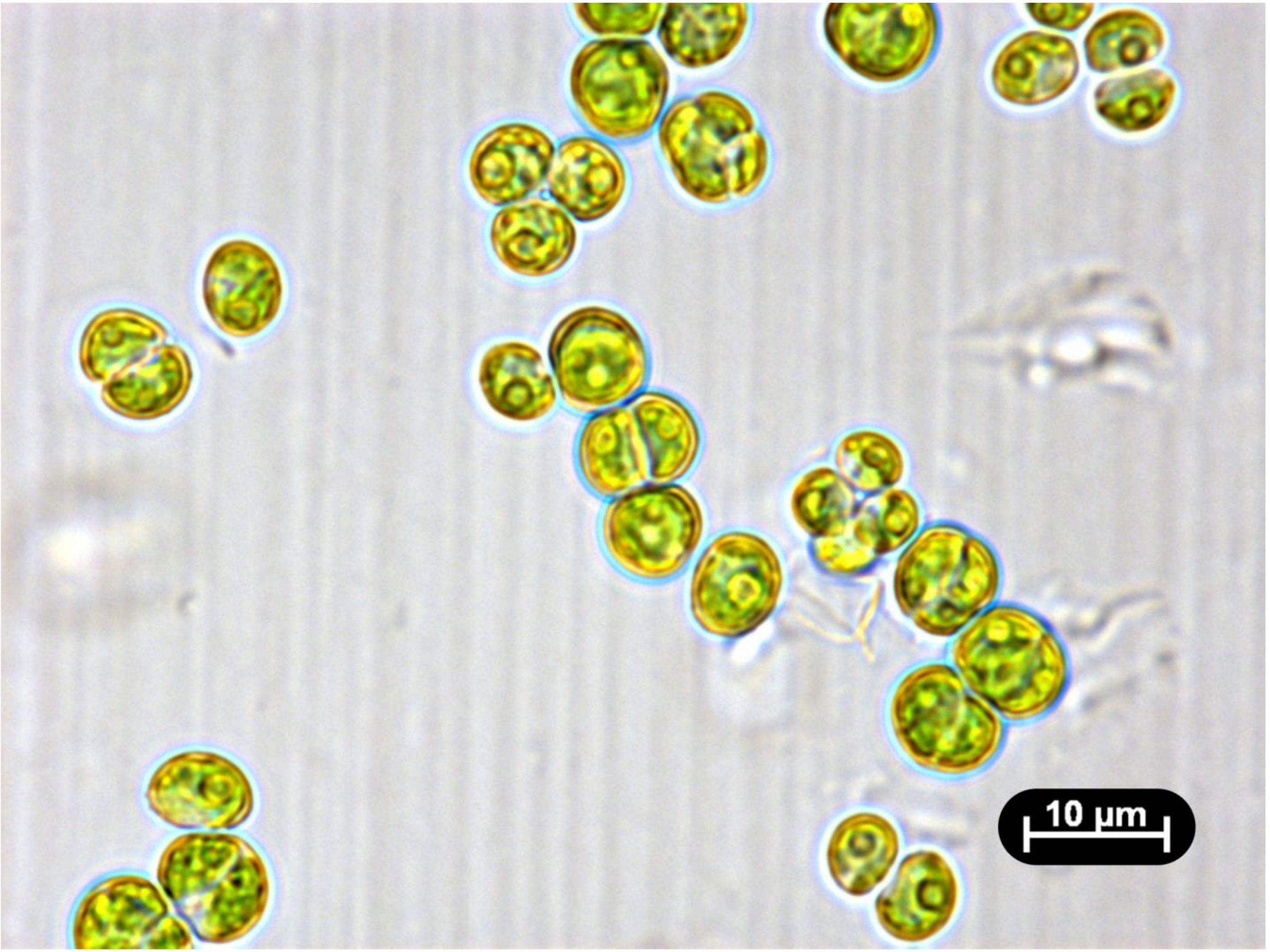

Desmodesmus sp. TCF-81g

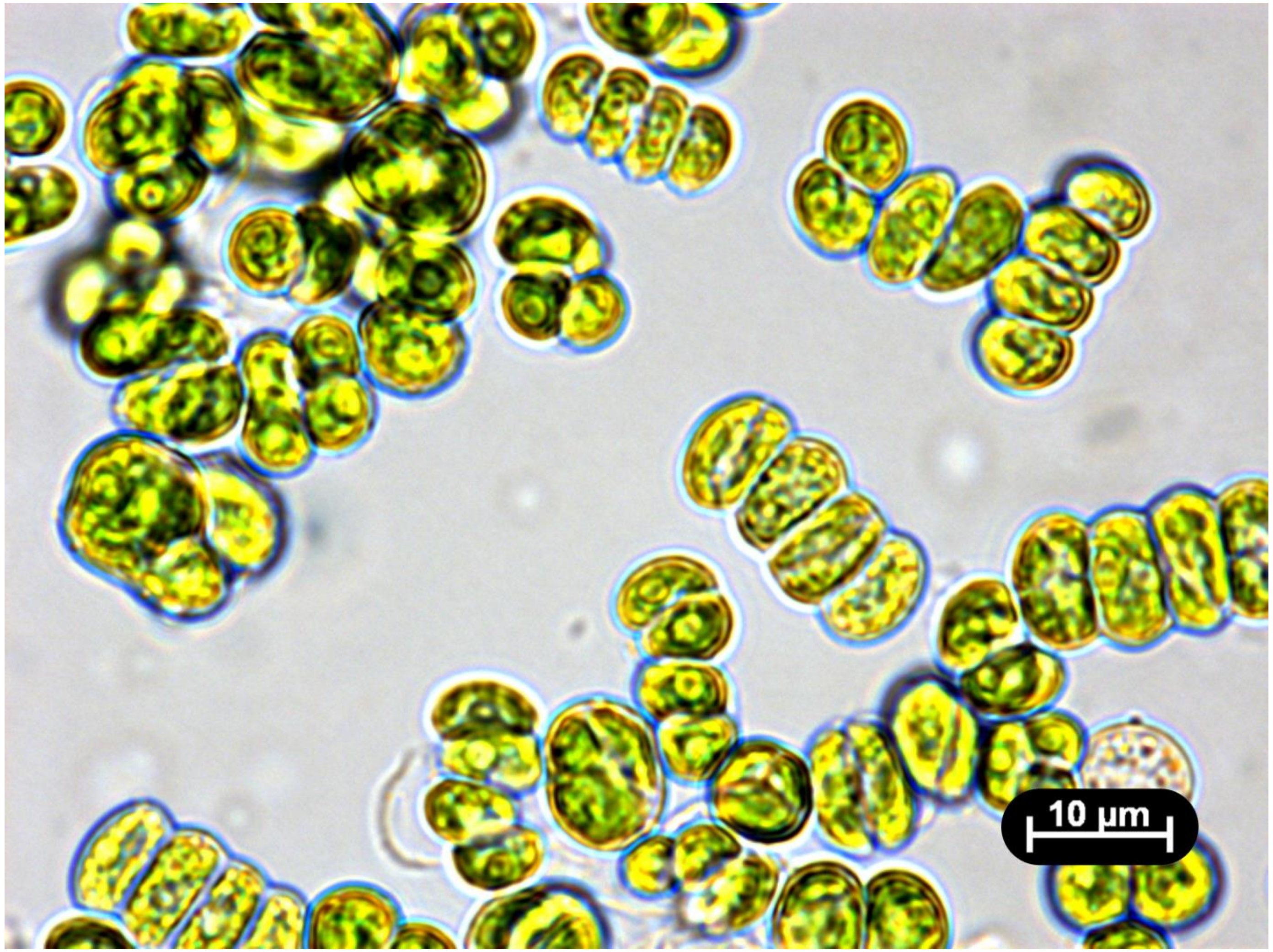

Tetradesmus obliquus TCF-82g

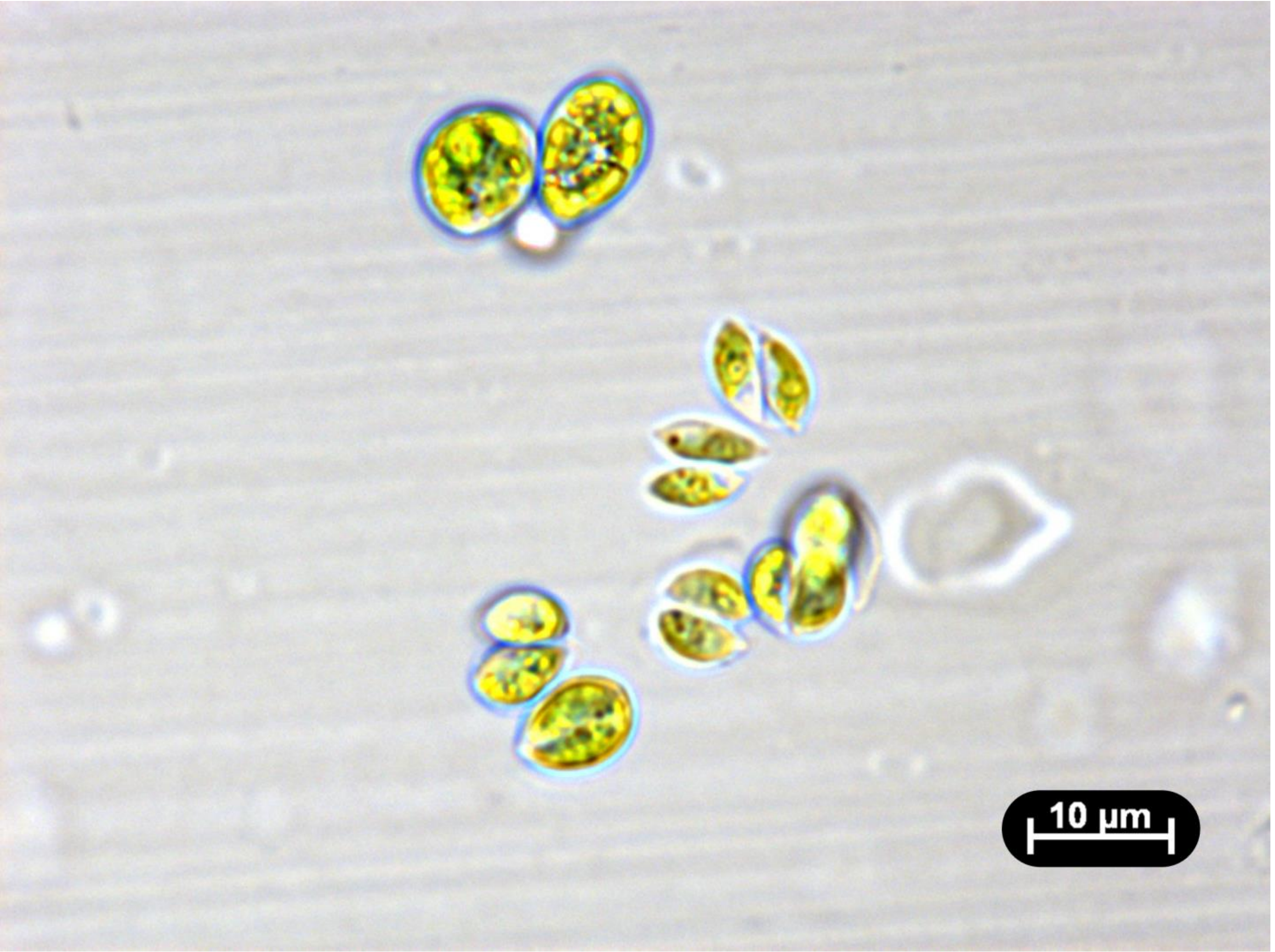

Cyanobacteria

Nostoc edaphicum TCF-1c

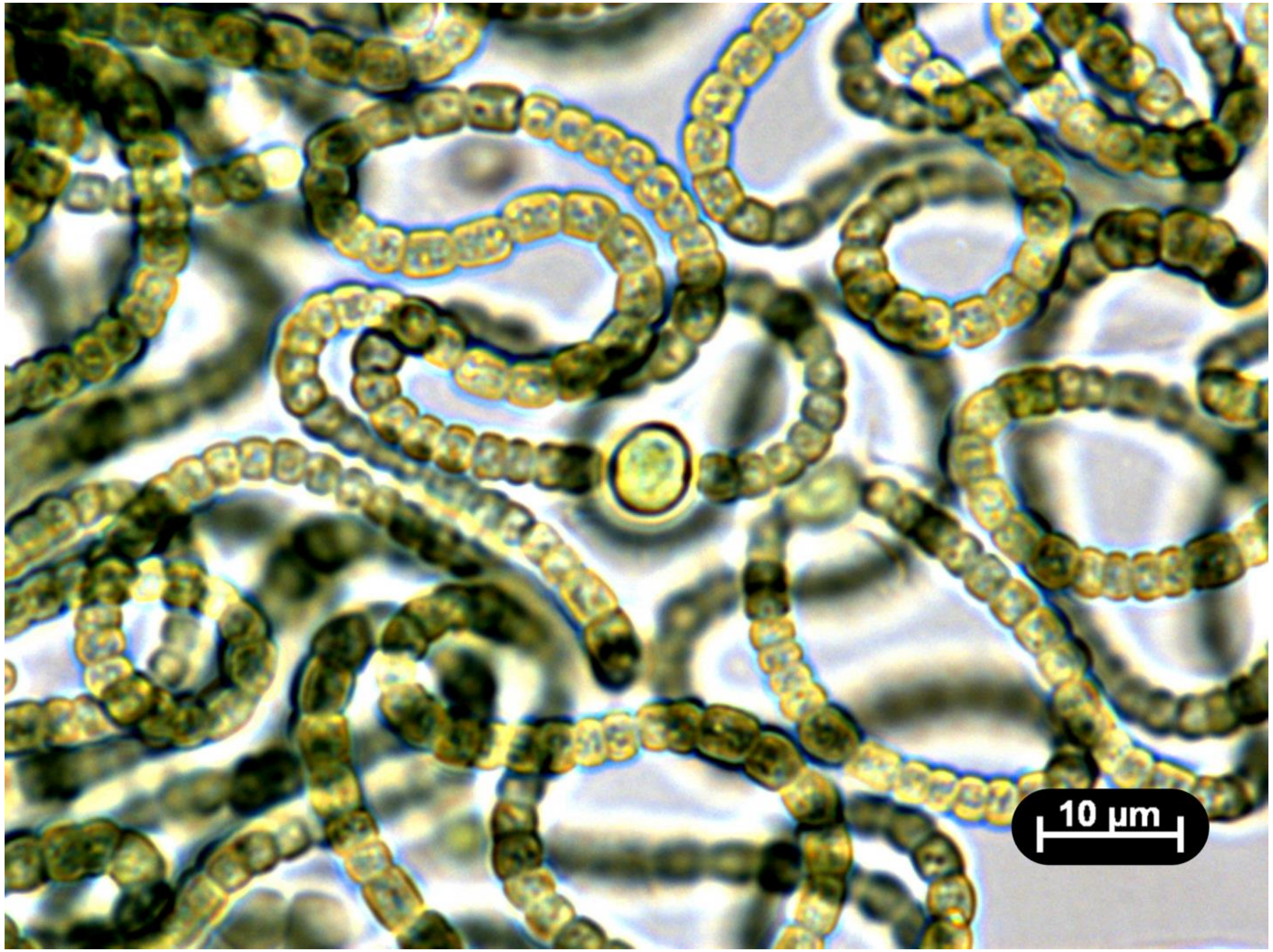

Leptolyngbya sp. TCF-2c

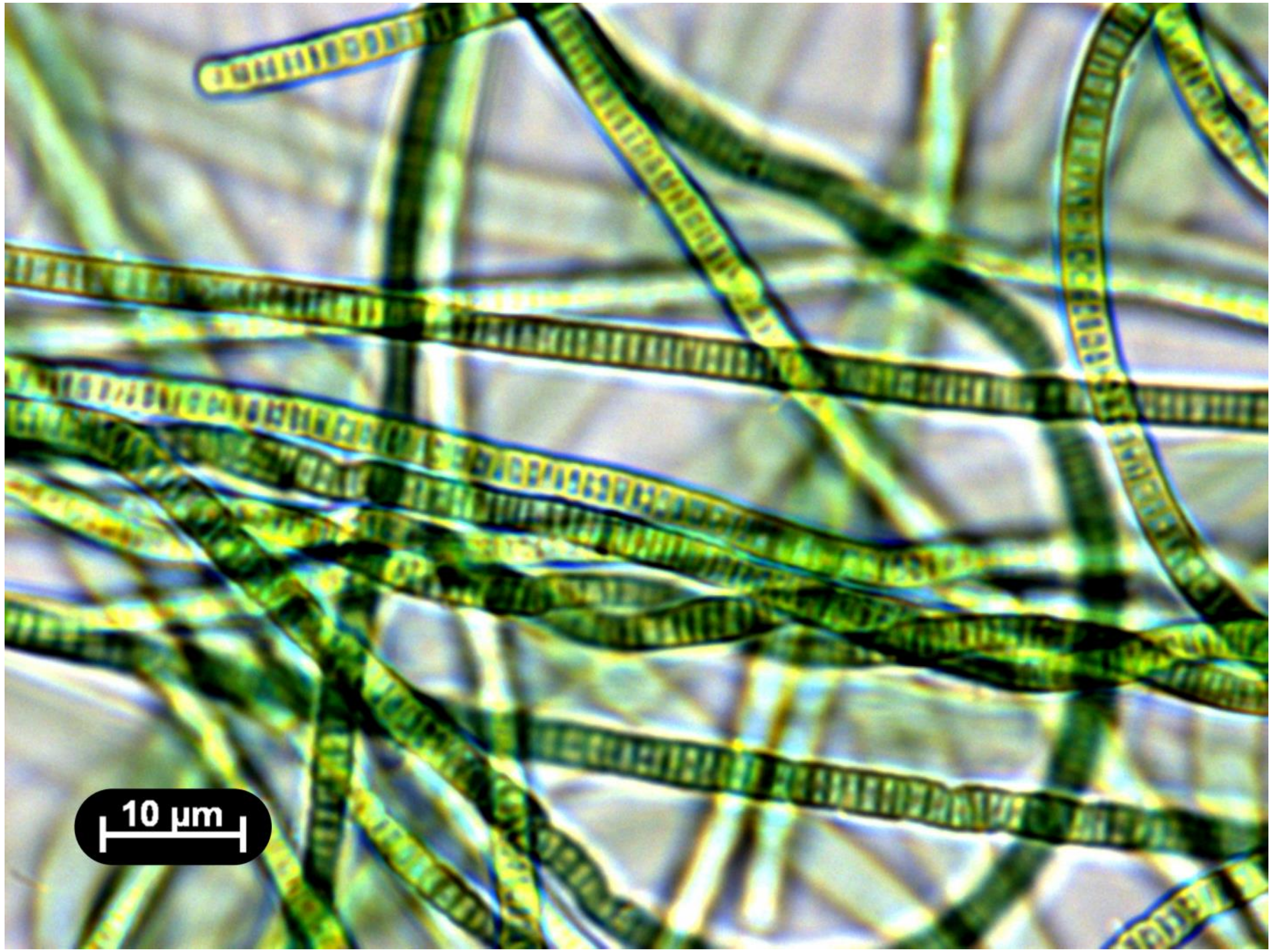

Leptolyngbya sp. TCF-3c

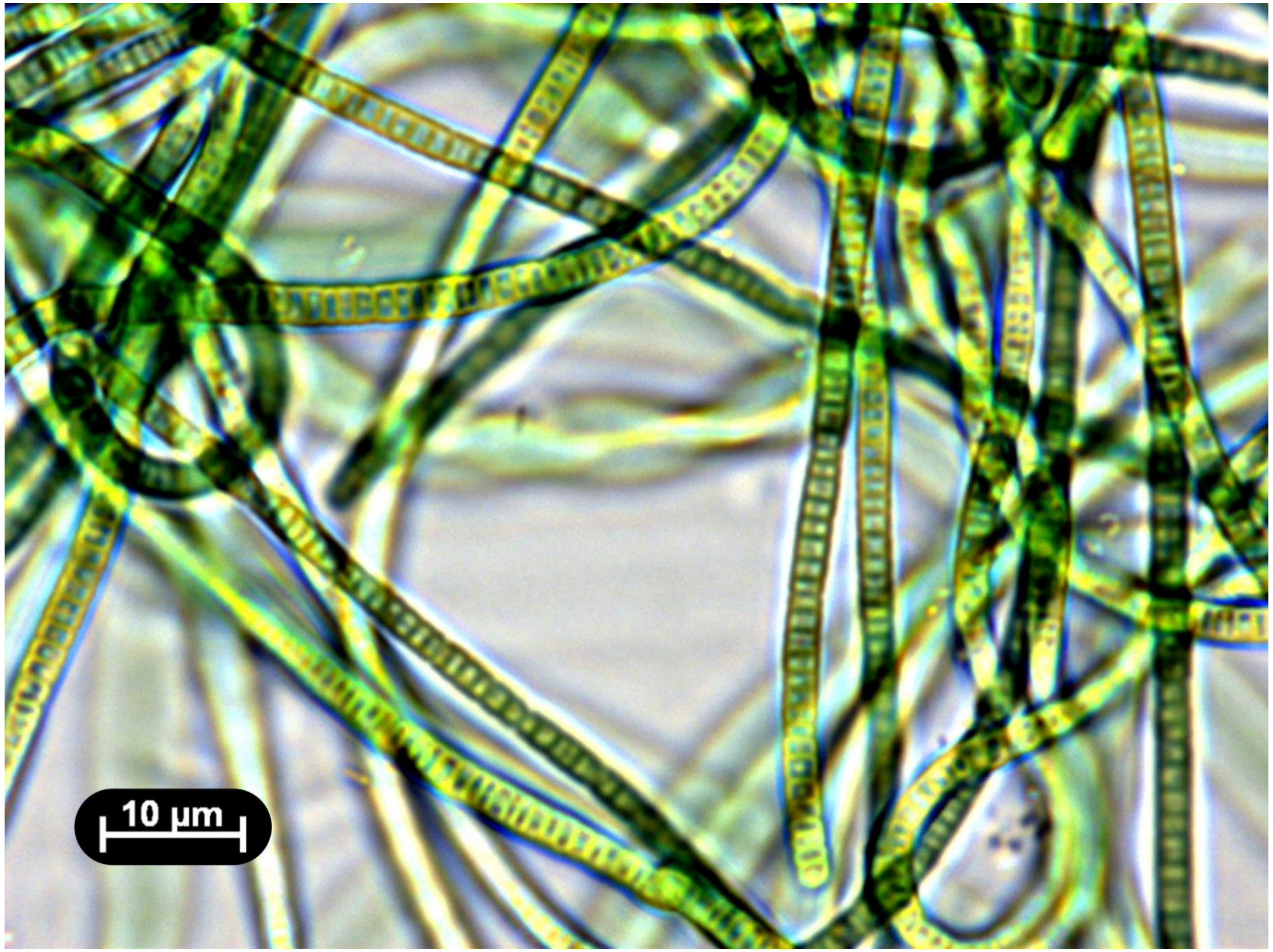

Nodosilinea sp. TCF-4c

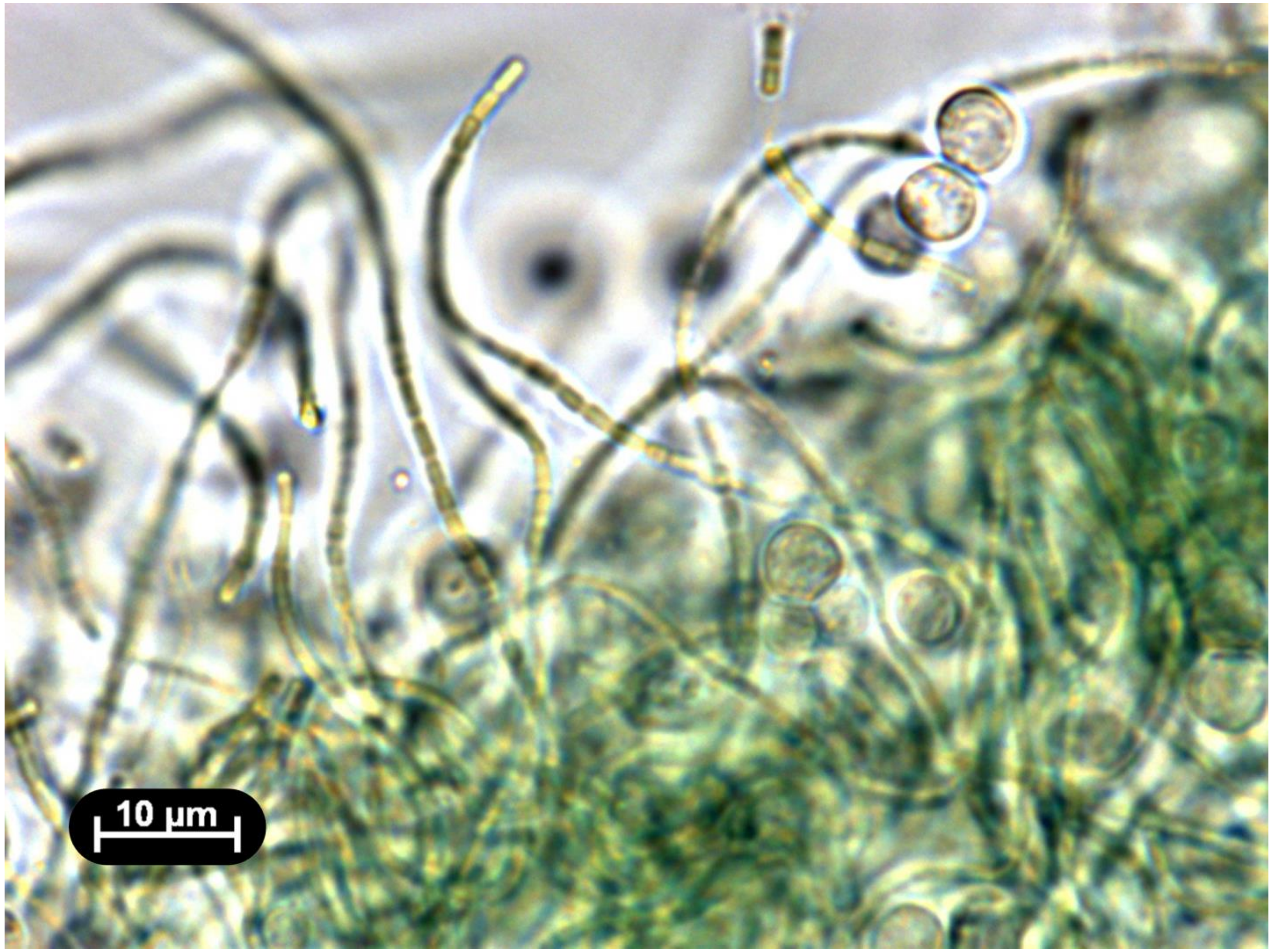

Synechocystis sp. TCF-5c

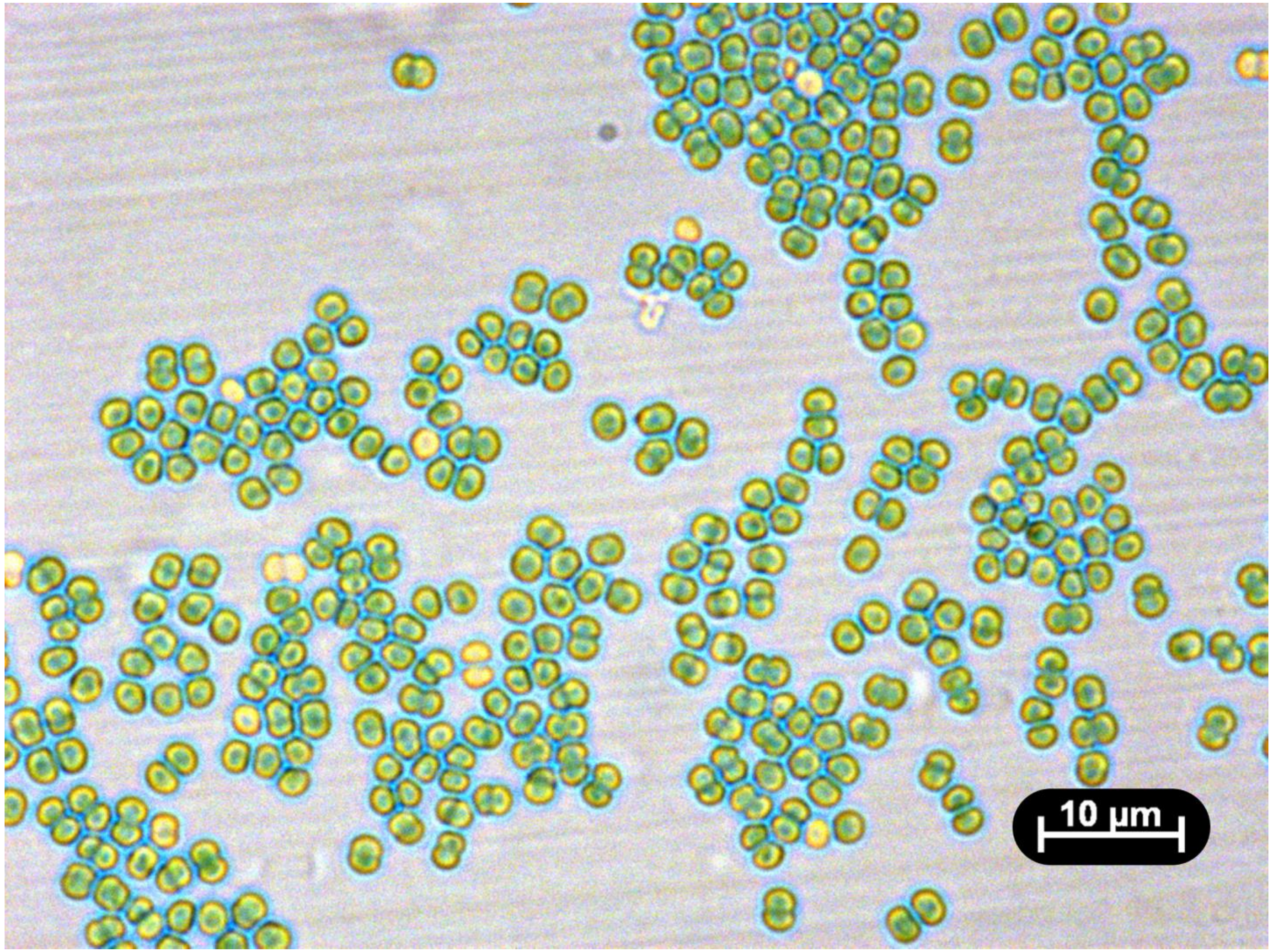

Leptolyngbya sp. TCF-6c

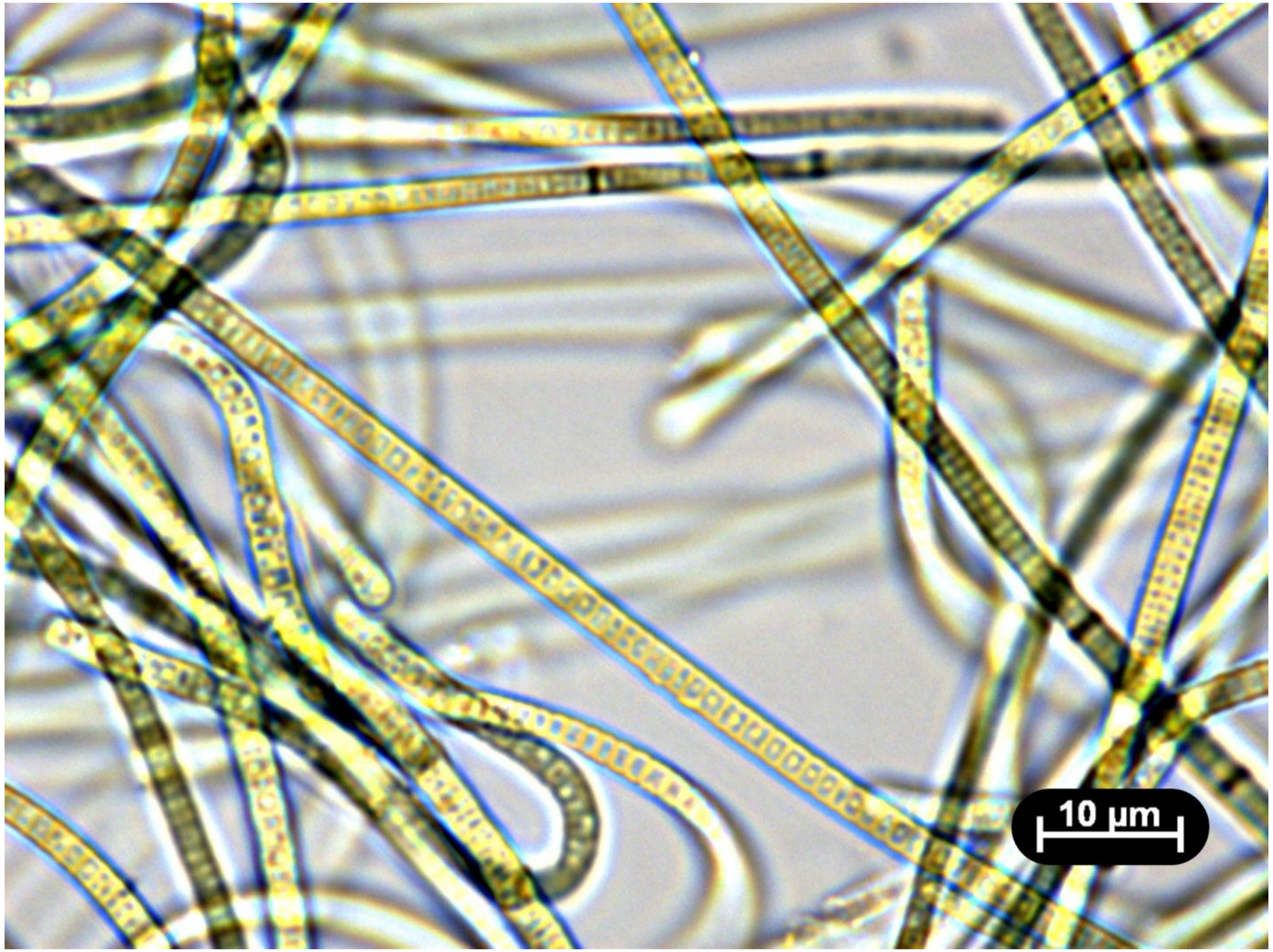

Leptolyngbya sp. TCF-7c

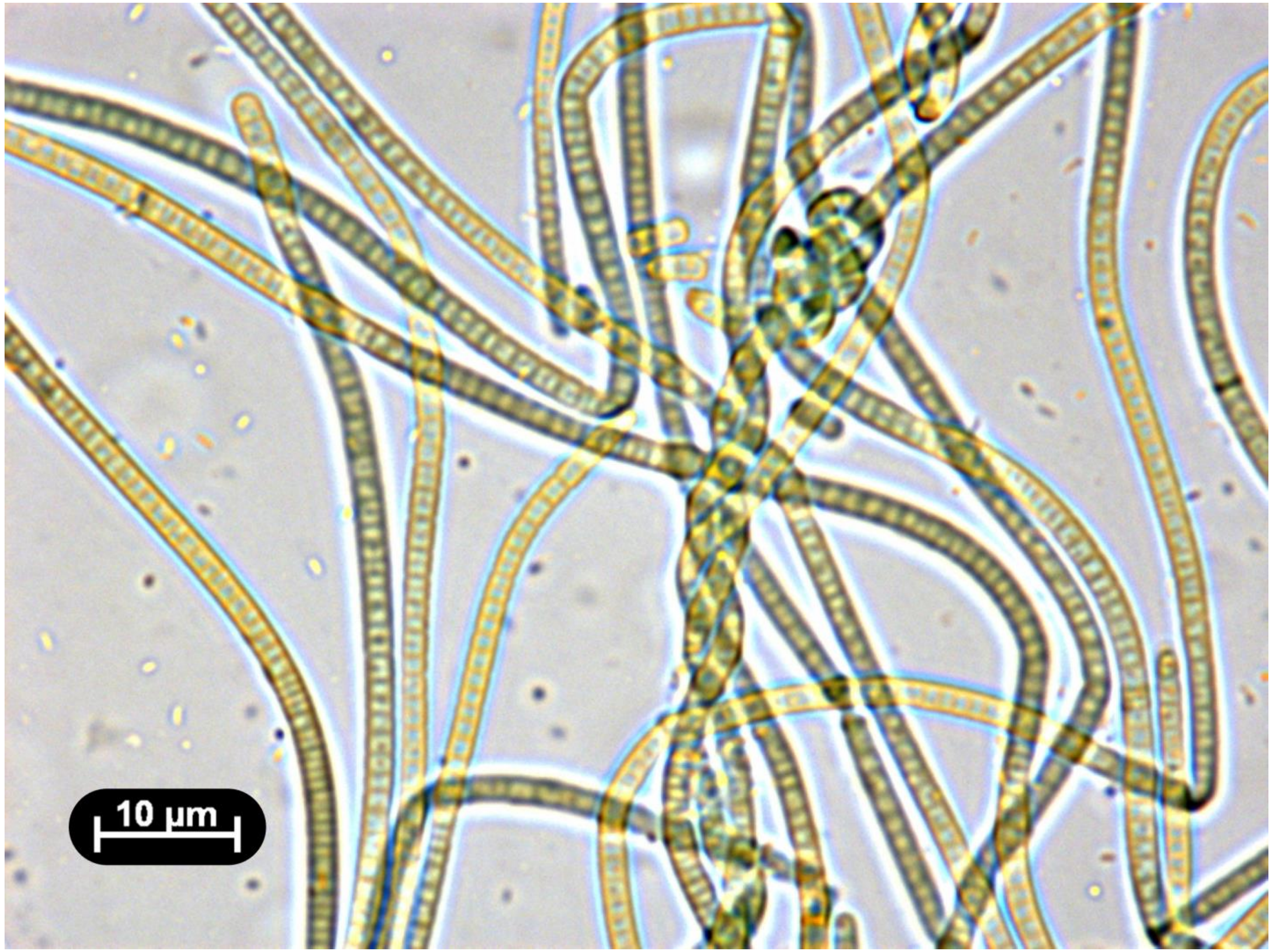

Leptolyngbya sp. TCF-8c

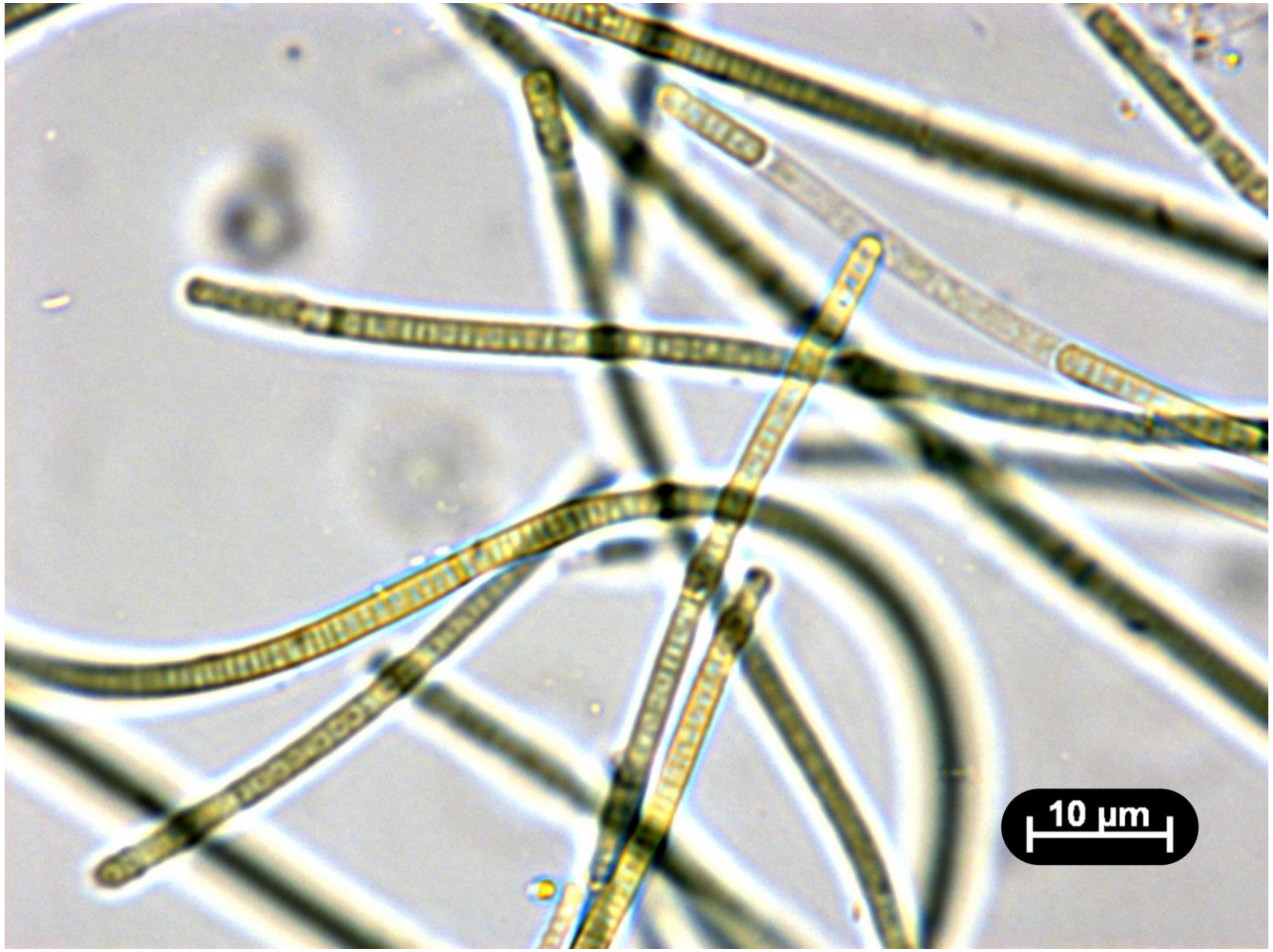

Pseudanabaena sp. TCF-9c

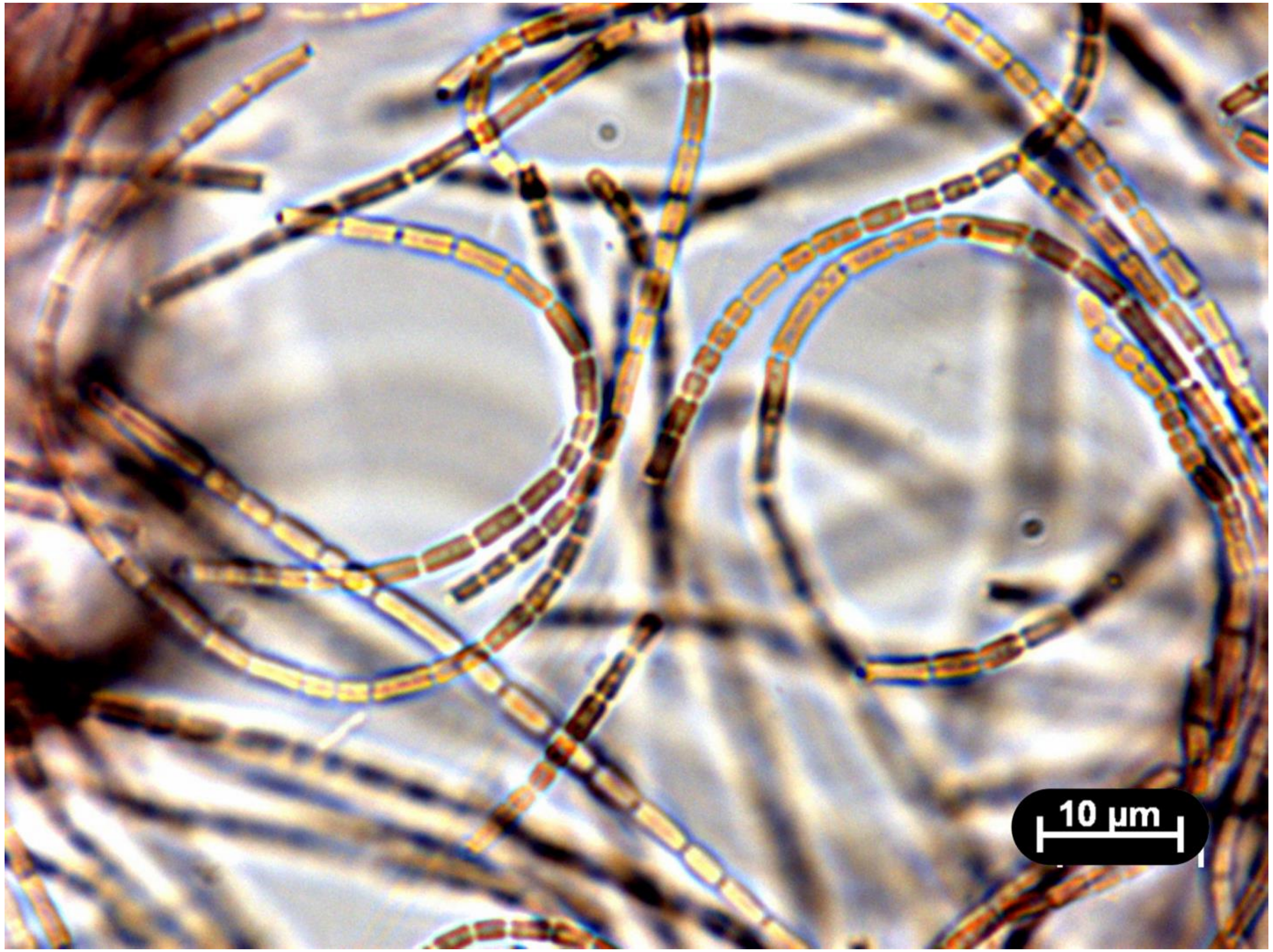

Nodosilinea sp. TCF-10c

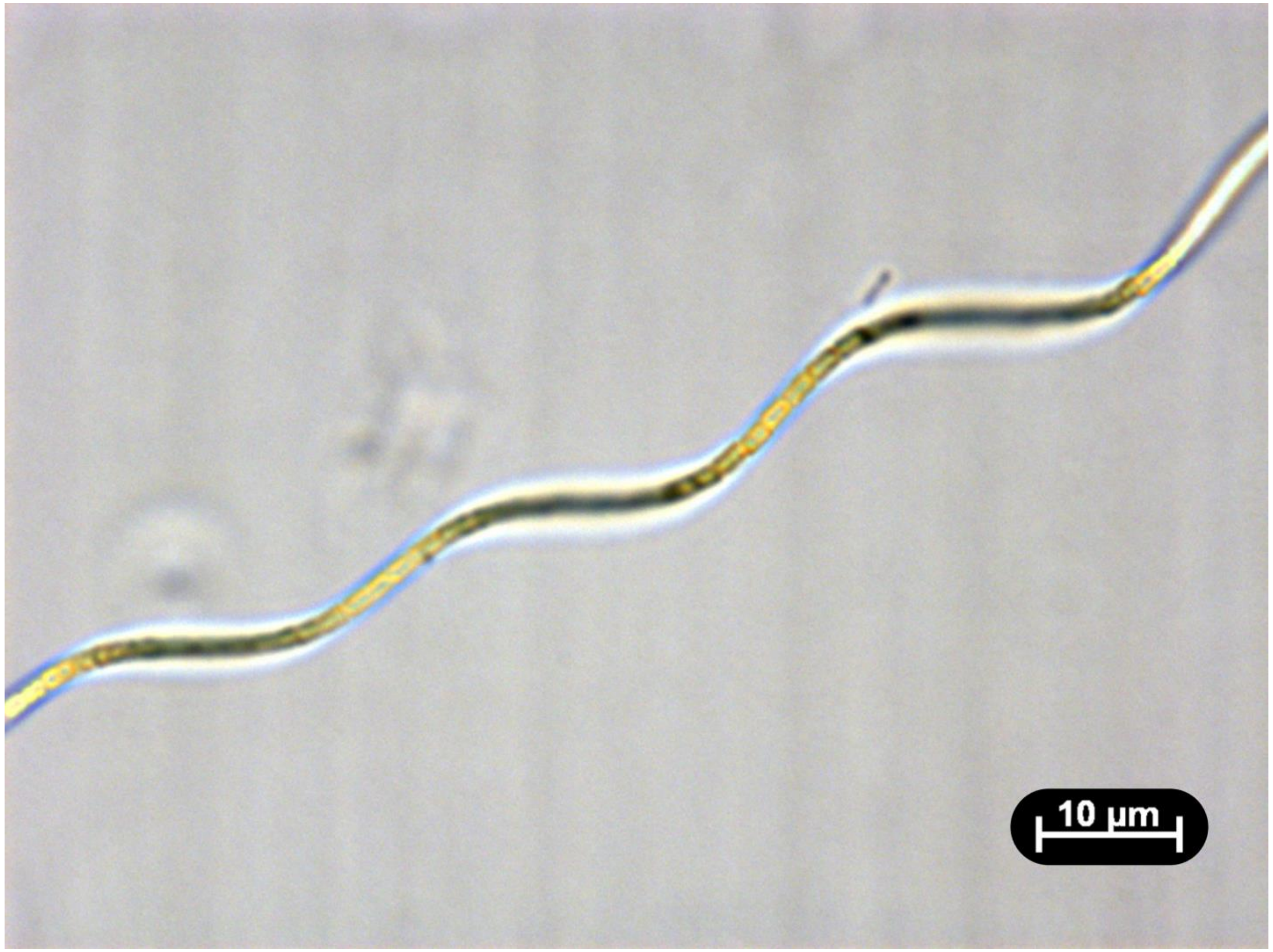

Diatoms

Craticula molestiformis TCF-1d

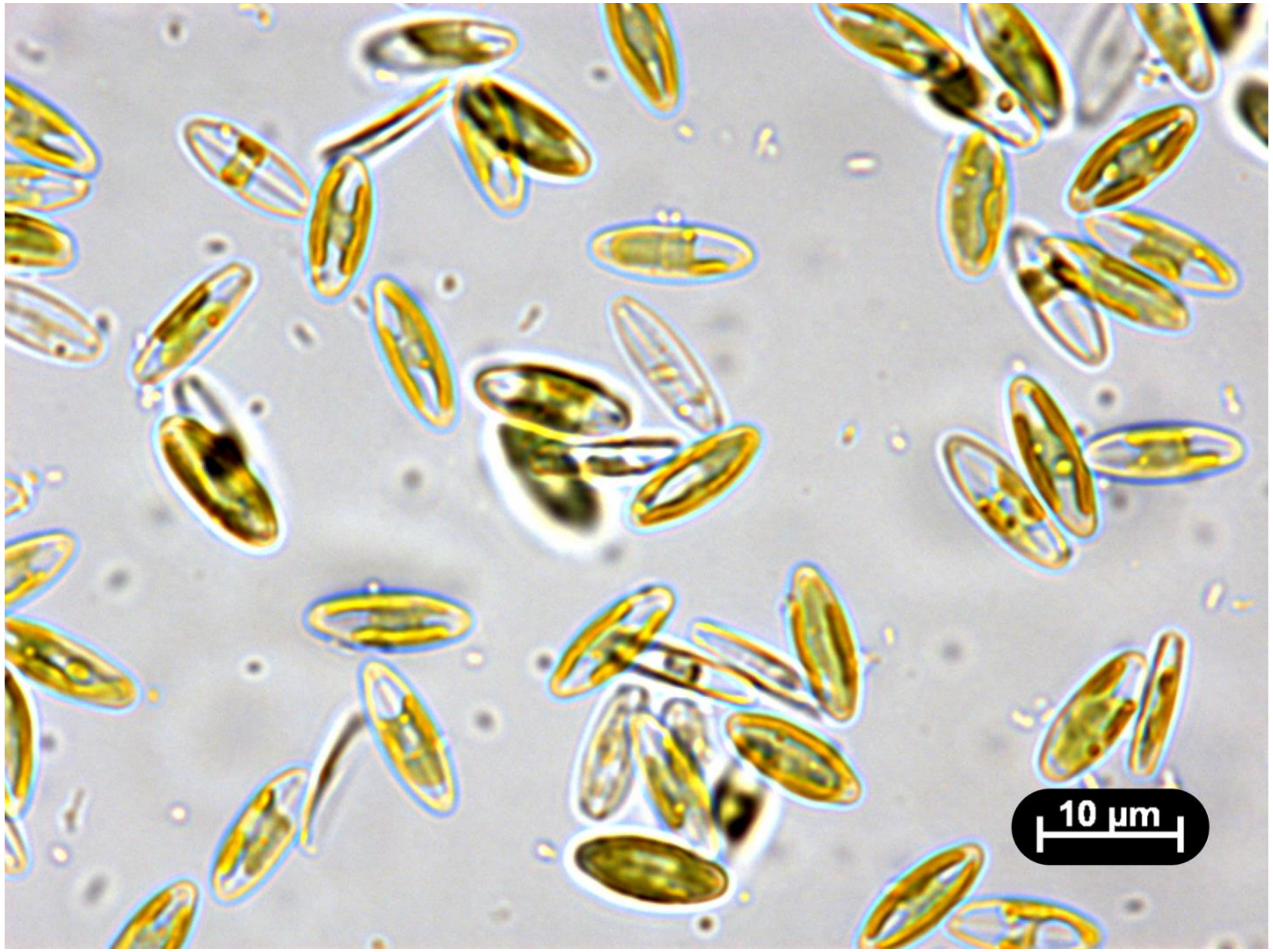

Craticula molestiformis TCF-2d

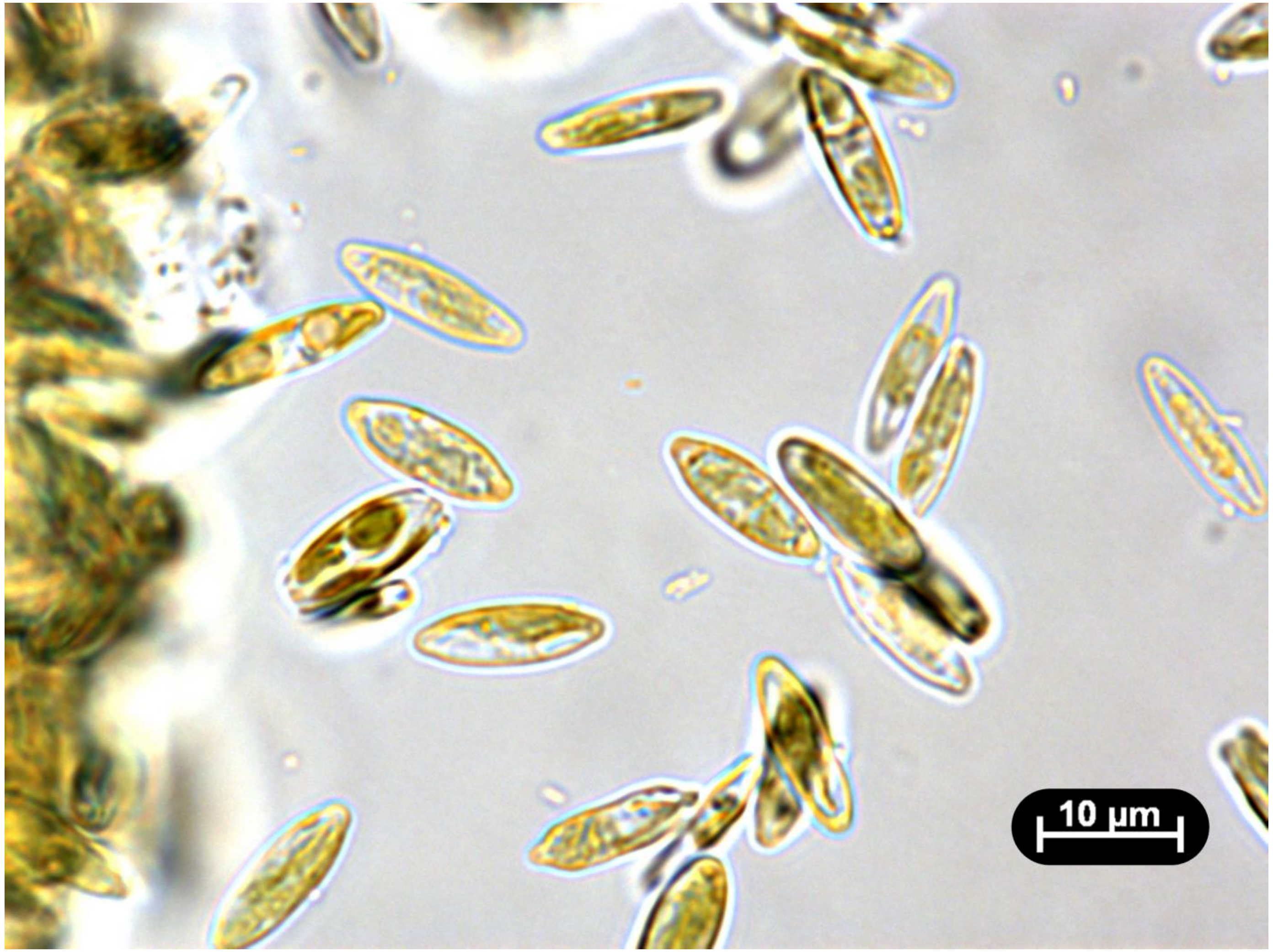

Eolimna minima TCF-3d

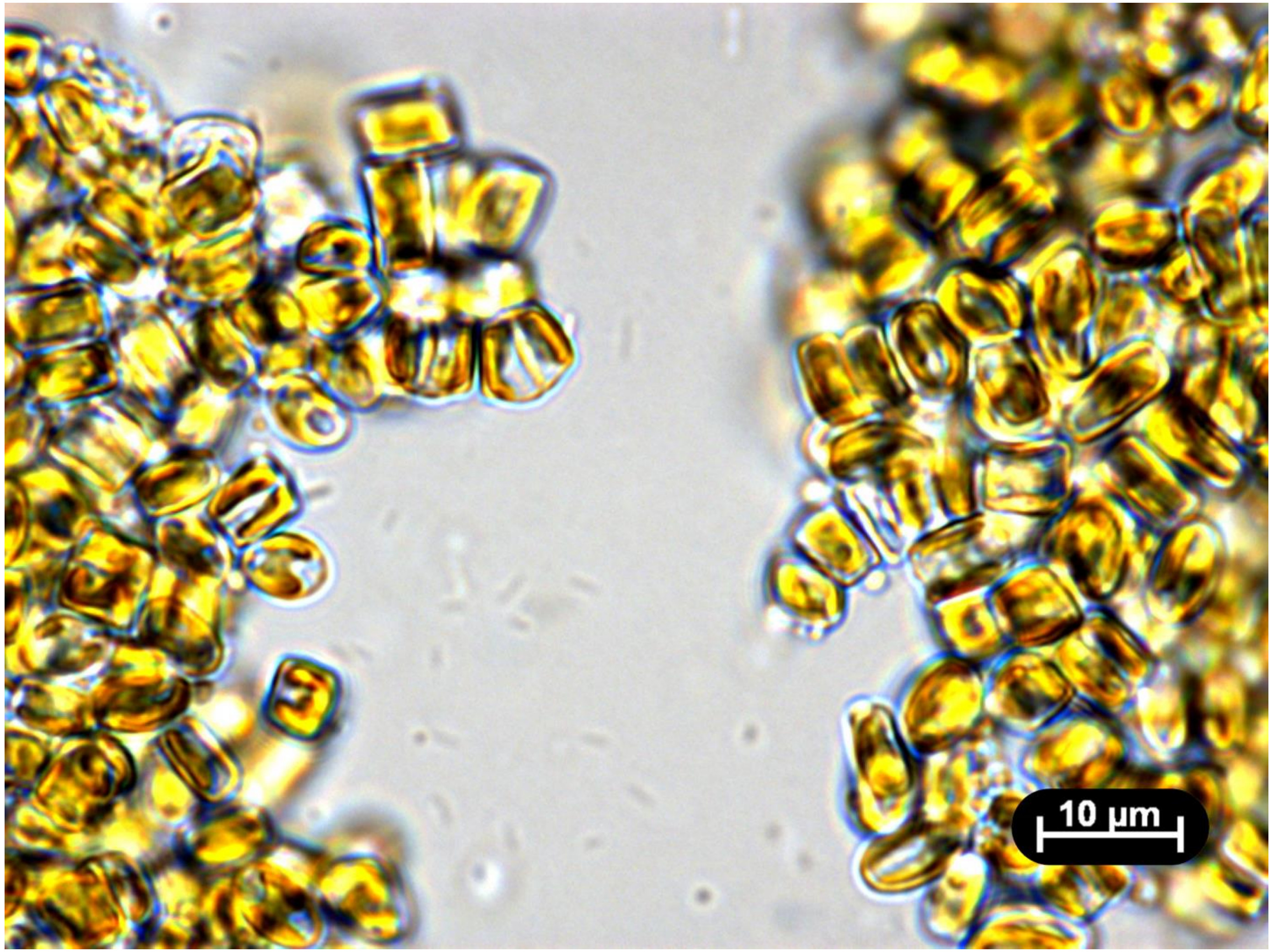

Nitzschia palea TCF-4d

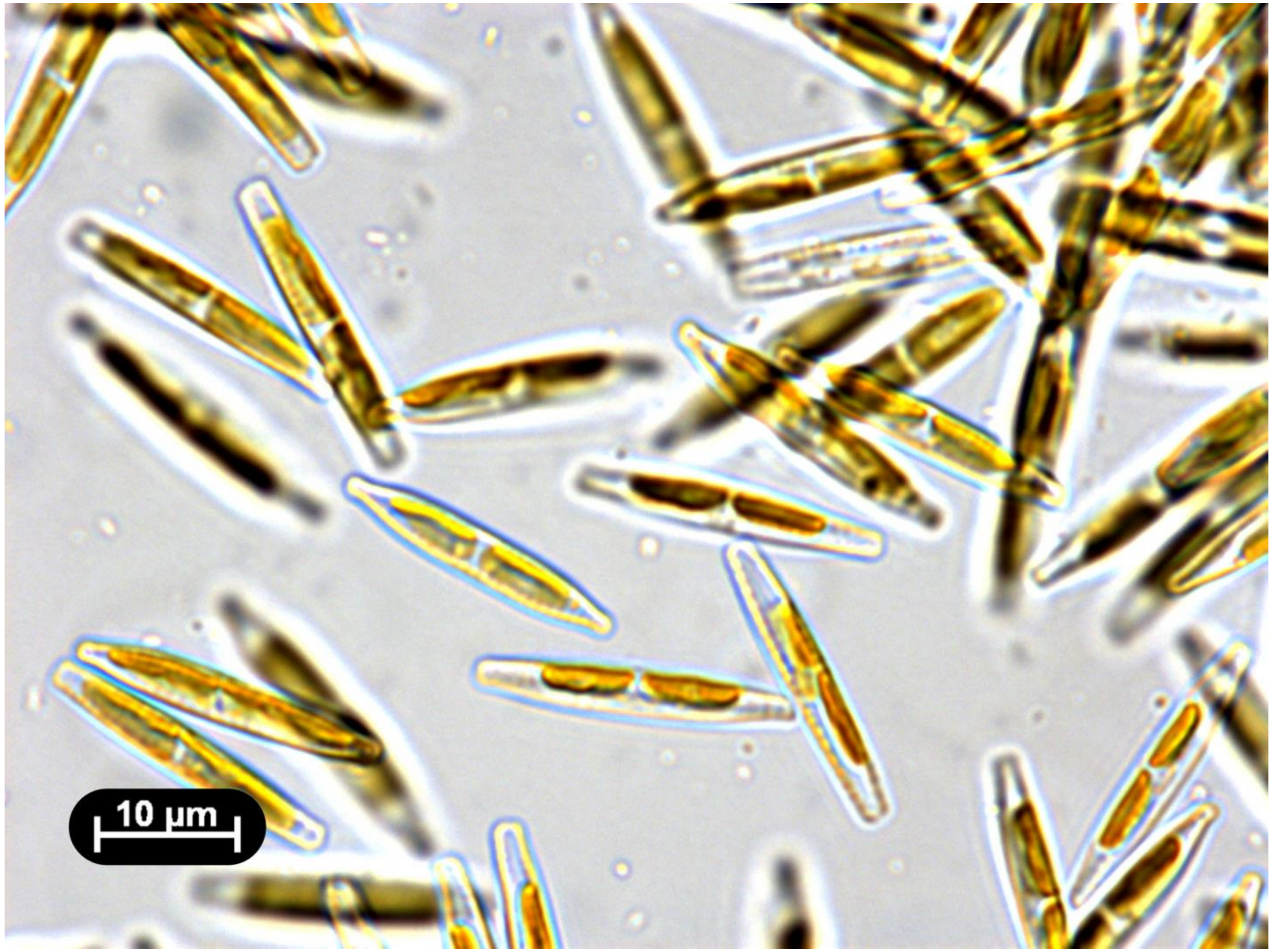

Nitzschia sp. TCF-5d

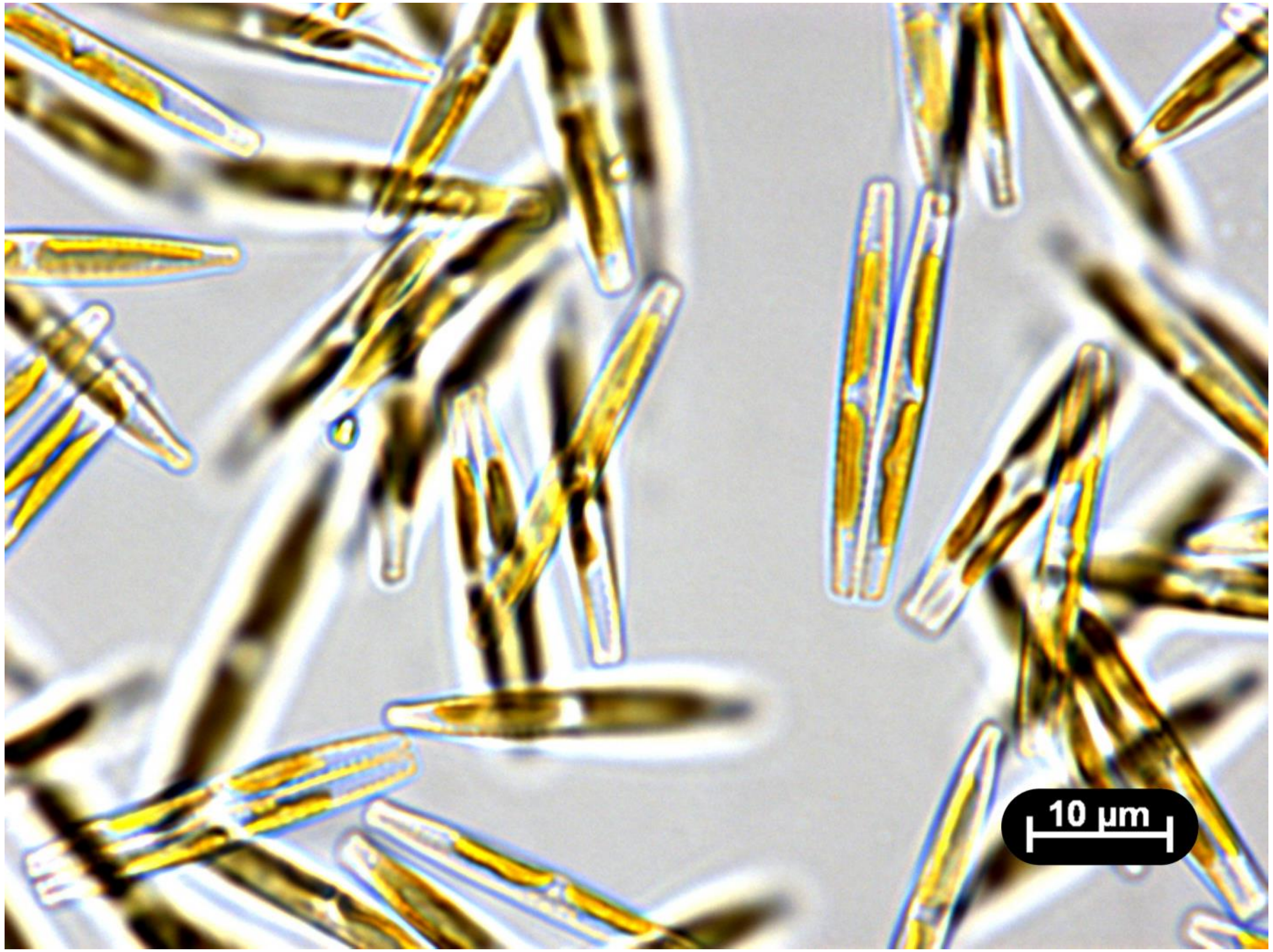

Craticula molestiformis TCF-6d

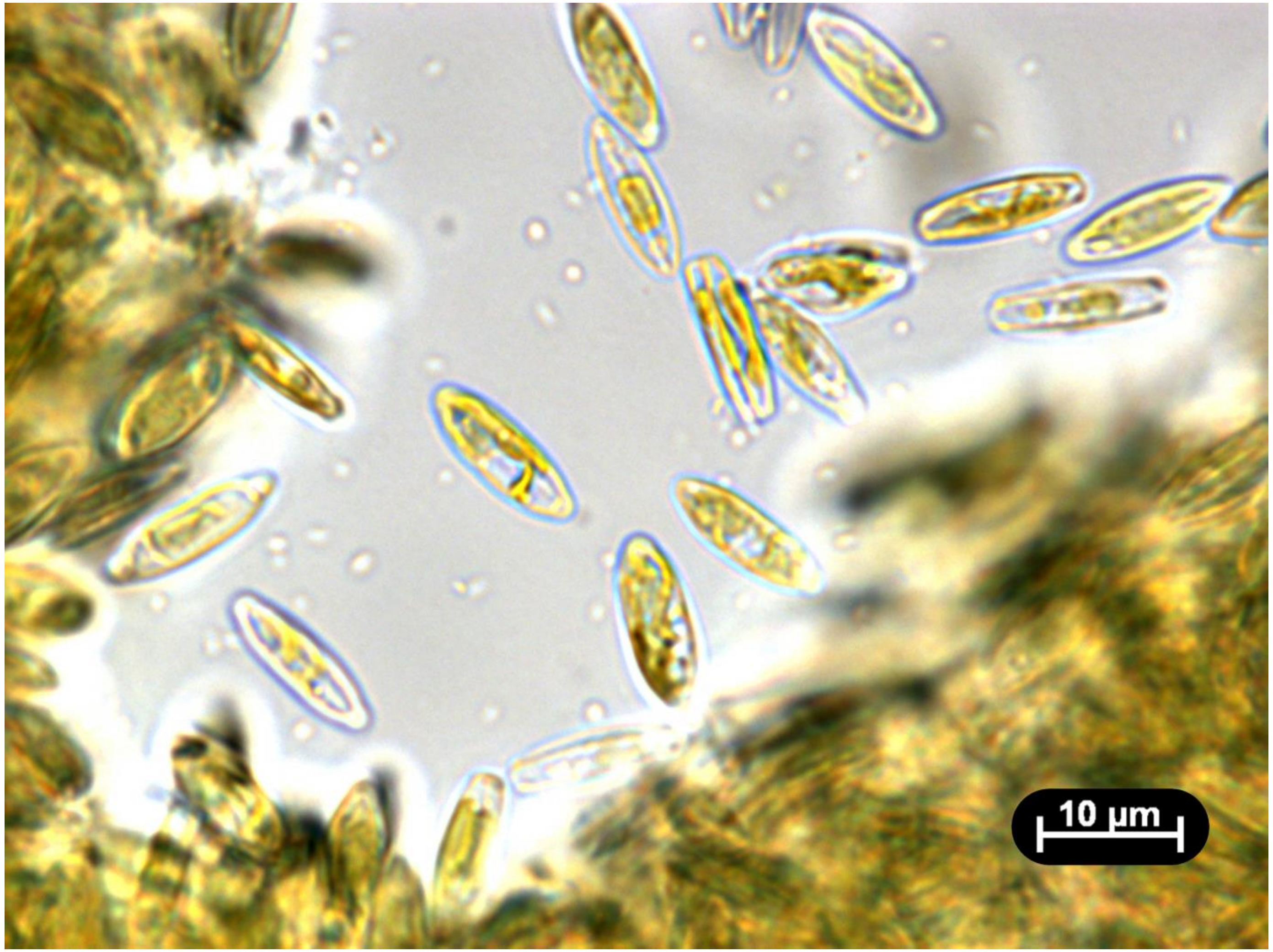

Craticula molestiformis TCF-7d

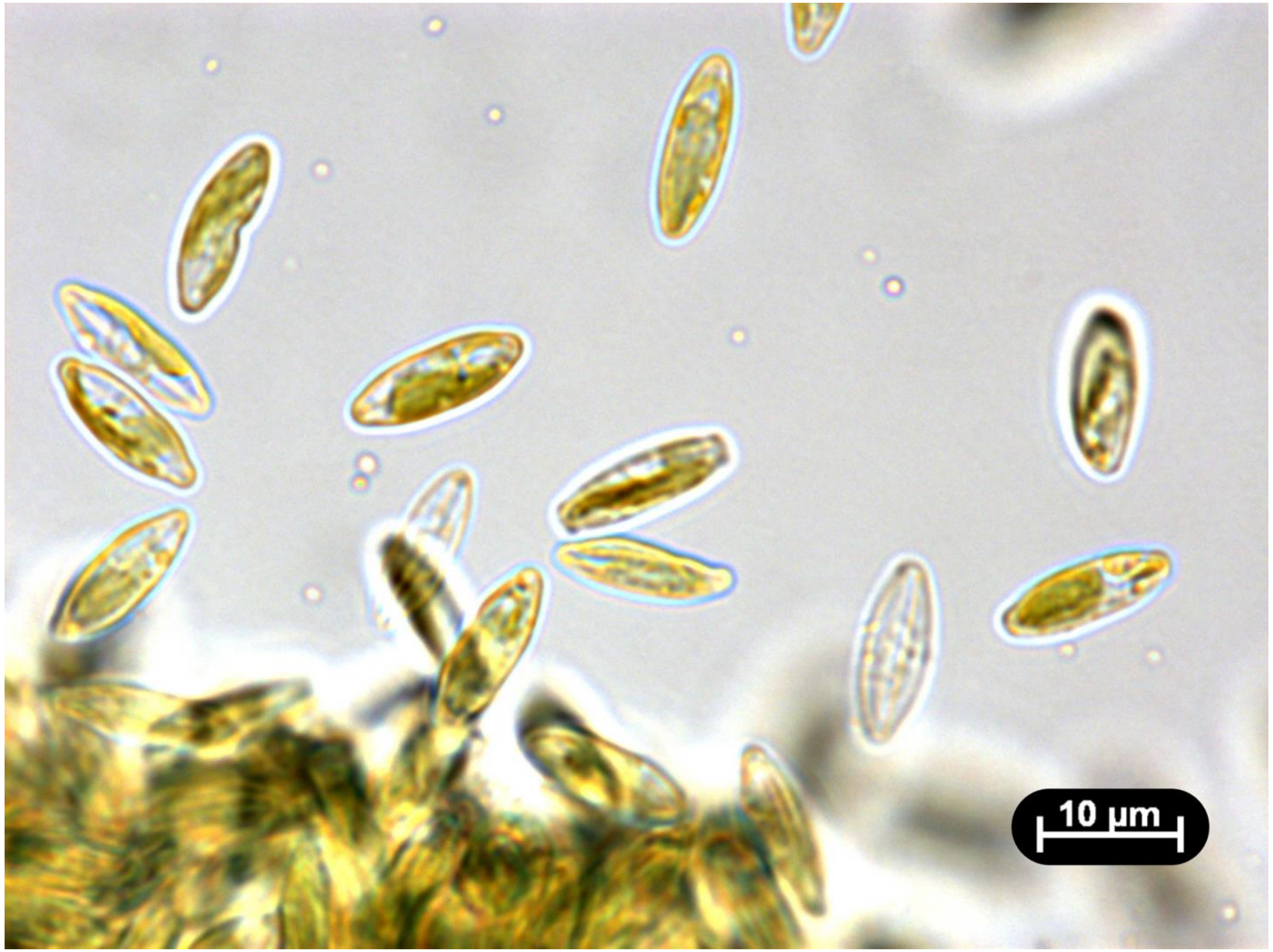

Craticula molestiformis TCF-8d

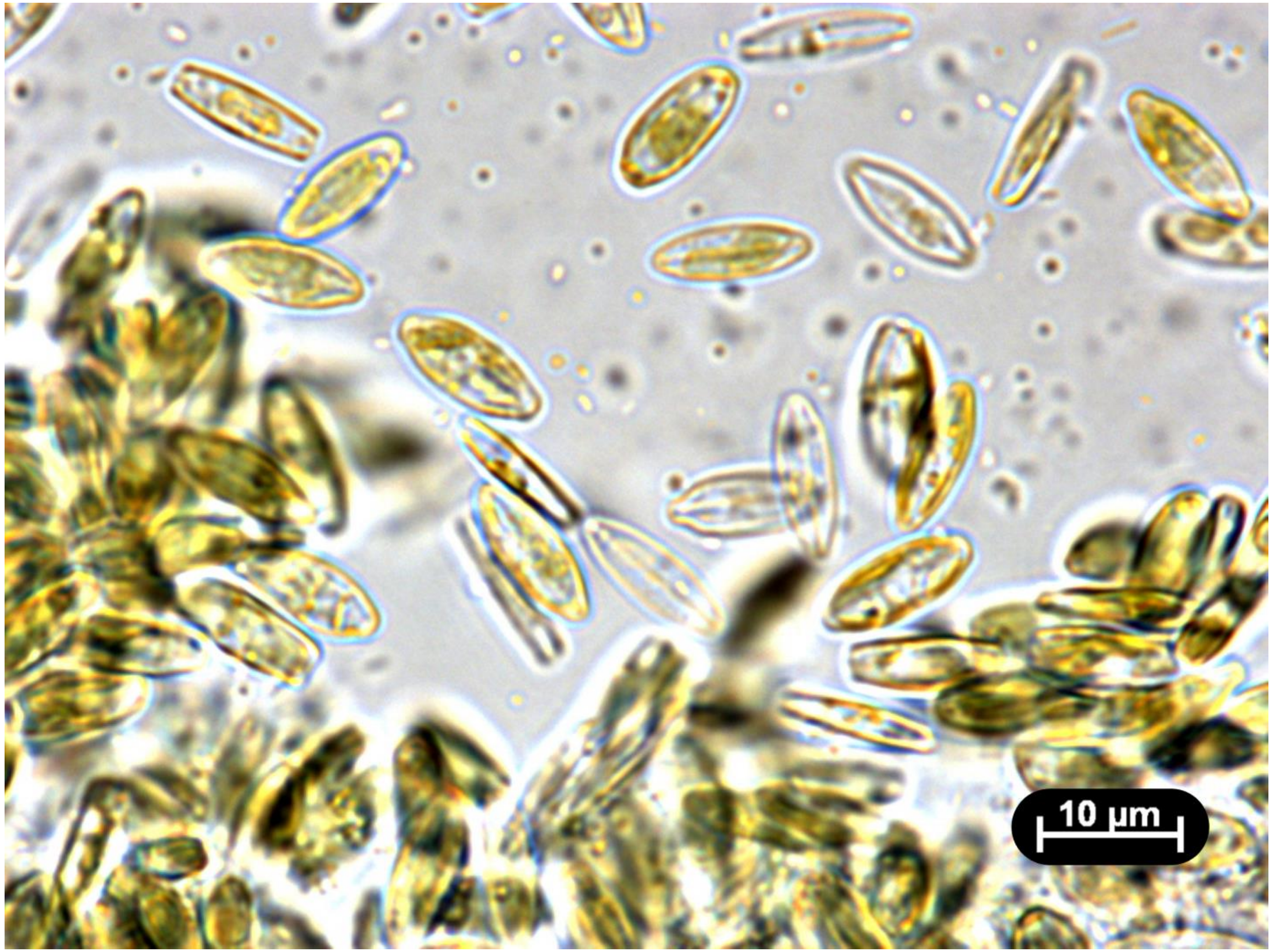

Nitzschia palea TCF-9d

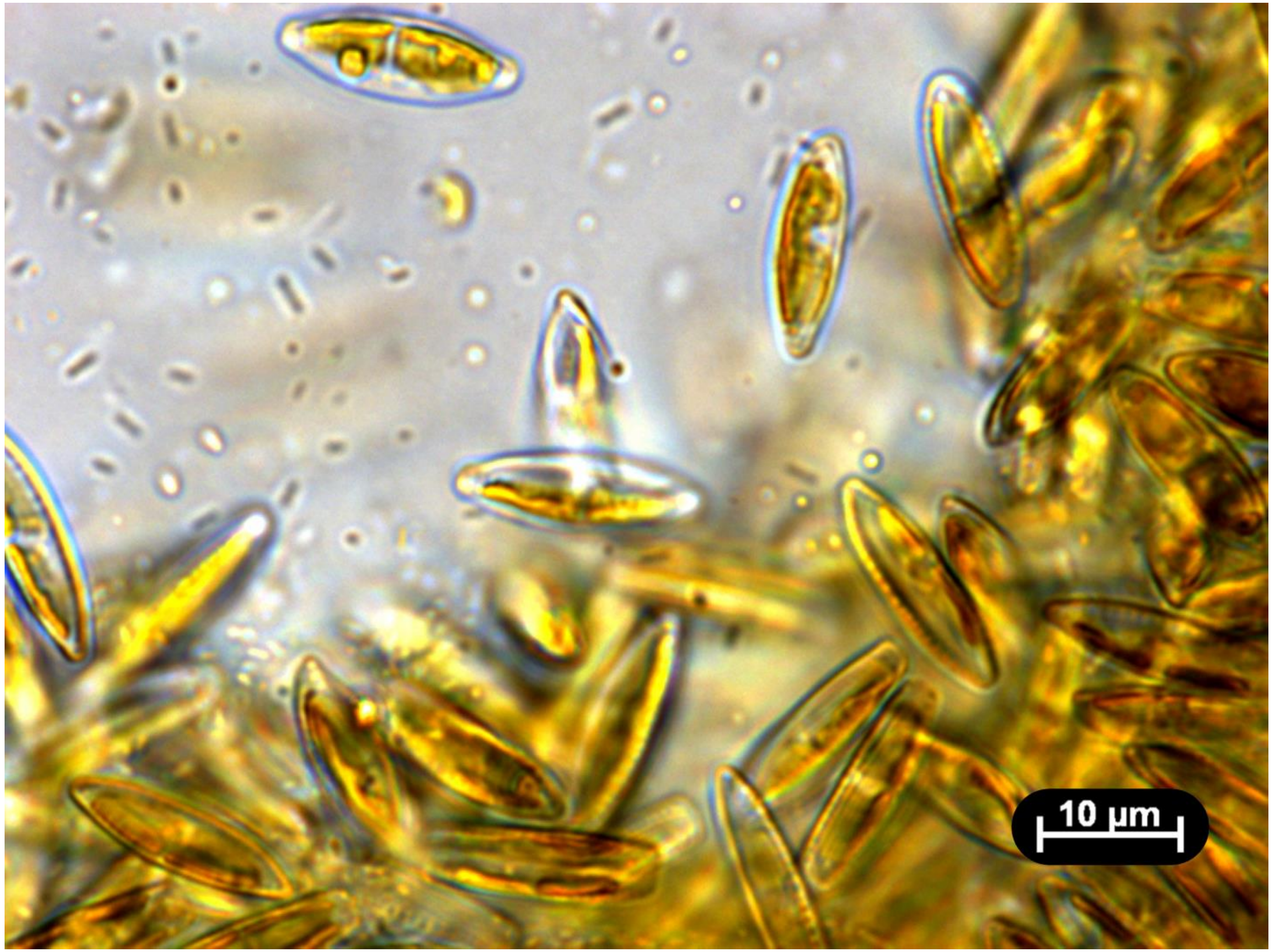

RAB Microalgae Consortium from Slater, Iowa

RAB Microalgae Consortium

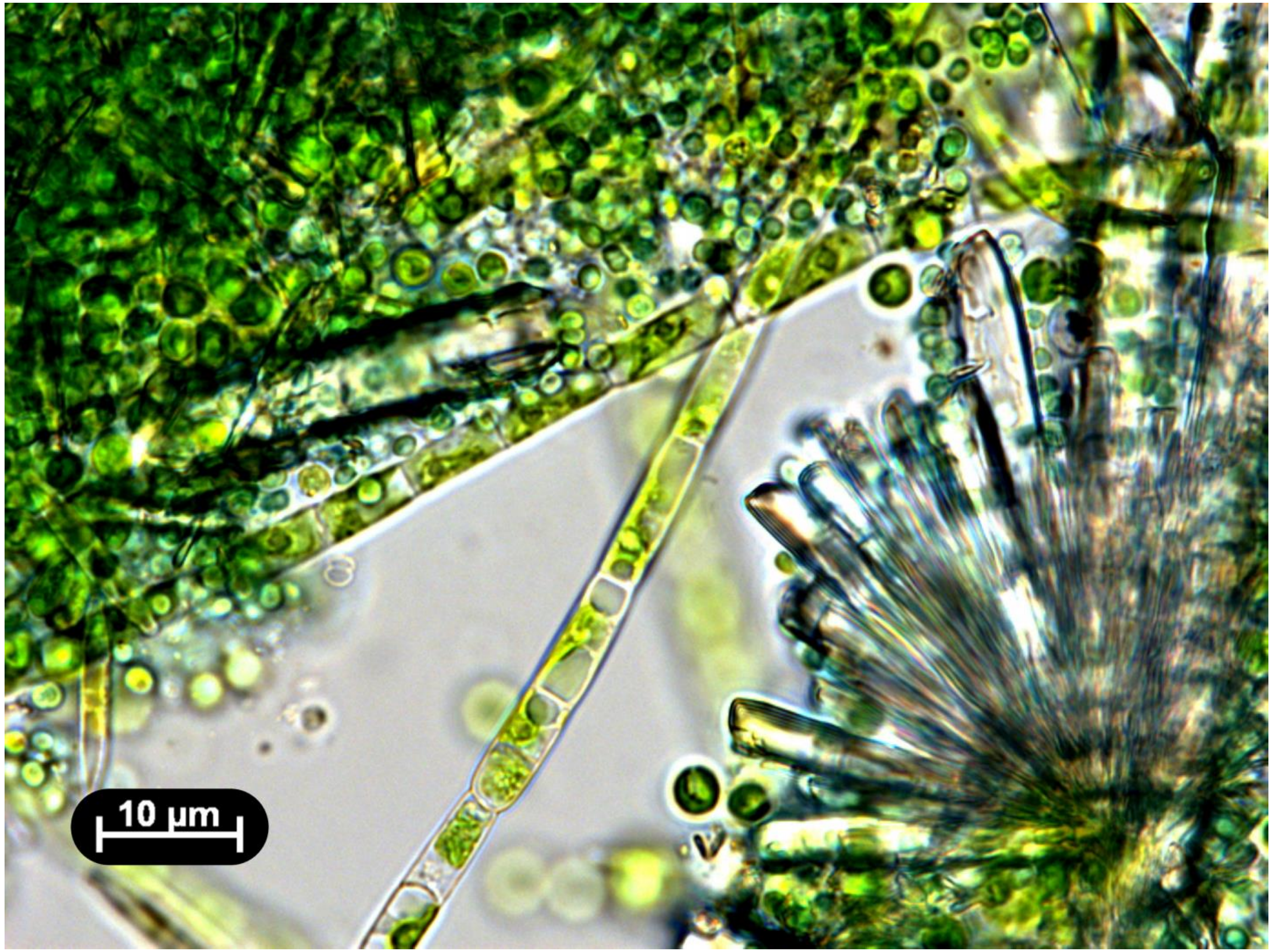

(**Image 1/7** of RAB microalgae consortium used in this study cultured in BBM medium)

RAB Microalgae Consortium

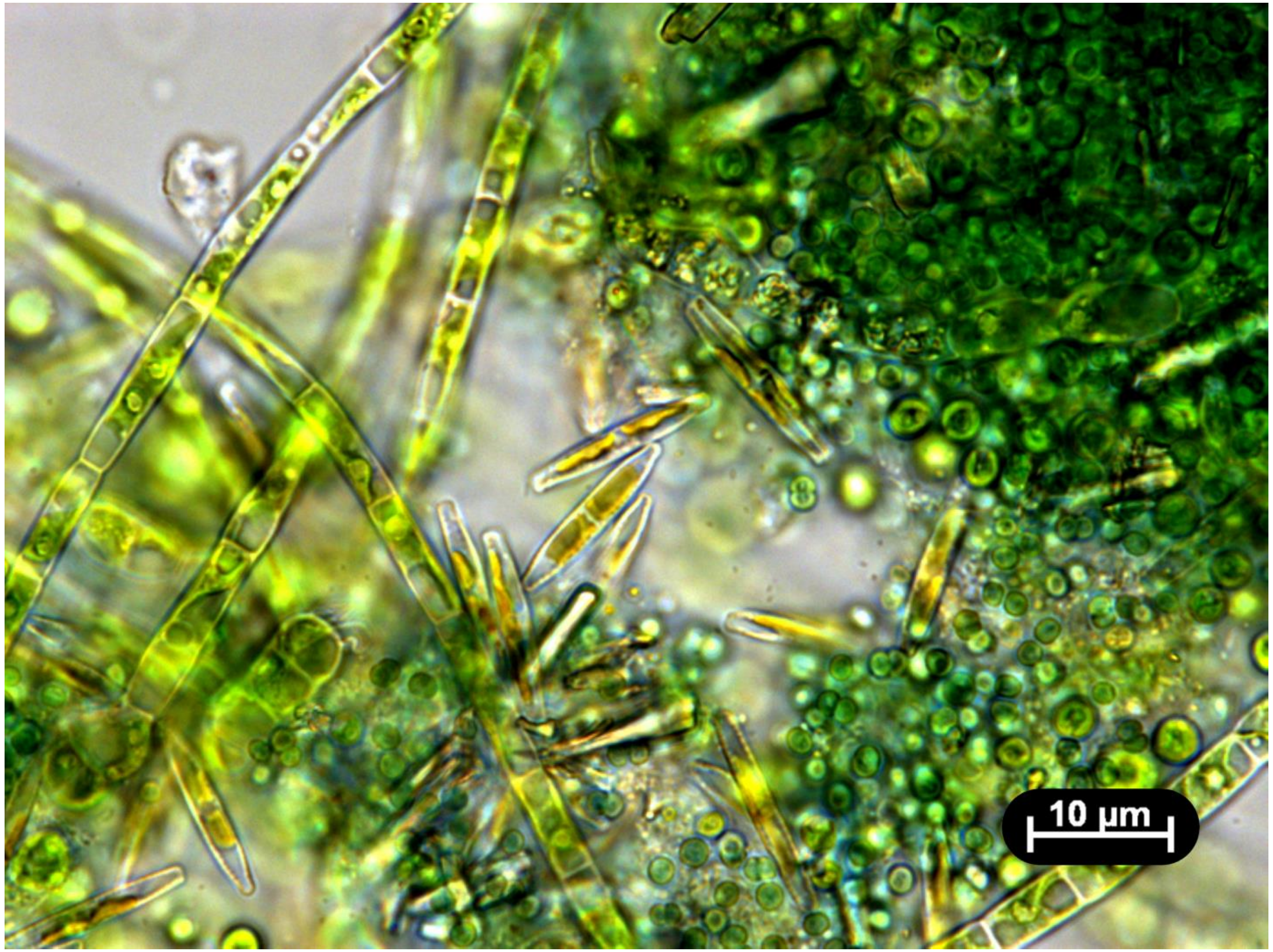

(**Image 2/7** of RAB microalgae consortium used in this study cultured in BBM medium)

RAB Microalgae Consortium

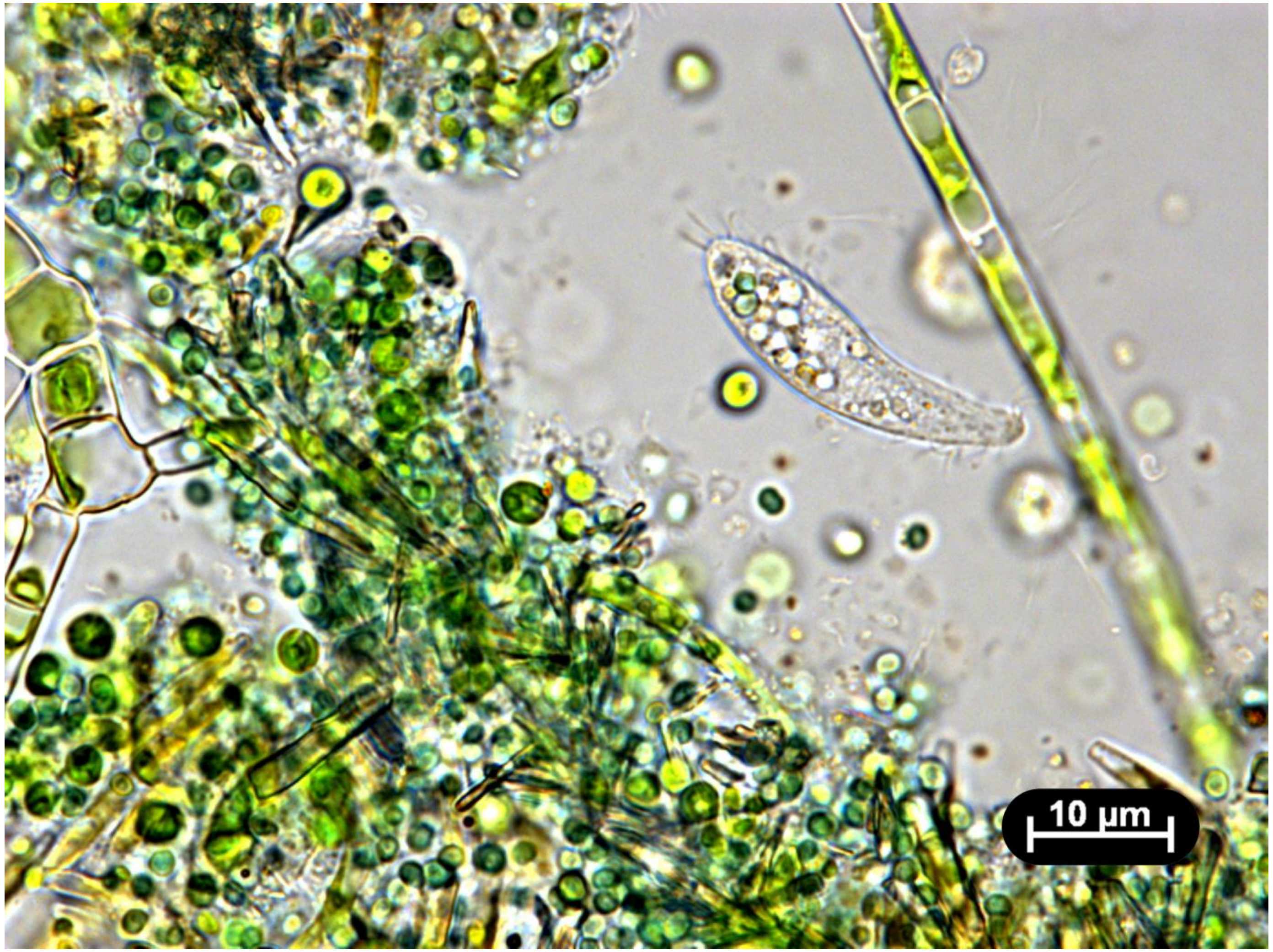

(**Image 3/7** of RAB microalgae consortium used in this study cultured in BBM medium)

RAB Microalgae Consortium

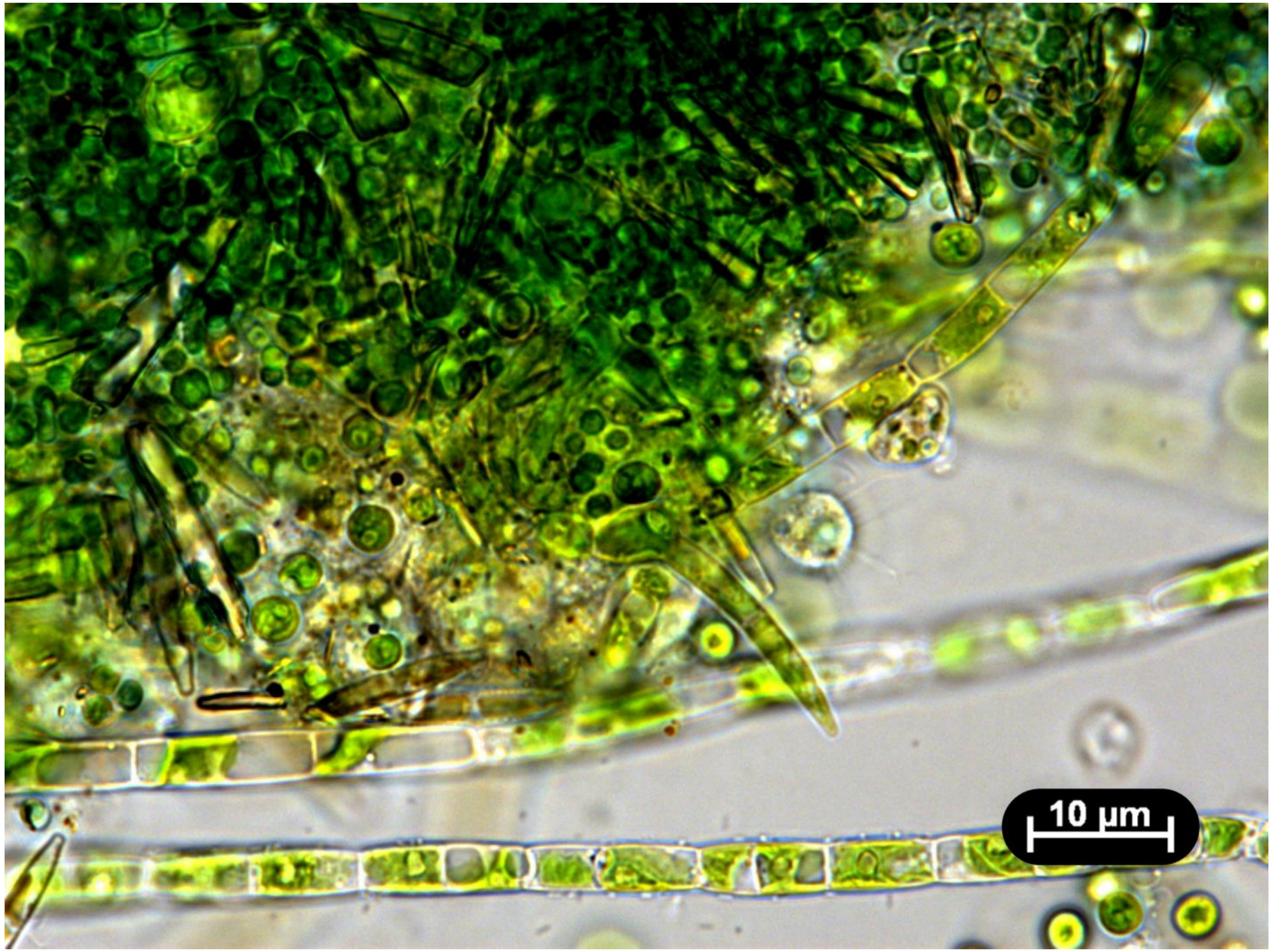

(**Image 4/7** of RAB microalgae consortium used in this study cultured in BBM medium)

RAB Microalgae Consortium

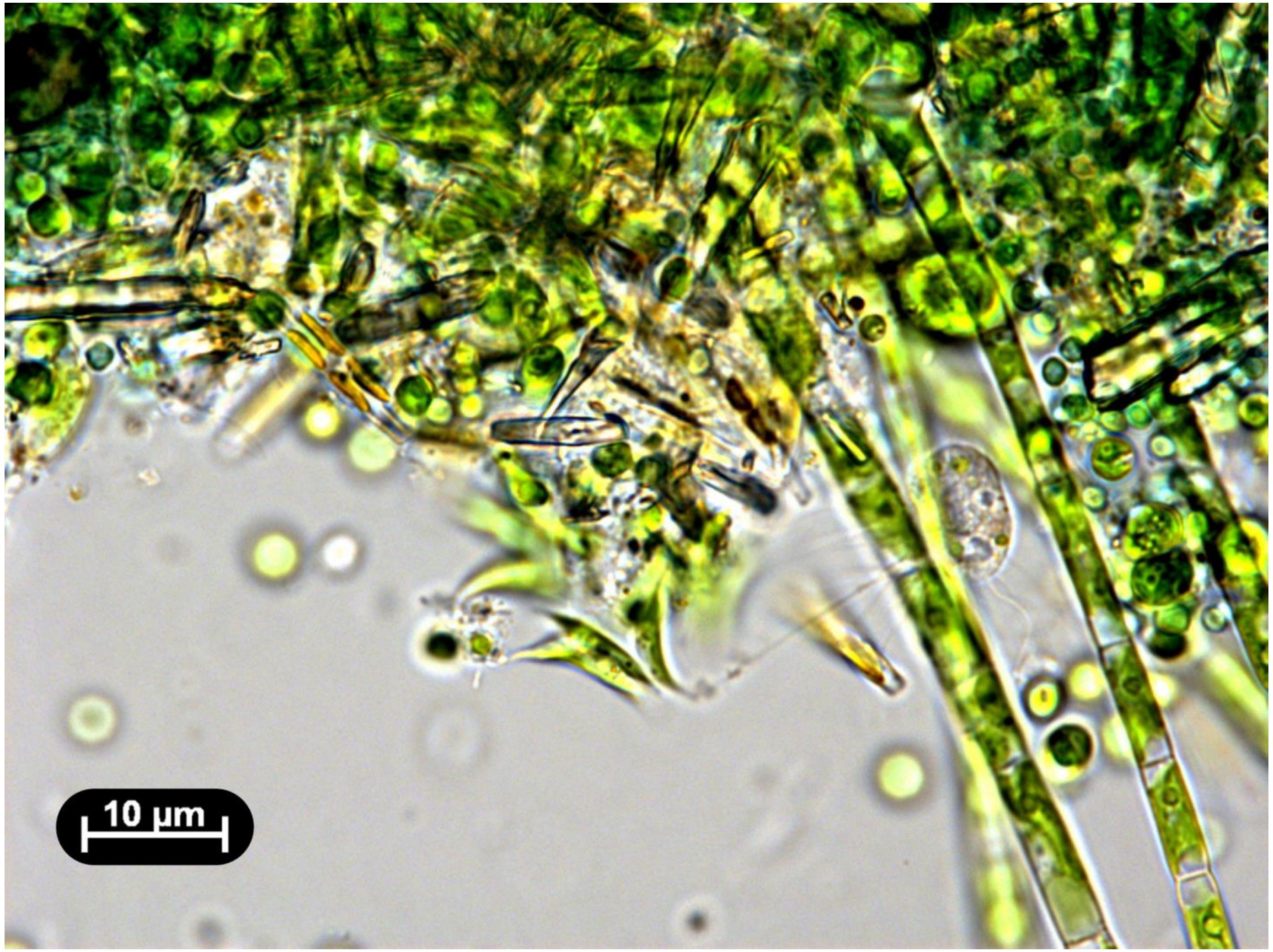

(**Image 5/7** of RAB microalgae consortium used in this study cultured in BBM medium)

RAB Microalgae Consortium

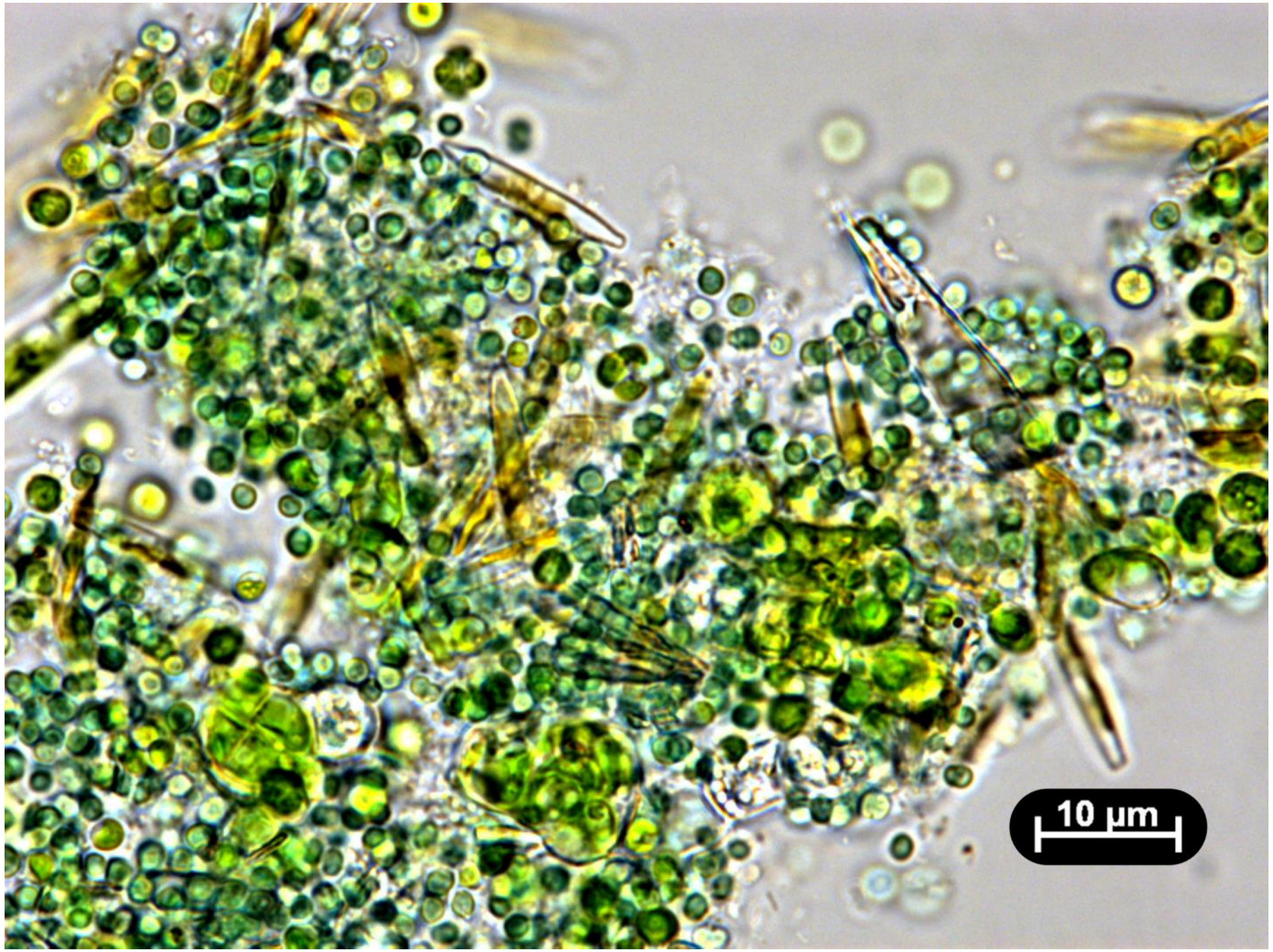

(**Image 6/7** of RAB microalgae consortium used in this study cultured in BBM medium)

RAB Microalgae Consortium

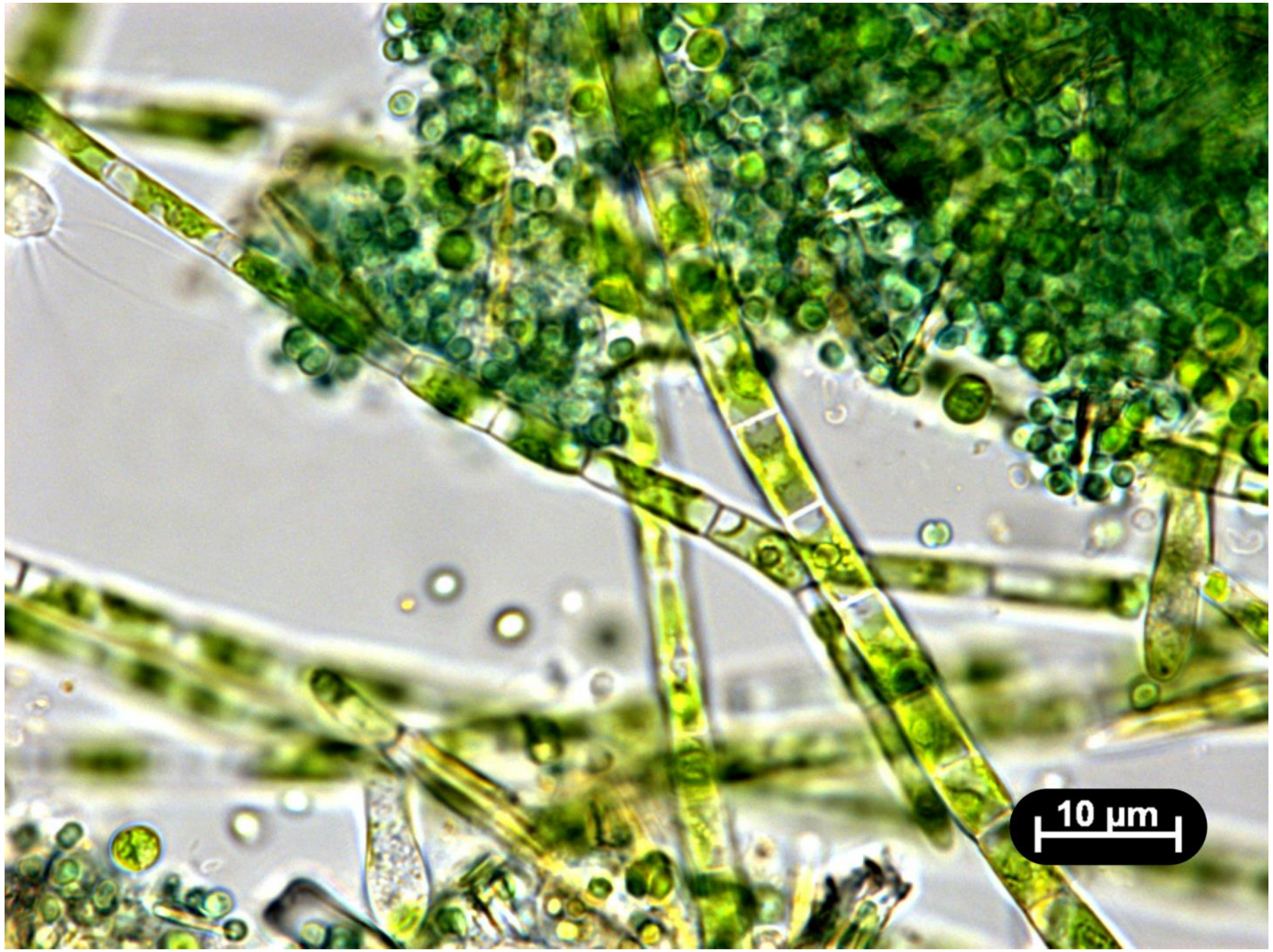

(**Image 7/7** of RAB microalgae consortium used in this study cultured in BBM medium)
