# Supplementary material for: Isolation of phosphorus-hyperaccumulating microalgae from revolving algal biofilm (RAB) wastewater treatment systems
Source: Front Microbiol. 2023 Jul 17;14:1219318. doi: 10.3389/fmicb.2023.1219318 (PMC10389661; doi:10.3389/fmicb.2023.1219318)
Supplement: Supplementary file 1 [file Data_Sheet_1.zip › Supplementary Material - Schaedig et al. (2023)/Supplementary Material.pdf]

Supplementary Material

Isolation of Phosphorus-Hyperaccumulating Microalgae from Revolving Algal Biofilm (RAB) Wastewater Treatment Systems

Eric Schaedig, Michael Cantrell, Chris Urban, Xuefei Zhao, Drew Greene, Jens Dancer, Michael Gross, Jacob Sebesta, Katherine J. Chou, Jonathan Grabowy, Martin Gross, Kuldip Kumar, and Jianping Yu

Table S1. Media compositions used to isolate and cultivate microalgae from RAB systems.

	Solid Media			Liquid Media
**Media Composition**	GWT-SE (Table S2)	BBM (Nichols and Bold, 1965)	BG-11 (Rippka et al., 1979)	BBM (Nichols and Bold, 1965)
**Concentration**	1-10X	1X only	1X only	1X only
**Media Modifications** (final concentration)	• 100 µg L^{-1} cyanocobalamin (BBM only)<br>• 5 g L^{-1} SiO_2*<br>• 100 µg mL^{-1} to 1 mg mL^{-1} sodium ampicillin†<br>• 1 µg mL^{-1} to 5 µg mL^{-1} carbendazim†			• 100 µg L^{-1} cyanocobalamin<br>• 10 g L^{-1} diatomaceous earth*<br>• 8.4 g L^{-1} sodium bicarbonate‡

*Included only for diatom isolates. Diatomaceous earth (Sigma) was added to liquid media, autoclaved at 121°C for 60 min, and clarified with a 0.22 µm PES bottle-top filter.
†Included only to axenize microalgal isolates.
‡ Included only for unicellular green algae and cyanobacteria. Dissolved in 1/10th volume of prepared medium and pH adjusted to 7.5-7.6 with HCl before re-addition.

Table S2. Chemical composition of GWT-SE synthetic secondary municipal wastewater effluent medium. “TKN” denotes Total Kjeldahl Nitrogen. “SLS” denotes sodium lignin sulfonate. Recipe modified from Voumard et al. (2019).

Substance	Supplier	Concentration (mg L^{-1})	Total Phosphorus (mg L^{-1})	Total Nitrogen (mg L^{-1})	NH_3 Nitrogen (mg L^{-1})	NO_2^-/NO_3^- Nitrogen (mg L^{-1})	TKN (mg L^{-1})
Beef extract	MP Biomedicals	1.8		0.216			0.216
Peptone	BD	2.7		0.405			0.405
Humic acid	Alfa Aesar	4.25		0.262			0.262
Tannic acid	Acros Organics	4.18					
SLS	Tokyo Chem. Ind.	2.4					
Gum arabic	Acros Organics	4.7		0.031			0.31
$(NH_4)_2SO_4$	Sigma	2.5		0.530	0.530		0.530
K_2HPO_4	Sigma-Aldrich	10	1.78				
$NaNO_3$	Sigma-Aldrich	36.5		9.876		9.786	
$MgSO_4$	Sigma	0.71					
**Total Concentration** (mg L^{-1})			1.78	11.32	0.53	9.88	1.44

Table S3. Primers used in this study.

Primer name	Target region & expected amplicon length	Sequence (5'→3')	Annealing temperature/ extension time	Reference
CYA359F*<br>CYA781R(a)<br>CYA781R(b)	16S rDNA V3-V4 region<br>446 bp	GGGGAATYTTCCGCAATGGG<br>GACTACTGGGGTATCTAATCCCATT<br>GACTACAGGGGTATCTAATCCCTTT	66°C / 20 sec	Nübel et al. (1997)
D512for*<br>D978rev	18S rDNA V4 region<br>466 bp	ATTCCAGCTCCAATAGCG<br>GACTACGATGGTATCTAATC	51°C / 20 sec	Zimmermann et al. (2011)
DIV4for*<br>DIV4rev3	18S rDNA V4 region<br>329 bp	GCGGTAATTCCAGCTCCAATAG<br>CTCTGACAATGGAATACGAATA	60°C / 20 sec	Visco et al. (2015)
ss5<br>ss3	18S rDNA gene<br>1823 bp	GGTGATCCTGCCAGTAGTCATATGCTTG<br>GATCCTTCCGCAGGTTCACCTACGGAAACC	72°C / 60 sec	Matsumoto et al. (2010)
mod-ss5*<br>mod-ss3*	-	CCTGCCAGTAGTCATATGCTTG<br>GGTTCACCTACGGAAACC	-	Modified from Matsumoto et al. (2010)

*Primers used to prime sequencing reactions. Mod-ss5/mod-ss3 are trimmed ss5/ss3 primers modified for Sanger sequencing priming and were not used for DNA amplification.

Table S3 Notes.

Microalgal isolates were identified by colony PCR and amplicon sequencing of the 18S/16S SSU rRNA genes using four previously published primer sets (**Table S3**). The CYA359F/CYA781R primer set was sufficient for the identification of all prokaryotic isolates. In contrast, multiple primer sets were needed for the identification of all eukaryotic isolates. The D512for/D978rev primer set was used initially for all eukaryotic isolates. This primer set efficiently amplified DNA from diatoms, and lowering the annealing temperature used with this primer set was sufficient to improve amplification in green algae (not shown). However, the hypervariable V3-V4 region amplified by D512for/D978rev was found to offer poor resolution in identifying many isolated green algae. Additionally, the primer set was found to be non-specific and amplified DNA from eukaryotic contaminants. Several isolates from RAB samples collected in Chicago, Illinois in February 2021 were inadvertently found to have been co-isolated with the common bacterivorous amoeba *Vermamoeba vermiformis.* The ss5/ss3 primer set was also non-specific but amplified the entirety of the 18S gene, offering sufficient sequence coverage to identify green algae that could not be identified by the V3-V4 region alone, given that the culture did not possess eukaryotic contaminants. However, the ss3/ss5 primer set did not amplify DNA from any isolated diatom strain. DIV4for/DIV4rev3 was adapted from the D512for/D978rev primer set by Visco et al. (2015) and similarly amplified the V3-V4 region but was not susceptible to contaminating eukaryotic DNA. Thus, this primer set was used to sequence and identify isolates for which eukaryotic contaminants were present.

Supplemental File S1. Microscopy image bank. Representative microscopy images of each unique strain isolated in this study and of the RAB microalgae consortium included in both the polyphosphate screening and bench-scale RAB testing. Available online.

Supplemental File S2. Isolated RAB strain information datasheet. Information on the microalgae isolated and characterized in this study, including information on the origin and isolation methods, polyphosphate screening methods and resulting data, and sequencing methods and resulting data for each strain. Available online.

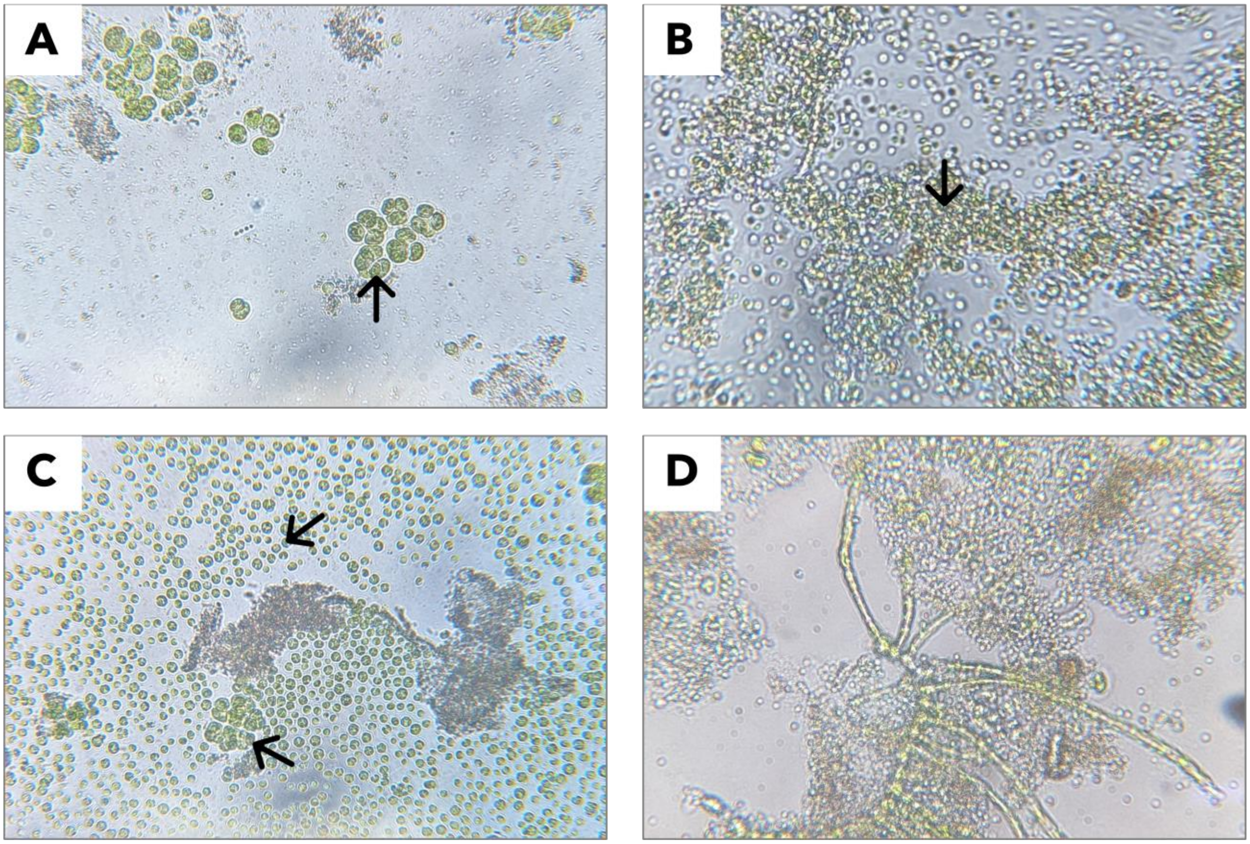

Figure S1. Microscopy images of the bench-scale RAB biofilms seven days after inoculation. Objective magnification at 100X. Arrows point to cells matching the morphology of the inoculated strain(s). **(A)** Biofilm inoculated with *Chlamydomonas pulvinata* TCF-48g. **(B)** Biofilm inoculated with *Chlorellaceae* sp. TCF-17g. **(C)** Biofilm inoculated with both *Chlamydomonas pulvinata* TCF-48g and *Chlorellaceae* sp. TCF-17g. **(D)** Biofilm inoculated with the RAB microalgae consortium from Slater, Iowa.
